# Menopausal timing and senescent–immune coupling in age-related lobular involution of the human breast: a longitudinal cohort study

**DOI:** 10.1016/j.ebiom.2026.106463

**Published:** 2026-08-31

**Authors:** Jaida C. Lue, Melody Stallings-Mann, Tanya Hoskin, Bryan M. McCauley, Robert A. Vierkant, Laura Pacheco-Spann, Jessica L. Fischer, Denice L. Gehling, Lisa R. Seymour, Darren J. Baker, Amy C. Degnim, Stacey J. Winham, Mark E. Sherman, Derek C. Radisky

**Affiliations:** a Department of Cancer Biology, Mayo Clinic, Jacksonville, FL, USA; b Department of Quantitative Health Sciences, Mayo Clinic, Rochester, MN, USA; c Department of Quantitative Health Sciences, Mayo Clinic, Jacksonville, FL, USA; d Department of Surgery, Mayo Clinic, Rochester, MN, USA; e Departments of Biochemistry and Molecular Biology and Pediatrics, Mayo Clinic, Rochester, MN, USA

**Keywords:** Age-related lobular involution, Menopause, Cellular senescence, Breast cancer, Spatial proteomics, Senescence-associated secretory phenotype

## Abstract

**Background:**

Incomplete postmenopausal breast involution leaves persistent epithelial-rich lobules and elevated breast density in about 40% of women and is associated with higher breast cancer risk, but why remodelling stalls remains unclear.

**Methods:**

We studied a longitudinal cohort of 81 women with paired benign breast biopsies (baseline age 45–55 years; follow-up 2–10 years), all with baseline NanoString transcriptomics and two-timepoint digital morphometry, and with multiplex immunofluorescence in spatial-imaging subsets (baseline n = 14–16 depending on panel; follow-up n = 14). A separate postmenopausal endpoint cohort (12 women: eight noninvoluted, four completely involuted), profiled by genome-wide expression array and multiplex immunofluorescence, defined the persistent-lobule phenotype.

**Findings:**

Noninvoluted postmenopausal tissue retained a proliferation-competent, tumour-associated epithelial state and showed immune accumulation at lobular boundaries with reduced access to p16^+^ (senescence-associated) epithelial foci. The same SASP and innate immune programmes that predicted slower involution across the menopausal transition predicted faster involution after menopause. Follow-up boundary CD45→p16 engagement was directionally consistent with this reversal in Pre→Post and Post→Post women. Spatial imaging resolved this reversal into a perimenopausal stall architecture and a postmenopausal clearance-associated architecture marked by direct CD16^+^ innate-effector engagement of p16^+^ epithelium; macrophage targeting provided convergent support (two-sided exact permutation interaction p = 0.0077).

**Interpretation:**

Menopausal timing conditions whether senescent–immune programmes couple to productive clearance or to spatially uncoupled surveillance and persistent risk-associated tissue. Biomarker interpretation should therefore be anchored to menopausal timing.

**Funding:**

Casey DeSantis Cancer Fund and US National Cancer Institute.

**Research in context**

**Evidence before this study**

Incomplete postmenopausal breast involution affects approximately 40% of women and is among the strongest tissue-based predictors of breast cancer risk, yet why remodelling stalls has remained unexplained. Prior cross-sectional studies established that ageing breast tissue accumulates p16^+^ senescent epithelial cells, a SASP, and immune infiltration, and linked these features to elevated cancer risk. However, none anchored findings to menopausal timing, none resolved the spatial relationship between p16^+^ (senescence-associated) epithelial foci and immune effectors at the lobule scale, and none tested whether senescent–immune programmes reverse their association with tissue fate across the menopausal transition.

**Added value of this study**

Using paired benign breast biopsies across midlife in 81 women, integrated with NanoString transcriptomics, longitudinal morphometry, and lobule-scale multiplex imaging, we show that menopausal timing conditions how senescent–immune programmes relate to breast tissue fate. The same EGFR–STAT3–innate hub programmes that associate with slower remodelling before menopause associate with faster regression after menopause, with convergent support across transcriptomic, spatial, and macrophage-targeting analyses. Spatial imaging maps this reversal to two architectures: a perimenopausal stall state in which immune cells accumulate but remain uncoupled from senescent epithelial foci, and a postmenopausal clearance state consistent with productive innate engagement of senescent epithelium.

**Implications of all the available evidence**

Incomplete breast involution is better understood as an execution-limited tissue state in which immune effectors are present but not consistently engaging p16^+^ epithelial targets. SASP and innate immune biomarkers should be interpreted relative to menopausal timing; pooling women across the menopausal transition may obscure or reverse biologically meaningful associations. The menopausal transition therefore emerges as a biologically motivated window for future studies aimed at promoting senescent-cell clearance or preventing persistence of risk-associated postmenopausal breast tissue.

## Introduction

A central question in cancer risk associated with tissue ageing is what determines whether immune programmes that accumulate at sites of cellular senescence drive clearance or sustain a persistence-prone state; where that endpoint tilts towards persistence, senescent cells evade immune clearance, accumulate and drive chronic inflammation, clonal selection, and malignant transformation.^1^, ^2^, ^3^, ^4^ Age-related lobular involution (ARLI) is a prolonged regression of terminal duct lobular units that unfolds across midlife and the menopausal transition. In most women, this remodelling reduces breast density and is associated with lower breast cancer risk. In approximately 40% of postmenopausal women, however, involution remains incomplete, leaving epithelial-rich lobules associated with a denser, risk-associated breast state.^5^, ^6^, ^7^, ^8^ Why involution progresses in some women but stalls in others has remained unclear.

Recent cross-sectional studies have begun to define the cellular landscape of the ageing human breast, showing menopause-associated loss of lobules, declining cellularity, and inflammatory immune remodelling.^9^ These atlases establish the population-level backdrop for breast ageing, but they do not explain why remodelling outcomes diverge across women. Tissue ageing is also inherently spatially heterogeneous: remodelling unfolds asynchronously across repeating microanatomical units, producing within-tissue mosaics of persistence and regression.^10^, ^11^, ^12^, ^13^, ^14^, ^15^, ^16^, ^17^ This means that bulk inflammatory or senescence signatures need not have a single directional relationship with remodelling outcomes. Instead, the spatial organisation of senescent and immune cells within individual lobules, whether effectors are positioned to engage senescent targets or are present but held at a distance, may determine local tissue fate independently of overall cell abundance.

A similar persistence-versus-resolution problem has been described in other renewing tissues, including intestine, endometrium, and liver, where chronic stress or hormonal withdrawal can preserve residual functional units rather than eliminate them outright.^18^, ^19^, ^20^, ^21^, ^22^, ^23^, ^24^ In the intestine, endometrium, liver, skeletal muscle, and haematopoietic system, tissues do not simply exhaust their regenerative reserves. Instead, immune-supported programmes hold residual tissue-sustaining cells in a poised, survival-ready state. This confers resilience, but when it is chronically maintained, or when clearance is incomplete, it can become a substrate for clonal selection, chronic inflammation, and malignant transformation.^19^, ^20^, ^21^ We reasoned that persistent breast lobules may represent an analogous state: not a passive remnant of incomplete regression, but a proliferation-competent epithelial compartment sustained by ongoing senescent–immune interactions whose outcome is conditioned by menopausal timing.

This hypothesis is biologically plausible because p16^INK4A^-positive epithelial cells accumulate across midlife,^25^^,^^26^ and senescent cells communicate through a senescence-associated secretory phenotype (SASP) that reshapes local immune microenvironments and recruits innate effectors.^1^, ^2^, ^3^ When surveillance is effective, senescent cells can be removed and tissue remodelling can proceed; when clearance is incomplete or spatially miscoordinated, senescent cells may persist together with chronic inflammatory and pro-tumourigenic tissue states.^1^^,^^4^ Because ARLI spans years of systemic physiologic change and tissue-local immune remodelling,^27^, ^28^, ^29^, ^30^, ^31^ menopause may condition whether the same senescence-linked inflammatory programmes are associated with persistence during the transition or with clearance after menopause.

Here, we aimed to test this model using three linked human datasets. First, we analyse a postmenopausal endpoint cohort spanning extremes of ARLI (noninvoluted versus completely involuted benign tissue) using Illumina cDNA-mediated annealing, selection, extension, and ligation (DASL) expression profiling and multiplex immunofluorescence to define the molecular and spatial phenotype of persistent postmenopausal lobules. Second, we study a longitudinal cohort of 81 women with paired midlife benign biopsies (baseline age 45–55 years; follow-up 2–10 years) profiled at baseline by NanoString transcriptomics to relate senescence-linked programmes to subsequent involution pace and to determine whether those associations are modified by menopausal timing. Third, we use paired multiplex spatial imaging in a deeply profiled subset to map the lobule-scale microarchitectures associated with stalled versus resolving remodelling. Together, these analyses show that senescence-linked inflammatory and innate programmes reverse their association with involution pace across menopause and that this reversal maps to distinct spatial organisations consistent with perimenopausal stall and postmenopausal clearance states.

## Methods

### Ethics

Human breast tissue and associated clinical data were obtained from the Mayo Clinic tissue repository and derived from the Mayo Clinic Benign Breast Disease cohort^32^ under Mayo Clinic Institutional Review Board approval (IRB 78–87) and in adherence to the 2024 Declaration of Helsinki ethical principles. Written informed consent was obtained from all participants. Enrolment/biopsy accrual occurred from August 1967 to May 2001; laboratory analyses (NanoString and DASL profiling and multiplex immunofluorescence) were conducted between July 2010 and May 2026. All samples and associated data were deidentified before analysis, and the study complied with relevant ethical regulations. Biopsies were obtained for clinical indications, including abnormal imaging findings or palpable abnormalities, during benign breast disease evaluation. Women with concurrent carcinoma were not included.

### Study design and cohorts

The study integrated two complementary human tissue datasets. The first was a postmenopausal endpoint cohort used to define the senescence–proliferation–immune phenotype of persistent versus fully regressed postmenopausal lobules. The second was a longitudinal midlife cohort with paired benign biopsies used to quantify involution pace and to test whether baseline molecular and spatial programmes were associated with subsequent remodelling in a menopause-dependent manner. The longitudinal cohort (n = 81) provided baseline NanoString transcriptomics and two-timepoint morphometry, with multiplex immunofluorescence in imaging subsets; the postmenopausal endpoint cohort (n = 12) provided genome-wide expression-array and multiplex-immunofluorescence data. Participants self-identified as female.

### Postmenopausal endpoint cohort

Postmenopausal benign biopsies were selected to represent extremes of age-related lobular involution (ARLI) and were classified by breast pathologists as noninvoluted or completely involuted using established terminal duct lobular unit (TDLU) involution criteria. The endpoint cohort comprised 12 women: eight with noninvoluted and four with completely involuted tissue. All women contributed benign, carcinoma-free lobular tissue. For histology-anchored immunohistochemistry (IHC) and multiplex immunofluorescence (MxIF), regions of interest (ROIs) corresponding to individual TDLUs were annotated on haematoxylin and eosin (H&E)-stained sections by a breast pathologist before marker review and were propagated to serial sections. Annotation was restricted to benign lobules, and regions containing atypia, other lesions, or lobules immediately adjacent to lesions were excluded. For expression profiling, RNA was extracted from lobule-enriched regions macrodissected from the same formalin-fixed paraffin-embedded (FFPE) blocks.

### Longitudinal pace cohort

The longitudinal cohort comprised 81 women who had a baseline benign breast biopsy between ages 45 and 55 years with FFPE RNA profiled on NanoString Technologies Human PanCancer IO 360 (cat. no. XT-CSO-HIO360-12) or Breast Cancer 360 (cat. no. XT-CSO-BC360-2-12) panels, together with a subsequent benign biopsy obtained 2–10 years later. Baseline and follow-up biopsies underwent central pathology review to confirm benign status and tissue adequacy. Digital morphometry was performed at both time points to quantify longitudinal changes in lobular structure. The endpoint and longitudinal cohorts sample the same age-related lobular involution continuum: the endpoint cohort represents its extremes: noninvoluted versus completely involuted postmenopausal lobules, whereas the longitudinal cohort represents its trajectory, summarised by the continuous involution pace index defined below.

### Imaging subsets

Baseline MxIF was performed on a subset of longitudinal-cohort biopsies selected to span the involution pace distribution from fast to stagnant remodelling. Follow-up biopsies from the same cohort were also analysed by MxIF in a paired subset to quantify leucocyte organisation and p16^+^-target engagement at the outcome time point. Analyses requiring menopausal timing or trajectory classification were restricted to women with known registry menopause age. For the baseline spatial architecture analyses, each woman contributed a single baseline imaging biopsy and was classified by her baseline menopausal status; this single-timepoint status is distinct from the longitudinal menopausal trajectory (Pre→Post/Post→Post) used in the effect-modification models. For the CD68/CD206/p16 macrophage panel (a custom MxIF panel; antibody sources, catalogue numbers, and RRIDs are given under Multiplex immunofluorescence staining and imaging), 16 baseline biopsies were stained; two women lacked registry menopause age and were therefore excluded from menopause-conditioned analyses, leaving 14 evaluable women (Pre→Post n = 8; Post→Post n = 6).

### Experimental procedures

#### Histologic assessment of ARLI and digital morphometry

H&E-stained sections were reviewed by a breast pathologist to classify ARLI on an ordinal scale as noninvoluted, partial involution, or complete involution using established criteria. For quantitative digital morphometry, representative TDLUs were pathologist-annotated for each biopsy, typically up to 10 per specimen when present. For MxIF, all evaluable pathologist-annotated benign TDLUs were retained, yielding 310 lobules across the 14-woman baseline subset (approximately 22 lobules per woman). Area and acini number per lobule were measured for each annotated TDLU. Each biopsy was summarised by the median lobule area and median acini count across annotated lobules.

#### Definition of involution pace, menopausal timing, and trajectory strata

To compare women with different inter-biopsy intervals, morphometric change was converted to per-year rates on the log scale. For each woman with inter-biopsy interval Δt in years, pace_area was defined as [log(median area_baseline) − log(median area_follow-up)]/Δt and pace_acini was defined as [log(median acini_baseline) − log(median acini_follow-up)]/Δt. The involution pace index was defined as the mean of pace_area and pace_acini, such that larger values indicate faster structural regression. Pace was z-scored across women (pace_z) where indicated. The pace index is thus a continuous summary of the same epithelial-content reduction that the ARLI scale captures categorically. Graded ordinal ARLI categories (noninvoluted/partial/complete) were not separately scored for the longitudinal cohort, which used a binary involuted/noninvoluted pathologist classification; baseline biology is therefore related to this continuous morphometric index rather than to ordinal categories.

Registry-reported age at menopause was used to anchor each biopsy relative to the menopause boundary. Years-from-menopause (yfm) was defined as age at biopsy minus menopause age, such that negative values indicate premenopausal biopsies and positive values indicate postmenopausal biopsies. Baseline yfm was used as a continuous menopausal–physiology axis in regression and interaction models. Menopausal trajectory strata were defined using yfm at both time points: Pre→Post for women premenopausal at baseline and postmenopausal at follow-up, and Post→Post for women postmenopausal at both biopsies. Pre→Pre intervals were excluded from primary trajectory-stratified analyses. Women missing menopause age were retained for analyses that did not require yfm, but were excluded from menopause-anchored and trajectory-based models.

#### RNA isolation from FFPE tissue

FFPE sections were scraped or macrodissected to enrich for benign lobular regions. Total RNA was isolated using the RNeasy FFPE Kit (Qiagen, cat. no. 73504) according to the manufacturer's instructions.

#### Illumina DASL expression profiling in the postmenopausal endpoint cohort

As RNA from FFPE tissue is fragmented, expression profiling of the postmenopausal endpoint cohort was performed using the Illumina Whole-Genome DASL HT Assay (Illumina WG-DASL HT) and processed using Illumina GenomeStudio 2011.1. Samples were retained only if at least 80% of probes had Illumina detection p values < 0.05. Probes with detection p ≥ 0.05 in more than 20% of samples were removed. Probe intensities were background-subtracted, log2 transformed, and quantile normalised.

Differential expression between noninvoluted and completely involuted groups was assessed probe-by-probe using Welch's two-sample t test. Log2 fold-change was calculated as mean(log2 expression)_noninvoluted − mean(log2 expression)_complete. Benjamini–Hochberg false discovery rate (FDR) was calculated across all tested probes. For sample-level structure, principal component analysis (PCA) was performed on the 500 most variable genes from the genome-wide DASL feature set after filtering, using gene-wise z-scored expression across samples. Restricting the projection to the most variable genes focuses the analysis on informative, coordinately varying transcripts and reduces the influence of the many low-expression, low-variance probes typical of FFPE-derived data; the value of 500 lies within the range commonly used for high-variance feature selection in expression PCA, large enough to capture coordinated expression programmes yet small enough to exclude the low-variance tail. A composite proliferation index was calculated for each sample as the mean of gene-wise z scores for canonical proliferation genes present on the DASL array (*TOP2A, UBE2C, TYMS, CENPA, KIF11, KIF20A, CDCA8, CDCA3, CENPM, CENPK, and CCNB1*). To place the noninvoluted transcriptional programme in tumour context, the noninvoluted-high gene set (nominal two-sided Welch's two-sample t-test p < 0.05 and positive fold-change) was compared with phenotype-aligned gene sets derived from public breast tumour microarray contrasts^6^^,^^33^, ^34^, ^35^, ^36^, ^37^, ^38^, ^39^, ^40^, ^41^, ^42^, ^43^, ^44^, ^45^, ^46^, ^47^ using Illumina Correlation Engine v2.5.0.^48^

#### NanoString preprocessing, harmonisation, and programme-score construction

Baseline expression profiling in the longitudinal cohort used NanoString nCounter Human PanCancer IO 360 (NanoString Technologies, cat. no. XT-CSO-HIO360-12) or Breast Cancer 360 (cat. no. XT-CSO-BC360-2-12) panels. Normalised counts were transformed as log1p(x) = log(1 + x). Genes present on both panels were aligned by gene symbol, duplicated symbols were collapsed by averaging log1p values, and genes absent from a panel were treated as missing. Composite signature scores were constructed by z-scoring each gene across the cohort and averaging across genes within a prespecified programme, requiring coverage of at least half of member genes with a minimum of two genes.

A priori panel-constrained programmes represented four biological axes central to the study: EGFR signalling, senescence/SASP and inflammatory signalling, innate/myeloid immune engagement, and proliferation. Explicitly defined programmes included an EGFR-axis score (*EREG, AREG, EGFR*), a myeloid activation score (including canonical myeloid markers such as *CD14, CSF1R, and ITGAM*), and a proliferation score (*MKI67, TOP2A, PCNA, MCM2, CCNB1, CCNE1, CDC20, AURKA, BIRC5, MAD2L1, UBE2C, CENPF, and CCNA2*). Gene submodules included SASPcore, Hub12, and related STAT3/cytokine and EGFR-field. The STAT3 axis was defined a priori as a minimal pathway spine (*IL6 → JAK1 → STAT3 → SOCS3*), reflecting the genetically established role of STAT3 as the central regulator of mammary epithelial apoptosis and lobular involution.^49^, ^50^, ^51^, ^52^, ^53^ Single-gene anchors, including *EREG, FCGR3A, and FCGR3B*, were analysed separately.

Within trajectory strata, women were also divided into pace tertiles and the top and bottom tertiles were contrasted as FAST versus STAGNANT groups. For each gene with at least four non-missing values per group, log2 fold-change was computed from log1p expression and significance was assessed using Welch's two-sample t test. These ranked gene statistics were used for preranked Hallmark gene set enrichment analysis.

#### Immunohistochemistry

IHC was performed on 5 μm FFPE sections using standard deparaffinisation, citrate antigen retrieval (pH 6.0; Agilent S1699), HRP-DAB detection (Agilent K4001; antimouse secondary), and haematoxylin (Epridia 6765006) counterstaining. Whole slides were scanned on anAperio ScanScope AT2 slide scanner (Leica Biosystems, Nussloch, Germany) using a 20 × objective and analysed in QuPath v0.6.0. Lobule ROIs defined on H&E were applied to serial IHC sections. For each ROI, acini count, ROI area, and the number of positive epithelial nuclei were recorded. Marker abundance was expressed as positive epithelial nuclei per mm^2^ of ROI area. IHC analyses focused on TOP2A and Ki67 as complementary proliferation markers.

#### Multiplex immunofluorescence staining and imaging

Opal-based MxIF (Akoya Biosciences) was performed using iterative rounds of primary antibody incubation, tyramide signal amplification, and heat-mediated stripping between cycles, followed by DAPI counterstaining. All Opal fluorophores and DAPI were from Akoya Biosciences; DAPI was cat. no. FP1490. Slides were scanned on a Vectra Polaris Automated Quantitative Pathology Imaging System (Akoya Biosciences, Marlborough, MA, USA). Spectral unmixing and autofluorescence subtraction were performed in InForm v3.1.0.

The postmenopausal senescence–proliferation–immune (“triad”) panel comprised Ki67 (DAKO M7240, RRID:AB_2142367; 1:200; Opal 520, 1:100), p16 (Santa Cruz SC-56330, RRID:AB_785018; 1:600; Opal 570, 1:100), TOP2A (Invitrogen, recombinant rabbit monoclonal clone SY27-00, cat. no. MA5-32096; RRID:AB_2809389; 1:1200; Opal 620, 1:100), CD45 (Abcam Ab40763, RRID:AB_726545; 1:300; Opal 690, 1:100), pan-cytokeratin (DAKO M3515, RRID:AB_2132885; 1:150; Opal 780, 1:25), and DAPI (Akoya FP1490, 1:10).

Longitudinal baseline panel 1 comprised p16 (Santa Cruz SC-56330; RRID:AB_785018; 1:400; Opal 480, 1:150), Ki67 (DAKO M7240; RRID:AB_2142367; 1:100; Opal 520, 1:100), CD45 (Abcam Ab40763; RRID:AB_726545; 1:300; Opal 570, 1:100), EREG (Santa Cruz SC-376284; RRID:AB_11014189; 1:100; Opal 690, 1:50), pan-cytokeratin (DAKO M3515; RRID:AB_2132885; 1:100; Opal 780, 1:25), and DAPI (Akoya Biosciences FP1490; 1:10).

Longitudinal baseline panel 2 comprised Ki67 (DAKO M7240; RRID:AB_2142367; 1:100; Opal 480, 1:150), phospho-STAT3 (Abcam AB76315; RRID:AB_1658549; 1:100; Opal 520, 1:100), p16 (Santa Cruz SC-56330; RRID:AB_785018; 1:400; Opal 570, 1:50), CD16A (Abcam AB246222; RRID:AB_2910166; 1:500; Opal 620, 1:50), EREG (Santa Cruz SC-376284; RRID:AB_11014189; 1:100; Opal 690, 1:50), pan-cytokeratin (DAKO M3515; RRID:AB_2132885; 1:100; Opal 780, 1:25), and DAPI (Akoya Biosciences FP1490; 1:10).

Longitudinal baseline panel 3 comprised CD206 (Abcam AB64693; RRID:AB_1523910; 1:200; Opal 520, 1:100), p16 (Santa Cruz SC-56330; RRID:AB_785018; 1:600; Opal 570, 1:100), CD68 (Abcam AB125212; RRID:AB_10975465; 1:500; Opal 690, 1:100), pan-cytokeratin (DAKO M3515; RRID:AB_2132885; 1:150; Opal 780, 1:25), and DAPI (Akoya Biosciences FP1490; 1:10).

The longitudinal follow-up panel comprised Ki67 (DAKO M7240; RRID:AB_2142367; 1:100; Opal 520, 1:100), p16 (Santa Cruz SC-56330; RRID:AB_785018; 1:600; Opal 570, 1:100), CD45 (Abcam Ab40763; RRID:AB_726545; 1:300; Opal 690, 1:100), pan-cytokeratin (DAKO M3515; RRID:AB_2132885; 1:150; Opal 780, 1:25), and DAPI (Akoya Biosciences FP1490; 1:10). Full details in Table S1.

#### Antibody validation

Antibody specificity was established in a two-stage workflow. Each antibody was first validated by chromogenic immunohistochemistry (IHC) on formalin-fixed paraffin-embedded tissue using positive and negative control tissues appropriate to the target, with staining distribution and subcellular localisation confirmed against manufacturer validation data and the Human Protein Atlas for the indicated application and species. For multiplex immunofluorescence (MxIF), each antibody was then optimised in monoplex to reproduce the localisation and intensity distribution of its validated IHC staining before incorporation into the panel, and singleplex and multiplexed signal were confirmed to be concordant for every marker. Because tyramide signal amplification deposits fluorophore covalently and signal can be sensitive to position within the staining series, the antibody–Opal pairing and cycle order were optimised empirically to preserve the validated monoplex pattern of each marker and to protect lower-abundance or interpretation-critical markers. Fluorophore assignments were chosen to maximise spectral separation between markers, and, to further guard against misinterpretation of any residual crosstalk, spectrally adjacent fluorophores were assigned to markers occupying distinct subcellular compartments (e.g., nuclear versus membranous) as much as possible, so that closely-spaced signals remained spatially resolvable and separable during unmixing. Spectral unmixing with autofluorescence subtraction was performed in InForm v3.1.0 using single-stain and unstained control slides. Marker identity was further supported by expected compartmental localisation (nuclear p16, Ki67 and TOP2A; nuclear pSTAT3 upon activation; epithelial pan-cytokeratin; membranous/myeloid CD45, CD16, CD68 and CD206). Marker-positivity thresholds for IHC and MxIF were established per staining batch using control tissues and visual inspection, and were applied uniformly within batch without reference to involution pace, menopausal trajectory, or group assignment.

#### Segmentation, compartment assignment, phenotyping, and abundance features

Whole-slide multispectral images were segmented into single cells in InForm v3.1.0 using DAPI-defined nuclei and the pan-cytokeratin mask. Cells were assigned to epithelial or stromal compartments based on the pan-cytokeratin mask. Lobule ROIs were defined by pathologist annotation. All spatial metrics were computed at the level of individual annotated benign TDLUs (lobule-level ROIs) and then summarised within woman, with women as the unit of inference; the noninvoluted/completely involuted designation refers to the donor-level ARLI phenotype from which lobules were drawn, whereas all proximity and density measurements were made per lobule. Within-biopsy heterogeneity in lobule involution stage is therefore retained in the lobule-level data rather than reduced to a single per-biopsy value. Marker positivity thresholds were set per staining batch using control tissues and visual inspection and were applied uniformly within batch without reference to involution pace or menopausal trajectory. Epithelial marker fractions (e.g., EREG^+^, pSTAT3^+^, p16^+^, Ki67^+^, CD16^+^) are single-marker fractions, computed independently for each marker as the number of marker-positive epithelial cells divided by the total epithelial cells in the ROI; they are not co-expression (double-positive) fractions. All p16-based endpoints were computed only on epithelial p16^+^ cells, defined as p16^+^ nuclei coincident with the pan-cytokeratin^+^ epithelial segmentation mask; p16^+^ objects outside the epithelial compartment were excluded from all analyses. Spatial relationships between markers are quantified separately by nearest-neighbour and field-embedding metrics.

Per-lobule abundance features included compartment-specific cell densities (cells/mm^2^) and epithelial marker fractions, calculated as the number of marker-positive epithelial cells divided by the total epithelial cell count in the ROI. Woman-level summaries were constructed by aggregating lobule-level values within woman, using prespecified means, maxima, or fractions depending on the endpoint. Within-breast mosaicism (HubPatchiness) was defined as the mean of the z-scored between-lobule variances in epithelial EREG-positive fraction and epithelial phospho-STAT3-positive fraction among women with at least two evaluable lobules for each component. These two fields, the EGFR ligand field and the STAT3 signalling field central to the hub model, were specified a priori, and the metric was fixed before relating it to involution pace.

#### General nearest-neighbour framework and CSR-adjusted enrichment

Nearest-neighbour distances were computed within each lobule ROI from inForm-exported cell centroids using Euclidean distance in μm. For an ordered source→target pair (A→B), each source cell of phenotype A was assigned the distance to its nearest eligible target cell of phenotype B within the same ROI, subject to any specified compartment restrictions. For each A→B pair and radius r, the contact fraction CF_r(A→B) was defined as the fraction of A-source cells whose nearest eligible B target lay within radius r. ROIs were eligible for an A→B endpoint only if they contained at least one eligible source cell after phenotype and compartment filtering. ROIs with at least one source but zero eligible targets were retained and assigned CF_r = 0 for all radii, whereas ROIs with zero sources were excluded for that pair.

Because cells are more likely to lie near a common target simply when that target is abundant, observed contact fractions were compared with the contact fraction expected if target cells were randomly distributed at the same ROI-specific density. Under this complete-spatial-randomness (CSR) model, with target density λ, CSR_exp(r) = 1 − exp(−λπr_mm^2^), where radii measured in μm were converted to mm. CSR-adjusted enrichment was defined as EXCESS(r) = CF_r(obs) − CSR_exp(r); a positive EXCESS value therefore indicates greater proximity than expected from target abundance alone. Radii of 5, 10, 15, 20, 25, and 30 μm captured immediate adjacency and the periacinar halo. For compartment-resolved analyses, EXCESS was also calculated within epithelium→epithelium, stroma→stroma, and stroma→epithelium source–target families and averaged within Near (0–15 μm), Halo (15–45 μm), and Far (45–100 μm) bands to generate ΔG summary features.

#### Postmenopausal triad spatial endpoints

For postmenopausal triad analyses, boundary-adjacent epithelial source cells were defined as epithelial cells located within 20 μm of the epithelium–stroma interface. For p16→CD45 and TOP2A→CD45 endpoints, the nearest CD45 target was required to be stromal based on tissue-compartment labels. Boundary contact fractions were computed as the fraction of boundary-adjacent epithelial sources whose nearest stromal CD45-positive neighbour lay within each specified radius. Distance-to-boundary gradients were computed by binning source-cell distance to the epithelium–stroma boundary in 2 μm increments from 0 to 80 μm, classifying whether the nearest CD45-positive neighbour was stromal or epithelial, averaging within women and bin, and then averaging across women. Reciprocal source→target orientations were evaluated where indicated to ensure that conclusions were not artefacts of source/target assignment.

#### Follow-up leucocyte–senescence interface endpoints

Follow-up interface analyses quantified leucocyte access to epithelial p16-positive targets at the outcome time point. Boundary stromal CD45-positive source cells were defined as stromal CD45-positive cells within 20 μm of the epithelial–stromal interface based on the inForm distance-to-boundary field. For each eligible lobule, CF_r(CD45→p16) was computed as the fraction of boundary stromal CD45-positive source cells whose nearest epithelial p16-positive neighbour was within radius r. The primary endpoint used r = 20 μm, and Complete20 was defined as CF_20 (CD45→p16) = 1.0, corresponding to uniform target access among all boundary leukocytes in the lobule. Lobules with zero boundary stromal CD45-positive sources were ineligible for Complete20. Density-adjusted coupling across multiple radii was summarised with EXCESS(r) and area-under-the-curve summaries.

Local cell aggregation (boundary CD45^+^ clustering; epithelial pSTAT3^+^ and p16–CD16 clustering) was quantified with DBSCAN (ClusterFrac), implemented with the dbscan v1.2.5 package in R v4.6.1. DBSCAN was chosen because the number of aggregates per lobule is not known a priori, aggregates may be spatially irregular, and isolated cells can be labelled as noise. Robustness to parameter choice was assessed across ε values of 8–14 μm and minPts values of 4–6, and clustering conclusions were compared with the parameter-free Complete20 adjacency endpoint. Boundary stromal CD45-positive cells were defined as stromal CD45-positive cells whose nearest pan-cytokeratin-positive epithelial cell was within 20 μm. DBSCAN was applied within each ROI to the x,y centroid coordinates of boundary CD45-positive cells using ε = 12 μm and minPts = 5. ClusterFrac was the fraction of boundary CD45-positive cells assigned to any non-noise cluster. Lobules with fewer than minPts boundary CD45-positive cells, including zero, were assigned ClusterFrac = 0 to retain all lobules for modelling. Parameter stability was evaluated across ε values of 8, 10, 12, and 14 μm and minPts values of 4, 5, and 6.

#### Residual-lobule and denominator-sensitivity analyses

To test whether follow-up interface behaviour was concentrated within the epithelial-rich lobules most likely to persist, residual lobules were defined a priori as the top tertile of log(1 + pan-cytokeratin-positive epithelial cell count) across all follow-up lobules. Within-woman residual lobules were defined analogously as the top tertile of log(1 + pan-cytokeratin-positive epithelial cell count) within each woman. Tail analyses summarised each woman by the fraction of within-woman residual lobules meeting Complete20. Because Complete20 can be unstable when the number of boundary stromal CD45-positive sources is small, denominator-sensitivity analyses were performed by refitting models after requiring minimum source counts of at least 1, 5, or 10 cells per lobule.

#### Baseline EREG- and phospho-STAT3-centred spatial endpoints

For baseline p16/Ki67/EREG/pSTAT3/CD16 panels, nearest-neighbour distances were computed within each lobule ROI using the same Euclidean framework. Distances from Ki67-positive epithelial sources to the nearest epithelial p16-positive neighbour were stratified by EREG or phospho-STAT3 coexpression status. CF10 was defined as the fraction of source cells with a target within 10 μm. An EREG-biased adjacency index was defined as ΔCF10 = CF10 (Ki67^+^EREG^+^ → p16) − CF10 (Ki67^+^EREG^−^ → p16).

Field embedding was summarised at the lobule level as the median nearest-neighbour distance from proliferative cells lacking the field marker to the nearest field-positive epithelial cell. For EREG embedding, source cells were Ki67^+^EREG^−^ epithelial cells and targets were EREG-positive epithelial cells. For phospho-STAT3 embedding, source cells were Ki67^+^phospho-STAT3^−^ epithelial cells and targets were phospho-STAT3-positive epithelial cells. “Hot” or embedded lobules were defined using prespecified cohort-wide percentile thresholds, typically the lowest quintile of the eligible lobule-median distance distribution within the relevant imaging dataset. Thresholds were computed once from the full eligible lobule distribution and then applied uniformly in all downstream models, including menopause-stratified analyses. Ki67-rich sensitivity analyses restricted attention to lobules containing at least five Ki67^+^EREG^−^ epithelial source cells.

#### Stall-hub and clearance-hub classification

Stall and clearance implementations were defined in the baseline p16/Ki67/EREG/phospho-STAT3/CD16 panel using prespecified distance and abundance criteria. For each lobule, median nearest-neighbour distances were computed for p16-positive epithelium to the nearest phospho-STAT3-positive epithelium, epithelial CD16-positive cells to the nearest phospho-STAT3-positive epithelium, and stromal CD16-positive cells to the nearest phospho-STAT3-positive epithelium. For the innate arm, d_CD16_min→pSTAT3 was defined as the smaller of the epithelial and stromal CD16→phospho-STAT3 median distances where both were available. A lobule was designated phospho-STAT3-hot if d_p16→pSTAT3 was at or below the cohort-wide 20th percentile of eligible lobules, and CD16–phospho-STAT3-hot if d_CD16_min→pSTAT3 was at or below the corresponding 20th percentile. Stall hubs were defined as lobules meeting both criteria simultaneously. Lobules meeting only one criterion were designated senescent-only or innate-only, and lobules meeting neither criterion were designated neutral.

Clearance hubs were defined with a two-component criterion intended to capture direct innate engagement of p16^+^ epithelium together with sufficient innate abundance. First, direct engagement was summarised by d_CD16_min→p16, defined as the smaller of the median epithelial CD16→epithelial p16 distance and the median stromal CD16→epithelial p16 distance across available compartments. Lobules were designated engagement-hot if d_CD16_min→p16 was at or below the cohort-wide 20th percentile. Second, high CD16 abundance was defined as epithelial CD16 fraction at or above the cohort-wide 80th percentile, stromal CD16 fraction at or above the cohort-wide 80th percentile, or both. Lobules meeting both the engagement-hot and abundance-high criteria were classified as clearance hubs.

Per-woman stall-hub and clearance-hub burdens were calculated as the number of eligible lobules meeting the class definition divided by the number of eligible lobules for that woman. To assess minority-lobule behaviour, mean and maximum epithelial phospho-STAT3 fractions were also summarised per woman and related to involution pace.

#### M2-like macrophage panel and macrophage-targeting endpoints

In the CD68/CD206/p16 baseline panel, cells were segmented and assigned to epithelial or stromal compartments as above. CD68 positivity defined macrophages, and CD206 positivity among CD68-positive cells defined M2-like macrophages. M2-like macrophages were therefore defined as CD68^+^CD206^+^ cells and quantified separately in epithelium and stroma as densities per mm^2^ of the relevant compartment.

For each woman, hotspot endpoints were defined as the maximum stromal M2-like macrophage density across all evaluable lobules. Tail analyses focused on p16-low lobules, defined within each woman as lobules below that woman's 80th percentile of epithelial p16-positive density. Within p16-low lobules, two additional per-woman summaries were computed: the maximum stromal M2-like density and an immune–senescence targeting index defined as the within-woman Pearson correlation between stromal M2-like macrophage density and epithelial p16-positive density across p16-low lobules.

A transcript-only mirror-balance score was also computed within the imaging subset to summarise whether baseline expression favoured a resistant versus clearance-like myeloid programme. Resistant-module genes were *IL6, STAT3, FCGR3A, and FCGR3B. Clearance-module genes were MSR1, MRC1, CD163, CSF1R, ITGAM, and CD68*. Expression values were z-scored across women, averaged within module, and the mirror-balance score was defined as resistant-module score minus clearance-module score.

#### Supportive spatial analyses and validation procedures

Several supportive analyses were used to validate and localise the primary spatial findings. For postmenopausal triad data, p16 heterogeneity was defined as the within-woman between-ROI variance of epithelial p16-positive density, and boundary coupling was defined as the fraction of boundary-adjacent p16-positive epithelial cells whose nearest CD45-positive neighbour was stromal and within 20 μm. Relative predictor importance in multivariable spatial models was evaluated using LMG/Shapley R^2^ decomposition with woman bootstrap. Exploratory mediation analyses tested whether the association between p16 organisation and epithelial proliferation might be partly carried by immune abundance or immune positioning using ROI-level models with woman-clustered inference.

DBSCAN-based validation metrics were also computed to distinguish stall from clearance implementations using an analysis independent of the defining nearest-neighbour thresholds. For phospho-STAT3 clustering, DBSCAN was applied to epithelial phospho-STAT3-positive centroids and the phospho-STAT3 clustering fraction was defined as the fraction of phospho-STAT3-positive cells assigned to a cluster. For p16–CD16 engagement, p16-positive epithelial and CD16-positive cells were pooled, DBSCAN was applied to the combined coordinates, and the mixed-cluster fraction of p16 was defined as the fraction of p16-positive epithelial cells residing in clusters containing at least one p16-positive and at least one CD16-positive cell. The main validation used eps = 12 μm and minPts = 5, with sensitivity sweeps across eps values.

Two mechanistic integration endpoints were also evaluated by stall-hub status. STAT3-coupled proliferation was defined per lobule as the fraction of Ki67-positive epithelial cells that were also phospho-STAT3 positive, calculated as n_Ki67∩pSTAT3/n_Ki67 among lobules with at least one Ki67-positive epithelial cell. Senescence-anchored EREG proximity was defined as the median nearest-neighbour distance from p16-positive epithelial source cells to EREG-positive epithelial target cells within the same lobule, analysed after log1p transformation.

### Statistics

#### General statistical framework

The woman was treated as the primary unit of inference. When multiple lobules from the same woman contributed to an analysis, regression models used robust sandwich standard errors clustered by woman. Group comparisons of lobule/ROI-level endpoints (reported as “cluster-robust” P values) were performed by fitting a linear model with the group indicator as predictor and Huber–White sandwich standard errors clustered by woman, and testing the group coefficient by a two-sided Wald t-test; this retains all lobules while accounting for the correlation among lobules from the same woman. Endpoints summarised as a single value per woman (e.g., sample-level expression indices) were instead compared by Welch's two-sample t-test, which does not assume equal group variances and requires no clustering. Complementary uncertainty estimates were obtained by bootstrap resampling of women with percentile confidence intervals. For paired within-woman baseline-to-follow-up comparisons, two-sided Wilcoxon signed-rank tests were used. Unless otherwise noted, continuous predictors were z-scored before model fitting.

#### DASL and IHC analyses

For DASL data, differential expression between noninvoluted and completely involuted groups was assessed with Welch's two-sample t tests on normalised log2 expression values and FDR was controlled with the Benjamini–Hochberg method. PCA was performed on the 500 most variable filtered genes using gene-wise z-scored expression. The proliferation index was compared between groups with Welch's t test. Overlap between the noninvoluted-high gene set and public breast tumour signatures was assessed with Fisher's exact test.

For IHC, ROI-level marker densities were visualised as beeswarm plots coloured by woman with hollow symbols marking woman medians. For inference, ROI-level marker densities were modelled at the ROI level with group as predictor and woman-clustered robust standard errors (the cluster-robust t-test defined above), retaining all ROIs; woman medians are shown as open symbols for visualisation only. As a complementary analysis, 95% confidence intervals were also estimated by woman-level bootstrap. Ki67–TOP2A coordination was assessed by z-scoring each marker density within group and fitting z(TOP2A) = β0 + β1 z(Ki67) + β2 Group + β3 z(Ki67) × Group with woman-clustered robust standard errors.

#### Longitudinal NanoString pace models

For each baseline NanoString feature F, the primary continuous model related involution pace to the feature while adjusting for baseline structure and follow-up interval: pace ∼ F + follow-up interval + baseline median lobular area + baseline median acini + baseline years-from-menopause. Menopause-dependent effect modification was tested by adding a feature × years-from-menopause term: pace ∼ F + yfm + F × yfm + follow-up interval + baseline median lobular area + baseline median acini. The feature × yfm coefficient was interpreted as the change in the feature→pace association across menopausal physiology, and simple slopes were evaluated at representative yfm values.

Trajectory-stratified extreme-group analyses contrasted FAST and STAGNANT women defined as the top and bottom tertiles of involution pace within Pre→Post and Post→Post strata. Genes were ranked by the FAST − STAGNANT Welch t statistic and analysed by preranked GSEA using GenePattern GSEAPreranked v4.4.0 with MSigDB Hallmark gene sets v2026.1, weighted scoring, 1000 permutations, minimum gene-set size of 10, random seed 12,345, and no collapse or remap. Gene sets were restricted to genes present in the ranked panel-constrained list. Robustness was assessed by restricting the follow-up interval, varying the menopause buffer around zero, changing panel-coverage thresholds, examining Cook's distance, and, for Hub12, recomputing the score after dropping each member gene in turn.

#### Lobule-level imaging models

Primary imaging endpoints were modelled at the lobule level using generalised linear models with woman-clustered robust standard errors. Binary endpoints, including Complete20 and hot-lobule indicators, used a logit link. Continuous endpoints, including densities, fractions, and log-transformed distances, used ordinary least squares. To test menopause-dependent effect modification, models included involution pace, trajectory (Post→Post versus Pre→Post), their interaction, and inter-biopsy interval where indicated. For analyses using baseline years-from-menopause as a continuous axis, pace × yfm interaction terms were used analogously.

For follow-up interface analyses, the main model form was g{E(Y_ij)} = β0 + β1 pace_z(i) + β2 PostPost(i) + β3 pace_z(i) × PostPost(i) + β4 Δt_z(i), where i indexes women and j indexes lobules. For binary Complete20, g was the logit link and odds ratios were reported. For ClusterFrac, g was the identity link and interaction coefficients were reported on the fraction scale. Because the evaluable follow-up subset was modest, interaction p values were also checked with conservative t-based Wald tests using degrees of freedom equal to the number of women minus 1 and, where indicated, by exact two-sided permutation tests that permuted trajectory labels across women while keeping group sizes fixed.

#### Bayesian hierarchical model for clearance hubs

As clearance hubs were defined as a lobule-level binary outcome but the number of evaluable women per trajectory was small, clearance-hub inference used a hierarchical mixed-effects logistic model with partial pooling across lobules within woman. For lobule j from woman i, clearance-hub status Y_ij was modelled as Bernoulli(p_ij) with logit(p_ij) = β0 + β1 pace_z(i) + β2 PostPost(i) + β3 pace_z(i) × PostPost(i) + u_i, where u_i is a woman-specific random intercept. The model was fit in R v4.6.1 using brms v2.23.0 with Stan v2.39.0 and mean-field variational Bayes. Fixed effects were assigned weakly informative Normal(0, 2^2^) priors and the standard deviation of the woman random intercept was assigned a half-normal Normal^+^(0, 1) prior. Posterior summaries were reported as posterior means and posterior standard deviations. Menopause-conditioned effect modification was summarised by β3, the corresponding ratio of odds ratios exp(β3), and the approximate posterior probability that β3 > 0. Trajectory-specific simple slopes were summarised as β1 in Pre→Post women and β1 + β3 in Post→Post women. Because mean-field variational Bayes can understate posterior uncertainty, the clearance-hub posterior is reported as an approximation; the menopause-conditioned reversal it indicates is concordant in direction with the independent, assumption-light evidence from the follow-up boundary CD45→p16 interaction and the macrophage-targeting permutation tests.

#### M2-like macrophage and mirror-balance models

Per-woman M2-like macrophage hotspot and targeting endpoints were z-scored across women prior to modelling. Menopause-conditioned effect modification was tested with the per-woman interaction model pace_i = α + β z(F_i) + γ I(Post→Post)_i + δ z(F_i) × I(Post→Post)_i + ε_i, where F_i denotes the woman-level feature and I(Post→Post) identifies women postmenopausal at both biopsies. Two-sided exact permutation p values for the interaction term were obtained by permuting trajectory labels across women while preserving observed Pre→Post and Post→Post group sizes. The same interaction-and-permutation framework was applied to the transcript mirror-balance score.

#### Supportive and sensitivity analyses

To reduce outcome-driven thresholding, spatial “hot” cutoffs, including quintile-based distance thresholds and abundance thresholds, were computed once using cohort-wide distributions within the relevant imaging dataset and then applied unchanged in all downstream models. Geometry-aware CSR corrections, boundary-mask perturbations, and marked-null phenotype permutations were used as supportive robustness checks for spatial enrichment metrics. DBSCAN parameter sweeps were used to assess stability of clustering-based endpoints. For supportive stall-versus-clearance contrasts, lobule-level linear models with woman-clustered robust standard errors were used to compare continuous validation metrics. Throughout, women were treated as the inferential unit and lobule-level analyses were interpreted in the context of cluster-respecting uncertainty estimates.

#### Model assumptions and diagnostics

The woman was the unit of inference throughout, and continuous predictors were z-scored before fitting. Assumptions were addressed by model family. For ordinary least-squares endpoints, including IHC marker densities, continuous spatial metrics, and the NanoString pace models, inference used heteroscedasticity-consistent sandwich standard errors clustered by woman; these are valid under within-woman correlation and non-constant error variance and therefore do not require homoscedasticity or independence of lobules. Influential observations were screened by Cook's distance, and 95% confidence intervals were corroborated by distribution-free bootstrap resampling of women. For binary endpoints modelled by logistic GLMs (Complete20 and “hot"-lobule indicators), the principal small-sample concerns are quasi-separation and influential clusters; because the evaluable subsets were modest, we did not rely on logistic large-sample approximations alone but corroborated each interaction with conservative t-based Wald tests (degrees of freedom equal to the number of women minus one) and with exact two-sided permutation tests that permute trajectory labels across women and make no distributional or asymptotic assumptions, and we confirmed that clustering-based endpoints were stable across DBSCAN parameters. For the two follow-up interface interactions (Complete20 and boundary clustering), we additionally screened individual-woman influence by leave-one-woman-out refitting (each woman omitted in turn), and refit the Complete20 logistic model by Firth penalised likelihood to bound estimates under quasi-separation; for these two interactions we based significance on the exact permutation test (enumerating all C(14,6) = 3003 trajectory assignments) rather than the cluster-robust Wald statistic, which can be anti-conservative with few clusters. The per-woman macrophage and mirror-balance interaction models rested on the same exact-permutation framework, whose only assumption is exchangeability of trajectory labels under the null. Paired within-woman baseline-to-follow-up comparisons used the two-sided Wilcoxon signed-rank test, avoiding distributional assumptions on the paired differences. For the Bayesian hierarchical clearance-hub model, weakly informative priors were used (Normal (0, 2^2^) on fixed effects; half-normal (0, 1) on the woman random-intercept standard deviation), so that posterior conclusions are not driven by the priors.

#### Sample-size determination

This study utilised pre-existing longitudinal and endpoint cohorts assembled through the Mayo Clinic Benign Breast Disease biobank^7^^,^^8^; sample sizes were determined by biobank availability, tissue adequacy, and RNA quality rather than by a formal power calculation. The postmenopausal endpoint cohort (n = 12; 8 noninvoluted, 4 completely involuted) was selected to represent extremes of lobular involution status. The longitudinal pace cohort (n = 81) comprised all women meeting eligibility criteria for paired biopsies with baseline NanoString profiling and follow-up morphometry within the specified age and interval windows. The baseline MxIF imaging subset (n = 14 to 16 women depending on panel) was selected a priori to span the involution pace distribution from fast to stagnant remodelling, and the follow-up MxIF subset (n = 14) represents the subset with known registry menopause age and evaluable paired imaging. No formal power analysis was conducted for the spatial imaging or transcriptomic endpoints. These interaction analyses require sufficient representation across menopausal trajectory strata; the observed effect sizes and p-values are reported with cluster-robust inference and, where noted, exact permutation tests to reflect the modest stratum sizes.

#### Blinding

Pathology review for ARLI classification and lobule annotation was performed by a breast pathologist before quantitative outcome ascertainment. Marker positivity thresholds for IHC and MxIF were established per staining batch using control tissues and visual inspection, and were applied uniformly within batch without reference to involution pace, menopausal trajectory, or group assignment. Spatial metric thresholds (quintile-based distance and abundance cutoffs for hub classification and ‘hot’ lobule designation) were computed once from cohort-wide distributions and applied unchanged across all downstream analyses. Statistical analyses were performed using pre-specified models; where post-hoc endpoints were included they are identified as such. Full allocation concealment was not possible given the retrospective longitudinal cohort design, in which the investigative team had access to clinical and morphometric data. These limitations are acknowledged in the Discussion.

### Role of funders

The funders had no role in the study design, data collection, analysis, interpretation of the data, or in writing of the manuscript.

## Results

### Stalled postmenopausal tissue retains a tumour-associated epithelial state

**Fig. 1:**
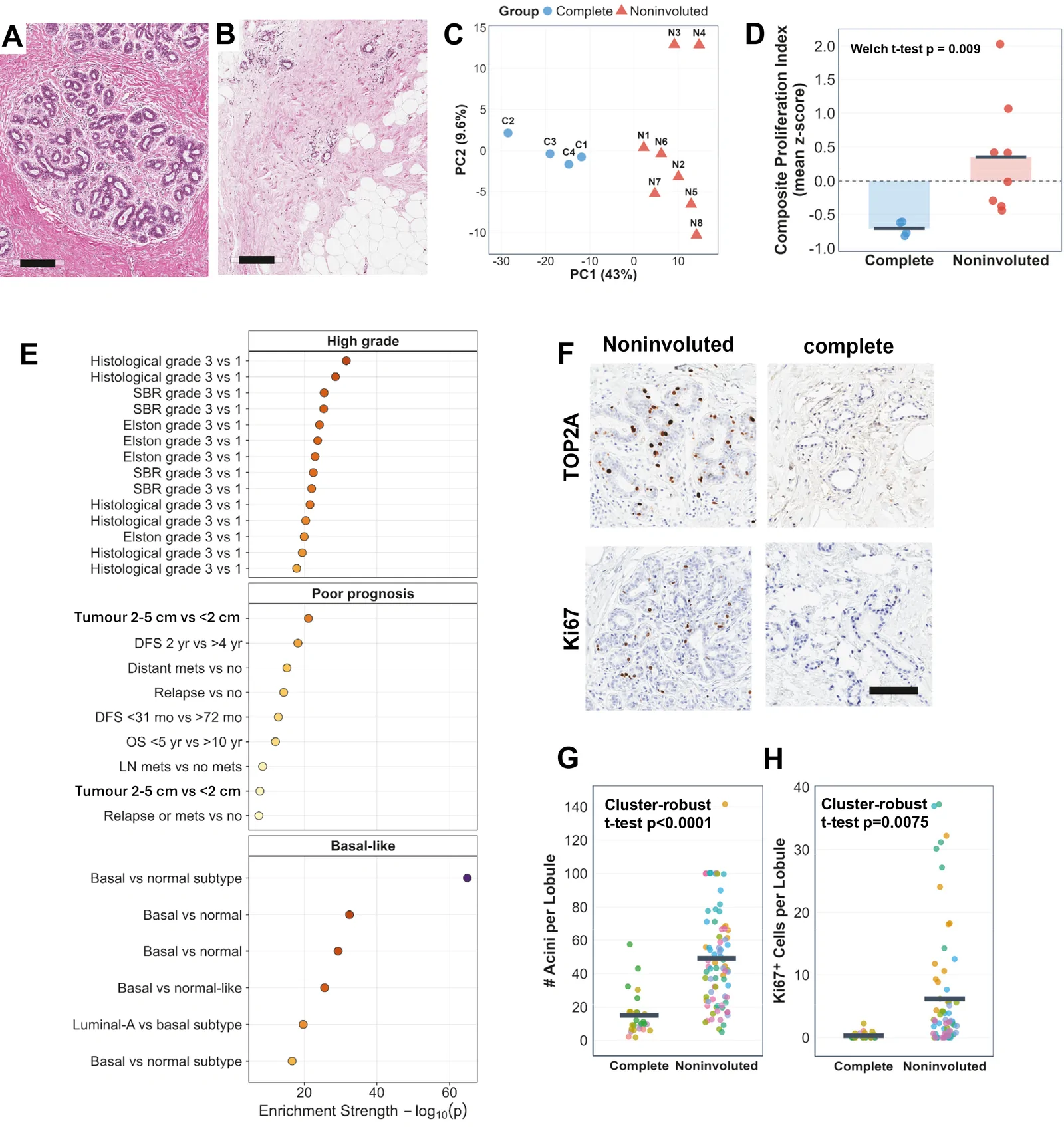
**Breast tissue that stalls at the menopausal transition retains a proliferation-competent epithelial state with tumour-associated gene expression.** (A–B) Representative H&E from postmenopausal benign breast biopsies classified as noninvoluted (A; n = 8) or completely involuted (B; n = 4) by ARLI status. (C) Principal component analysis of genome-wide Illumina DASL expression profiles (top 500 most variable genes from the filtered transcriptome) separates noninvoluted from completely involuted samples along PC1. (D) Composite proliferation index (mean z-score across canonical cell-cycle genes measured on the DASL array) is higher in noninvoluted samples; points, individual samples; bars, group means. (E) Tumour-context enrichment: overlap of the noninvoluted-high gene set (higher in noninvoluted vs completely involuted; nominal two-sided Welch's two-sample t-test P < 0.05) with independent breast-tumour microarray contrasts grouped by histological grade, clinical outcome, and intrinsic subtype. Points, individual contrasts (including repeated phenotype comparisons across independent datasets); x-axis, enrichment strength (−log10 Fisher's exact-test P); tick marks, median; thick bars, interquartile range; n contrasts per category shown on the y-axis. (F) Representative TOP2A (top) and Ki67 (bottom) IHC in noninvoluted (left) and completely involuted (right) lobules (qualitative; quantitative Ki67–TOP2A coupling is shown in Fig. S1F). Scale bar, 100 μm. (G, H) Acini per lobule (G) and Ki67^+^ epithelial nuclei density (H). Points, lobule ROIs coloured by woman; open symbols, woman medians. Statistical tests. Differential expression between groups was assessed per gene by Welch's two-sample t-test on normalised log2 DASL expression with Benjamini–Hochberg FDR control. Cluster-robust P values (G, H) are two-sided Wald tests on the group coefficient from a linear model with woman-clustered robust standard errors (noninvoluted n = 8 women; completely involuted n = 4 women; all lobule ROIs plotted). The composite proliferation index (D) was compared by Welch's two-sample t-test (samples n = 12; 8 noninvoluted, 4 completely involuted). (E) Fisher's exact test across 29 tumour contrasts (n contrasts per category on the y-axis).

To characterise what distinguishes breast tissue that completes the ageing transition from tissue that stalls, we compared postmenopausal benign biopsies spanning extremes of lobular involution (noninvoluted n = 8; completely involuted n = 4). Across multiple TDLU ROIs per biopsy, histology and DASL transcriptomics showed that noninvoluted tissue retained enlarged, epithelial-rich TDLUs, whereas completely involuted tissue showed regressed lobules embedded in adipose and fibrous stroma (Fig. 1A and B). Principal component analysis (PCA) of the top 500 most variable genes separated noninvoluted from completely involuted samples (Fig. 1C), with PC1 loadings dominated by canonical cell-cycle/proliferation genes (Table S2; Fig. S1A–C). A composite proliferation index derived from cell-cycle transcripts was higher in noninvoluted tissue (Fig. 1D). Differential expression between the two groups (Welch's two-sample t-test per gene; Benjamini–Hochberg FDR; 1414 genes) identified 373 genes higher and 204 lower in noninvoluted tissue at Benjamini–Hochberg FDR <0.05 (550 and 439, respectively, at nominal two-sided Welch's two-sample t-test P < 0.05; full ranked results in Table S3). Strikingly, the transcriptional programme retained in noninvoluted tissue shared substantial similarity with high-risk breast tumours: across 29 published tumour contrasts (including repeated phenotype comparisons across independent datasets), the noninvoluted-high gene set (nominal two-sided Welch's two-sample t-test P < 0.05, positive fold-change; 550 genes) consistently overlapped breast-tumour expression contrasts spanning high histological grade, poor outcome, and basal-like subtype (Fig. 1E; Table S4), with overlap dominated by shared cell-cycle regulators. This is a set-overlap enrichment between transcriptional programmes and does not imply that tumour driver alterations or tumour-level expression of individual genes are present in these benign samples. The overlap was dominated by shared cell-cycle and proliferation regulators, indicating that noninvoluted tissue resembles these tumours in proliferative competence rather than in basal/ER-negative lineage identity, consistent with the established link between delayed lobular involution and ER-positive cancer risk, in which the persistent epithelium retains a proliferative programme without adopting a basal lineage.

To confirm this transcriptional phenotype at the protein level, we performed IHC on Ki67 and TOP2A, complementary cell-cycle markers. Representative noninvoluted lobules showed higher nuclear TOP2A and Ki67 staining than completely involuted lobules (Fig. 1F). Quantitatively, noninvoluted lobules contained more acini and higher Ki67^+^ epithelial nuclear density (Fig. 1G and H). TOP2A^+^ epithelial density was directionally higher but more heterogeneous across lobules, and Ki67 and TOP2A densities were more tightly correlated in noninvoluted tissue (Fig. S1E and F), consistent with a coordinated, proliferation-competent epithelial state persisting after menopause. This raised a question that the remainder of the study was designed to address: whether the spatial positioning of p16^+^ (senescence-associated) epithelial foci and immune effectors within individual lobules determines whether such persistence resolves or is sustained.

### Stalled postmenopausal lobules harbour immune cells excluded from p16^+^ epithelial foci

**Fig. 2:**
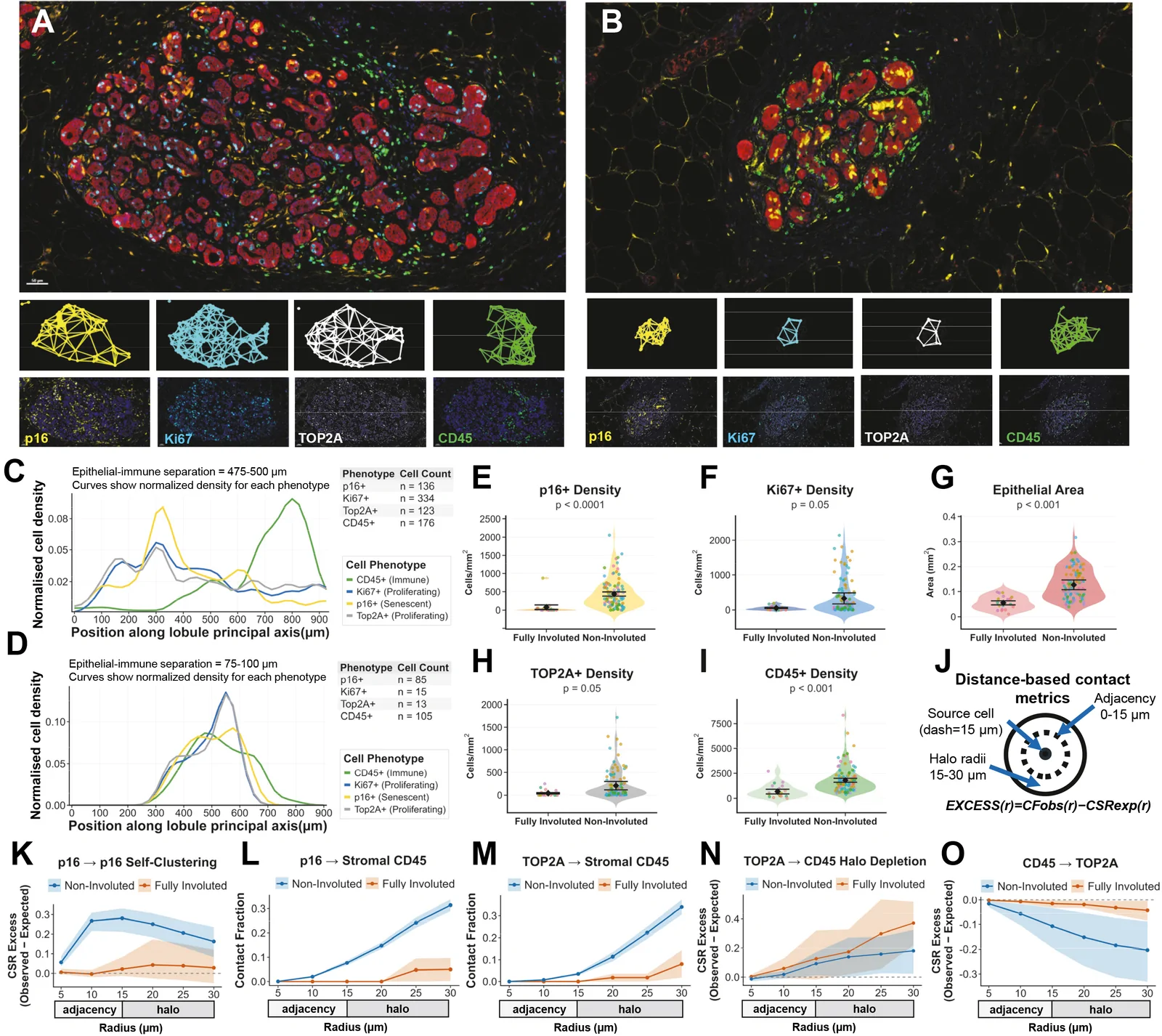
**Stalled postmenopausal breast tissue is immune-rich but immune cells fail to engage p16^+^ epithelial foci.** (A, B) Representative multiplex immunofluorescence (MxIF) composites (panCK, red; p16, yellow; Ki67, cyan; TOP2A, white; CD45, green; DAPI, blue) of a noninvoluted lobule with marked immune–epithelial spatial dislocation (A) or a more interdigitated pattern in an involuted lobule (B). p16 endpoints are restricted to panCK^+^ epithelium. Below each composite, marker-positive cells are rendered as Delaunay graphs (nodes = marker^+^ cells; edges = Delaunay neighbours) and as single-marker renders. (C, D) One-dimensional projections of marker-positive cell density along each lobule's principal axis for the lobules in (A) and (B), respectively (densities normalised within lobule). Epithelial–immune separation is the distance between the CD45^+^ peak and the epithelial-marker peak. (E–I) Lobule-level p16^+^ epithelial density (E), Ki67^+^ epithelial density (F), epithelial area (G), TOP2A^+^ epithelial density (H), and CD45^+^ cell density (I) in completely involuted versus noninvoluted postmenopausal tissue (women: n = 4 and n = 8). Dots, lobules coloured by woman; bars, woman means and group means; two-sided cluster-robust Wald P values shown. (J) Distance-based contact metrics: contact fraction within radius *r* (adjacency 0–15 μm; halo 15–30 μm) and enrichment over complete spatial randomness (CSR; density-matched random mixing), EXCESS(*r*) = observed − CSR expectation. (K) CSR-adjusted EXCESS for p16→p16 self-clustering. (L, M) Contact fraction to stromal CD45^+^ targets for boundary-adjacent epithelial p16^+^ (L) or TOP2A^+^ (M) source cells. (N, O) CSR-adjusted EXCESS for TOP2A→CD45 and CD45→TOP2A interactions across radii; lines, group means; shading, ±s.e.m. In (K–O), shaded bands beneath the x-axis indicate the adjacency (0–15 μm) and halo (15–30 μm) radius ranges (as defined schematically in J). Densities and proximity metrics are computed per annotated lobule (TDLU) and summarised within woman; group labels refer to the donor-level ARLI phenotype. Statistical tests. Lobule-level densities (E–I) were compared by the cluster-robust t-test (completely involuted n = 4 women; noninvoluted n = 8 women; all lobule ROIs plotted). (K–O) CSR-adjusted EXCESS; group means ± s.e.m. across the same women (n = 4 and n = 8); no hypothesis test.

Having established that stalled postmenopausal tissue retains a proliferation-competent, p16^+^-enriched epithelial state, we next asked whether immune cells within these persistent lobules were positioned to engage p16^+^ epithelial foci. Using multiplex immunofluorescence (panCK, p16, Ki67, TOP2A and CD45) in the postmenopausal endpoint cohort, noninvoluted lobules showed marked immune–epithelial spatial separation, whereas involuted lobules were more interdigitated (Fig. 2A and B). We quantified separation as the distance between CD45^+^ and epithelial-marker density peaks along each lobule's principal axis (Fig. 2C and D) and summarised lobule-level marker abundance (women as the unit of inference). Compared with completely involuted lobules, noninvoluted lobules had higher densities of p16^+^ epithelium and CD45^+^ leukocytes, with higher Ki67^+^/TOP2A^+^ densities and larger epithelial area (Fig. 2E–I). To test whether immune cells were positioned for contact with p16^+^ or proliferative epithelial foci, we quantified leucocyte–epithelial proximity across radii using directional contact fractions and a density-adjusted enrichment metric relative to a random-mixing baseline (EXCESS; Methods) (Fig. 2J).

Despite higher overall leucocyte burden, immune cells in noninvoluted lobules fail to access p16^+^ epithelial foci, accumulating at the epithelial–stromal boundary but excluded from periacinar neighbourhoods around senescent targets. Noninvoluted lobules showed tighter intraepithelial p16^+^ clustering (p16→p16 EXCESS; density-adjusted enrichment), most pronounced at periacinar “halo” radii, consistent with focal p16^+^ neighbourhoods (Fig. 2K). For boundary-adjacent epithelial p16^+^ and TOP2A^+^ sources (≤20 μm from the epithelium–stroma interface), the fraction with a nearby stromal CD45^+^ neighbour was higher in noninvoluted than completely involuted lobules across radii, indicating immune accumulation at the boundary (Fig. 2L and M); these metrics are evaluated separately for each epithelial phenotype and reflect interface accumulation rather than p16–TOP2A co-localisation. Distance-to-boundary gradients independently localised cross-interface coupling to the interface-proximal zone, with the probability that the nearest CD45^+^ neighbour is stromal diminishing with increasing distance into the lobule interior (Fig. S2A and B).

In contrast to boundary accumulation, leucocyte enrichment around proliferative epithelium was reduced in periacinar halo neighbourhoods: CSR-adjusted TOP2A→CD45 (and reciprocal CD45→TOP2A) EXCESS was attenuated at halo radii in noninvoluted lobules (Fig. 2N and O). Thus, noninvoluted lobules are immune-rich yet exhibit an immune-present but halo-depleted architecture at the spatial scale most relevant to periacinar proliferative foci. Consistent with the idea that immune abundance alone does not capture spatial engagement, a joint model integrating stromal CD45^+^ density and boundary-restricted p16→CD45 coupling suggested that the inverse association between immune burden and proliferative marker densities is strongest when boundary coupling is high (Fig. S2C). Finally, compartment- and band-resolved CSR-adjusted contact decomposition localised the mismatch to a cross-interface halo gap: relative to completely involuted lobules, noninvoluted lobules show reduced stromal CD45^+^→epithelial p16^+^ EXCESS beyond immediate adjacency (halo/mid and far bands) (Fig. S2D). Together, these findings define a persistent-lobule microenvironment in which immune cells accumulate at the epithelial-stromal boundary but are spatially excluded from p16^+^ and proliferative epithelial foci, a configuration consistent with surveillance present but clearance execution incomplete.

### Menopausal timing is associated with how immune cells access p16^+^ epithelium during breast ageing

**Fig. 3:**
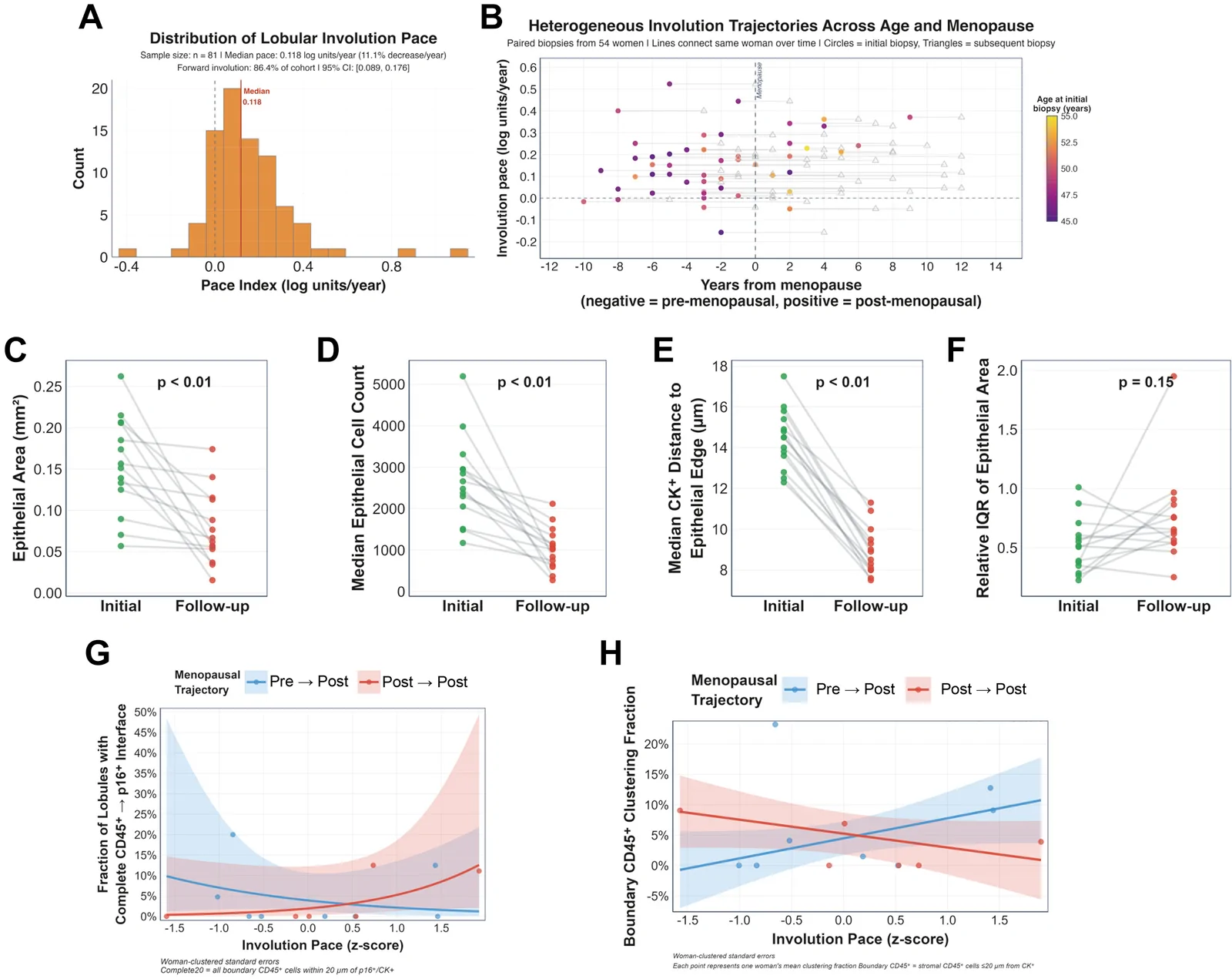
**Immune access to p16^+^ breast epithelium associates with ageing pace in opposite directions depending on menopausal context.** (A) Involution pace index in 81 women with paired benign biopsies (baseline 45–55 years). Pace is the mean per-year log decrease in median lobular area and median acini per lobule (higher = faster regression). (B) Each woman's interval anchored to years-from-menopause (biopsy age − registry menopause age; negative premenopausal, positive postmenopausal). Baseline (filled circles) and follow-up (open triangles) biopsies are shown for women with known menopause age and baseline within ±10 years of menopause. This panel includes n = 51 women. (C–F) In the follow-up MxIF subset (n = 14 women; 8 Pre→Post, 6 Post→Post), paired baseline→follow-up changes in lobule-scale epithelial content and architecture (woman-level summaries across annotated lobules): epithelial area per lobule (C), panCK^+^ epithelial cell count (D), median epithelial panCK^+^ distance to the epithelial edge (thickness proxy, E), and within-woman lobule-size heterogeneity (relative IQR, F); paired Wilcoxon P values shown. (G) Follow-up boundary leucocyte– p16^+^ proximity. CF20 (CD45→p16), fraction of boundary stromal CD45^+^ cells (≤20 μm from interface) whose nearest epithelial p16^+^ cell lies within 20 μm; Complete20 indicates CF20 = 1. Points show per-woman fractions of follow-up lobules meeting Complete20; curves show logistic regression fits adjusted for inter-biopsy interval (171 lobules). (H) Boundary CD45 clustering fraction (DBSCAN) versus pace, stratified by trajectory; regression fits as in (G) (195 lobules). Statistical tests. (C–F) Two-sided Wilcoxon signed-rank test (n = 14 women). (G) Two-sided Wald test on the pace × trajectory interaction from a logistic GLM with woman-clustered robust standard errors (n = 14 women [8 Pre→Post, 6 Post→Post], 171 lobules). (H) As in (G) using an identity-link GLM for the boundary CD45 clustering fraction (195 lobules). Throughout, longitudinal menopausal trajectory is denoted Pre→Post (premenopausal at baseline, postmenopausal at follow-up) and Post→Post (postmenopausal at both biopsies). Within-trajectory pace slopes (for reference): (G) Pre→Post −0.62 (95% CI −1.85 to 0.61), Post→Post 1.03 (0.33–1.73), log-odds scale; (H) Pre→Post 0.033 (0.007–0.059), Post→Post −0.023 (−0.048 to 0.002), fraction scale. Panel annotations give two-sided cluster-robust Wald p-values for the descriptive within-trajectory slopes; the pace × trajectory interaction was assessed by a two-sided exact permutation test.

Having established that persistent postmenopausal lobules harbour immune cells that fail to engage p16^+^ epithelial foci, we asked whether this miscoordination is fixed or whether it varies with the pace of involution and the menopausal context in which remodelling occurs. We analysed 81 women with paired benign biopsies (baseline age 45–55; follow-up 2–10 years) who had noninvoluted lobules at baseline (Table 1). We summarised involution pace as the average per-year proportional (log) decrease in lobular epithelial content (lobular area and acini per lobule) between a woman's two biopsies, so that higher values denote faster regression and negative values denote net epithelial expansion (Methods; Fig. 3A). Because pace is expressed per year on a log scale, it is comparable across intervals of differing length; but as the instantaneous rate may vary across the menopausal transition, we did not treat it as a single menopause-independent quantity, instead testing every association with baseline programmes for modification by menopausal timing.

**Table 1:** Cohort characteristics.

Characteristic	Overall cohort (n = 81)	Imaging subset (n = 14)	Non-imaging (n = 67)	P value^a^
Timing and anthropometrics				
Age at baseline biopsy, years	49.1 (2.9)	48.8 (2.9)	49.1 (3.0)	0.720
Age at follow-up biopsy, years	55.6 (4.5)	56.3 (3.3)	55.4 (4.7)	0.344
Biopsy interval, years	6.6 (3.5)	7.6 (3.6)	6.4 (3.4)	0.248
Body mass index at baseline, kg/mˆ2	25.0 (4.6)	24.2 (3.3)	25.1 (4.8)	0.699
BMI category at baseline				0.807
≤21	15 (23.8%)	3 (30.0%)	12 (22.6%)	
22–25	24 (38.1%)	3 (30.0%)	21 (39.6%)	
26–29	14 (22.2%)	3 (30.0%)	11 (20.8%)	
30+	10 (15.9%)	1 (10.0%)	9 (17.0%)	
Missing	18 (22.2%)	4 (28.6%)	14 (20.9%)	
Menopause				
Baseline menopausal status				0.570
Premenopausal	46 (56.8%)	8 (57.1%)	38 (56.7%)	
Postmenopausal	35 (43.2%)	6 (42.9%)	29 (43.2%)	
Follow-up menopausal status				0.197
Premenopausal	10 (12.3%)	0 (0.0%)	10 (14.9%)	
Postmenopausal	71 (87.7%)	14 (100.0%)	57 (85.1%)	
Menopausal trajectory (baseline→follow-up)				0.149
Pre→Pre	10 (12.3%)	0 (0.0%)	10 (14.9%)	
Pre→Post	36 (44.4%)	8 (57.1%)	28 (41.8%)	
Post→Post	35 (43.2%)	6 (42.9%)	29 (43.2%)	
Age at menopause (registry), years	47.7 (7.2)	49.4 (3.2)	47.3 (7.9)	0.783
Years from menopause at baseline (baseline age − menopause age), years	1.5 (7.8)	−0.6 (3.6)	2.0 (8.5)	0.789
Digital morphometry				
Baseline median lobule area, μm^2^	124,960 [90,721, 183,138]	134,085 [96,538, 163,635]	124,884 [84,666, 195,374]	0.713
Baseline median acini per lobule, n	39.6 (17.9)	44.0 (20.7)	38.7 (17.3)	0.431
Follow-up median lobule area, μm^2^	53,194 [32,241, 82,520]	45,978 [20,623, 52,987]	59,062 [33,425, 82,574]	0.156
Follow-up median acini per lobule, n	23.0 (17.0)	17.5 (11.2)	24.1 (17.9)	0.110
Involution pace index (composite)	0.15 (0.20)	0.18 (0.11)	0.15 (0.21)	0.168

The overall longitudinal cohort includes 81 women with paired benign breast biopsies (baseline age 45–55 years) with digital morphometry at both time points. The imaging subset comprises women with baseline multiplex immunofluorescence and registry age at menopause available, used for menopause-anchored spatial analyses. Continuous variables are shown as mean (SD), except lobule area shown as median [IQR]. Categorical variables are n (% among non-missing).

a P values compare the imaging subset versus the non-imaging women (Wilcoxon rank-sum for continuous variables; Fisher's exact test for 2 × 2 categorical variables; χ^2^ test otherwise); P value for BMI category excludes missing.

Chronological age alone misses the physiologic inflection point of menopause, so we anchored each biopsy to years-from-menopause (biopsy age minus registry menopause age) and classified intervals as Pre→Post (traversing menopause) or Post→Post (postmenopausal at both biopsies) (Fig. 3B). We treated menopausal trajectory as an interval-level physiologic context and tested it as an effect modifier of pace–architecture associations.

To connect pace to microanatomic phenotypes measured at the outcome timepoint, we performed follow-up MxIF in a subset of selected a priori to span the pace distribution. In this menopause-anchored subset (n = 14 women), eight women were Pre→Post and six were Post→Post (Fig. S3A; Table 1). Follow-up lobules showed epithelial contraction and remodelling (reduced epithelial area and panCK^+^ epithelial cell content) whereas within-woman heterogeneity showed only a non-significant directional increase (two-sided Wilcoxon signed-rank p = 0.15; Fig. 3C–F), consistent with asynchronous, lobule-level remodelling during ARLI.

Boundary leucocyte access to p16^+^ epithelial targets associated with involution pace in opposite directions depending on menopausal trajectory, quantified using a 20-μm interface coupling metric (Complete20; Methods). Complete20 is an all-or-nothing per-lobule criterion met when every boundary stromal CD45^+^ leucocyte has an epithelial p16^+^ cell within 20 μm, i.e., uniform immune access to p16^+^-associated epithelium across the lobule boundary. In Post→Post women, faster involution was associated with higher odds of uniform p16 adjacency, whereas in Pre→Post women the association was in the opposite direction (Fig. 3G). The direction of this effect modification persisted after adjusting for boundary CD45^+^ counts (two-sided cluster-robust Wald interaction p = 0.0145; Fig. S3B) and across radii in density-adjusted coupling/AUC summaries (Fig. S3C). Each point in Fig. 3G shows one woman's fraction of follow-up lobules meeting Complete20, an all-or-nothing per-lobule criterion (every boundary CD45^+^ cell with an epithelial p16^+^ cell within 20 μm); women with no qualifying lobule therefore appear at 0%, while the interaction itself was estimated at the lobule level (171 lobules) using woman-clustered logistic regression rather than from the per-woman proportions. The density-adjusted and abundance-adjusted concordance above (Fig. S3B and C) indicates that this interface coupling is not an artefact of the smaller, denser lobules that accompany epithelial contraction (Fig. 3C and D).

Because target-proximal positioning can arise from either diffuse or aggregated boundary immune configurations, we also quantified boundary CD45 aggregation as the fraction of boundary CD45^+^ cells that fall within a dense spatial aggregate (DBSCAN clustering fraction; Methods), indexing immune aggregation rather than uniform distribution along the boundary. Boundary clustering showed the mirror-image trajectory pattern, with the opposite orientation to Complete20: in Post→Post women, faster involution aligned with less boundary clustering, whereas in Pre→Post women, faster involution aligned with more boundary clustering (Fig. 3H). This divergence was robust across DBSCAN parameter choices (Fig. S3D). Because Complete20 (uniform p16 adjacency) and boundary clustering (immune aggregation) are distinct and oppositely oriented metrics, a woman at 0% for one need not be at 0% for the other, and their per-woman floors are independent. This Complete20 result concerns the same pace × trajectory effect modification as the nominal abundance-adjusted Wald result above, but it was evaluated in the primary model with the prespecified exact woman-level permutation test rather than the cluster-robust Wald test. At n = 14, neither interface interaction was significant under this exact test (Complete20, two-sided exact permutation p = 0.37; boundary clustering, two-sided exact permutation p = 0.26), and both were sensitive to individual women in leave-one-woman-out refits, although the interaction direction was preserved under every omission and under the density- and abundance-adjusted models above (Fig. S3B and C). Interface proximal leukocytes thus appeared to adopt distinct organisations across menopausal interval contexts: diffuse, uniformly p16-adjacent positioning in Post→Post women, consistent with productive immune engagement, versus clustered, non-uniform adjacency in Pre→Post women, a directional pattern suggesting that the same innate immune cells may adopt different spatial strategies depending on which side of the menopausal transition they are operating on.

### Senescent–immune gene programmes reverse their link to breast ageing trajectory across menopause

Given that boundary leukocytes align with uniform p16 adjacency in one physiologic context but with boundary aggregation and less uniform adjacency in another (Fig. 3), we next asked whether baseline molecular programmes associate with subsequent remodelling speed, and whether these associations differ across the menopausal transition. We profiled baseline tissue from the same 81-woman cohort using NanoString IO360/BC360 panels, relating gene expression features to the involution pace index while explicitly testing whether menopausal timing modified those associations (Table S5).

**Fig. 4:**
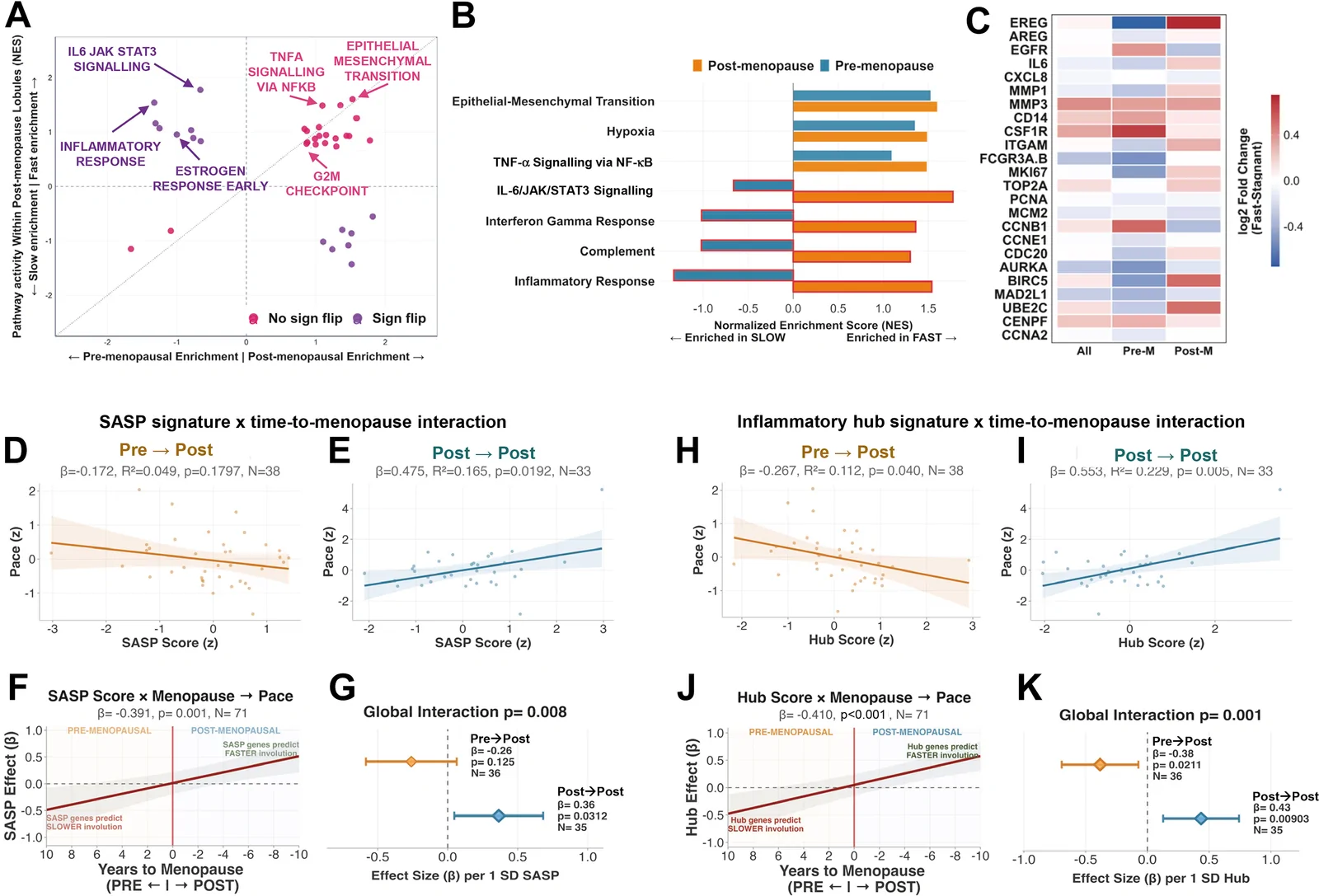
**Transcriptomic senescent-immune programmes reverse their association with breast ageing trajectory across the menopausal transition.** (A) Preranked GSEA of MSigDB Hallmark gene sets performed on trajectory-stratified baseline FAST versus STAGNANT contrasts (pace tertiles); normalised enrichment scores (NES) shown for Pre→Post and Post→Post strata (NanoString panel genes), displaying all sets tested and highlighting those that reverse direction across menopause. (B) Representative pathways from (A); positive NES indicates enrichment in FAST involution and negative NES indicates enrichment in STAGNANT involution. The se`nescence-associated programmes here are the SASP/innate-inflammatory sets (*IL6/JAK/STAT3* signalling, TNFα signalling via NF-κB, inflammatory response, interferon-γ and complement responses); the proliferative and hormonal sets are shown to mark the accompanying axes rather than senescence per se. (C) Targeted gene-tile summary (NanoString transcripts; longitudinal cohort). Tile colour encodes the log2 fold-change of each transcript between FAST and STAGNANT involution (FAST − STAGNANT) computed within each trajectory stratum (red, higher in FAST involution; blue, higher in STAGNANT involution). Columns show the contrast across all women (All) and within the Pre→Post and Post→Post strata. Asterisks denote nominal significance. (D, E) Baseline SASPcore score versus involution pace, shown separately for women traversing the menopausal transition during the follow-up interval (Pre→Post, D) and women postmenopausal at both biopsies (Post→Post, E). (F) Adjusted SASPcore × years-from-menopause interaction model relating baseline SASPcore to involution pace (95% CI); vertical line marks menopause (0). (G) Trajectory summary for SASPcore: standardised associations (β ± 95% CI; per +1 SD score) from an adjusted score × trajectory interaction model comparing women who traverse menopause (Pre→Post) versus women postmenopausal at both biopsies (Post→Post). (H–K) As in D–G for the inflammatory hub transcript programme (Hub12 score): baseline-stratified scatterplots (H, I), continuous hub × years-from-menopause interaction (J), and trajectory-interaction summary (K). Panels F,J include women with known registry menopause age (n = 71). Panels G, K include trajectory-defined women (Pre→Post n = 36; Post→Post n = 35). Statistical tests. (A,B) Preranked GSEA (MSigDB Hallmark, weighted scoring, 1000 permutations); normalised enrichment scores shown. (C) log2 fold-change (FAST − STAGNANT) computed within each trajectory stratum; asterisks denote nominal per-gene significance. (D, E, H, I) within-stratum associations of the baseline score with involution pace by ordinary least-squares regression (β, R^2^, two-sided p; per-stratum n shown in each panel). (F, J) adjusted feature × years-from-menopause interaction models fit with woman-clustered (Huber–White) robust standard errors (β, 95% CI; n = 71 women with known registry menopause age). (G, K) score × trajectory interaction summaries with woman-clustered robust standard errors and two-sided Wald p for the interaction term (Pre→Post n = 36 women [FAST n = 12, STAGNANT n = 12]; Post→Post n = 35 women [FAST n = 12, STAGNANT n = 12]).

The imaging data suggested that menopausal timing reorganises how immune cells engage p16^+^ epithelium; we next asked whether this reorganisation was detectable at the level of gene expression programmes measured before the transition. To obtain a programme-level view, we first performed trajectory-stratified fast-versus-stagnant contrasts (top vs bottom pace tertile within each trajectory stratum) and applied preranked Hallmark GSEA to the resulting ranked gene statistics. Inflammatory and stress-response enrichment was strongly context-dependent: cytokine/STAT3-linked pathways show opposing enrichment patterns across the menopausal transition window versus established postmenopausal states (Fig. 4A and B). These results motivated a focused interrogation of four biologic axes repeatedly implicated by enrichment patterns and prior ARLI/senescence biology: EGFR signalling, SASP/inflammatory core, innate/myeloid activation, and proliferation.

Guided by these themes, we summarised gene-level behaviour using an effect heatmap of log2FC (FAST − STAGNANT) within each trajectory stratum across representative genes spanning these four axes. This view makes menopause-conditioned directionality shifts explicit: transcripts such as the EGFR ligand *EREG* and the Fcγ receptor *FCGR3A/B* (CD16), which distinguish stagnation during the Pre→Post transition window, exhibit the opposite directionality in Post→Post women (Fig. 4C; expanded gene-by-individual displays, Fig. S4A and B). These tiles encode the direction of each transcript's association with involution pace within a stratum (FAST − STAGNANT), not its absolute abundance; accordingly, a transcript may associate with faster involution in one menopausal context and with stagnation in the other, reflecting the menopause-conditioned reversal rather than inconsistent expression.

To test whether the menopause-conditioned reversal held across the full distribution of involution pace rather than only at the extremes, we evaluated a priori, panel-constrained signature scores and anchor genes (prespecified membership, category, biological basis, and aligned protein readouts in Table S6) in models relating baseline features to the continuous pace index (Table S5). These feature × years-from-menopause models relate each programme directly to a woman's time relative to menopause, spanning the pre- and post-menopausal range. A composite SASPcore score showed clear effect modification (Fig. 4D–G): higher SASPcore aligned with slower involution in more premenopausal states but with faster involution in more postmenopausal states. The pre-menopausal SASPcore–pace slope was itself weak and not individually significant (R^2^ = 0.049, two-sided ordinary least-squares t-test p = 0.18); the reversal is supported by the significant SASPcore × years-from-menopause interaction (Fig. 4F) and the individually significant post-menopausal slope (Fig. 4E), not by two independently significant simple slopes. Submodule analyses localised this switch to STAT3/cytokine-associated components, whereas an EGFR-field-only module provided a weaker comparator signal (Fig. S4C–E).

Guided by this localisation, we evaluated an EGFR–STAT3–innate hub transcript programme (Hub12; defined a priori as the union of the EGFR ligand field, the STAT3 axis, an innate CD16 anchor, and a minimal inflammatory drive set; Table S6). Hub12 showed the strongest menopause-linked reversal (Fig. 4H–K), remained stable under leave-one-gene-out recomputation (Fig. S4F), and matched the pathway- and gene-level trajectory patterns (Fig. 4A–C). Trajectory-stratified standardised effect summaries make the two-regime pattern explicit for both Hub12 and SASPcore (Fig. 4G–K): in Pre→Post women, higher baseline scores align with subsequent stagnation, whereas in Post→Post women, the same programmes align with faster ongoing involution. Together, these analyses indicate that senescence-linked inflammatory/innate programmes are not intrinsically pro- or anti-remodelling; rather, their relation to the menopausal transition defines whether these programmes couple to persistence-associated organisation during the transition window versus clearance-associated execution after menopause.

### Lobule-scale hub marker geometry reverses its association with ageing trajectory across menopause

**Fig. 5:**
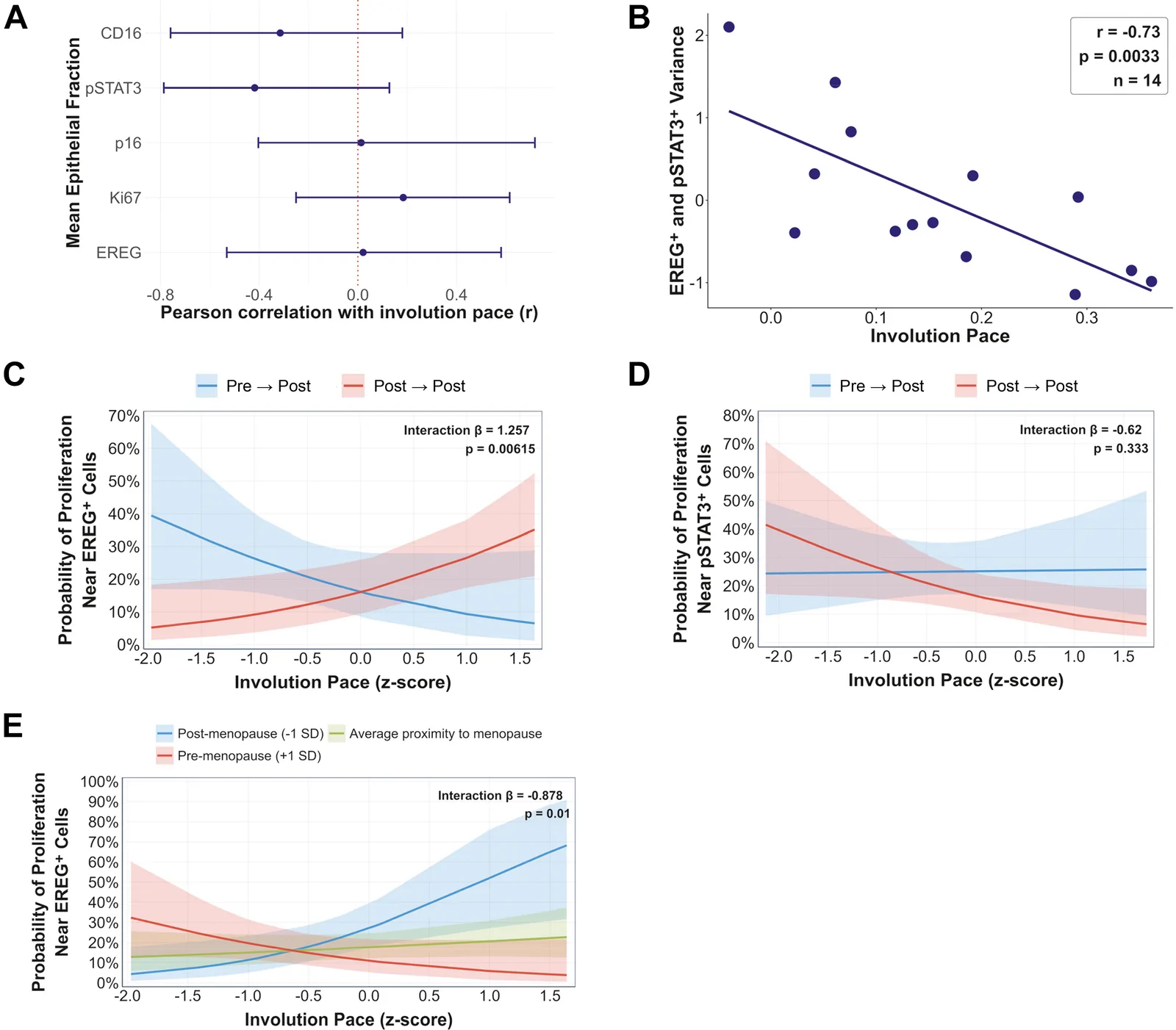
**Within-breast mosaicism in senescent-immune hub markers reflects ageing trajectory, with hub geometry reversing meaning across the menopausal transition.** (A) Pearson correlations between involution pace and each woman's mean epithelial fractions of pSTAT3^+^, CD16^+^, p16^+^, Ki67^+^ and EREG^+^ cells; bars show bootstrap 95% CIs. For each marker, the Pearson correlation of its mean epithelial single-marker fraction with involution pace (one correlation per marker; bootstrap 95% CIs; n = 14 women); these are marker-versus-pace associations, not correlations between markers. (B) HubPatchiness versus pace: HubPatchiness is the prespecified mean of the z-scored between-lobule variances of the epithelial EREG^+^ and pSTAT3^+^ single-marker fractions within each woman, chosen because EREG and pSTAT3 index the ligand and signalling fields central to the hub model. (C) Menopause-dependent reversal for EREG-field embedding of proliferation. Curves show the probability that a lobule is EREG-hot (lowest quintile of the lobule-median Ki67^+^EREG^−^→EREG^+^ distance; cohort-wide q20 = 5.76 μm) versus pace, stratified by trajectory. Models include women with known registry menopause age (n = 14; Pre→Post n = 8; Post→Post n = 6) and their eligible lobules (176 lobules); shaded bands, 95% CIs (woman-clustered). (D) As in (C) for pSTAT3-field embedding (Ki67^+^pSTAT3^−^→pSTAT3^+^; cohort-wide q20 = 7.93 μm, computed across all eligible lobules; 210 eligible lobules from the same n = 14 women). (E) Continuous years-from-menopause model for EREG embedding (biopsy age − registry menopause age; negative premenopausal, positive postmenopausal) using the same n = 14 women; predictions at −1 SD, 0 and +1 SD are shown (shaded, 95% CIs). Statistical tests. (A) Pearson correlations between involution pace and each woman's mean epithelial marker fractions, with bootstrap 95% CIs (n = 14 women); Spearman rank coefficients were concordant and non-significant (pSTAT3 ρ = −0.43, CD16 ρ = −0.31, p16 ρ = 0.28, Ki67 ρ = 0.40, EREG ρ = 0.02; all bootstrap CIs spanning zero). (B) Pearson correlation (n = 14 women). (C, D, E) Logistic GLM with woman-clustered robust standard errors; two-sided Wald test on the pace × trajectory (C, D) or pace × years-from-menopause (E) interaction.

Having shown that the transcriptional direction reversal operates at the level of gene programmes, we next asked whether it is visible at the spatial level within individual lobules, and whether the geometry of senescent-immune hub markers within the breast predicts ageing trajectory differently across the menopausal transition. In the 14-woman pace-linked imaging subset, baseline multiplex imaging quantified lobule-resolved Ki67, p16, EREG, pSTAT3 and CD16 (Fig. 5A; Fig. S5A–C). EREG expression is shown directly at the transcript level in the targeted gene summaries (Fig. 4C; Fig. S4A and B); at the protein level, the per-woman epithelial EREG^+^ fraction did not differ by menopausal trajectory (two-sided Wilcoxon rank-sum P = 0.85). Mean epithelial fractions showed weak associations with pace, so we focused on within-breast heterogeneity. EREG^+^ and pSTAT3^+^ fractions varied substantially across lobules, so we summarised their within-woman, between-lobule variability as a single score (HubPatchiness; Methods). HubPatchiness summarises within-breast mosaicism: it is the average between-lobule variability of a woman's epithelial EREG^+^ and pSTAT3^+^ fractions, with higher values indicating that the EGFR-ligand and STAT3-signalling fields are concentrated in a minority of lobules rather than spread evenly. HubPatchiness increased as involution slowed (Pearson r = −0.73, two-sided correlation p = 0.0033; n = 14; Fig. 5B), consistent with asynchronous lobule-level remodelling.

Both field components carried this association individually and in the same direction (EREG-field between-lobule variance Pearson r = −0.66, two-sided correlation p = 0.01; pSTAT3-field variance Pearson r = −0.69, two-sided correlation p = 0.006) and were themselves correlated (Pearson r = 0.73, two-sided correlation p = 0.0028; Fig. S5A–C), indicating that HubPatchiness reflects a shared mosaicism rather than either marker alone. We prespecified the metric on the EREG and pSTAT3 epithelial fields, the EGFR-ligand and STAT3-signalling fields central to the hub model, rather than selecting among markers to maximise its association with pace; p16, Ki67 and CD16 enter the model in their distinct roles (senescence-associated target, proliferating source, innate effector) through the engagement and embedding metrics appropriate to each.

To add spatial context, we classified lobules by whether proliferative cells lacking the field marker were embedded within the nearest field-positive epithelial neighbourhood (lowest quintile of median nearest-neighbour distance; Methods). Field embedding measures how closely proliferating (Ki67^+^) epithelial cells sit within the EREG^+^ (or pSTAT3^+^) field; a lobule is “hot” when this proximity is in the closest cohort-wide quintile, indicating proliferation embedded within the ligand (or signalling) field. Ki67-EREG proximity recapitulated the menopause-conditioned reversal suggested by the transcriptomic EGFR–EREG axis. Using the EREG-hot indicator (q20 = 5.76 μm; 176 eligible lobules), the pace × trajectory interaction remained significant (two-sided woman-clustered Wald p = 0.00615; Fig. 5C): faster involution corresponded to fewer EREG-hot lobules in women traversing menopause (Pre→Post), but to more EREG-hot lobules in women postmenopausal at both biopsies (Post→Post). Thus, the association between EREG^+^ epithelial fields around proliferative events and remodelling speed depends on menopausal physiology, noting that EREG positivity marks ligand-producing cells, not secreted ligand. The direction reversal was specific to the EREG field: Ki67 proximity to the pSTAT3 field showed no comparable reversal (two-sided woman-clustered Wald p for the pace × trajectory interaction = 0.333; Fig. 5D), with Post→Post women showing only a weak tendency toward lower Ki67-pSTAT3 proximity at faster pace and the Pre→Post relationship near flat. EREG and pSTAT3 therefore capture distinct aspects of hub geometry rather than serving as interchangeable field readouts.

Modelling menopause continuously yielded the same qualitative switch for EREG embedding: the pace association varied smoothly across years-from-menopause, transitioning from negative in more premenopausal states to positive in more postmenopausal states (two-sided woman-clustered Wald p for the pace × years-from-menopause interaction = 0.01; Fig. 5E). This menopause-conditioned reversal was robust across related Ki67-centred spatial endpoints and tail-restricted definitions (Fig. S5D–J). Together, these spatial data ground the transcript-level direction reversal in a concrete lobule-scale geometry: proliferative cells embedded within an EREG-rich field mark stalled remodelling during the transition but accelerated regression after it, motivating a closer examination of the hub architectures that mediate this switch.

### Menopausal timing is associated with whether senescent–immune hub architectures drive stalling or clearance

**Fig. 6:**
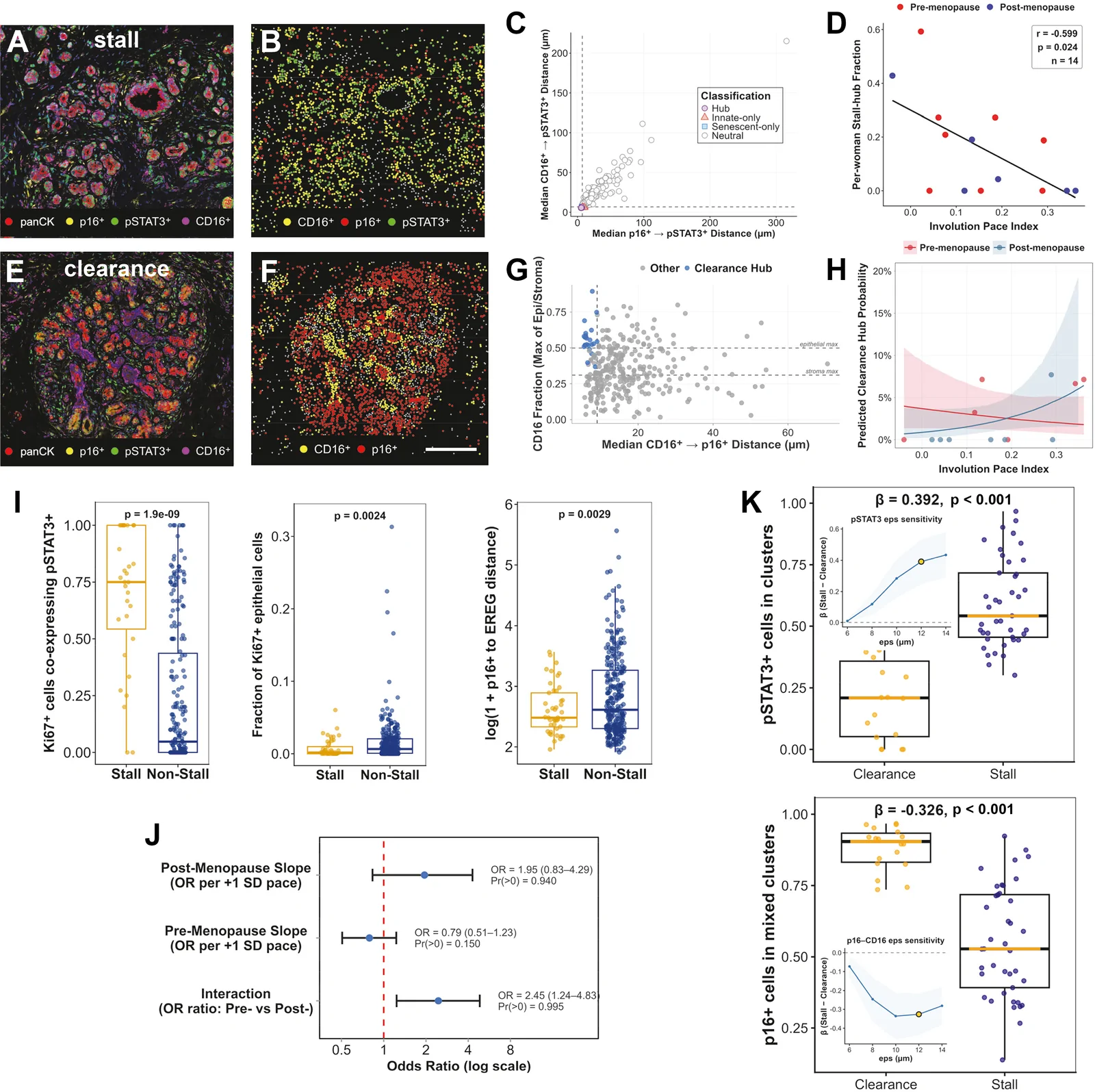
**Lobule-scale senescent-immune architectures adopt stall or clearance-associated configurations with opposite implications for breast ageing depending on menopausal timing.** (A) Representative stall lobule (panCK/CK red, p16 yellow, pSTAT3 green, CD16 magenta; scale bar, 200 μm). (B) Corresponding centroid map for the stall exemplar (cell colours as indicated). (C) Stall-hub classification in p16→pSTAT3 and CD16→pSTAT3 proximity space using 20th-percentile distance cutoffs across lobules (N = 310; hub n = 45; cutoffs shown as dashed lines), yielding stall hub (both distances below cutoff), senescent-only, innate-only, and neutral classes. (D) Per-woman stall-hub burden (mean across each woman's baseline lobules) versus involution pace (Pearson correlation; n = 14 women). Each point is one woman; colour denotes her baseline menopausal status (pre- vs post-menopausal at the imaging biopsy). Pre- and post-menopausal points are different women, not paired pre-/post-menopausal samples from the same woman. (E) Representative clearance lobule (cell colours and scale bar as in A). (F) Corresponding centroid map for the clearance exemplar. (G) Clearance-hub definition. The y-axis is the CD16^+^ fraction taken as the maximum of the epithelial and stromal compartments; a lobule is a clearance hub if it combines a short CD16→p16 engagement distance (bottom quintile; cutoff shown) with high CD16 abundance (top quintile in either epithelium or stroma; cutoffs shown). High CD16 abundance in either compartment satisfies the criterion; clearance hubs are not defined by epithelial CD16 specifically. Highlighted points indicate clearance hubs. (H) Bayesian mixed-effects logistic model of lobule-level clearance-hub probability, testing whether the association between involution pace and clearance-hub odds differs between Post→Post and Pre→Post women, with a per-woman random intercept (model specification in Methods). Curves show population-averaged posterior mean and 95% credible interval; points show observed per-woman clearance-hub fractions (N = 310 lobules, 14 women). (I) Stall-hub epithelial programme endpoints: stall hubs show increased pSTAT3 co-expression among Ki67^+^ epithelial cells (left), reduced overall Ki67^+^ epithelial cells (middle), and reduced p16→EREG distances (right). (J) Bayesian model summary for clearance (OR per +1 SD pace; interaction, OR ratio). (K) Orthogonal validation of the clearance hubs and stall hubs defined in (G) and (C), using an independent DBSCAN mixed-clustering metric computed without reference to the defining cutoffs: stall hubs show increased pSTAT3 clustering (top) and clearance hubs show increased p16–CD16 mixed clustering (bottom) (cluster-robust OLS contrasts; insets show ε-sensitivity summaries). Sample sizes and tests. (D) Pearson correlation (n = 14 women). (H,J) Bayesian mixed-effects logistic model (N = 310 lobules, 14 women); reported as posterior mean, 95% credible interval and posterior probability Pr(β > 0) rather than a frequentist P value. (I) Cluster-robust OLS contrast (stall n = 45 vs non-stall n = 265 lobules). (K) Cluster-robust OLS contrast (stall n = 45 vs clearance n = 24 lobules).

Building on the field-geometry results, multiplex imaging revealed two distinct lobule-scale hub implementations: a signalling-centred stall configuration and an execution-centred clearance-associated configuration. Exemplars show a stall architecture with a p16^+^ focus embedded within a pSTAT3^+^ epithelial field and CD16^+^ cells proximal to that field (Fig. 6A and B), versus a clearance-associated architecture with abundant CD16^+^ cells positioned to directly engage p16^+^ epithelium (Fig. 6E and F).

Stall-hub burden was inversely associated with involution pace (Fig. 6D), consistent with a configuration in which p16^+^ foci and innate effectors are recruited into proximity with an active STAT3 signalling field but without direct engagement of p16^+^ targets. Stall hubs are lobules in which p16^+^ foci and CD16^+^ innate effectors are both positioned close to an active pSTAT3^+^ epithelial field; a configuration of recruitment and signalling engagement without direct clearance of the p16^+^-associated targets (Methods; Fig. 6C), with supportive lobule-level and class-composition analyses (Fig. S6A and B). Notably, within-woman pSTAT3 maxima, but not means, tracked pace more strongly than stall-hub burden alone (Fig. 6D; Fig. S6A–C), consistent with threshold-like trapping of a minority of lobules in persistence-prone configurations rather than a uniform shift across the breast.

Clearance hubs are lobules in which CD16^+^ innate effectors are both abundant (top-quintile CD16 fraction in epithelium or stroma) and positioned for direct contact with p16^+^ epithelium (bottom-quintile CD16→p16 distance), the two conditions productive clearance of senescence-associated epithelium would require, denoting a clearance-associated spatial configuration rather than a directly observed clearance event (Fig. 6G). Clearance-hub probability showed opposite associations with involution pace across menopausal trajectories: in Post→Post women, faster involution corresponded to higher odds of clearance hubs, whereas in Pre→Post women the association was near-null/negative (pace × Post→Post OR ratio ≈2.45, 95% CI ≈1.24–4.83; Fig. 6H and J; Fig. S6D), implying a positive pace effect in Post→Post (median OR ≈1.95 per +1 SD) and lower/near-null in Pre→Post (median OR ≈0.79).

To validate that stall hubs reflect a distinct epithelial state rather than simply a spatial arrangement, we examined their epithelial programme characteristics. Stall hubs showed increased pSTAT3 co-expression among Ki67^+^ epithelial cells, reduced overall Ki67^+^ epithelial fraction, and shorter p16–EREG distances (Fig. 6I), features describing a lobule that is STAT3-active and EREG-proximate but proliferatively suppressed, consistent with a signalling-engaged but execution-incomplete state. Orthogonal clustering metrics further differentiated implementations: clearance hubs showed increased p16–CD16 mixed clustering, whereas stall hubs showed increased pSTAT3 clustering (Fig. 6K), recovering the expected signatures through a method that did not define the hubs. Together, these data show that the senescent-immune hub programmes identified transcriptomically (Fig. 4) manifest spatially as either a stall architecture associated with persistence or a clearance-associated architecture associated with regression, and that the same underlying programmes can carry opposite implications for breast ageing trajectory depending on whether they are present before or after the menopausal transition.

An overview of the study design, cohorts, and the analyses each supports is provided in Fig. S7.

### Menopause-conditioned macrophage targeting of p16^+^ epithelium confirms the hub reversal

**Fig. 7:**
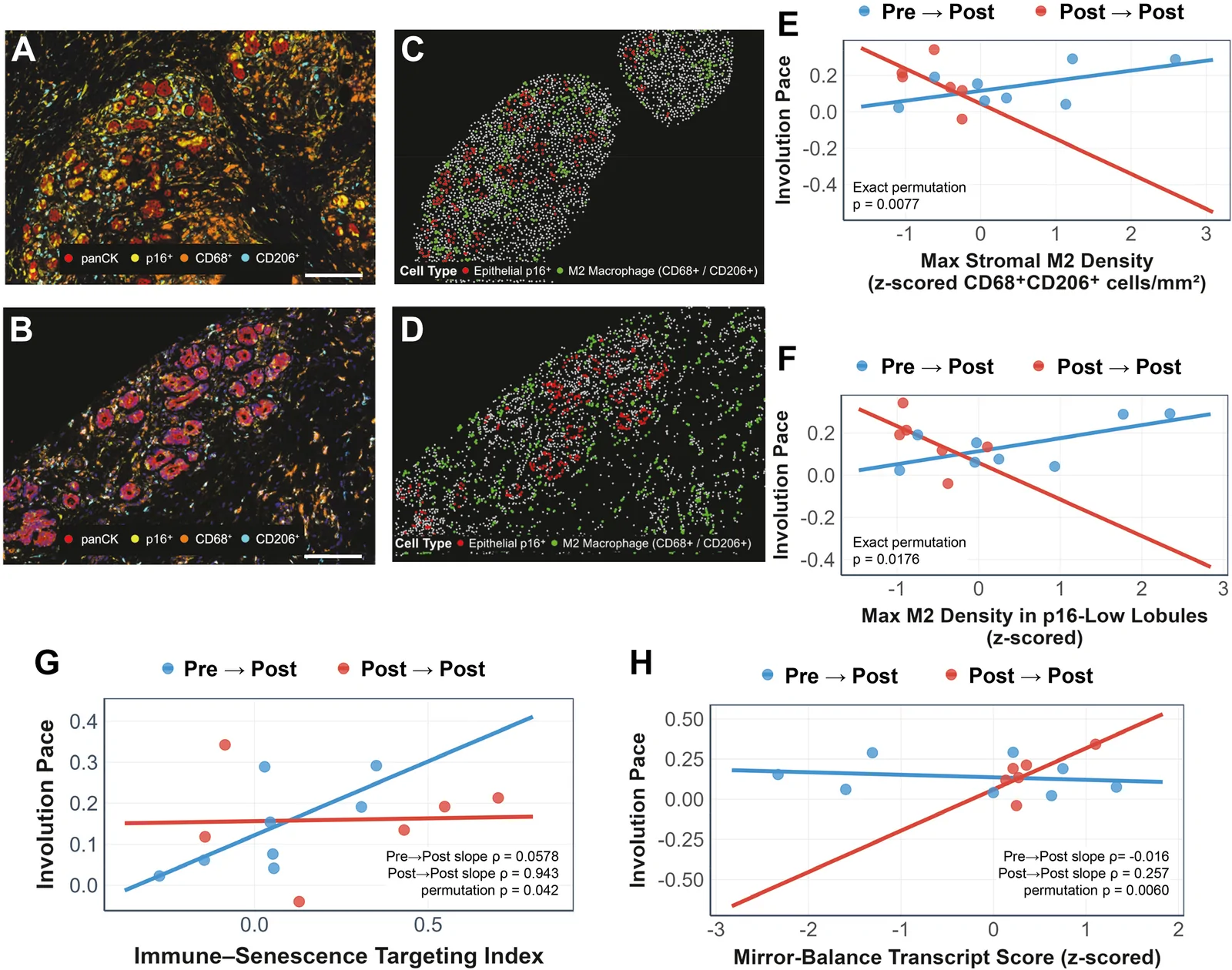
**M2-like macrophage targeting of p16^+^ epithelium reverses its association with breast ageing trajectory depending on menopausal timing.** (A, B) Representative baseline lobules (CD68/CD206/p16 MxIF) showing a targeted architecture with dense CD68^+^CD206^+^ (M2-like) macrophages apposed to epithelial p16^+^ foci (A) versus a spatially decoupled pattern with macrophages present but not positioned at p16^+^ epithelium (B) (panCK/CK red, p16 yellow, CD68 orange, CD206 cyan; scale bar, 200 μm). (C, D) Corresponding centroid maps for (A) and (B), showing epithelial p16^+^ cells (red), CD68^+^CD206^+^ macrophages (green), and all other segmented cells (grey). (E) Per-woman maximum stromal CD68^+^CD206^+^ density across lobules (hotspot metric; z-scored) versus involution pace, stratified by menopausal trajectory (Pre→Post vs Post→Post). Each point is one woman (n = 14; Pre→Post n = 8; Post→Post n = 6); lines show within-trajectory linear fits. Effect modification was tested with an interaction model relating involution pace to the metric, trajectory, and their interaction (specification in Methods), using a two-sided exact permutation p_int for the interaction (group sizes fixed) (p_int_ = 0.0077). (F) As in (E) using the maximum stromal CD68^+^CD206^+^ density among p16-low lobules (below each woman's 80th percentile of epithelial p16 density) (two-sided exact permutation p_int = 0.0176). (G) Immune–senescence targeting index versus involution pace (two-sided exact permutation p_int = 0.042); the targeting index is the within-woman correlation (across p16-low lobules) between stromal CD68^+^CD206^+^ density and epithelial p16 density. (H) Transcript mirror-balance score versus involution pace (two-sided exact permutation p_int = 0.0060): mean z(*IL6, STAT3, FCGR3A, FCGR3B*) − mean z(*MSR1, MRC1, CD163, CSF1R, ITGAM, CD68*), with z-scoring across women. Women are the unit of inference (n = 14; Pre→Post n = 8; Post→Post n = 6). Statistical tests. Interaction P values in (E–H) are two-sided exact permutation tests on the interaction term, permuting trajectory labels across women (Pre→Post n = 8; Post→Post n = 6); within-trajectory lines are per-woman ordinary least-squares fits.

The clearance hub architecture implicates CD16^+^ innate effectors in direct engagement of p16^+^ epithelium, but productive clearance likely also requires myeloid effectors capable of phagocytosing apoptotic debris. We therefore examined M2-like macrophage organisation as an orthogonal test of whether myeloid targeting of p16^+^ foci shows the same menopause-conditioned pattern. Representative baseline lobules from the CD68/CD206/p16 MxIF panel illustrate two patterns: a targeted architecture with dense CD68^+^CD206^+^ macrophages extending through stroma (and into epithelium) and tightly apposed to epithelial p16^+^ foci, versus a decoupled pattern in which macrophages are present but not positioned at p16^+^ epithelium (Fig. 7A–D).

In Post→Post women, higher macrophage hotspot density was associated with faster involution, whereas in Pre→Post women the association reversed, a menopause-conditioned effect modification that was significant (two-sided exact permutation p_int_ = 0.0077; Fig. 7E). This trajectory-dependent pattern persisted outside the p16-high tail. Restricting to p16-low lobules (below each woman's 80th percentile of epithelial p16 density), maximum stromal M2-like density remained menopause-modified (two-sided exact permutation p_int_ = 0.0176; Fig. 7F).

A macrophage hotspot in one lobule need not reflect coordinated targeting of senescent cells specifically; macrophages might simply be enriched where tissue remodelling is most active regardless of p16 status. To test whether macrophage enrichment specifically tracked p16^+^ epithelial foci, we computed macrophage–p16 coordination using an immune–senescence targeting index: within each woman (across p16-low lobules), the correlation between stromal CD68^+^CD206^+^ density and epithelial p16 density. The targeting index also showed menopause-dependent association with pace (two-sided exact permutation p_int_ = 0.042; Fig. 7G), indicating that menopausal timing conditions whether macrophage enrichment tracks senescent epithelial foci across lobules.

As an orthogonal, transcript-level test of the same clearance-versus-persistence axis, independent of the spatial geometry, we evaluated a mirror-balance score contrasting persistence-associated *IL6/STAT3/FCGR3A/B* against clearance/efferocytosis-associated myeloid transcripts *MSR1/MRC1/CD163/CSF1R/ITGAM/CD68*. This score likewise showed strong menopause-dependent effect modification (two-sided exact permutation p_int_ = 0.0060; Fig. 7H), supporting the clearance-associated interpretation through a modality that does not depend on the spatial readouts. Together, these findings provide convergent evidence, from spatial imaging of macrophage positioning, macrophage–p16^+^ cell coordination across lobules, and orthogonal transcript-level signatures, that the spatial organisation of myeloid clearance programmes differs across the menopausal transition, consistent with the menopause-conditioned direction reversal identified both transcriptomically and spatially.

## Discussion

After menopause, incomplete age-related lobular involution leaves a persistent, epithelial-rich breast state associated with higher breast density and greater breast cancer risk. Here we show that this state is not captured simply by how much senescence-linked inflammation is present, but by how p16^+^ (senescence-associated) epithelial cells and immune effectors are spatially organised across the menopausal transition. An EGFR–STAT3–innate hub that associates with stalled remodelling in women traversing menopause associates instead with faster ongoing regression in women already postmenopausal, and this direction reversal is mirrored across transcriptomic and spatial readouts. These findings position menopause not as a demographic covariate, but as a physiologic context that conditions how senescent–immune programmes relate to involution outcome and helps explain persistence of a risk-associated postmenopausal breast state.

Viewed translationally, persistent postmenopausal lobules represent a risk-relevant tissue state defined not only by retained epithelial mass but also by retained proliferative capacity, clustered p16^+^ epithelial foci, and abundant leukocytes that remain spatially decoupled from epithelial targets. This extends prior morphometric risk models based mainly on epithelial quantity^7^^,^^8^^,^^54^ and suggests that the spatial organisation of immune surveillance within the breast may help distinguish persistent from resolving postmenopausal tissue states. It also provides a framework for interpreting why senescence/SASP and innate inflammatory biomarkers may perform inconsistently if women are not anchored to menopausal timing.

A key interpretive insight from the longitudinal design is that the same persistence-prone baseline state carries different meaning depending on its relationship to the menopausal transition. In Pre→Post women, regression reflects traversing the expected remodelling window; stagnation reflects failure to execute it. In Post→Post women, the same baseline state represents persistence beyond that window, and regression resembles late catch-up remodelling. This framing has broader implications: senescence-linked inflammatory hubs are not intrinsically pro- or anti-remodelling, and their association with tissue outcomes will appear inconsistent, or even contradictory, across studies that sample women at different points relative to menopause without accounting for that timing. Menopausal physiology and sampling timing together condition whether these hubs couple to clearance-competent execution or persistence-associated organisation.^5^^,^^54^, ^55^, ^56^

Recent single-cell and spatial atlases provide population-level context for these findings. The Human Breast Cell Atlas mapped a rich tissue-resident immune ecosystem within ducts and lobules and resolved ductal–lobular molecular differences,^57^ and a complementary scRNA-seq atlas localised the principal age-related changes to the epithelium (lineage-fidelity shifts), with selected fibroblast changes and plasma-cell depletion, although it could not assess menopause separately.^58^ A spatial-proteomic analysis of 527 breasts subsequently showed pronounced lobular loss, reduced cellularity and proliferation, fewer epithelial–microenvironment interactions, and relative enrichment of M2 macrophages, with several microenvironment populations positioned farther from the epithelium in older tissue, most marked around the menopausal transition; the breast arm of a pan-tissue single-cell study independently identified a menopause-associated breast fibroblast trajectory.^9^^,^^59^ Murine single-cell, epigenomic, and spatial data similarly show pro-inflammatory epithelial programmes, senescence- and cancer-associated fibroblast states, and expansion of M2-like macrophage and Gzmk^+^ T-cell populations with age, although mice lack the endocrine menopausal transition.^60^ Our findings are concordant with this broad remodelling but indicate that persistent postmenopausal lobules represent a selected residual state within the average age-related decline: they retain proliferation-competent epithelium and abundant immune cells, yet immune abundance is not equivalent to target-proximal positioning around p16-positive epithelial foci. As these atlases are cross-sectional, our paired design adds longitudinal involution pace and individual timing relative to menopause; the association of senescence-associated and innate programmes with remodelling varied across the transition, with the interaction providing the primary evidence for this context dependence.

Recent benign-breast and mammary-involution studies add a risk- and function-oriented layer to this atlas context. Image-based analyses of benign breast disease biopsies showed that senescence-associated nuclear morphologies in epithelial and adipose compartments predicted subsequent invasive breast cancer risk, with stronger associations among postmenopausal women and women with epithelial hyperplasia.^61^ Although postpartum involution is distinct from ARLI, experimental work in mice indicates that p16^+^-dependent senescence in alveolar luminal cells can be required for normal mammary remodelling, yet can also promote tumourigenic behaviour when oncogenic activation coincides with involution.^62^ Conversely, parity and lactation studies show that a complete lactation–involution cycle can durably remodel breast immunity, increasing CD8^+^ tissue-resident-memory-like cells in normal human breast tissue and supporting CD8^+^ T-cell-dependent tumour control in experimental models.^63^ Together, these studies reinforce a context-dependent model in which senescence-associated programmes can contribute to physiological remodelling, immune surveillance, or risk-associated persistence depending on tissue state, timing, and immune execution capacity.

This pattern fits a broader principle of adaptive-reserve maintenance documented across multiple organ systems in which functional tissue units are actively sustained under hormonal withdrawal or chronic stress, preserving architecture at the cost of elevated disease risk when the reserve becomes chronically maintained.^18^, ^19^, ^20^, ^21^, ^22^, ^23^, ^24^ In the breast, this reframes the persistent lobule not as a remnant of incomplete regression but as a proliferation-competent epithelial compartment sustained by active senescent–immune interaction programmes. We therefore use *stall* to describe the involution outcome and *adaptive-reserve maintenance* to describe a possible mechanism; the same tissue state can be viewed as sustained from the standpoint of tissue integrity and stalled from the standpoint of long-term risk.

The spatial data further argue that this biology unfolds at the level of individual lobules rather than uniformly across the breast, consistent with recent murine mammary ageing work showing asynchronous, progressive senescence programmes that precede cancer-associated transcriptional vulnerability.^64^ In our study, greater within-breast patchiness in hub markers marked slower involution, and EREG-rich field geometry showed opposite associations with pace depending on menopausal context. This is consistent with ARLI proceeding through asynchronous transitions of repeated microanatomical units, so that a minority of lobules may remain trapped in persistence-prone states while others continue to regress. Similar mosaic logic has been described in other tissues composed of repeating units, where local epithelial programmes interact with stromal cues, mechanics, and immune surveillance.^10^, ^11^, ^12^, ^13^, ^14^, ^15^, ^16^, ^17^ In such settings, bulk inflammatory or senescence signatures need not have a single directional relationship with outcome; the same programme can mark stalled units in one physiologic state and effectively remodelling units in another. Our menopause-conditioned sign reversals provide a concrete breast example of this broader context dependence.

At the lobule scale, the distinction between stalled and resolving remodelling mapped onto two immune–senescent architectures that differed more in execution than in inflammatory input. A stall architecture was characterised by p16^+^ epithelial foci and CD16^+^ cells compactly coupled to a surrounding pSTAT3^+^ epithelial field, consistent with recruitment and signalling engagement without consistent target clearance. A clearance-associated architecture was characterised by abundant innate effectors with direct CD16→p16 engagement and by convergent evidence of M2-like macrophage targeting of p16^+^ epithelium, and was more frequent in faster-involuting women who were postmenopausal at both biopsies. These data support an execution-limited model in which the relevant distinction is not how much innate inflammation is present, but whether immune effectors are positioned to engage and clear p16^+^ targets. Recent senescence-immunosurveillance studies identify candidate mechanisms by which p16-positive or senescent cells can persist despite immune-cell presence, providing plausible routes by which immune-rich lobules could remain execution-limited.^65^^,^^66^ Within this model, STAT3 is a plausible integrative node because it is known to link SASP- and cytokine-associated cues to either productive remodelling or chronic inflammatory persistence depending on spatial context and immune execution capacity,^49^, ^50^, ^51^, ^52^, ^53^ making it a candidate integrator through which the same inflammatory inputs yield different tissue outcomes across the menopausal transition. Our results are consistent with EGFR/IL6-family→STAT3 signalling operating in a spatial context that shifts across menopause, associated with stall-hub architectures in which innate effectors are recruited but p16^+^ targets are not consistently engaged during the transition, and with clearance-associated architectures marked by direct innate engagement of p16^+^ epithelium afterwards. In this view, the menopause-conditioned reversal reflects reorganisation of immune execution capacity and spatial targeting rather than a change in inflammatory inputs.

These findings suggest several breast-specific implications. First, incomplete involution may be better understood as a spatially defined, risk-relevant postmenopausal tissue state rather than a morphometric endpoint alone. Second, senescence/SASP and innate immune biomarkers are menopause-state dependent; future biomarker models should distinguish women traversing menopause from those already postmenopausal rather than pooling them. Third, the direction reversal identifies a plausible window for intervention around the menopausal transition. Stall architectures during the transition may reflect immune recruitment without execution, whereas clearance implementations detected after menopause may index productive remodelling. This motivates testable strategies to destabilise stall states during the transition or to promote clearance after menopause, including approaches that modulate senescent-cell survival, paracrine reinforcement of senescent microenvironments, or macrophage-mediated efferocytosis.^2^^,^^67^, ^68^, ^69^, ^70^, ^71^

These conclusions should be interpreted in light of several limitations. Imaging and endpoint cohorts were modest and trajectory stratification further reduced power; we therefore based inference on woman-clustered robust standard errors corroborated by exact permutation tests and, for clearance-hub modelling, Bayesian partial pooling, and we treat the sign-reversal effects as robust in direction but provisional in magnitude, requiring replication in larger cohorts; in particular, the follow-up interface interactions (Fig. 3G and H) are directionally consistent but, at n = 14, are not individually significant under assumption-light (permutation) inference, and the reversal is most firmly supported by the larger transcriptomic cohort (Fig. 4) and the permutation-based macrophage analyses (Fig. 7). Consistent with guidance emphasising that in vivo senescence assignment is tissue- and context-dependent,^72^^,^^73^ senescence was indexed here by epithelial p16 immunopositivity alone; we therefore describe p16^+^ (senescence-associated) epithelium rather than definitively senescent cells. Our cohorts comprise benign breast tissue without linked incident-tumour subtype data; whether persistence-associated tissue states preferentially precede ER-positive cancers, and whether such cancers differ biologically from typical ER-positive disease, will require studies linking these tissue states to the subtype of subsequent tumours. Additionally, trajectory stratification reduced power for interaction tests, the targeted transcriptomic and MxIF panels limited immune-state resolution and direct in situ assessment of efferocytosis or exhaustion, and clearance-hub inference relied on a mean-field variational approximation, which can understate posterior uncertainty. Relatedly, our clearance endpoints quantify the spatial positioning of innate effectors relative to p16^+^ epithelium and do not directly assess apoptosis, efferocytosis, or effector functional states in situ; we therefore describe a clearance-associated configuration rather than a demonstrated clearance event, with functional confirmation (e.g., apoptosis or phagocytosis readouts) left to future work. Registry-reported age at menopause provided a practical physiologic anchor but not a direct hormonal measure, and interval misclassification is possible near the menopausal boundary. Involution pace is, moreover, an interval-averaged annualised rate, so its instantaneous value may vary across the transition and two biopsies cannot resolve that trajectory; our years-from-menopause interaction framework accommodates this timing dependence but cannot substitute for the denser longitudinal sampling needed to characterise the pace trajectory itself. Marker specificity also constrains lineage assignment, particularly for CD16, so we emphasise spatial engagement patterns over definitive immune subtype calls. Finally, specimens derive from benign breast disease biopsies rather than screening-normal tissue, and the key sign-reversal effects are supported by many lobules but relatively few women. These considerations temper causal inference and the associations reported here are observational and hypothesis-generating, identifying menopausal timing as a context for interpretation rather than establishing causation.

In summary, incomplete involution after menopause is a persistent, risk-associated breast tissue state whose biology is conditioned by how p16^+^ epithelial cells and immune effectors are spatially organised across the menopausal transition. Menopausal timing helps determine whether senescence-linked inflammatory programmes align with stalled remodelling or with clearance-associated regression, and should be treated as a physiologic context for biomarker interpretation, woman stratification, and future breast cancer prevention studies.

## Contributors

**Jaida C. Lue**: Conceptualisation, Investigation, Methodology, Formal analysis, Visualisation, Writing—original draft, Writing—review & editing, Data access and verification. **Melody Stallings-Mann**: Investigation, Methodology, Writing—review & editing. **Tanya Hoskin**: Formal analysis, Methodology, Writing—review & editing. Bryan M. **McCauley**: Formal analysis, Methodology, Writing—review & editing. **Robert A. Vierkant**: Formal analysis, Methodology, Writing—review & editing. **Laura Pacheco-Spann**: Data curation, Writing—review & editing. **Jessica L. Fischer**: Data curation, Writing—review & editing. **Denice L. Gehling**: Data curation, Writing—review & editing. **Lisa R. Seymour**: Data curation, Writing—review & editing. **Darren J. Baker**: Resources, Writing—review & editing. **Amy C. Degnim**: Resources, Funding acquisition, Writing—review & editing. **Stacey J. Winham**: Formal analysis, Methodology, Funding acquisition, Writing—review & editing. **Mark E. Sherman**: Formal analysis, Resources, Funding acquisition, Writing—review & editing. **Derek C. Radisky**: Conceptualisation, Supervision, Project administration, Funding acquisition, Writing—original draft, Writing—review & editing, Data access and verification. The corresponding author had full access to the data and final responsibility for the decision to submit. All authors have read and approved the final version of the manuscript.

## Data sharing statement

Derived spatial-feature data (per-lobule and per-woman phenotype counts, densities, nearest-neighbour distances, contact fractions, EXCESS curves, and hub classifications) and the spatial-metric construction code underlying all imaging analyses are available to qualified researchers from the corresponding author under a data-use agreement with Mayo Clinic IRB approval, owing to the identifiable nature of the linked clinical and whole-slide-image data. Baseline gene-expression data (GEO GSE320321) and analysis code (Zenodo https://doi.org/10.5281/zenodo.18775052) are publicly available.

## Declaration of interests

D.J.B. has a potential financial interest related to this research. He is a co-inventor on patents held by Mayo Clinic, patent applications licenced to or filed by Unity Biotechnology, and a Unity Biotechnology shareholder. Research in the Baker laboratory has been reviewed by the Mayo Clinic Conflict of Interest Review Board and is being conducted in compliance with Mayo Clinic Conflict of Interest policies. All other authors declare no competing interests.

## Appendix A Supplementary data

Supplementary Tables

Key Resources and Reagents Table with RRIDs
