# Supplementary material for: Menopausal timing and senescent–immune coupling in age-related lobular involution of the human breast: a longitudinal cohort study
Source: eBioMedicine. 2026 Aug 31;131:106463. doi: 10.1016/j.ebiom.2026.106463 (PMC13560535; doi:10.1016/j.ebiom.2026.106463)
Supplement: Supplementary file 1 [file mmc1.pdf]

Figure S1. Transcriptomic detail in postmenopausal noninvoluted tissue

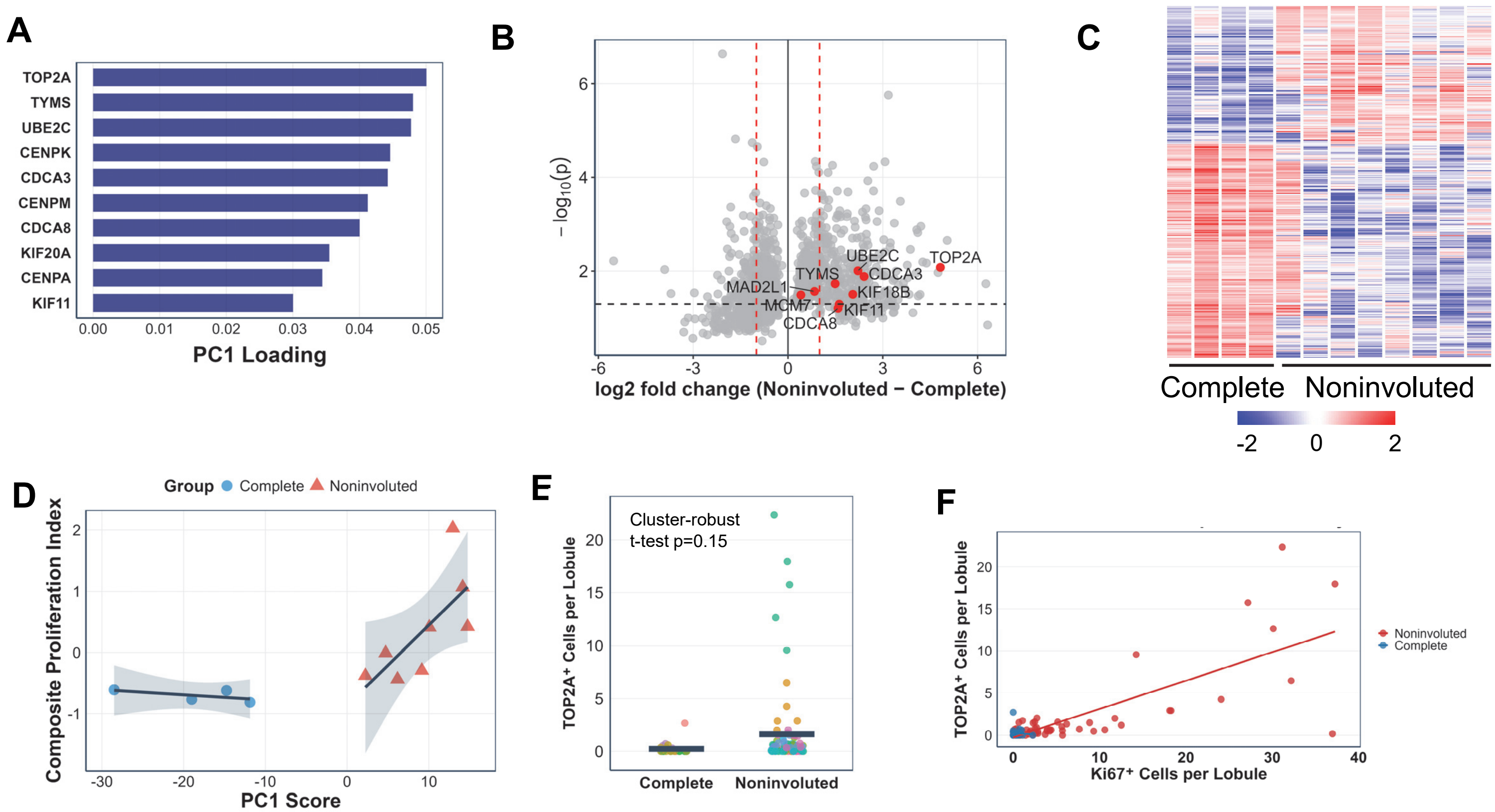

Figure S1. Transcriptomic detail in postmenopausal noninvoluted tissue. (A) PC1 loadings for canonical proliferation/cell-cycle genes within the PCA feature set (top 500 most variable DASL genes). (B) Volcano plot of differential expression (noninvoluted vs completely involuted) with proliferation-panel genes labelled. (C) Heat map of the top differentially expressed genes (ranked by nominal P), z-scored by gene, with hierarchical clustering of genes and samples. (D) Composite proliferation index versus PC1 score. (E) TOP2A$^+$ epithelial nuclei density per lobule quantified from IHC. (F) Lobule-level coupling of Ki67 and TOP2A (z-scored within group); points, lobule ROIs coloured by woman; open symbols, woman medians. The within-group Pearson correlation between Ki67 and TOP2A is strong in noninvoluted tissue ($r = 0.76$, $R^2 = 0.58$; 8 women, 80 lobule ROIs) but absent in completely involuted tissue ($r = -0.07$, $R^2 < 0.01$; 4 women, 27 lobule ROIs), quantifying the tighter proliferative coupling in persistent lobules; the fitted line is the noninvoluted within-group regression. Statistical tests. (B) Welch's two-sample t-test per probe (noninvoluted vs completely involuted); dashed lines mark the log2FC and −log10(P) cutoffs. (E) Cluster-robust t-test (noninvoluted n = 8 women; completely involuted n = 4 women). (F) Linear regression with patient-clustered robust standard errors (Ki67–TOP2A coordination); r and R^2 shown.

Figure S2. Boundary localization, joint modeling, and compartment-resolved decomposition of senescent–immune miscoordination

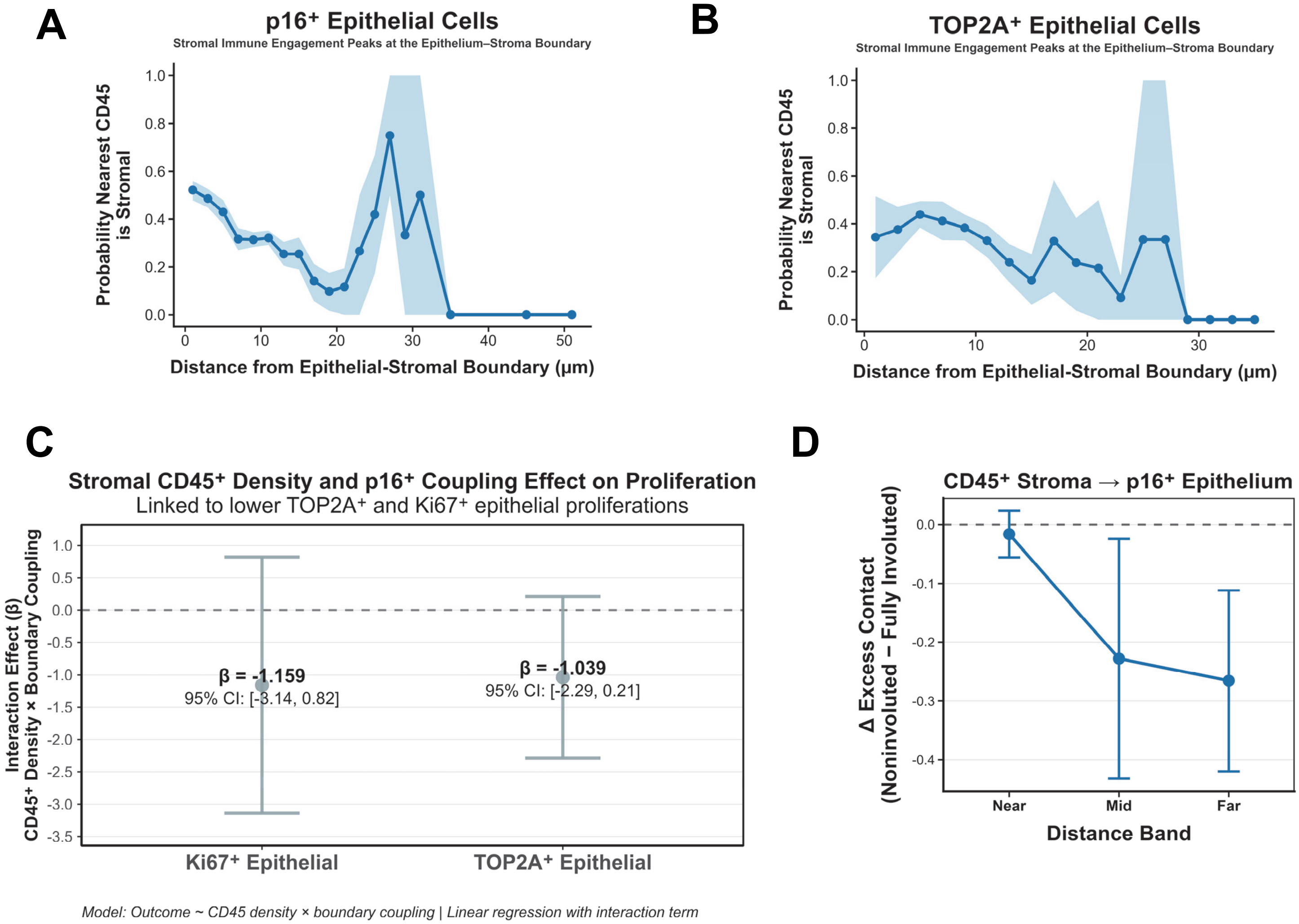

Figure S2. Boundary localisation, joint modelling, and compartment-resolved decomposition of senescent–immune miscoordination. (A,B) Distance-to-boundary gradients localise cross-interface immune proximity to the epithelium–stroma interface: for each epithelial p16⁺ (A) or TOP2A⁺ (B) source cell, plots show the probability that its nearest CD45⁺ neighbour resides in stroma as a function of the source distance from the epithelial boundary. (C) Joint-model interaction term (β ±95% CI) for stromal CD45⁺ density × boundary p16→stromal CD45 coupling in linear models predicting epithelial Ki67⁺ or TOP2A⁺ density (more negative β indicates a stronger inverse association between immune burden and proliferation when boundary coupling is high). (D) Compartment-resolved, band-averaged CSR-adjusted enrichment for stromal CD45⁺→epithelial p16⁺ positioning (ΔG_ES). EXCESS is averaged within Near (0–15 μm), Halo/Mid (15–45 μm), and Far (45–100 μm) bands and plotted as the group contrast (noninvoluted − completely involuted; patient-bootstrap 95% CIs). Statistical tests. (C) Linear regression; interaction term shown as β ± 95% CI. (D) CSR-adjusted EXCESS contrast (noninvoluted − completely involuted) with patient-bootstrap 95% CIs. (A,B) Descriptive distance-to-boundary gradients (no hypothesis test). Endpoint cohort: noninvoluted n = 8 women; completely involuted n = 4 women.

Figure S3. Robustness and multiscale validation of follow-up immune–senescence interface endpoints

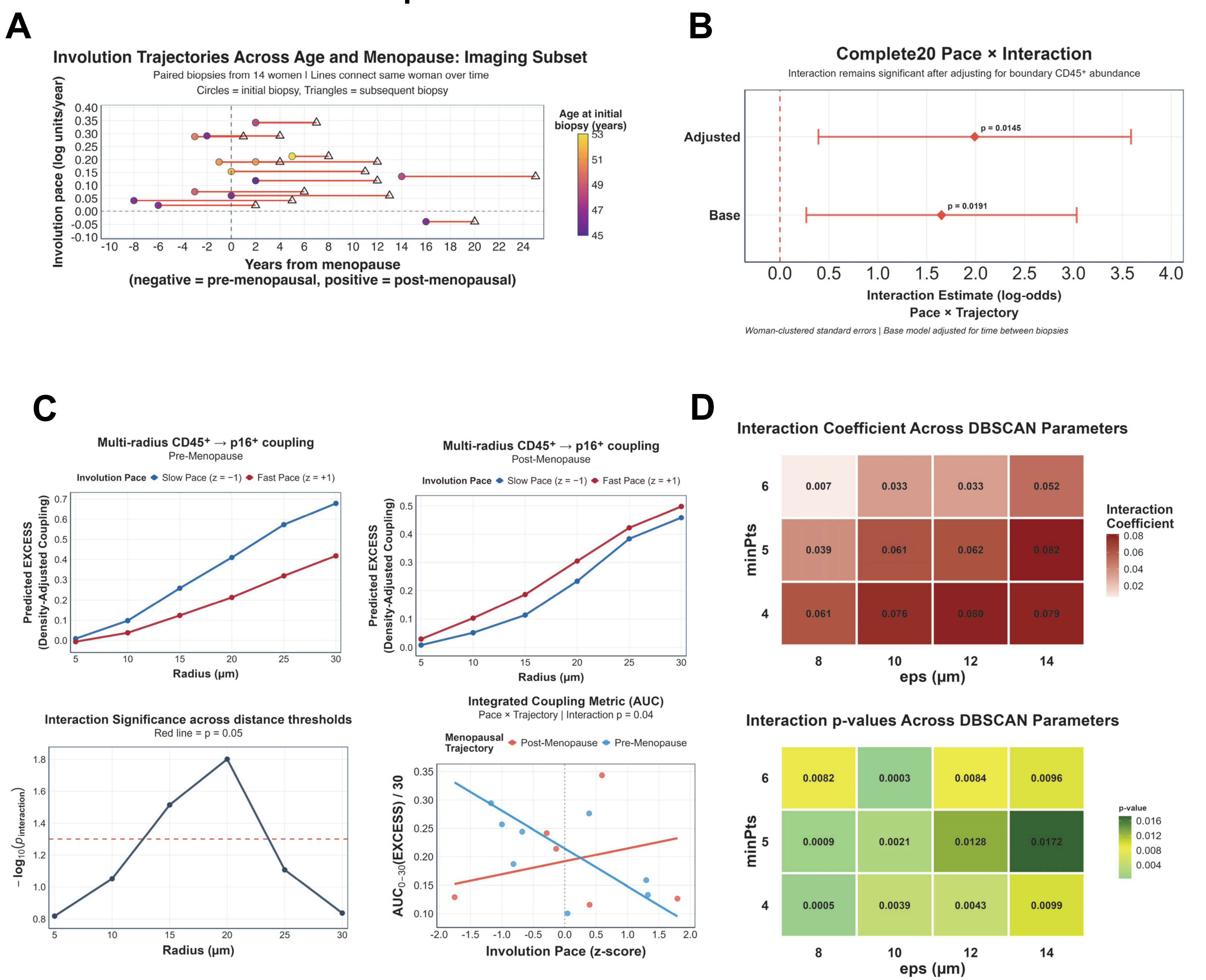

Figure S3. Robustness and multiscale validation of follow-up immune–senescence interface endpoints. (A) Swimlane visualisation for the evaluable follow-up MxIF subset with known menopause age (n=14 women; 8 Pre→Post, 6 Post→Post), anchored to years-from-menopause, baseline (filled circles) and follow-up (open triangles) positions shown for each woman. (B) Robustness of the Complete20 pace×trajectory interaction to boundary CD45 abundance. Interaction estimates (log-odds scale) from lobule-level logistic regression are shown for the base model (pace×trajectory + inter-biopsy interval) and for the model additionally adjusted for log(boundary $CD45^{+}$ source count), using woman-clustered robust standard errors. (C) Multi-radius density-adjusted coupling for boundary stromal CD45→epithelial p16 at follow-up. For r=5–30 µm, EXCESS(r) = CF(r) − CF_CSR(r), where CF_CSR(r) is the CSR expectation conditioned on epithelial p16 density; curves show fitted EXCESS(r) profiles for pace_z = −1 versus +1 within each trajectory. Additional panels summarise interaction significance across radii and the relationship between AUC(EXCESS_0–30) and pace. (D) DBSCAN parameter-stability analysis for boundary CD45 clustering fraction across ε (8–14 µm) and min_samples (4–6), showing stability of the pace×trajectory interaction magnitude and significance. Statistical tests. (B,C,D) Logistic GLM with woman-clustered robust standard errors; two-sided Wald test on the pace×trajectory interaction, shown for the base and CD45-adjusted models (B), across radii (C), and across DBSCAN ε and min_samples (D) (n = 14 women; 8 Pre→Post, 6 Post→Post). (A) Descriptive swimlane (no test).

Figure S4. Gene-level detail, module localization, and robustness supporting the menopause-conditioned transcript reversal

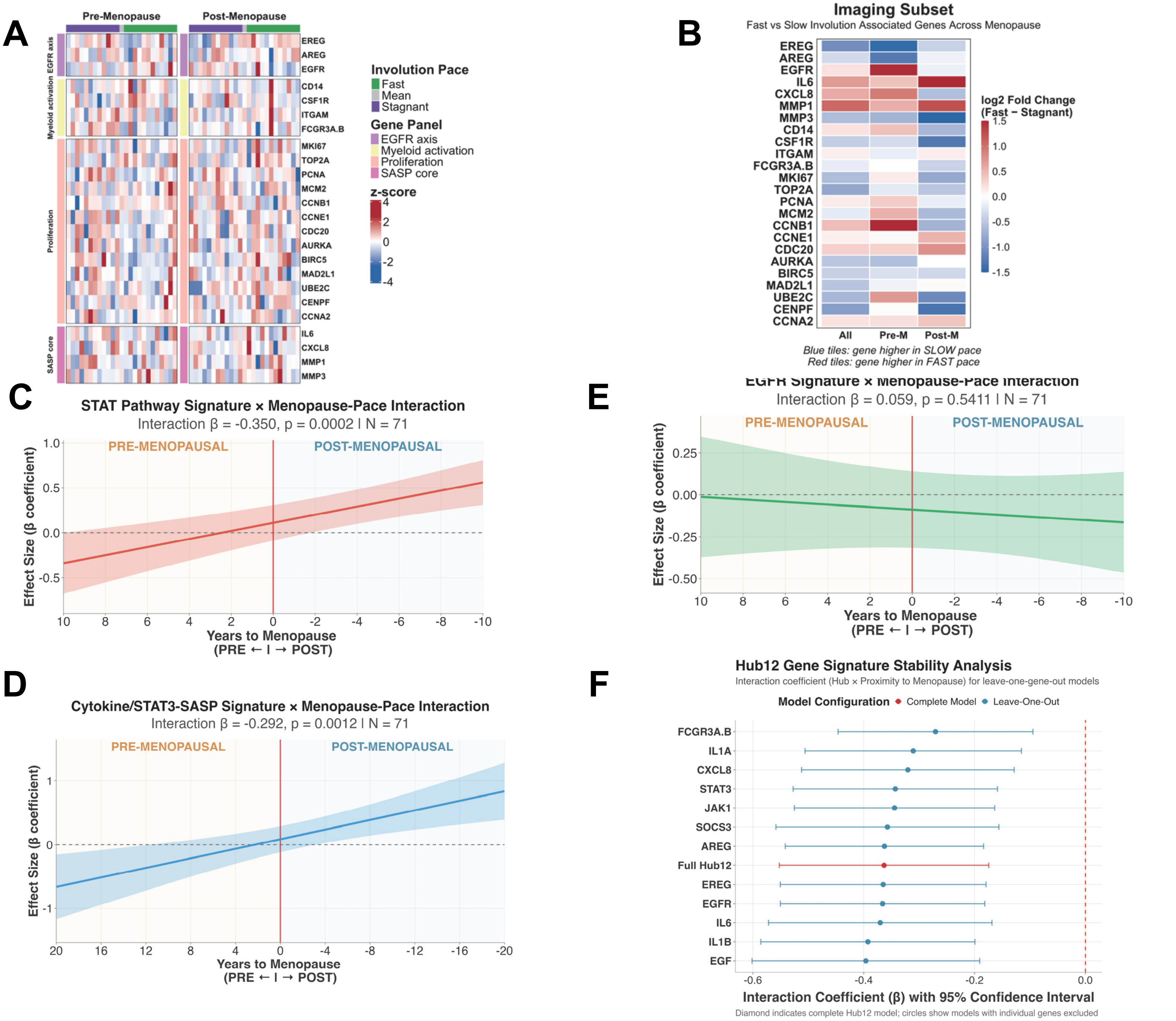

Figure S4. Gene-level detail, module localisation, and robustness supporting the menopause-conditioned transcript reversal. (A) Baseline expression of the pre-specified gene set shown as gene-wise z-scores within each trajectory stratum, with individuals ordered by pace. Columns show the STAGNANT extreme, the FAST − STAGNANT contrast, and the FAST extreme to visualise within-stratum heterogeneity. (B) Targeted gene summary computed within the 16-woman spatial-imaging subset, supporting consistent directionality in the deeply profiled cohort. (C-E) Submodule localisation across menopausal physiology using the same feature×years-from-menopause interaction framework as Fig. 4 (pace ~ feature + years-from-menopause + feature×years-from-menopause + follow-up interval + baseline morphometrics; continuous predictors z-scored). Curves show the model-implied standardised feature→pace slope (±95% CI) as a function of baseline years-from-menopause for: (C) STAT3-axis spine, (D) cytokine/STAT3–SASP module, and (E) EGFR ligand-field module (gene membership in Supplementary Table S6). (F) Leave-one-gene-out recomputation of Hub12 followed by refitting the Hub12×years-from-menopause model; the direction of the menopause-conditioned reversal is preserved across omissions. Statistical tests. (C,D,E) OLS interaction model (submodule×years-from-menopause); two-sided Wald test on the interaction term (n = 71 women). (F) Leave-one-gene-out recomputation of the same model; interaction coefficients shown as β ± 95% CI. (A,B) Descriptive gene-level heatmaps (no test).

Figure S5. Robustness and sensitivity analyses for the menopause-dependent sign flip in baseline hub engagement versus involution pace

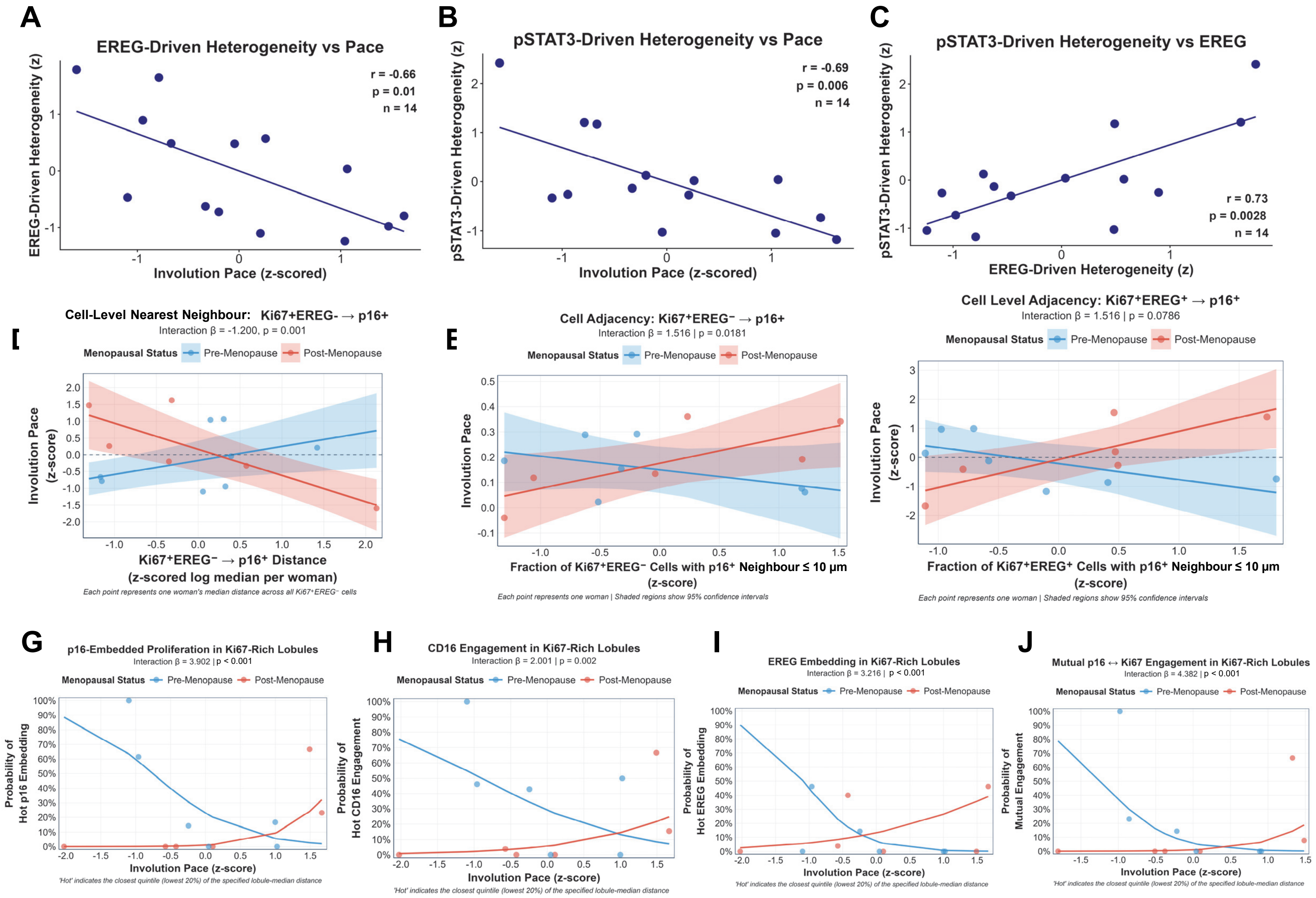

Figure S5. Robustness and sensitivity analyses for the menopause-dependent sign flip in baseline hub engagement versus involution pace. (A-C) HubPatchiness components: EREG-driven between-lobule variance versus pace (A), pSTAT3-driven variance versus pace (B), and correlation between the two components (C) (n = 14 women). (D-F) Metric robustness for the same proliferative–senescent interface question (per-woman endpoints). Each panel summarises, per woman, proximity/adjacency between proliferative epithelium and senescent epithelium using different but aligned definitions; models test menopause-dependent effect modification (pace as outcome; endpoint×menopausal trajectory as effect modifier) among women with known registry menopause age (trajectory-defined subset n = 14; effective n varies by endpoint based on cell-state abundance/QC). (D) Continuous distance metric: median nearest-neighbour distance from Ki67$^+$EREG$^-$ epithelial sources to p16$^+$ epithelial targets (smaller = tighter engagement). (E) Adjacency fraction (CF10): fraction of Ki67$^+$EREG$^-$ sources with a p16$^+$ neighbour within 10 µm. (F) Source-subset sensitivity: CF10 computed using Ki67$^+$EREG$^+$ sources (tests that conclusions are not specific to EREG-negative proliferators). (G-J) Robustness in Ki67-rich lobules (≥5 Ki67$^+$EREG$^-$ sources per lobule) from trajectory-defined women. "Hot" indicates the closest quintile (lowest 20%) of the specified lobule-median distance (or highest quintile where noted). Lobule-level binomial GLMs test hot ~ pace × menopausal physiology/trajectory with woman-clustered inference: (G) hot p16-embedded proliferation (Ki67$^+$EREG$^-$→p16$^+$), (H) hot innate engagement bridge (Ki67$^+$EREG$^-$→CD16$^+$), (I) hot EREG embedding (Ki67$^+$EREG$^-$→EREG$^+$), and (J) stringent mutual-engagement composite requiring hot p16 embedding and high reverse-direction coupling (top quintile CF10 p16→Ki67), emphasising bidirectional interface coupling. Statistical tests. (A,B,C) Pearson correlation (n = 14 women). (D–J) Logistic (binomial) GLM with woman-clustered robust standard errors; two-sided Wald test on the pace×trajectory (or pace×menopausal-physiology) interaction (trajectory-defined subset n = 14 women; effective n varies by endpoint as stated).

Figure S6. Secondary endpoints supporting stall architecture and Bayesian partial-pooling clearance inference

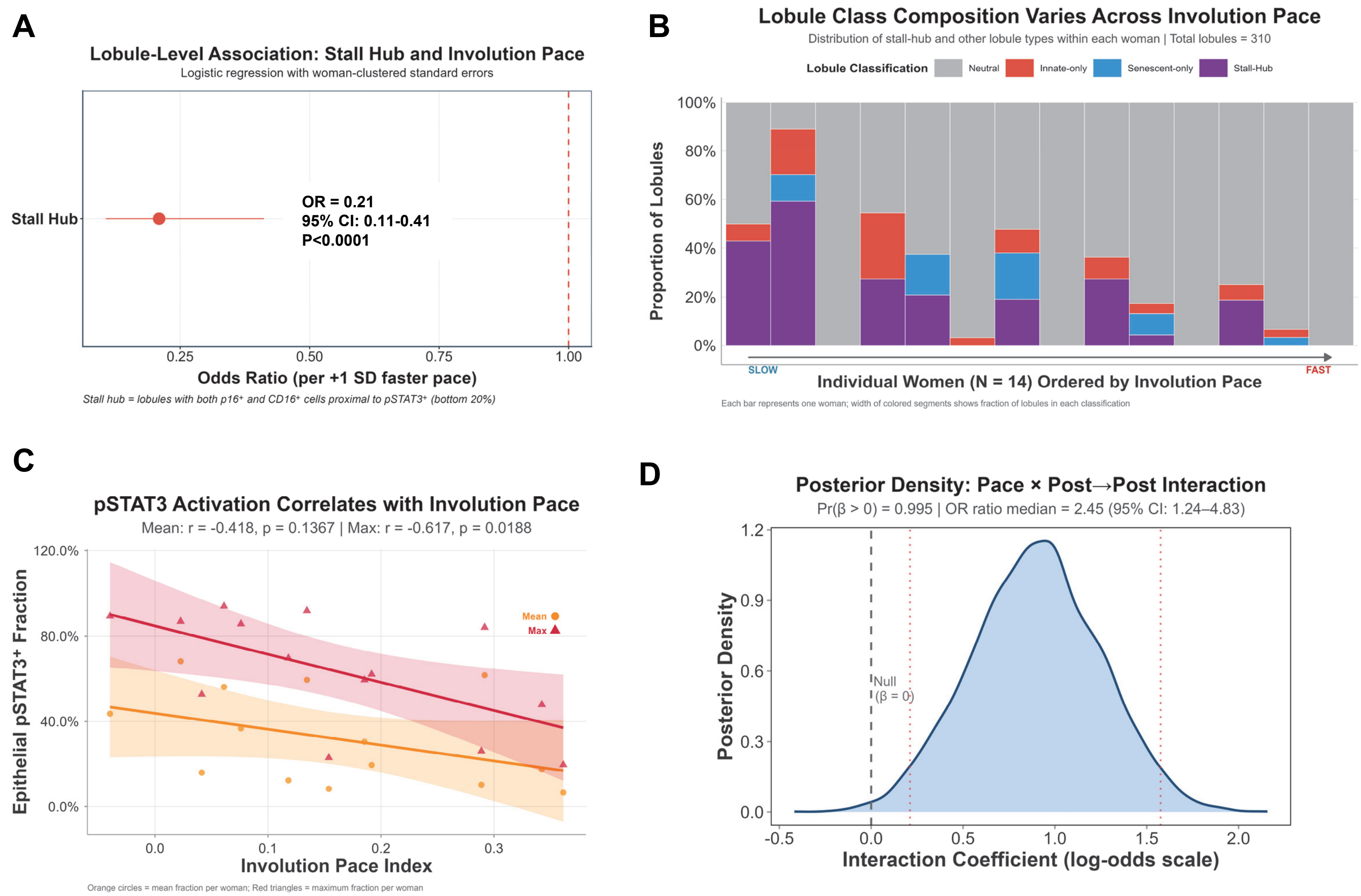

Figure S6. Secondary endpoints supporting stall architecture and Bayesian partial-pooling clearance inference. (A) Lobule-level odds model for stall-hub odds versus involution pace (cluster-robust CI; N = 14 women, 354 lobules). (B) Lobule-class composition (neutral, innate-only, senescent-only, stall hub) across women ordered by involution pace. (C) Mean versus maximum epithelial pSTAT3$^+$ fraction per woman versus involution pace, illustrating minority-lobule ("tail") behaviour. (D) Posterior distribution (VB normal approximation) for the pace×Post→Post interaction coefficient in the clearance mixed model (Pr(β>0) and OR shown). Statistical tests. (A) Logistic regression with woman-clustered robust standard errors (odds ratio with 95% CI and Wald P; N = 14 women, 354 lobules). (C) Pearson correlation (mean and maximum per-woman pSTAT3$^+$ fraction; n = 14 women). (D) Bayesian partial-pooling model; posterior probability Pr(β > 0), OR ratio and 95% credible interval rather than a frequentist P value. (B) Descriptive lobule-class composition (no test).
