# Supplementary material for: Menopausal timing and senescent–immune coupling in age-related lobular involution of the human breast: a longitudinal cohort study
Source: eBioMedicine. 2026 Aug 31;131:106463. doi: 10.1016/j.ebiom.2026.106463 (PMC13560535; doi:10.1016/j.ebiom.2026.106463)
Supplement: Supplementary file 2 [file mmc2.docx]

**Table S1. Antibody clone/vendor/catalog/dilution and Opal fluor assignments.**

**Post-menopausal Biopsy Panel**

| **Antibody** | **Host** | **Vendor** | **Catalog** | **Dilution** | **Opal Fluor** |
| --- | --- | --- | --- | --- | --- |
| Ki67 | Mouse | DAKO | M7240 | 1:200 | Opal 520 (1:100) |
| P16 | Mouse | Santa Cruz | SC - 56330 | 1:600 | Opal 570 (1:100) |
| Top2A | Rabbit | Invitrogen | MA5-32096 | 1:1200 | Opal 620 (1:100) |
| CD45 | Rabbit | Abcam | Ab40763 | 1:300 | Opal 690 (1:100) |
| Pan-Cytokeratin | Mouse | DAKO | M3515 | 1:150 | Opal 780 (1:25) |
| DAPI |  | Akoya | FP1490 | 1:10 |  |

**Longitudinal Baseline Biopsy Panels**

**(i)**

| **Antibody** | **Host** | **Vendor** | **Catalog** | **Dilution** | **Opal Fluor** |
| --- | --- | --- | --- | --- | --- |
| P16 | Mouse | Santa Cruz | SC - 56330 | 1:400 | Opal 480 (1:150) |
| Ki67 | Mouse | DAKO | M7240 | 1:100 | Opal 520(1:100) |
| CD45 | Rabbit | Abcam | Ab40763 | 1:300 | Opal 570 (1:100) |
| EREG | Mouse | Santa Cruz | SC-376284 | 1:100 | Opal 690 (1:50) |
| Pan-Cytokeratin | Mouse | DAKO | M3515 | 1:100 | Opal 780 (1:25) |
| DAPI |  | AKOYA |  | 1:10 |  |

**(ii)**

| **Antibody** | **Host** | **Vendor** | **Catalog** | **Dilution** | **Opal Fluor** |
| --- | --- | --- | --- | --- | --- |
| Ki67 | Mouse | DAKO | M7240 | 1:100 | Opal 480 (1:150) |
| pSTAT3 | Rabbit | Abcam | AB76315 | 1:100 | Opal 520 (1:100) |
| P16 | Mouse | Santa Cruz | SC - 56330 | 1:400 | Opal 570 (1:50) |
| CD16A | Rabbit | Abcam | AB246222 | 1:500 | Opal 620 (1:50) |
| EREG | Mouse | Santa Cruz | SC-376284 | 1:100 | Opal 690 (1:50) |
| Pan-Cytokeratin | Mouse | DAKO | M3515 | 1:100 | Opal 780 (1:25) |
| DAPI |  | AKOYA |  | 1:10 |  |

**(iii)**

| **Antibody** | **Host** | **Vendor** | **Catalog** | **Dilution** | **Opal Fluor** |
| --- | --- | --- | --- | --- | --- |
| CD206 | Rabbit | Abcam | AB64693 | 1:200 | Opal 520 (1:100) |
| P16 | Mouse | Santa Cruz | SC - 56330 | 1:600 | Opal 570 (1:100) |
| CD68 | Rabbit | Abcam | AB125212 | 1:500 | Opal 690 (1:100) |
| Pan-Cytokeratin | Mouse | DAKO | M3515 | 1:150 | Opal 780 (1:25) |
| DAPI |  | AKOYA |  | 1:10 |  |

**Longitudinal Subsequent Biopsy Panel**

| **Antibody** |  | **Vendor** | **Catalog** | **Dilution** | **Opal Fluor** |
| --- | --- | --- | --- | --- | --- |
| Ki67 | Mouse | DAKO | M7240 | 1:100 | Opal 520 (1:100) |
| P16 | Mouse | Santa Cruz | SC - 56330 | 1:600 | Opal 570 (1:100) |
| CD45 | Rabbit | Abcam | Ab40763 | 1:300 | Opal 690 (1:100) |
| Pan-Cytokeratin | Mouse | DAKO | M3515 | 1:150 | Opal 780 (1:25) |
| DAPI |  | AKOYA |  |  | 1:10 |

**Table S2. PCA loadings for the most variable genes in postmenopausal DASL profile**

Principal component (PC) loadings from PCA performed on the top 500 most variable genes across postmenopausal benign breast lobular samples profiled by Illumina DASL (n=12; 8 noninvoluted, 4 completely involuted). Expression values were log2-normalized; each gene was z-scored across samples before PCA. PC1 was sign-oriented so that noninvoluted samples have higher PC1 scores than completely involuted samples; therefore, positive PC1 loadings correspond to genes contributing to the noninvoluted/proliferation-high direction. Loadings are unitless weights; larger absolute values indicate greater contribution to the corresponding PC.

| **PC1 rank (desc)** | **Gene** | **Variance rank (all genes)** | **PC1 loading** | **PC2 loading** |
| --- | --- | --- | --- | --- |
| 1 | NETO2 | 393 | 0.058744 | -0.021396 |
| 2 | EXTL1 | 301 | 0.057667 | 0.025674 |
| 3 | FHDC1 | 442 | 0.056557 | 0.058311 |
| 4 | ETV4 | 318 | 0.055808 | 0.049387 |
| 5 | PTHLH | 337 | 0.054833 | 0.020430 |
| 6 | DDAH1 | 415 | 0.052847 | 0.000544 |
| 7 | TCTEX1D2 | 313 | 0.052756 | -0.041091 |
| 8 | KRT18 | 160 | 0.052097 | 0.034342 |
| 9 | CRYBB3 | 366 | 0.052077 | 0.046323 |
| 10 | FMO6P | 216 | 0.051276 | -0.010404 |
| 11 | CHAF1A | 469 | 0.050867 | 0.025540 |
| 12 | ECEL1 | 82 | 0.050493 | 0.015424 |
| 13 | CYP46A1 | 458 | 0.050456 | -0.052814 |
| 14 | WNT10A | 449 | 0.050346 | 0.053311 |
| 15 | MLF1IP | 448 | 0.050181 | -0.003600 |
| 16 | TOP2A | 151 | 0.050053 | -0.042119 |
| 17 | AQP5 | 232 | 0.049450 | 0.050911 |
| 18 | SLC17A7 | 421 | 0.049276 | -0.067856 |
| 19 | GTF3C5 | 430 | 0.049052 | -0.014678 |
| 20 | RAMP1 | 382 | 0.048899 | 0.014843 |
| 21 | SOX12 | 466 | 0.048714 | 0.071848 |
| 22 | DCX | 222 | 0.048692 | 0.003980 |
| 23 | DPYSL4 | 404 | 0.048475 | 0.014834 |
| 24 | NRG3 | 308 | 0.048268 | -0.026003 |
| 25 | DIO2 | 202 | 0.048127 | 0.013877 |
| 26 | TYMS | 293 | 0.048027 | -0.031815 |
| 27 | FUT6 | 154 | 0.047943 | 0.080778 |
| 28 | UBE2C | 331 | 0.047745 | -0.043216 |
| 29 | FAM111B | 460 | 0.047636 | -0.039951 |
| 30 | RHOXF1 | 459 | 0.047543 | 0.062389 |
| 31 | PLS1 | 363 | 0.047284 | -0.071683 |
| 32 | HIST1H2BM | 445 | 0.047282 | -0.048896 |
| 33 | PNMT | 36 | 0.047264 | 0.042382 |
| 34 | ZP3 | 374 | 0.047146 | -0.009158 |
| 35 | SARDH | 355 | 0.047027 | -0.034714 |
| 36 | C11ORF82 | 264 | 0.046731 | -0.002207 |
| 37 | STRA6 | 282 | 0.046518 | 0.013579 |
| 38 | GTSE1 | 16 | 0.046478 | 0.038979 |
| 39 | LRRN1 | 273 | 0.045751 | -0.039127 |
| 40 | STS-1 | 367 | 0.045303 | -0.015788 |
| 41 | HIST1H2AJ | 205 | 0.045245 | -0.056884 |
| 42 | CXCL10 | 201 | 0.045239 | -0.063128 |
| 43 | KCNMB2 | 428 | 0.045047 | 0.063023 |
| 44 | HIST1H3G | 128 | 0.044886 | -0.058275 |
| 45 | LRRC4B | 324 | 0.044804 | -0.016472 |
| 46 | IGSF5 | 361 | 0.044762 | 0.019084 |
| 47 | CENPK | 272 | 0.044614 | -0.037379 |
| 48 | CDCA3 | 405 | 0.044235 | -0.028204 |
| 49 | SLC23A3 | 396 | 0.044210 | -0.007585 |
| 50 | ALPK2 | 270 | 0.044172 | -0.038109 |
| 51 | CASS4 | 409 | 0.044143 | 0.000755 |
| 52 | NCAPG | 228 | 0.044126 | -0.075111 |
| 53 | ZNF137 | 198 | 0.044050 | -0.067549 |
| 54 | WDR17 | 383 | 0.044018 | 0.060440 |
| 55 | SLC4A11 | 224 | 0.043815 | 0.044074 |
| 56 | DIAPH3 | 364 | 0.043797 | -0.078417 |
| 57 | CLSPN | 432 | 0.043786 | -0.004173 |
| 58 | LRRC37A4 | 178 | 0.043742 | 0.002981 |
| 59 | MLLT11 | 175 | 0.043728 | -0.063923 |
| 60 | APLN | 40 | 0.043712 | -0.051655 |
| 61 | HUNK | 474 | 0.043650 | 0.018610 |
| 62 | CDX1 | 79 | 0.043628 | 0.010280 |
| 63 | FAM109B | 420 | 0.043626 | -0.050582 |
| 64 | HIST1H1B | 200 | 0.043572 | -0.066214 |
| 65 | SHC4 | 357 | 0.043496 | -0.073560 |
| 66 | CXCL6 | 328 | 0.043447 | 0.020357 |
| 67 | SYT8 | 126 | 0.043276 | 0.019500 |
| 68 | GLB1L2 | 278 | 0.043186 | 0.056306 |
| 69 | CYP24A1 | 53 | 0.043164 | 0.077215 |
| 70 | OTOF | 65 | 0.043074 | -0.028714 |
| 71 | CXCL13 | 148 | 0.042857 | -0.045689 |
| 72 | COL4A6 | 149 | 0.042761 | 0.069302 |
| 73 | DNA2 | 497 | 0.042612 | 0.013820 |
| 74 | DLG4 | 267 | 0.042570 | 0.035488 |
| 75 | IKBKE | 164 | 0.042220 | -0.032188 |
| 76 | CORO7 | 213 | 0.042194 | -0.031244 |
| 77 | SCGB3A1 | 214 | 0.042160 | 0.068543 |
| 78 | GABRR1 | 196 | 0.042153 | -0.013350 |
| 79 | E2F2 | 495 | 0.041977 | -0.028178 |
| 80 | RLN2 | 484 | 0.041907 | -0.043291 |
| 81 | UFSP1 | 168 | 0.041853 | -0.035820 |
| 82 | UNQ830 | 444 | 0.041816 | 0.009740 |
| 83 | SLC34A2 | 378 | 0.041775 | 0.090311 |
| 84 | EDG7 | 86 | 0.041684 | -0.069747 |
| 85 | WNT4 | 142 | 0.041679 | 0.052842 |
| 86 | GPR125 | 118 | 0.041630 | -0.031262 |
| 87 | TESSP5 | 236 | 0.041629 | 0.068202 |
| 88 | GPR12 | 4 | 0.041542 | -0.057985 |
| 89 | SIRT6 | 42 | 0.041515 | 0.067617 |
| 90 | RIMS3 | 223 | 0.041463 | -0.010831 |
| 91 | CENPM | 219 | 0.041251 | -0.037250 |
| 92 | DDX23 | 235 | 0.041235 | 0.075600 |
| 93 | LRRC27 | 326 | 0.041228 | -0.035597 |
| 94 | ALDH3B1 | 170 | 0.040986 | 0.063301 |
| 95 | PTH2 | 140 | 0.040923 | -0.015124 |
| 96 | FAM83D | 67 | 0.040794 | -0.026396 |
| 97 | LGR6 | 315 | 0.040785 | 0.087934 |
| 98 | BTN2A3 | 165 | 0.040712 | -0.003944 |
| 99 | CNIH2 | 100 | 0.040623 | 0.049184 |
| 100 | PDE1C | 289 | 0.040570 | 0.065960 |
| 101 | CHGA | 138 | 0.040409 | -0.076639 |
| 102 | KIFC1 | 7 | 0.040248 | -0.050319 |
| 103 | GPR35 | 167 | 0.040180 | 0.091749 |
| 104 | PCDH11X | 162 | 0.040099 | 0.076333 |
| 105 | SGSM3 | 97 | 0.040008 | 0.015101 |
| 106 | CDCA8 | 143 | 0.040008 | -0.002577 |
| 107 | LOC255374 | 186 | 0.039804 | 0.098140 |
| 108 | C6ORF224 | 314 | 0.039653 | 0.030926 |
| 109 | C15ORF42 | 14 | 0.039491 | 0.081918 |
| 110 | HRASLS | 85 | 0.039227 | -0.001246 |
| 111 | NTRK1 | 456 | 0.039138 | -0.021359 |
| 112 | MND1 | 139 | 0.039081 | -0.062075 |
| 113 | GLT25D2 | 193 | 0.038976 | -0.009539 |
| 114 | ITIH5L | 124 | 0.038804 | -0.047876 |
| 115 | RPP30 | 169 | 0.038754 | 0.055194 |
| 116 | ARL9 | 116 | 0.038747 | 0.001470 |
| 117 | LRRC61 | 184 | 0.038722 | 0.106310 |
| 118 | LIPG | 31 | 0.038687 | 0.038521 |
| 119 | HS6ST2 | 172 | 0.038454 | -0.028575 |
| 120 | TFEB | 80 | 0.038406 | 0.063839 |
| 121 | HIST1H3B | 258 | 0.038380 | -0.011442 |
| 122 | PCTK3 | 180 | 0.038300 | 0.013194 |
| 123 | KNDC1 | 384 | 0.038116 | 0.071539 |
| 124 | CASKIN1 | 8 | 0.038081 | 0.032435 |
| 125 | FAM119A | 211 | 0.038070 | 0.004916 |
| 126 | ZNF425 | 381 | 0.037951 | -0.009888 |
| 127 | F12 | 257 | 0.037851 | 0.058840 |
| 128 | TP73 | 253 | 0.037846 | -0.037367 |
| 129 | KIF18B | 106 | 0.037749 | -0.101701 |
| 130 | NEK2 | 302 | 0.037689 | -0.051799 |
| 131 | C14ORF178 | 20 | 0.037527 | -0.081779 |
| 132 | KIF15 | 21 | 0.037373 | 0.019509 |
| 133 | HIST1H2BB | 347 | 0.037242 | -0.024965 |
| 134 | PAK3 | 221 | 0.037157 | -0.019944 |
| 135 | WNT2 | 243 | 0.036956 | -0.061205 |
| 136 | HMMR | 234 | 0.036812 | -0.042679 |
| 137 | FDX1L | 215 | 0.036593 | 0.011136 |
| 138 | GJB2 | 269 | 0.036434 | -0.060880 |
| 139 | ESPL1 | 123 | 0.036344 | -0.059471 |
| 140 | SH3BGR | 127 | 0.036302 | 0.099370 |
| 141 | SP6 | 481 | 0.036288 | 0.008514 |
| 142 | MGC20983 | 144 | 0.036219 | 0.097734 |
| 143 | TNFRSF17 | 266 | 0.036157 | -0.014499 |
| 144 | TM6SF2 | 354 | 0.036020 | -0.064708 |
| 145 | CACNG4 | 475 | 0.035971 | 0.064868 |
| 146 | TMEM171 | 286 | 0.035956 | -0.026574 |
| 147 | CASC5 | 28 | 0.035745 | -0.097391 |
| 148 | FLJ43806 | 114 | 0.035625 | 0.097179 |
| 149 | KIF20A | 10 | 0.035479 | -0.099903 |
| 150 | DISP2 | 51 | 0.035419 | -0.084100 |
| 151 | TFRC | 117 | 0.035230 | -0.027190 |
| 152 | WDR93 | 70 | 0.035177 | 0.053713 |
| 153 | C1ORF61 | 274 | 0.035131 | 0.060321 |
| 154 | POU4F1 | 54 | 0.034671 | 0.023325 |
| 155 | FAM64A | 130 | 0.034431 | -0.075490 |
| 156 | CPNE7 | 38 | 0.034415 | 0.086475 |
| 157 | CENPA | 300 | 0.034412 | -0.040352 |
| 158 | ZNF544 | 241 | 0.034363 | 0.070507 |
| 159 | CKAP2L | 3 | 0.034333 | -0.013996 |
| 160 | CST6 | 78 | 0.034054 | 0.047332 |
| 161 | MAG | 13 | 0.033859 | 0.047177 |
| 162 | CDH12 | 77 | 0.033828 | 0.000227 |
| 163 | LOC283551 | 163 | 0.033798 | -0.080093 |
| 164 | GALE | 410 | 0.033590 | 0.076923 |
| 165 | C2ORF48 | 242 | 0.033539 | -0.084813 |
| 166 | KCNK12 | 350 | 0.033493 | 0.010337 |
| 167 | TMEM121 | 9 | 0.033476 | -0.044176 |
| 168 | SERPINB13 | 156 | 0.033456 | -0.037332 |
| 169 | NPPC | 237 | 0.033445 | 0.011813 |
| 170 | C9ORF163 | 12 | 0.033086 | 0.095003 |
| 171 | CCDC65 | 390 | 0.033065 | 0.005444 |
| 172 | TESSP2 | 182 | 0.033052 | 0.088142 |
| 173 | ZNF114 | 76 | 0.032935 | 0.053743 |
| 174 | NT5M | 33 | 0.032750 | -0.079119 |
| 175 | FASLG | 385 | 0.032542 | -0.068857 |
| 176 | PSD | 332 | 0.032538 | -0.092759 |
| 177 | C6ORF174 | 191 | 0.032462 | 0.022034 |
| 178 | WFDC2 | 120 | 0.032453 | 0.109623 |
| 179 | MSH4 | 49 | 0.032328 | 0.055850 |
| 180 | FOXI1 | 72 | 0.032034 | 0.001897 |
| 181 | C9ORF140 | 60 | 0.031912 | 0.001339 |
| 182 | BRUNOL5 | 37 | 0.031862 | 0.116614 |
| 183 | LCT | 131 | 0.031842 | -0.054229 |
| 184 | FAM71E1 | 45 | 0.031634 | 0.120002 |
| 185 | CAMK2A | 34 | 0.031605 | -0.045265 |
| 186 | SH2D3A | 91 | 0.031548 | 0.022756 |
| 187 | NOX5 | 68 | 0.031454 | -0.089134 |
| 188 | ZMYND10 | 150 | 0.031453 | 0.000185 |
| 189 | SLC16A8 | 23 | 0.031402 | 0.082716 |
| 190 | ZSCAN4 | 93 | 0.031358 | 0.092356 |
| 191 | MPI | 488 | 0.031292 | 0.026781 |
| 192 | MAP4K1 | 252 | 0.031281 | 0.018876 |
| 193 | PRAM1 | 199 | 0.031273 | -0.087837 |
| 194 | BLK | 159 | 0.031115 | -0.001256 |
| 195 | LGALS7 | 429 | 0.031077 | 0.111246 |
| 196 | UBD | 362 | 0.031025 | -0.037145 |
| 197 | TRIP13 | 18 | 0.030880 | -0.026024 |
| 198 | FBXO15 | 50 | 0.030851 | 0.053044 |
| 199 | FAM83B | 155 | 0.030787 | 0.039879 |
| 200 | FAM62C | 47 | 0.030654 | -0.004533 |
| 201 | ASPHD2 | 46 | 0.030624 | -0.093869 |
| 202 | KCNQ5 | 105 | 0.030460 | 0.124312 |
| 203 | TTMA | 22 | 0.030170 | -0.079489 |
| 204 | KIF11 | 189 | 0.030011 | -0.062312 |
| 205 | KCNK5 | 147 | 0.029965 | 0.068870 |
| 206 | OVOL1 | 108 | 0.029831 | 0.095146 |
| 207 | AGBL4 | 71 | 0.029675 | 0.120516 |
| 208 | HSPC171 | 333 | 0.029211 | -0.056773 |
| 209 | HCG4 | 188 | 0.028774 | 0.041577 |
| 210 | RECQL4 | 203 | 0.028745 | 0.061907 |
| 211 | IGLL3 | 190 | 0.028637 | 0.104734 |
| 212 | RNF212 | 146 | 0.028603 | -0.091255 |
| 213 | CHI3L2 | 132 | 0.028603 | 0.076349 |
| 214 | C13ORF3 | 399 | 0.028593 | -0.038332 |
| 215 | SLC2A9 | 110 | 0.028565 | -0.041525 |
| 216 | UNC5D | 87 | 0.028471 | -0.072575 |
| 217 | COX6B2 | 335 | 0.028466 | 0.052845 |
| 218 | CRLF1 | 92 | 0.028395 | 0.098954 |
| 219 | MGC70870 | 88 | 0.028340 | -0.061826 |
| 220 | TMTC2 | 288 | 0.028204 | -0.078607 |
| 221 | SLC36A2 | 11 | 0.028040 | 0.009038 |
| 222 | UGT2B15 | 107 | 0.027928 | 0.002332 |
| 223 | PAX2 | 166 | 0.027864 | 0.026527 |
| 224 | MSTN | 57 | 0.027828 | 0.065500 |
| 225 | ITIH2 | 29 | 0.027677 | -0.017372 |
| 226 | C4ORF45 | 161 | 0.027559 | -0.057941 |
| 227 | RASAL1 | 90 | 0.027535 | 0.057693 |
| 228 | CCDC148 | 35 | 0.027361 | -0.063023 |
| 229 | CHAC2 | 52 | 0.026861 | -0.047020 |
| 230 | CHST4 | 102 | 0.026762 | -0.043478 |
| 231 | TNFRSF8 | 62 | 0.026116 | -0.009327 |
| 232 | TOE1 | 306 | 0.026044 | 0.071397 |
| 233 | C1ORF168 | 69 | 0.025959 | -0.035529 |
| 234 | C19ORF4 | 74 | 0.025929 | 0.009386 |
| 235 | IFNE1 | 32 | 0.025866 | 0.032736 |
| 236 | ZNF385C | 30 | 0.024332 | 0.027816 |
| 237 | SH2D2A | 422 | 0.024130 | -0.033158 |
| 238 | CDH4 | 179 | 0.023651 | 0.054451 |
| 239 | FKSG30 | 17 | 0.023535 | 0.065682 |
| 240 | ICOS | 339 | 0.023233 | -0.040641 |
| 241 | SAPS2 | 75 | 0.023014 | 0.034639 |
| 242 | ENPP6 | 24 | 0.022168 | 0.058058 |
| 243 | POU5F1P1 | 41 | 0.022057 | 0.056117 |
| 244 | OR52K1 | 83 | 0.021797 | 0.036610 |
| 245 | CDKL3 | 125 | 0.020740 | 0.066657 |
| 246 | SLC12A3 | 99 | 0.020261 | 0.046297 |
| 247 | GPR172B | 6 | 0.018837 | -0.043538 |
| 248 | PNLDC1 | 152 | 0.018578 | 0.061125 |
| 249 | PVRIG | 351 | 0.018211 | 0.007525 |
| 250 | TSSK3 | 280 | 0.018159 | -0.015636 |
| 251 | HLA-DQA2 | 195 | 0.017355 | 0.004915 |
| 252 | TACR2 | 56 | 0.016154 | -0.045030 |
| 253 | SEMA7A | 55 | 0.015092 | -0.048786 |
| 254 | LALBA | 1 | 0.013062 | -0.003791 |
| 255 | LOXL2 | 250 | -0.020692 | 0.100404 |
| 256 | TMEM63C | 303 | -0.026220 | 0.018052 |
| 257 | LSAMP | 465 | -0.026753 | -0.048291 |
| 258 | MGC35361 | 482 | -0.026982 | 0.077057 |
| 259 | C16ORF3 | 419 | -0.027566 | 0.046747 |
| 260 | REEP3 | 344 | -0.028958 | -0.038666 |
| 261 | TPO | 454 | -0.029050 | 0.063294 |
| 262 | XRN2 | 26 | -0.029550 | -0.021277 |
| 263 | BBS2 | 496 | -0.029967 | -0.053245 |
| 264 | UGT2B28 | 19 | -0.031433 | -0.007607 |
| 265 | UBAP2 | 292 | -0.031628 | 0.039753 |
| 266 | PNLIPRP3 | 58 | -0.032186 | -0.001734 |
| 267 | DHRS2 | 61 | -0.032279 | -0.004933 |
| 268 | DRD4 | 477 | -0.032309 | 0.067530 |
| 269 | SELPLG | 407 | -0.032947 | -0.050583 |
| 270 | PLEK | 499 | -0.032958 | -0.040869 |
| 271 | C22ORF34 | 220 | -0.033427 | -0.035777 |
| 272 | C6ORF27 | 279 | -0.033529 | 0.082394 |
| 273 | UGT2B11 | 73 | -0.033587 | -0.011649 |
| 274 | FAM36A | 500 | -0.033659 | -0.025843 |
| 275 | C12ORF60 | 376 | -0.033846 | -0.067116 |
| 276 | PPARGC1A | 276 | -0.034766 | -0.031235 |
| 277 | CDO1 | 281 | -0.035173 | -0.007938 |
| 278 | IFITM4P | 476 | -0.035199 | 0.103695 |
| 279 | KLK4 | 194 | -0.035598 | -0.035999 |
| 280 | CD207 | 239 | -0.035688 | 0.009804 |
| 281 | PAPSS2 | 397 | -0.036132 | -0.073179 |
| 282 | LHCGR | 185 | -0.036270 | -0.020366 |
| 283 | CYP4Z1 | 227 | -0.036790 | 0.001335 |
| 284 | KYNU | 349 | -0.036891 | -0.012142 |
| 285 | MYOC | 5 | -0.037195 | -0.008089 |
| 286 | ECM2 | 295 | -0.037498 | -0.024828 |
| 287 | PLAG1 | 297 | -0.037550 | 0.020539 |
| 288 | ZNF687 | 462 | -0.038409 | -0.052903 |
| 289 | SLC8A2 | 197 | -0.038457 | 0.052612 |
| 290 | MAN1C1 | 360 | -0.038572 | 0.006826 |
| 291 | NCK1 | 472 | -0.038575 | -0.039579 |
| 292 | C1ORF115 | 468 | -0.038903 | -0.051920 |
| 293 | TKTL1 | 485 | -0.039146 | -0.047892 |
| 294 | DKK2 | 309 | -0.039159 | 0.016497 |
| 295 | PTPN11 | 359 | -0.039382 | -0.032882 |
| 296 | ZNF284 | 290 | -0.039499 | -0.005224 |
| 297 | CLDN6 | 39 | -0.039645 | 0.015863 |
| 298 | TMEM104 | 327 | -0.039792 | 0.003232 |
| 299 | SILV | 229 | -0.040692 | 0.003404 |
| 300 | TNIK | 447 | -0.040936 | -0.026214 |
| 301 | HOXB2 | 322 | -0.040968 | -0.018155 |
| 302 | HLA-F | 412 | -0.041150 | 0.078123 |
| 303 | SPTBN4 | 231 | -0.041331 | 0.043913 |
| 304 | LOC340527 | 368 | -0.041363 | -0.045514 |
| 305 | ANKRD29 | 251 | -0.041466 | -0.020804 |
| 306 | C4ORF31 | 136 | -0.041670 | -0.013310 |
| 307 | KIF13A | 411 | -0.041730 | -0.007204 |
| 308 | KIAA0367 | 483 | -0.042366 | -0.015867 |
| 309 | NNAT | 249 | -0.042562 | -0.025040 |
| 310 | RPA4 | 437 | -0.042833 | 0.000041 |
| 311 | PYY | 271 | -0.042857 | 0.077584 |
| 312 | KIAA1826 | 453 | -0.043112 | -0.004262 |
| 313 | RIMS1 | 218 | -0.043168 | 0.079750 |
| 314 | SOX30 | 268 | -0.043230 | 0.059407 |
| 315 | LRRC8B | 451 | -0.043316 | -0.035899 |
| 316 | ABLIM2 | 379 | -0.043957 | -0.052862 |
| 317 | DOC2B | 423 | -0.044124 | 0.041785 |
| 318 | ACR | 371 | -0.044132 | 0.050466 |
| 319 | N4BP3 | 287 | -0.044405 | 0.055957 |
| 320 | GIPC2 | 494 | -0.044439 | -0.065520 |
| 321 | PTGIR | 353 | -0.044455 | 0.034770 |
| 322 | CACNB2 | 408 | -0.044621 | 0.000406 |
| 323 | LRRN4CL | 443 | -0.044706 | -0.002162 |
| 324 | PCDHGB7 | 208 | -0.044934 | 0.002007 |
| 325 | PTPN7 | 238 | -0.045540 | -0.011032 |
| 326 | LEPR | 455 | -0.045629 | -0.022719 |
| 327 | DAO | 311 | -0.045693 | 0.083211 |
| 328 | MYCT1 | 372 | -0.045825 | -0.000091 |
| 329 | CA4 | 256 | -0.045844 | 0.006017 |
| 330 | RASL10B | 320 | -0.045966 | 0.022936 |
| 331 | ARL8B | 440 | -0.046287 | -0.031397 |
| 332 | TNMD | 342 | -0.046469 | 0.030406 |
| 333 | LIPE | 25 | -0.046812 | 0.014756 |
| 334 | C1QTNF7 | 204 | -0.047519 | -0.011807 |
| 335 | CYP2C19 | 248 | -0.047613 | 0.014545 |
| 336 | CORO2B | 461 | -0.047702 | 0.044229 |
| 337 | C4ORF36 | 492 | -0.047762 | 0.032876 |
| 338 | COL12A1 | 403 | -0.047799 | -0.018522 |
| 339 | PCDHB2 | 121 | -0.048063 | -0.024665 |
| 340 | PGM5 | 392 | -0.048121 | -0.031761 |
| 341 | SIX1 | 305 | -0.048167 | 0.000804 |
| 342 | CLCN4 | 463 | -0.048187 | -0.039313 |
| 343 | IL6R | 291 | -0.048413 | 0.028295 |
| 344 | LAMC1 | 402 | -0.048423 | -0.018169 |
| 345 | THRSP | 312 | -0.048450 | -0.003032 |
| 346 | NOS3 | 439 | -0.048472 | -0.035315 |
| 347 | ID1 | 401 | -0.048532 | 0.053262 |
| 348 | SVEP1 | 416 | -0.048802 | -0.022043 |
| 349 | C21ORF7 | 391 | -0.048897 | -0.033146 |
| 350 | SLC22A14 | 135 | -0.049087 | 0.050664 |
| 351 | ASPRV1 | 112 | -0.049290 | -0.027726 |
| 352 | FLJ32011 | 48 | -0.049480 | 0.051340 |
| 353 | MCF2L | 157 | -0.049654 | 0.035133 |
| 354 | OR4F21 | 115 | -0.049755 | 0.032730 |
| 355 | C6 | 109 | -0.049854 | -0.017486 |
| 356 | PRDM16 | 304 | -0.049887 | 0.037326 |
| 357 | TRIM35 | 433 | -0.049951 | -0.007680 |
| 358 | PPP1R16B | 486 | -0.050011 | -0.032546 |
| 359 | CYP4X1 | 255 | -0.050073 | 0.015549 |
| 360 | SLC4A4 | 119 | -0.050080 | -0.010434 |
| 361 | THEM2 | 345 | -0.050092 | -0.019198 |
| 362 | LOC650695 | 84 | -0.050162 | -0.001756 |
| 363 | NKD1 | 434 | -0.050279 | -0.012333 |
| 364 | PCDHGA4 | 310 | -0.050486 | 0.047720 |
| 365 | VEGFC | 261 | -0.050618 | 0.011194 |
| 366 | NXNL1 | 373 | -0.050742 | 0.039129 |
| 367 | EPB41L5 | 457 | -0.050875 | 0.021112 |
| 368 | SLC25A20 | 441 | -0.051028 | -0.003636 |
| 369 | CCDC57 | 104 | -0.051066 | 0.040597 |
| 370 | SHISA2 | 277 | -0.051181 | -0.048849 |
| 371 | GABRE | 467 | -0.051238 | 0.033385 |
| 372 | TUSC5 | 66 | -0.051264 | 0.017896 |
| 373 | C2ORF32 | 210 | -0.051373 | -0.033969 |
| 374 | GRRP1 | 348 | -0.051423 | 0.034156 |
| 375 | CNTLN | 192 | -0.051504 | 0.052121 |
| 376 | C1ORF51 | 329 | -0.051513 | -0.035747 |
| 377 | CALCRL | 369 | -0.051568 | 0.018581 |
| 378 | STC1 | 260 | -0.051708 | 0.020432 |
| 379 | ACADL | 129 | -0.051770 | -0.003062 |
| 380 | HOXB7 | 207 | -0.051807 | -0.014939 |
| 381 | SCNN1B | 413 | -0.052212 | 0.041409 |
| 382 | NOTCH4 | 259 | -0.052244 | 0.056165 |
| 383 | PRELP | 479 | -0.052296 | 0.010623 |
| 384 | PRSS16 | 336 | -0.052353 | 0.010753 |
| 385 | IL3RA | 387 | -0.052391 | -0.020642 |
| 386 | IGFL3 | 245 | -0.052446 | 0.026188 |
| 387 | ELP4 | 343 | -0.052461 | -0.023416 |
| 388 | NUAK1 | 487 | -0.052612 | -0.037191 |
| 389 | CDKN2B | 101 | -0.052731 | 0.013833 |
| 390 | STK32A | 338 | -0.052824 | 0.006678 |
| 391 | STOX1 | 431 | -0.052938 | -0.018427 |
| 392 | EPHX1 | 298 | -0.052946 | 0.018722 |
| 393 | SORCS1 | 389 | -0.053025 | 0.004721 |
| 394 | WNT11 | 63 | -0.053066 | 0.014768 |
| 395 | FLJ90650 | 133 | -0.053136 | 0.004523 |
| 396 | DUSP15 | 283 | -0.053173 | 0.004990 |
| 397 | LEFTY2 | 285 | -0.053380 | 0.027951 |
| 398 | GPR146 | 317 | -0.053530 | -0.018838 |
| 399 | MCTP1 | 425 | -0.053588 | -0.033773 |
| 400 | PLA2G4F | 299 | -0.053592 | 0.018062 |
| 401 | GLYAT | 95 | -0.053748 | 0.003659 |
| 402 | LOC284009 | 446 | -0.053884 | 0.014443 |
| 403 | PMEPA1 | 375 | -0.053900 | -0.026155 |
| 404 | SNF1LK2 | 319 | -0.053906 | -0.015315 |
| 405 | HOXA13 | 225 | -0.053910 | 0.020881 |
| 406 | STX7 | 493 | -0.053926 | -0.021624 |
| 407 | CCDC85A | 247 | -0.054119 | -0.007282 |
| 408 | RANBP10 | 177 | -0.054148 | 0.031129 |
| 409 | RLTPR | 307 | -0.054221 | -0.034650 |
| 410 | GPC6 | 294 | -0.054275 | -0.023041 |
| 411 | ADA | 275 | -0.054483 | -0.033969 |
| 412 | ST6GALNAC3 | 436 | -0.054543 | -0.007579 |
| 413 | GPR4 | 226 | -0.054613 | 0.034218 |
| 414 | LTBR | 491 | -0.054624 | 0.020488 |
| 415 | EVI1 | 473 | -0.054628 | 0.010886 |
| 416 | AVPR1A | 435 | -0.054644 | -0.009827 |
| 417 | FAM80B | 358 | -0.054699 | -0.028241 |
| 418 | CALB2 | 187 | -0.054739 | 0.008582 |
| 419 | C6ORF107 | 183 | -0.054886 | 0.007121 |
| 420 | KCNAB1 | 406 | -0.054924 | 0.016393 |
| 421 | GPD1 | 89 | -0.054968 | 0.004958 |
| 422 | BMP5 | 246 | -0.055053 | 0.002027 |
| 423 | GPR20 | 230 | -0.055055 | 0.005100 |
| 424 | NMB | 59 | -0.055282 | -0.000522 |
| 425 | GGNBP2 | 134 | -0.055349 | 0.031467 |
| 426 | MTHFSD | 424 | -0.055454 | 0.000313 |
| 427 | ENPP4 | 478 | -0.055852 | 0.006513 |
| 428 | CXCR7 | 346 | -0.055865 | -0.010556 |
| 429 | DMRT3 | 174 | -0.055962 | 0.004395 |
| 430 | ANGPT1 | 489 | -0.055975 | 0.051611 |
| 431 | HOXB8 | 262 | -0.056093 | -0.022212 |
| 432 | IL8 | 141 | -0.056113 | 0.023516 |
| 433 | HSD11B1 | 206 | -0.056129 | 0.000586 |
| 434 | ITK | 471 | -0.056178 | -0.004009 |
| 435 | ANKRD43 | 296 | -0.056190 | 0.001073 |
| 436 | KCNB1 | 145 | -0.056224 | 0.020185 |
| 437 | CDH8 | 44 | -0.056287 | 0.005044 |
| 438 | A2BP1 | 64 | -0.056301 | 0.021926 |
| 439 | TTPA | 452 | -0.056349 | 0.005720 |
| 440 | ETFA | 321 | -0.056619 | -0.028214 |
| 441 | ADH1B | 417 | -0.056945 | -0.002148 |
| 442 | FHL5 | 217 | -0.056983 | -0.000920 |
| 443 | MMD | 122 | -0.056996 | 0.005448 |
| 444 | MRAP | 173 | -0.057001 | 0.012501 |
| 445 | ITGA7 | 356 | -0.057019 | 0.014663 |
| 446 | RORB | 103 | -0.057254 | 0.000448 |
| 447 | LGR5 | 212 | -0.057543 | 0.010947 |
| 448 | ADRA1A | 81 | -0.057593 | 0.006195 |
| 449 | PLIN | 113 | -0.057617 | 0.010226 |
| 450 | AGT | 96 | -0.057684 | -0.005299 |
| 451 | PRKAR2B | 233 | -0.057688 | 0.005393 |
| 452 | FLJ20674 | 330 | -0.057690 | 0.004817 |
| 453 | FAM21A | 153 | -0.057702 | -0.017268 |
| 454 | GALNTL2 | 480 | -0.057786 | -0.002205 |
| 455 | IGF2BP3 | 209 | -0.057872 | -0.013845 |
| 456 | ART4 | 171 | -0.057913 | -0.000046 |
| 457 | F3 | 418 | -0.057963 | -0.001585 |
| 458 | APOLD1 | 323 | -0.058123 | -0.015025 |
| 459 | HSPB7 | 176 | -0.058143 | -0.005522 |
| 460 | TMEM22 | 394 | -0.058154 | 0.003951 |
| 461 | FAM107A | 470 | -0.058216 | 0.014316 |
| 462 | PKD1L2 | 137 | -0.058225 | 0.021393 |
| 463 | C11ORF67 | 284 | -0.058229 | -0.033548 |
| 464 | SLC10A6 | 352 | -0.058591 | 0.005356 |
| 465 | ADIPOQ | 265 | -0.058643 | 0.000836 |
| 466 | PCDHGA11 | 398 | -0.058756 | -0.012220 |
| 467 | ATP1A2 | 340 | -0.058833 | 0.009411 |
| 468 | ASB9 | 377 | -0.058858 | -0.023145 |
| 469 | SLC7A10 | 98 | -0.058896 | 0.006740 |
| 470 | PDGFB | 386 | -0.059407 | 0.013848 |
| 471 | NAV3 | 464 | -0.059464 | 0.011564 |
| 472 | CES1 | 240 | -0.059480 | 0.005798 |
| 473 | C3ORF42 | 426 | -0.059809 | -0.017033 |
| 474 | PREX2 | 365 | -0.060010 | -0.015837 |
| 475 | LOC440925 | 94 | -0.060058 | 0.003814 |
| 476 | TPST2 | 370 | -0.060278 | 0.017918 |
| 477 | RAPGEF3 | 388 | -0.060527 | 0.015600 |
| 478 | SV2B | 325 | -0.060623 | -0.000744 |
| 479 | GPAM | 158 | -0.060672 | -0.003058 |
| 480 | GLDN | 111 | -0.060932 | -0.011200 |
| 481 | TYRO3P | 316 | -0.060960 | -0.004924 |
| 482 | RPS6KA2 | 490 | -0.061011 | -0.011409 |
| 483 | MEOX1 | 380 | -0.061177 | -0.008981 |
| 484 | CARD8 | 427 | -0.061491 | 0.003309 |
| 485 | RPL23AP13 | 395 | -0.061523 | 0.007824 |
| 486 | STX11 | 263 | -0.061754 | 0.002990 |
| 487 | AGTR1 | 450 | -0.061887 | -0.013647 |
| 488 | SLCO4A1 | 254 | -0.062072 | 0.017246 |
| 489 | SYCP2L | 334 | -0.062080 | 0.036559 |
| 490 | PCOLCE2 | 43 | -0.062583 | 0.001672 |
| 491 | NMUR1 | 244 | -0.062652 | 0.009686 |
| 492 | CD93 | 400 | -0.062683 | -0.012293 |
| 493 | SAA1 | 414 | -0.062949 | 0.029826 |
| 494 | SNTG2 | 15 | -0.062977 | 0.000280 |
| 495 | HRASLS3 | 438 | -0.063273 | -0.009199 |
| 496 | MPN2 | 27 | -0.064229 | 0.002383 |
| 497 | PTPRB | 498 | -0.064741 | -0.003908 |
| 498 | CES4 | 341 | -0.064828 | -0.018568 |
| 499 | ADH1A | 2 | -0.065802 | 0.002001 |
| 500 | AFAP1L1 | 181 | -0.065961 | 0.011616 |

**Table S3. Genome-wide differential expression between noninvoluted and completely involuted postmenopausal breast tissue**

Differential expression between the postmenopausal endpoint-cohort extremes (8 noninvoluted vs 4 completely involuted benign biopsies) was assessed for each gene on the genome-wide Illumina DASL expression array. Per-gene comparisons used Welch's two-sample t-test on normalised log2 expression, with Benjamini–Hochberg (BH) FDR control across the 1,414 tested genes; genes are ranked by FDR. log2 fold-change is expressed as noninvoluted minus completely involuted, so positive values indicate higher expression in noninvoluted tissue. Significant genes (FDR < 0.05): 373 higher and 204 lower in noninvoluted tissue. At the less stringent nominal P < 0.05: 550 higher and 439 lower; the nominal-P<0.05, positive-fold-change set (550 genes) is the “noninvoluted-high” signature used for the tumour-context enrichment in Figure 1E.

**Column definitions**

**Gene:** Gene symbol (DASL probe).

**Mean expression (noninvoluted):** Group mean normalised log2 expression, noninvoluted.

**Mean expression (completely involuted):** Group mean normalised log2 expression, completely involuted.

**log2 fold-change:** noninvoluted − completely involuted (positive = higher in noninvoluted).

**P value:** Welch's two-sample t-test, per gene.

**FDR (BH):** Benjamini–Hochberg false discovery rate across 1,414 genes.

**Direction:** Up/Down in noninvoluted at FDR < 0.05; otherwise Not significant.

| **Gene** | **Mean expr. (noninvoluted)** | **Mean expr. (compl. involuted)** | **log2 FC (NI − CI)** | **P value** | **FDR (BH)** | **Direction** |
| --- | --- | --- | --- | --- | --- | --- |
| **CES1** | 0.752 | 3.162 | -2.072 | 2.34E-07 | 3.30E-04 | Down in Noninvoluted |
| **BYSL** | 1.145 | 0.127 | 3.176 | 1.75E-06 | 1.24E-03 | Up in Noninvoluted |
| **SAA1** | 0.693 | 2.193 | -1.663 | 1.52E-05 | 6.26E-03 | Down in Noninvoluted |
| **NPR2** | 0.786 | 1.721 | -1.130 | 1.82E-05 | 6.26E-03 | Down in Noninvoluted |
| **RCAN2** | 0.918 | 1.801 | -0.972 | 2.22E-05 | 6.26E-03 | Down in Noninvoluted |
| **MICALCL** | 1.236 | 0.147 | 3.070 | 4.66E-05 | 7.50E-03 | Up in Noninvoluted |
| **BCL11A** | 1.154 | 0.634 | 0.865 | 4.59E-05 | 7.50E-03 | Up in Noninvoluted |
| **PTPN12** | 0.855 | 1.453 | -0.766 | 4.71E-05 | 7.50E-03 | Down in Noninvoluted |
| **ADAM12** | 1.217 | 0.458 | 1.409 | 5.53E-05 | 7.50E-03 | Up in Noninvoluted |
| **DCP2** | 1.136 | 0.610 | 0.897 | 5.83E-05 | 7.50E-03 | Up in Noninvoluted |
| **ZNF781** | 1.015 | 0.155 | 2.712 | 5.82E-05 | 7.50E-03 | Up in Noninvoluted |
| **KDELC1** | 1.092 | 0.453 | 1.268 | 7.92E-05 | 8.61E-03 | Up in Noninvoluted |
| **C3ORF59** | 1.192 | 0.606 | 0.975 | 7.78E-05 | 8.61E-03 | Up in Noninvoluted |
| **SLC6A14** | 1.414 | 0.246 | 2.526 | 1.19E-04 | 1.20E-02 | Up in Noninvoluted |
| **ALX4** | 1.335 | 0.204 | 2.707 | 1.95E-04 | 1.48E-02 | Up in Noninvoluted |
| **ABHD6** | 0.832 | 1.690 | -1.022 | 2.12E-04 | 1.48E-02 | Down in Noninvoluted |
| **WNK2** | 1.133 | 0.384 | 1.561 | 1.96E-04 | 1.48E-02 | Up in Noninvoluted |
| **PUSL1** | 1.194 | 0.321 | 1.898 | 2.05E-04 | 1.48E-02 | Up in Noninvoluted |
| **CBLL1** | 1.100 | 0.764 | 0.526 | 2.16E-04 | 1.48E-02 | Up in Noninvoluted |
| **RPL13** | 1.213 | 0.662 | 0.873 | 1.90E-04 | 1.48E-02 | Up in Noninvoluted |
| **XKR6** | 1.157 | 0.555 | 1.059 | 2.20E-04 | 1.48E-02 | Up in Noninvoluted |
| **TRAF4** | 1.142 | 0.537 | 1.088 | 2.46E-04 | 1.54E-02 | Up in Noninvoluted |
| **YPEL2** | 0.875 | 1.865 | -1.091 | 2.51E-04 | 1.54E-02 | Down in Noninvoluted |
| **C8ORF47** | 1.225 | 0.501 | 1.290 | 3.04E-04 | 1.72E-02 | Up in Noninvoluted |
| **SLC38A5** | 1.230 | 0.555 | 1.147 | 3.01E-04 | 1.72E-02 | Up in Noninvoluted |
| **NETO2** | 1.555 | 0.132 | 3.556 | 3.23E-04 | 1.76E-02 | Up in Noninvoluted |
| **DPM1** | 0.837 | 1.544 | -0.883 | 3.52E-04 | 1.84E-02 | Down in Noninvoluted |
| **HOOK3** | 0.883 | 1.422 | -0.688 | 4.01E-04 | 2.03E-02 | Down in Noninvoluted |
| **PIGR** | 1.296 | 0.301 | 2.105 | 8.61E-04 | 2.06E-02 | Up in Noninvoluted |
| **DLK1** | 1.177 | 0.230 | 2.358 | 5.48E-04 | 2.06E-02 | Up in Noninvoluted |
| **S1PR2** | 1.209 | 0.171 | 2.819 | 8.82E-04 | 2.06E-02 | Up in Noninvoluted |
| **ITGA3** | 1.227 | 0.414 | 1.566 | 6.34E-04 | 2.06E-02 | Up in Noninvoluted |
| **C21ORF58** | 1.145 | 0.207 | 2.466 | 4.66E-04 | 2.06E-02 | Up in Noninvoluted |
| **CHAF1A** | 1.389 | 0.188 | 2.884 | 5.15E-04 | 2.06E-02 | Up in Noninvoluted |
| **DKFZP564O0823** | 0.624 | 1.418 | -1.185 | 6.79E-04 | 2.06E-02 | Down in Noninvoluted |
| **GEN1** | 1.205 | 0.381 | 1.662 | 7.03E-04 | 2.06E-02 | Up in Noninvoluted |
| **PVRL1** | 1.311 | 0.454 | 1.531 | 7.46E-04 | 2.06E-02 | Up in Noninvoluted |
| **GNAL** | 0.747 | 1.511 | -1.016 | 8.73E-04 | 2.06E-02 | Down in Noninvoluted |
| **USP31** | 1.151 | 0.568 | 1.020 | 5.37E-04 | 2.06E-02 | Up in Noninvoluted |
| **ITGA11** | 1.099 | 0.606 | 0.859 | 8.70E-04 | 2.06E-02 | Up in Noninvoluted |
| **SHISA2** | 0.730 | 2.565 | -1.812 | 4.29E-04 | 2.06E-02 | Down in Noninvoluted |
| **ZNF274** | 0.856 | 1.278 | -0.579 | 7.25E-04 | 2.06E-02 | Down in Noninvoluted |
| **IL17RE** | 1.395 | 0.384 | 1.863 | 6.99E-04 | 2.06E-02 | Up in Noninvoluted |
| **C2ORF18** | 0.873 | 1.311 | -0.586 | 8.33E-04 | 2.06E-02 | Down in Noninvoluted |
| **RNF38** | 0.825 | 1.428 | -0.791 | 6.31E-04 | 2.06E-02 | Down in Noninvoluted |
| **ZNF322A** | 0.820 | 1.207 | -0.557 | 4.61E-04 | 2.06E-02 | Down in Noninvoluted |
| **MRPS12** | 1.238 | 0.735 | 0.753 | 8.54E-04 | 2.06E-02 | Up in Noninvoluted |
| **PHLDA1** | 1.263 | 0.675 | 0.904 | 6.39E-04 | 2.06E-02 | Up in Noninvoluted |
| **TTYH1** | 1.355 | 0.435 | 1.639 | 5.14E-04 | 2.06E-02 | Up in Noninvoluted |
| **GPC1** | 1.225 | 0.508 | 1.269 | 4.44E-04 | 2.06E-02 | Up in Noninvoluted |
| **ADI1** | 0.962 | 1.804 | -0.907 | 6.72E-04 | 2.06E-02 | Down in Noninvoluted |
| **IREB2** | 0.967 | 1.320 | -0.449 | 7.39E-04 | 2.06E-02 | Down in Noninvoluted |
| **PCBP4** | 1.098 | 0.810 | 0.439 | 8.54E-04 | 2.06E-02 | Up in Noninvoluted |
| **UGT8** | 1.181 | 0.580 | 1.025 | 8.25E-04 | 2.06E-02 | Up in Noninvoluted |
| **TAS2R43** | 1.078 | 0.197 | 2.452 | 7.09E-04 | 2.06E-02 | Up in Noninvoluted |
| **LOC284009** | 0.818 | 2.177 | -1.412 | 6.52E-04 | 2.06E-02 | Down in Noninvoluted |
| **HIST1H2AD** | 1.239 | 0.569 | 1.124 | 6.63E-04 | 2.06E-02 | Up in Noninvoluted |
| **PACRG** | 1.263 | 0.414 | 1.611 | 6.35E-04 | 2.06E-02 | Up in Noninvoluted |
| **RMI1** | 1.178 | 0.212 | 2.472 | 8.18E-04 | 2.06E-02 | Up in Noninvoluted |
| **STX11** | 0.773 | 2.964 | -1.939 | 8.88E-04 | 2.06E-02 | Down in Noninvoluted |
| **ZNF513** | 1.374 | 0.374 | 1.878 | 5.95E-04 | 2.06E-02 | Up in Noninvoluted |
| **ANKRD10** | 0.786 | 1.475 | -0.907 | 9.24E-04 | 2.11E-02 | Down in Noninvoluted |
| **NARS2** | 1.117 | 0.869 | 0.362 | 9.67E-04 | 2.14E-02 | Up in Noninvoluted |
| **WNT10A** | 1.317 | 0.128 | 3.365 | 9.55E-04 | 2.14E-02 | Up in Noninvoluted |
| **MAP2K1** | 0.714 | 1.392 | -0.963 | 1.15E-03 | 2.16E-02 | Down in Noninvoluted |
| **SPATA13** | 1.176 | 0.641 | 0.876 | 1.14E-03 | 2.16E-02 | Up in Noninvoluted |
| **SH3PXD2B** | 1.190 | 0.617 | 0.948 | 1.01E-03 | 2.16E-02 | Up in Noninvoluted |
| **TXNDC14** | 0.746 | 1.402 | -0.911 | 1.14E-03 | 2.16E-02 | Down in Noninvoluted |
| **SPC25** | 0.804 | 1.184 | -0.557 | 1.14E-03 | 2.16E-02 | Down in Noninvoluted |
| **FLJ20674** | 0.697 | 2.492 | -1.839 | 1.13E-03 | 2.16E-02 | Down in Noninvoluted |
| **PTPRK** | 1.096 | 0.793 | 0.467 | 1.13E-03 | 2.16E-02 | Up in Noninvoluted |
| **ST14** | 1.202 | 0.575 | 1.064 | 1.14E-03 | 2.16E-02 | Up in Noninvoluted |
| **CMAH** | 0.943 | 1.177 | -0.320 | 1.04E-03 | 2.16E-02 | Down in Noninvoluted |
| **DAG1** | 1.278 | 0.653 | 0.970 | 1.10E-03 | 2.16E-02 | Up in Noninvoluted |
| **RAMP1** | 1.442 | 0.078 | 4.205 | 1.12E-03 | 2.16E-02 | Up in Noninvoluted |
| **RAB7B** | 1.303 | 0.298 | 2.127 | 1.17E-03 | 2.17E-02 | Up in Noninvoluted |
| **PDK3** | 1.138 | 0.492 | 1.209 | 1.20E-03 | 2.20E-02 | Up in Noninvoluted |
| **ISLR** | 1.111 | 0.609 | 0.867 | 1.22E-03 | 2.22E-02 | Up in Noninvoluted |
| **ELL3** | 1.222 | 0.420 | 1.540 | 1.25E-03 | 2.24E-02 | Up in Noninvoluted |
| **EXTL1** | 1.743 | 0.103 | 4.074 | 1.32E-03 | 2.34E-02 | Up in Noninvoluted |
| **TNXA** | 0.729 | 1.468 | -1.010 | 1.40E-03 | 2.34E-02 | Down in Noninvoluted |
| **DUSP22** | 0.801 | 1.357 | -0.760 | 1.38E-03 | 2.34E-02 | Down in Noninvoluted |
| **C1ORF54** | 0.739 | 1.542 | -1.062 | 1.41E-03 | 2.34E-02 | Down in Noninvoluted |
| **AGR3** | 1.354 | 0.399 | 1.764 | 1.35E-03 | 2.34E-02 | Up in Noninvoluted |
| **NCOR2** | 1.087 | 0.887 | 0.293 | 1.37E-03 | 2.34E-02 | Up in Noninvoluted |
| **GIPC2** | 0.672 | 1.759 | -1.389 | 1.44E-03 | 2.36E-02 | Down in Noninvoluted |
| **G6PD** | 0.829 | 1.322 | -0.672 | 1.45E-03 | 2.36E-02 | Down in Noninvoluted |
| **IFNGR1** | 0.886 | 1.643 | -0.891 | 1.47E-03 | 2.36E-02 | Down in Noninvoluted |
| **MAP1S** | 1.172 | 0.609 | 0.945 | 1.66E-03 | 2.39E-02 | Up in Noninvoluted |
| **ZNF133** | 1.175 | 0.557 | 1.077 | 1.61E-03 | 2.39E-02 | Up in Noninvoluted |
| **AGRN** | 1.081 | 0.338 | 1.678 | 1.58E-03 | 2.39E-02 | Up in Noninvoluted |
| **40791** | 1.145 | 0.512 | 1.162 | 1.66E-03 | 2.39E-02 | Up in Noninvoluted |
| **UNQ830** | 1.371 | 0.246 | 2.481 | 1.65E-03 | 2.39E-02 | Up in Noninvoluted |
| **RLTPR** | 0.650 | 2.261 | -1.798 | 1.59E-03 | 2.39E-02 | Down in Noninvoluted |
| **RAB9A** | 0.923 | 1.267 | -0.457 | 1.53E-03 | 2.39E-02 | Down in Noninvoluted |
| **C19ORF48** | 1.302 | 0.733 | 0.829 | 1.64E-03 | 2.39E-02 | Up in Noninvoluted |
| **SEC23B** | 1.123 | 0.848 | 0.405 | 1.65E-03 | 2.39E-02 | Up in Noninvoluted |
| **SARDH** | 1.502 | 0.087 | 4.105 | 1.52E-03 | 2.39E-02 | Up in Noninvoluted |
| **FAM113A** | 1.152 | 0.832 | 0.470 | 1.84E-03 | 2.62E-02 | Up in Noninvoluted |
| **DTNB** | 1.357 | 0.561 | 1.274 | 1.86E-03 | 2.63E-02 | Up in Noninvoluted |
| **FHDC1** | 1.432 | 0.315 | 2.186 | 1.97E-03 | 2.63E-02 | Up in Noninvoluted |
| **OTUB2** | 1.172 | 0.490 | 1.257 | 1.95E-03 | 2.63E-02 | Up in Noninvoluted |
| **RAB15** | 1.254 | 0.572 | 1.132 | 1.94E-03 | 2.63E-02 | Up in Noninvoluted |
| **GOLGA6** | 0.728 | 1.200 | -0.720 | 1.97E-03 | 2.63E-02 | Down in Noninvoluted |
| **DPYSL4** | 1.606 | 0.391 | 2.040 | 2.00E-03 | 2.63E-02 | Up in Noninvoluted |
| **LAD1** | 1.342 | 0.650 | 1.046 | 1.93E-03 | 2.63E-02 | Up in Noninvoluted |
| **PLS1** | 1.709 | 0.343 | 2.316 | 1.89E-03 | 2.63E-02 | Up in Noninvoluted |
| **COL27A1** | 1.250 | 0.556 | 1.168 | 2.00E-03 | 2.63E-02 | Up in Noninvoluted |
| **TPST2** | 0.791 | 2.548 | -1.688 | 2.03E-03 | 2.63E-02 | Down in Noninvoluted |
| **BID** | 1.162 | 0.433 | 1.423 | 2.13E-03 | 2.63E-02 | Up in Noninvoluted |
| **ETV4** | 1.713 | 0.209 | 3.033 | 2.15E-03 | 2.63E-02 | Up in Noninvoluted |
| **ZWINT** | 1.351 | 0.601 | 1.169 | 2.07E-03 | 2.63E-02 | Up in Noninvoluted |
| **HECA** | 0.827 | 1.448 | -0.809 | 2.17E-03 | 2.63E-02 | Down in Noninvoluted |
| **FLJ10996** | 0.848 | 1.167 | -0.460 | 2.16E-03 | 2.63E-02 | Down in Noninvoluted |
| **OPN3** | 1.284 | 0.664 | 0.950 | 2.18E-03 | 2.63E-02 | Up in Noninvoluted |
| **PICALM** | 0.834 | 1.697 | -1.025 | 2.14E-03 | 2.63E-02 | Down in Noninvoluted |
| **ILKAP** | 0.891 | 1.891 | -1.085 | 2.09E-03 | 2.63E-02 | Down in Noninvoluted |
| **HNRNPH2** | 0.810 | 1.204 | -0.572 | 2.22E-03 | 2.65E-02 | Down in Noninvoluted |
| **DDX41** | 1.254 | 0.809 | 0.631 | 2.26E-03 | 2.65E-02 | Up in Noninvoluted |
| **WDR17** | 1.552 | 0.276 | 2.494 | 2.27E-03 | 2.65E-02 | Up in Noninvoluted |
| **AQP5** | 1.942 | 0.059 | 5.037 | 2.23E-03 | 2.65E-02 | Up in Noninvoluted |
| **WDR26** | 0.874 | 1.297 | -0.569 | 2.35E-03 | 2.72E-02 | Down in Noninvoluted |
| **UCK2** | 1.134 | 0.489 | 1.212 | 2.55E-03 | 2.79E-02 | Up in Noninvoluted |
| **CXCL2** | 1.396 | 0.684 | 1.030 | 2.49E-03 | 2.79E-02 | Up in Noninvoluted |
| **ZNF598** | 1.418 | 0.610 | 1.217 | 2.59E-03 | 2.79E-02 | Up in Noninvoluted |
| **RPS3A** | 1.220 | 0.794 | 0.618 | 2.48E-03 | 2.79E-02 | Up in Noninvoluted |
| **ABCC10** | 0.812 | 1.395 | -0.781 | 2.53E-03 | 2.79E-02 | Down in Noninvoluted |
| **STIM1** | 0.831 | 1.312 | -0.659 | 2.50E-03 | 2.79E-02 | Down in Noninvoluted |
| **HIST1H3D** | 1.232 | 0.556 | 1.146 | 2.59E-03 | 2.79E-02 | Up in Noninvoluted |
| **NLRP1** | 0.802 | 1.586 | -0.983 | 2.57E-03 | 2.79E-02 | Down in Noninvoluted |
| **RIMS3** | 2.179 | 0.231 | 3.239 | 2.51E-03 | 2.79E-02 | Up in Noninvoluted |
| **PEX26** | 0.842 | 1.116 | -0.405 | 2.64E-03 | 2.81E-02 | Down in Noninvoluted |
| **LPAR4** | 1.392 | 0.578 | 1.268 | 2.63E-03 | 2.81E-02 | Up in Noninvoluted |
| **LRAT** | 1.143 | 0.221 | 2.374 | 2.73E-03 | 2.83E-02 | Up in Noninvoluted |
| **FAM29A** | 1.194 | 0.650 | 0.876 | 2.74E-03 | 2.83E-02 | Up in Noninvoluted |
| **ZNF710** | 1.219 | 0.476 | 1.357 | 2.80E-03 | 2.83E-02 | Up in Noninvoluted |
| **IGSF5** | 1.575 | 0.272 | 2.533 | 2.79E-03 | 2.83E-02 | Up in Noninvoluted |
| **SFT2D1** | 0.803 | 1.303 | -0.698 | 2.80E-03 | 2.83E-02 | Down in Noninvoluted |
| **S100A10** | 0.837 | 1.386 | -0.728 | 2.73E-03 | 2.83E-02 | Down in Noninvoluted |
| **BTBD3** | 1.219 | 0.736 | 0.728 | 2.80E-03 | 2.83E-02 | Up in Noninvoluted |
| **BOC** | 1.183 | 0.750 | 0.658 | 2.91E-03 | 2.88E-02 | Up in Noninvoluted |
| **HTATSF1** | 0.781 | 1.238 | -0.665 | 2.94E-03 | 2.88E-02 | Down in Noninvoluted |
| **B3GALNT2** | 1.072 | 0.776 | 0.466 | 2.93E-03 | 2.88E-02 | Up in Noninvoluted |
| **GPR126** | 1.260 | 0.701 | 0.845 | 2.93E-03 | 2.88E-02 | Up in Noninvoluted |
| **CXORF15** | 0.899 | 1.554 | -0.790 | 2.96E-03 | 2.88E-02 | Down in Noninvoluted |
| **CRYBB3** | 1.428 | 0.152 | 3.228 | 3.11E-03 | 3.00E-02 | Up in Noninvoluted |
| **CAV1** | 0.899 | 1.523 | -0.761 | 3.13E-03 | 3.00E-02 | Down in Noninvoluted |
| **BEX5** | 0.987 | 1.614 | -0.709 | 3.14E-03 | 3.00E-02 | Down in Noninvoluted |
| **PTHLH** | 1.672 | 0.315 | 2.409 | 3.18E-03 | 3.00E-02 | Up in Noninvoluted |
| **MEGF9** | 0.888 | 1.302 | -0.551 | 3.19E-03 | 3.00E-02 | Down in Noninvoluted |
| **RPL23A** | 1.186 | 0.760 | 0.641 | 3.35E-03 | 3.14E-02 | Up in Noninvoluted |
| **BRCA2** | 1.347 | 0.582 | 1.211 | 3.44E-03 | 3.16E-02 | Up in Noninvoluted |
| **APOLD1** | 0.661 | 2.495 | -1.917 | 3.40E-03 | 3.16E-02 | Down in Noninvoluted |
| **CSF1** | 0.913 | 1.318 | -0.529 | 3.43E-03 | 3.16E-02 | Down in Noninvoluted |
| **NRG3** | 1.589 | 0.108 | 3.873 | 3.46E-03 | 3.16E-02 | Up in Noninvoluted |
| **MMP16** | 1.136 | 0.520 | 1.129 | 3.56E-03 | 3.20E-02 | Up in Noninvoluted |
| **NSFL1C** | 0.941 | 1.212 | -0.365 | 3.58E-03 | 3.20E-02 | Down in Noninvoluted |
| **FAM155A** | 1.231 | 0.386 | 1.673 | 3.56E-03 | 3.20E-02 | Up in Noninvoluted |
| **RAB5A** | 0.810 | 1.705 | -1.074 | 3.62E-03 | 3.22E-02 | Down in Noninvoluted |
| **WWC3** | 0.946 | 1.183 | -0.323 | 3.71E-03 | 3.27E-02 | Down in Noninvoluted |
| **TEX9** | 1.246 | 0.672 | 0.892 | 3.74E-03 | 3.28E-02 | Up in Noninvoluted |
| **LOC387646** | 1.442 | 0.524 | 1.460 | 3.81E-03 | 3.31E-02 | Up in Noninvoluted |
| **LYNX1** | 1.418 | 0.538 | 1.397 | 3.82E-03 | 3.31E-02 | Up in Noninvoluted |
| **ILF3** | 1.130 | 0.779 | 0.537 | 3.88E-03 | 3.34E-02 | Up in Noninvoluted |
| **CORO7** | 2.200 | 0.264 | 3.060 | 3.96E-03 | 3.39E-02 | Up in Noninvoluted |
| **UBXN11** | 1.153 | 0.778 | 0.566 | 4.05E-03 | 3.43E-02 | Up in Noninvoluted |
| **TOP1MT** | 1.309 | 0.869 | 0.591 | 4.03E-03 | 3.43E-02 | Up in Noninvoluted |
| **RORC** | 1.248 | 0.663 | 0.912 | 4.16E-03 | 3.48E-02 | Up in Noninvoluted |
| **FAM175B** | 0.735 | 1.226 | -0.738 | 4.15E-03 | 3.48E-02 | Down in Noninvoluted |
| **CES4** | 0.717 | 2.472 | -1.786 | 4.19E-03 | 3.48E-02 | Down in Noninvoluted |
| **ANO9** | 1.139 | 0.439 | 1.374 | 4.28E-03 | 3.50E-02 | Up in Noninvoluted |
| **SOX12** | 1.481 | 0.371 | 1.996 | 4.23E-03 | 3.50E-02 | Up in Noninvoluted |
| **AQP1** | 0.777 | 1.402 | -0.852 | 4.26E-03 | 3.50E-02 | Down in Noninvoluted |
| **TXNDC5** | 1.210 | 0.801 | 0.595 | 4.35E-03 | 3.51E-02 | Up in Noninvoluted |
| **DDX24** | 1.191 | 0.845 | 0.494 | 4.37E-03 | 3.51E-02 | Up in Noninvoluted |
| **DCX** | 2.147 | 0.292 | 2.876 | 4.36E-03 | 3.51E-02 | Up in Noninvoluted |
| **CYP7B1** | 1.153 | 0.778 | 0.567 | 4.45E-03 | 3.52E-02 | Up in Noninvoluted |
| **CARD8** | 0.716 | 2.091 | -1.546 | 4.43E-03 | 3.52E-02 | Down in Noninvoluted |
| **CD300A** | 1.239 | 0.464 | 1.417 | 4.45E-03 | 3.52E-02 | Up in Noninvoluted |
| **SURF2** | 1.102 | 0.689 | 0.678 | 4.48E-03 | 3.52E-02 | Up in Noninvoluted |
| **IL17RA** | 1.293 | 0.717 | 0.852 | 4.55E-03 | 3.56E-02 | Up in Noninvoluted |
| **CRLS1** | 0.638 | 1.353 | -1.085 | 4.60E-03 | 3.57E-02 | Down in Noninvoluted |
| **RBM6** | 1.120 | 0.659 | 0.765 | 4.62E-03 | 3.57E-02 | Up in Noninvoluted |
| **CYP46A1** | 1.326 | 0.275 | 2.270 | 4.71E-03 | 3.59E-02 | Up in Noninvoluted |
| **JPH1** | 1.161 | 0.588 | 0.983 | 4.89E-03 | 3.59E-02 | Up in Noninvoluted |
| **NT5DC2** | 1.209 | 0.672 | 0.847 | 4.92E-03 | 3.59E-02 | Up in Noninvoluted |
| **MATK** | 1.270 | 0.637 | 0.996 | 4.75E-03 | 3.59E-02 | Up in Noninvoluted |
| **A26C3** | 1.344 | 0.740 | 0.861 | 4.82E-03 | 3.59E-02 | Up in Noninvoluted |
| **ZNF749** | 1.140 | 0.661 | 0.786 | 4.79E-03 | 3.59E-02 | Up in Noninvoluted |
| **ETFA** | 0.691 | 2.487 | -1.848 | 4.92E-03 | 3.59E-02 | Down in Noninvoluted |
| **RPL17** | 1.117 | 0.843 | 0.406 | 4.91E-03 | 3.59E-02 | Up in Noninvoluted |
| **CLSTN2** | 0.834 | 1.338 | -0.682 | 4.73E-03 | 3.59E-02 | Down in Noninvoluted |
| **C4ORF41** | 0.776 | 1.415 | -0.866 | 4.86E-03 | 3.59E-02 | Down in Noninvoluted |
| **FLJ20254** | 0.915 | 1.445 | -0.659 | 4.92E-03 | 3.59E-02 | Down in Noninvoluted |
| **LOC441376** | 1.270 | 0.702 | 0.855 | 5.01E-03 | 3.62E-02 | Up in Noninvoluted |
| **RNF14** | 0.764 | 1.382 | -0.854 | 4.99E-03 | 3.62E-02 | Down in Noninvoluted |
| **TMEM148** | 1.571 | 0.675 | 1.218 | 5.31E-03 | 3.64E-02 | Up in Noninvoluted |
| **LRGUK** | 1.252 | 0.362 | 1.788 | 5.28E-03 | 3.64E-02 | Up in Noninvoluted |
| **CICK0721Q.1** | 1.121 | 0.592 | 0.920 | 5.23E-03 | 3.64E-02 | Up in Noninvoluted |
| **STAP2** | 1.150 | 0.687 | 0.743 | 5.17E-03 | 3.64E-02 | Up in Noninvoluted |
| **SND1** | 1.142 | 0.611 | 0.902 | 5.21E-03 | 3.64E-02 | Up in Noninvoluted |
| **B4GALT7** | 1.453 | 0.718 | 1.018 | 5.36E-03 | 3.64E-02 | Up in Noninvoluted |
| **SERINC1** | 0.897 | 1.131 | -0.334 | 5.35E-03 | 3.64E-02 | Down in Noninvoluted |
| **ADH1B** | 0.805 | 1.953 | -1.277 | 5.08E-03 | 3.64E-02 | Down in Noninvoluted |
| **CSRP2** | 0.772 | 1.540 | -0.996 | 5.16E-03 | 3.64E-02 | Down in Noninvoluted |
| **ZNF205** | 0.789 | 1.559 | -0.982 | 5.37E-03 | 3.64E-02 | Down in Noninvoluted |
| **TP73** | 1.904 | 0.304 | 2.649 | 5.20E-03 | 3.64E-02 | Up in Noninvoluted |
| **RLN2** | 1.449 | 0.491 | 1.561 | 5.18E-03 | 3.64E-02 | Up in Noninvoluted |
| **NCAPG** | 2.073 | 0.320 | 2.697 | 5.34E-03 | 3.64E-02 | Up in Noninvoluted |
| **PPAP2C** | 1.161 | 0.517 | 1.168 | 5.65E-03 | 3.64E-02 | Up in Noninvoluted |
| **CASS4** | 1.543 | 0.441 | 1.808 | 5.57E-03 | 3.64E-02 | Up in Noninvoluted |
| **TCTEX1D2** | 1.759 | 0.387 | 2.185 | 5.71E-03 | 3.64E-02 | Up in Noninvoluted |
| **LRRN1** | 1.927 | 0.442 | 2.125 | 5.77E-03 | 3.64E-02 | Up in Noninvoluted |
| **TWISTNB** | 1.076 | 0.740 | 0.539 | 5.69E-03 | 3.64E-02 | Up in Noninvoluted |
| **SMC4** | 1.232 | 0.718 | 0.778 | 5.73E-03 | 3.64E-02 | Up in Noninvoluted |
| **PLEKHO2** | 0.809 | 1.378 | -0.768 | 5.46E-03 | 3.64E-02 | Down in Noninvoluted |
| **RPS2** | 1.140 | 0.833 | 0.452 | 5.49E-03 | 3.64E-02 | Up in Noninvoluted |
| **ZNF233** | 0.789 | 1.264 | -0.680 | 5.61E-03 | 3.64E-02 | Down in Noninvoluted |
| **GRHL1** | 1.349 | 0.808 | 0.740 | 5.74E-03 | 3.64E-02 | Up in Noninvoluted |
| **MLF1IP** | 1.524 | 0.521 | 1.548 | 5.60E-03 | 3.64E-02 | Up in Noninvoluted |
| **RBBP8** | 1.250 | 0.922 | 0.439 | 5.52E-03 | 3.64E-02 | Up in Noninvoluted |
| **FAM33A** | 0.881 | 1.283 | -0.542 | 5.62E-03 | 3.64E-02 | Down in Noninvoluted |
| **KRT18** | 2.572 | 0.344 | 2.903 | 5.60E-03 | 3.64E-02 | Up in Noninvoluted |
| **FAM109B** | 1.308 | 0.239 | 2.452 | 5.76E-03 | 3.64E-02 | Up in Noninvoluted |
| **FGF1** | 1.107 | 0.667 | 0.732 | 5.82E-03 | 3.66E-02 | Up in Noninvoluted |
| **CAP2** | 0.863 | 1.317 | -0.610 | 5.85E-03 | 3.66E-02 | Down in Noninvoluted |
| **HDAC8** | 1.193 | 0.779 | 0.614 | 5.91E-03 | 3.68E-02 | Up in Noninvoluted |
| **CCL28** | 1.309 | 0.758 | 0.788 | 6.02E-03 | 3.72E-02 | Up in Noninvoluted |
| **TRUB2** | 1.267 | 0.833 | 0.605 | 6.00E-03 | 3.72E-02 | Up in Noninvoluted |
| **DDAH1** | 1.376 | 0.313 | 2.135 | 6.12E-03 | 3.72E-02 | Up in Noninvoluted |
| **LTA4H** | 1.126 | 0.610 | 0.884 | 6.44E-03 | 3.72E-02 | Up in Noninvoluted |
| **CPT1B** | 1.130 | 0.669 | 0.757 | 6.35E-03 | 3.72E-02 | Up in Noninvoluted |
| **TAF1A** | 1.195 | 0.612 | 0.965 | 6.50E-03 | 3.72E-02 | Up in Noninvoluted |
| **SLC9A3R1** | 1.257 | 0.728 | 0.787 | 6.27E-03 | 3.72E-02 | Up in Noninvoluted |
| **DSC2** | 1.134 | 0.743 | 0.610 | 6.19E-03 | 3.72E-02 | Up in Noninvoluted |
| **GMEB1** | 1.251 | 0.674 | 0.893 | 6.30E-03 | 3.72E-02 | Up in Noninvoluted |
| **E2F2** | 1.378 | 0.459 | 1.585 | 6.28E-03 | 3.72E-02 | Up in Noninvoluted |
| **FKBP5** | 0.832 | 1.822 | -1.130 | 6.14E-03 | 3.72E-02 | Down in Noninvoluted |
| **KCTD15** | 1.348 | 0.764 | 0.819 | 6.40E-03 | 3.72E-02 | Up in Noninvoluted |
| **SRGAP3** | 1.200 | 0.791 | 0.601 | 6.33E-03 | 3.72E-02 | Up in Noninvoluted |
| **DHRS4L2** | 0.709 | 1.254 | -0.824 | 6.50E-03 | 3.72E-02 | Down in Noninvoluted |
| **SLC9A3** | 1.270 | 0.610 | 1.059 | 6.18E-03 | 3.72E-02 | Up in Noninvoluted |
| **C9ORF75** | 0.719 | 1.480 | -1.043 | 6.53E-03 | 3.72E-02 | Down in Noninvoluted |
| **PMEPA1** | 0.887 | 2.343 | -1.401 | 6.49E-03 | 3.72E-02 | Down in Noninvoluted |
| **SC5DL** | 0.790 | 1.495 | -0.920 | 6.40E-03 | 3.72E-02 | Down in Noninvoluted |
| **PADI2** | 1.313 | 0.677 | 0.956 | 6.52E-03 | 3.72E-02 | Up in Noninvoluted |
| **BTN2A3** | 2.297 | 0.120 | 4.263 | 6.41E-03 | 3.72E-02 | Up in Noninvoluted |
| **ADH1A** | 0.869 | 39.860 | -5.519 | 6.09E-03 | 3.72E-02 | Down in Noninvoluted |
| **CORO2B** | 0.634 | 1.668 | -1.396 | 6.57E-03 | 3.73E-02 | Down in Noninvoluted |
| **C6ORF224** | 1.850 | 0.501 | 1.886 | 6.60E-03 | 3.73E-02 | Up in Noninvoluted |
| **STX7** | 0.765 | 1.971 | -1.365 | 6.62E-03 | 3.73E-02 | Down in Noninvoluted |
| **PAK1IP1** | 1.100 | 0.625 | 0.815 | 6.68E-03 | 3.73E-02 | Up in Noninvoluted |
| **C11ORF82** | 1.844 | 0.321 | 2.522 | 6.67E-03 | 3.73E-02 | Up in Noninvoluted |
| **ADIPOR1** | 0.874 | 1.226 | -0.489 | 6.71E-03 | 3.74E-02 | Down in Noninvoluted |
| **GINS4** | 1.355 | 0.485 | 1.480 | 6.77E-03 | 3.74E-02 | Up in Noninvoluted |
| **FUT6** | 2.349 | 0.113 | 4.381 | 6.77E-03 | 3.74E-02 | Up in Noninvoluted |
| **METTL6** | 1.108 | 0.887 | 0.321 | 6.80E-03 | 3.74E-02 | Up in Noninvoluted |
| **TMCO4** | 1.113 | 0.614 | 0.858 | 6.86E-03 | 3.76E-02 | Up in Noninvoluted |
| **SFRS2B** | 1.075 | 0.804 | 0.418 | 6.92E-03 | 3.76E-02 | Up in Noninvoluted |
| **WBP2NL** | 1.374 | 0.618 | 1.153 | 6.89E-03 | 3.76E-02 | Up in Noninvoluted |
| **PRMT5** | 1.149 | 0.686 | 0.744 | 6.94E-03 | 3.76E-02 | Up in Noninvoluted |
| **PHLDA3** | 0.745 | 1.665 | -1.161 | 6.97E-03 | 3.76E-02 | Down in Noninvoluted |
| **GTF3C5** | 1.390 | 0.272 | 2.356 | 7.02E-03 | 3.77E-02 | Up in Noninvoluted |
| **MRPL14** | 1.261 | 0.706 | 0.837 | 7.10E-03 | 3.77E-02 | Up in Noninvoluted |
| **NUMB** | 0.860 | 1.110 | -0.368 | 7.18E-03 | 3.77E-02 | Down in Noninvoluted |
| **CCBL1** | 0.843 | 1.456 | -0.789 | 7.17E-03 | 3.77E-02 | Down in Noninvoluted |
| **MTUS1** | 0.837 | 1.305 | -0.641 | 7.14E-03 | 3.77E-02 | Down in Noninvoluted |
| **CUL3** | 1.206 | 0.703 | 0.778 | 7.06E-03 | 3.77E-02 | Up in Noninvoluted |
| **HIST1H2BM** | 1.535 | 0.550 | 1.482 | 7.16E-03 | 3.77E-02 | Up in Noninvoluted |
| **OAT** | 1.214 | 0.840 | 0.531 | 7.22E-03 | 3.78E-02 | Up in Noninvoluted |
| **ADAMTS8** | 1.338 | 0.639 | 1.065 | 7.27E-03 | 3.79E-02 | Up in Noninvoluted |
| **MRPL42** | 1.392 | 0.662 | 1.072 | 7.46E-03 | 3.83E-02 | Up in Noninvoluted |
| **HAPLN3** | 1.415 | 0.720 | 0.976 | 7.58E-03 | 3.83E-02 | Up in Noninvoluted |
| **CXCL1** | 1.315 | 0.619 | 1.088 | 7.53E-03 | 3.83E-02 | Up in Noninvoluted |
| **CXADR** | 1.149 | 0.544 | 1.078 | 7.39E-03 | 3.83E-02 | Up in Noninvoluted |
| **CBLB** | 0.803 | 1.290 | -0.684 | 7.48E-03 | 3.83E-02 | Down in Noninvoluted |
| **ZMYM5** | 0.941 | 1.076 | -0.192 | 7.58E-03 | 3.83E-02 | Down in Noninvoluted |
| **ANAPC13** | 0.958 | 1.403 | -0.551 | 7.41E-03 | 3.83E-02 | Down in Noninvoluted |
| **HOXC8** | 0.684 | 1.442 | -1.076 | 7.55E-03 | 3.83E-02 | Down in Noninvoluted |
| **DDX23** | 1.778 | 0.130 | 3.774 | 7.49E-03 | 3.83E-02 | Up in Noninvoluted |
| **B3GNT2** | 1.074 | 0.686 | 0.646 | 7.62E-03 | 3.83E-02 | Up in Noninvoluted |
| **EDEM2** | 1.142 | 0.754 | 0.599 | 7.71E-03 | 3.87E-02 | Up in Noninvoluted |
| **TMEM185A** | 0.844 | 1.169 | -0.470 | 7.79E-03 | 3.88E-02 | Down in Noninvoluted |
| **SDCBP** | 0.845 | 1.359 | -0.686 | 7.78E-03 | 3.88E-02 | Down in Noninvoluted |
| **PAK3** | 2.159 | 0.402 | 2.426 | 7.82E-03 | 3.88E-02 | Up in Noninvoluted |
| **SMYD3** | 1.237 | 0.748 | 0.726 | 7.87E-03 | 3.89E-02 | Up in Noninvoluted |
| **DDX1** | 1.113 | 0.817 | 0.447 | 7.92E-03 | 3.89E-02 | Up in Noninvoluted |
| **MTRF1** | 1.261 | 0.657 | 0.940 | 7.91E-03 | 3.89E-02 | Up in Noninvoluted |
| **ST6GALNAC3** | 0.782 | 1.968 | -1.331 | 7.99E-03 | 3.91E-02 | Down in Noninvoluted |
| **RGMA** | 1.148 | 0.490 | 1.229 | 8.26E-03 | 3.91E-02 | Up in Noninvoluted |
| **SHC4** | 1.545 | 0.320 | 2.270 | 8.10E-03 | 3.91E-02 | Up in Noninvoluted |
| **C12ORF66** | 1.165 | 0.715 | 0.704 | 8.24E-03 | 3.91E-02 | Up in Noninvoluted |
| **ITK** | 0.709 | 1.808 | -1.350 | 8.23E-03 | 3.91E-02 | Down in Noninvoluted |
| **SFT2D2** | 1.158 | 0.804 | 0.525 | 8.27E-03 | 3.91E-02 | Up in Noninvoluted |
| **PMP22** | 0.923 | 1.255 | -0.444 | 8.15E-03 | 3.91E-02 | Down in Noninvoluted |
| **PDE1C** | 1.721 | 0.326 | 2.402 | 8.09E-03 | 3.91E-02 | Up in Noninvoluted |
| **KCNN4** | 1.333 | 0.720 | 0.889 | 8.14E-03 | 3.91E-02 | Up in Noninvoluted |
| **TMEM171** | 1.884 | 0.480 | 1.972 | 8.04E-03 | 3.91E-02 | Up in Noninvoluted |
| **GJB2** | 1.703 | 0.245 | 2.799 | 8.26E-03 | 3.91E-02 | Up in Noninvoluted |
| **TOP2A** | 2.274 | 0.080 | 4.821 | 8.34E-03 | 3.93E-02 | Up in Noninvoluted |
| **LRRC4B** | 1.647 | 0.362 | 2.186 | 8.37E-03 | 3.93E-02 | Up in Noninvoluted |
| **GALNAC4S-6ST** | 1.100 | 0.554 | 0.991 | 8.48E-03 | 3.93E-02 | Up in Noninvoluted |
| **TRIM14** | 1.218 | 0.637 | 0.936 | 8.49E-03 | 3.93E-02 | Up in Noninvoluted |
| **EPHB3** | 1.490 | 0.659 | 1.177 | 8.45E-03 | 3.93E-02 | Up in Noninvoluted |
| **C2ORF48** | 2.015 | 0.449 | 2.167 | 8.46E-03 | 3.93E-02 | Up in Noninvoluted |
| **SLIT3** | 0.943 | 1.285 | -0.446 | 8.59E-03 | 3.97E-02 | Down in Noninvoluted |
| **GPR107** | 0.643 | 1.332 | -1.050 | 8.73E-03 | 3.98E-02 | Down in Noninvoluted |
| **KNDC1** | 1.613 | 0.479 | 1.750 | 8.74E-03 | 3.98E-02 | Up in Noninvoluted |
| **LYAR** | 1.203 | 0.745 | 0.690 | 8.69E-03 | 3.98E-02 | Up in Noninvoluted |
| **PTGR2** | 1.475 | 0.666 | 1.147 | 8.70E-03 | 3.98E-02 | Up in Noninvoluted |
| **ANXA7** | 0.876 | 1.342 | -0.615 | 8.74E-03 | 3.98E-02 | Down in Noninvoluted |
| **VAMP7** | 0.827 | 1.184 | -0.517 | 8.79E-03 | 3.98E-02 | Down in Noninvoluted |
| **SYNGR1** | 1.239 | 0.879 | 0.496 | 8.85E-03 | 4.00E-02 | Up in Noninvoluted |
| **TM6SF2** | 1.549 | 0.379 | 2.033 | 8.89E-03 | 4.00E-02 | Up in Noninvoluted |
| **RORA** | 1.195 | 0.802 | 0.576 | 9.00E-03 | 4.04E-02 | Up in Noninvoluted |
| **PRPF39** | 1.157 | 0.891 | 0.377 | 9.05E-03 | 4.04E-02 | Up in Noninvoluted |
| **SLC30A6** | 0.931 | 1.259 | -0.436 | 9.09E-03 | 4.04E-02 | Down in Noninvoluted |
| **SOD3** | 0.711 | 1.792 | -1.334 | 9.08E-03 | 4.04E-02 | Down in Noninvoluted |
| **NTF5** | 1.305 | 0.442 | 1.561 | 9.16E-03 | 4.05E-02 | Up in Noninvoluted |
| **GALNT4** | 1.112 | 0.734 | 0.600 | 9.14E-03 | 4.05E-02 | Up in Noninvoluted |
| **ZMIZ2** | 1.124 | 0.711 | 0.660 | 9.22E-03 | 4.05E-02 | Up in Noninvoluted |
| **NTRK1** | 1.413 | 0.474 | 1.577 | 9.21E-03 | 4.05E-02 | Up in Noninvoluted |
| **MIER3** | 0.715 | 1.260 | -0.818 | 9.29E-03 | 4.06E-02 | Down in Noninvoluted |
| **MPN2** | 0.786 | 11.760 | -3.903 | 9.28E-03 | 4.06E-02 | Down in Noninvoluted |
| **FAM83H** | 1.408 | 0.643 | 1.132 | 9.32E-03 | 4.06E-02 | Up in Noninvoluted |
| **ADAMTS1** | 0.938 | 1.251 | -0.415 | 9.40E-03 | 4.08E-02 | Down in Noninvoluted |
| **ANKRA2** | 0.593 | 1.297 | -1.128 | 9.49E-03 | 4.08E-02 | Down in Noninvoluted |
| **ZNF425** | 1.435 | 0.341 | 2.074 | 9.46E-03 | 4.08E-02 | Up in Noninvoluted |
| **PPP1R12B** | 0.820 | 1.142 | -0.478 | 9.47E-03 | 4.08E-02 | Down in Noninvoluted |
| **SCML1** | 0.719 | 1.261 | -0.811 | 9.56E-03 | 4.10E-02 | Down in Noninvoluted |
| **ACAP3** | 1.248 | 0.707 | 0.821 | 9.67E-03 | 4.10E-02 | Up in Noninvoluted |
| **TIMP3** | 0.936 | 1.373 | -0.554 | 9.69E-03 | 4.10E-02 | Down in Noninvoluted |
| **TCF4** | 0.824 | 1.304 | -0.663 | 9.70E-03 | 4.10E-02 | Down in Noninvoluted |
| **STS-1** | 1.860 | 0.726 | 1.357 | 9.65E-03 | 4.10E-02 | Up in Noninvoluted |
| **NAV3** | 0.751 | 1.845 | -1.297 | 9.72E-03 | 4.10E-02 | Down in Noninvoluted |
| **UBE2C** | 1.562 | 0.337 | 2.213 | 9.85E-03 | 4.14E-02 | Up in Noninvoluted |
| **PLEKHJ1** | 1.265 | 0.786 | 0.687 | 9.87E-03 | 4.14E-02 | Up in Noninvoluted |
| **TNNT2** | 1.450 | 0.662 | 1.131 | 9.93E-03 | 4.15E-02 | Up in Noninvoluted |
| **CLDN14** | 0.883 | 1.640 | -0.893 | 9.95E-03 | 4.15E-02 | Down in Noninvoluted |
| **CHRDL1** | 0.669 | 1.599 | -1.256 | 9.98E-03 | 4.15E-02 | Down in Noninvoluted |
| **ATP1A2** | 0.645 | 2.353 | -1.868 | 1.00E-02 | 4.15E-02 | Down in Noninvoluted |
| **TRAF1** | 1.359 | 0.838 | 0.697 | 1.00E-02 | 4.15E-02 | Up in Noninvoluted |
| **LRRC37A4** | 2.141 | 0.200 | 3.417 | 1.01E-02 | 4.15E-02 | Up in Noninvoluted |
| **SLAIN1** | 1.263 | 0.421 | 1.584 | 1.02E-02 | 4.16E-02 | Up in Noninvoluted |
| **GPD2** | 1.228 | 0.867 | 0.503 | 1.01E-02 | 4.16E-02 | Up in Noninvoluted |
| **TFRC** | 3.143 | 0.507 | 2.633 | 1.02E-02 | 4.16E-02 | Up in Noninvoluted |
| **LOC255374** | 2.184 | 0.313 | 2.804 | 1.03E-02 | 4.18E-02 | Up in Noninvoluted |
| **NKIRAS2** | 1.193 | 0.838 | 0.509 | 1.03E-02 | 4.18E-02 | Up in Noninvoluted |
| **ERBB2IP** | 1.047 | 0.807 | 0.375 | 1.04E-02 | 4.20E-02 | Up in Noninvoluted |
| **FAM164C** | 1.322 | 0.744 | 0.831 | 1.04E-02 | 4.20E-02 | Up in Noninvoluted |
| **KCNAB3** | 1.207 | 0.348 | 1.794 | 1.12E-02 | 4.21E-02 | Up in Noninvoluted |
| **USP54** | 1.068 | 0.639 | 0.742 | 1.13E-02 | 4.21E-02 | Up in Noninvoluted |
| **S100A14** | 1.222 | 0.747 | 0.710 | 1.08E-02 | 4.21E-02 | Up in Noninvoluted |
| **RUNX1** | 1.264 | 0.737 | 0.778 | 1.09E-02 | 4.21E-02 | Up in Noninvoluted |
| **NCAPD2** | 1.305 | 0.606 | 1.108 | 1.10E-02 | 4.21E-02 | Up in Noninvoluted |
| **PLEKHB1** | 1.220 | 0.713 | 0.776 | 1.11E-02 | 4.21E-02 | Up in Noninvoluted |
| **HIST1H2BK** | 1.106 | 0.744 | 0.573 | 1.12E-02 | 4.21E-02 | Up in Noninvoluted |
| **GABRR1** | 2.166 | 0.382 | 2.503 | 1.11E-02 | 4.21E-02 | Up in Noninvoluted |
| **TXNDC4** | 1.214 | 0.779 | 0.641 | 1.13E-02 | 4.21E-02 | Up in Noninvoluted |
| **IRAK1BP1** | 1.268 | 0.748 | 0.762 | 1.05E-02 | 4.21E-02 | Up in Noninvoluted |
| **C2ORF40** | 1.191 | 0.822 | 0.535 | 1.09E-02 | 4.21E-02 | Up in Noninvoluted |
| **RPL4** | 1.106 | 0.844 | 0.390 | 1.13E-02 | 4.21E-02 | Up in Noninvoluted |
| **ATRX** | 1.355 | 0.777 | 0.802 | 1.10E-02 | 4.21E-02 | Up in Noninvoluted |
| **C8ORF42** | 1.398 | 0.801 | 0.803 | 1.10E-02 | 4.21E-02 | Up in Noninvoluted |
| **RAB27B** | 1.069 | 0.796 | 0.425 | 1.05E-02 | 4.21E-02 | Up in Noninvoluted |
| **BDP1** | 1.089 | 0.836 | 0.381 | 1.05E-02 | 4.21E-02 | Up in Noninvoluted |
| **MAP2K3** | 1.070 | 0.826 | 0.374 | 1.13E-02 | 4.21E-02 | Up in Noninvoluted |
| **NDUFC2** | 0.629 | 1.410 | -1.165 | 1.10E-02 | 4.21E-02 | Down in Noninvoluted |
| **IGF1** | 1.367 | 0.751 | 0.865 | 1.06E-02 | 4.21E-02 | Up in Noninvoluted |
| **ZBTB44** | 0.906 | 1.137 | -0.328 | 1.07E-02 | 4.21E-02 | Down in Noninvoluted |
| **ANKRD20A1** | 0.904 | 1.503 | -0.734 | 1.09E-02 | 4.21E-02 | Down in Noninvoluted |
| **ZP3** | 1.720 | 0.615 | 1.483 | 1.08E-02 | 4.21E-02 | Up in Noninvoluted |
| **ST8SIA4** | 0.889 | 1.717 | -0.949 | 1.09E-02 | 4.21E-02 | Down in Noninvoluted |
| **LOC653604** | 1.265 | 0.739 | 0.775 | 1.12E-02 | 4.21E-02 | Up in Noninvoluted |
| **CLSPN** | 1.594 | 0.638 | 1.320 | 1.12E-02 | 4.21E-02 | Up in Noninvoluted |
| **SOX13** | 0.907 | 1.437 | -0.664 | 1.10E-02 | 4.21E-02 | Down in Noninvoluted |
| **NME3** | 0.788 | 1.394 | -0.824 | 1.07E-02 | 4.21E-02 | Down in Noninvoluted |
| **LRRC27** | 1.419 | 0.188 | 2.914 | 1.09E-02 | 4.21E-02 | Up in Noninvoluted |
| **ECEL1** | 3.657 | 0.136 | 4.748 | 1.07E-02 | 4.21E-02 | Up in Noninvoluted |
| **SLC4A11** | 1.747 | 0.106 | 4.048 | 1.11E-02 | 4.21E-02 | Up in Noninvoluted |
| **SLC17A7** | 1.590 | 0.560 | 1.505 | 1.17E-02 | 4.22E-02 | Up in Noninvoluted |
| **HUNK** | 1.259 | 0.326 | 1.949 | 1.16E-02 | 4.22E-02 | Up in Noninvoluted |
| **PURA** | 0.734 | 1.274 | -0.796 | 1.17E-02 | 4.22E-02 | Down in Noninvoluted |
| **KAZALD1** | 0.673 | 1.507 | -1.162 | 1.14E-02 | 4.22E-02 | Down in Noninvoluted |
| **RPA4** | 0.652 | 1.811 | -1.475 | 1.16E-02 | 4.22E-02 | Down in Noninvoluted |
| **CCDC34** | 1.222 | 0.891 | 0.456 | 1.16E-02 | 4.22E-02 | Up in Noninvoluted |
| **WBP11** | 0.842 | 1.350 | -0.681 | 1.15E-02 | 4.22E-02 | Down in Noninvoluted |
| **FBXO3** | 0.897 | 1.362 | -0.602 | 1.14E-02 | 4.22E-02 | Down in Noninvoluted |
| **GLT25D2** | 2.032 | 0.241 | 3.077 | 1.17E-02 | 4.22E-02 | Up in Noninvoluted |
| **EMX2** | 0.772 | 1.377 | -0.835 | 1.16E-02 | 4.22E-02 | Down in Noninvoluted |
| **ETFDH** | 0.816 | 1.282 | -0.652 | 1.15E-02 | 4.22E-02 | Down in Noninvoluted |
| **TFIP11** | 0.901 | 1.274 | -0.499 | 1.15E-02 | 4.22E-02 | Down in Noninvoluted |
| **USP34** | 1.074 | 0.829 | 0.374 | 1.17E-02 | 4.22E-02 | Up in Noninvoluted |
| **CDC5L** | 1.194 | 0.826 | 0.531 | 1.18E-02 | 4.22E-02 | Up in Noninvoluted |
| **SEMA4D** | 1.158 | 0.820 | 0.498 | 1.18E-02 | 4.23E-02 | Up in Noninvoluted |
| **MEF2C** | 0.751 | 1.754 | -1.223 | 1.18E-02 | 4.23E-02 | Down in Noninvoluted |
| **FAM111B** | 1.525 | 0.620 | 1.298 | 1.20E-02 | 4.25E-02 | Up in Noninvoluted |
| **GPR146** | 0.966 | 2.559 | -1.406 | 1.20E-02 | 4.25E-02 | Down in Noninvoluted |
| **FMO6P** | 2.133 | 0.462 | 2.208 | 1.20E-02 | 4.25E-02 | Up in Noninvoluted |
| **SFRS2IP** | 0.907 | 1.103 | -0.283 | 1.21E-02 | 4.28E-02 | Down in Noninvoluted |
| **AFAP1L1** | 0.777 | 3.647 | -2.230 | 1.22E-02 | 4.29E-02 | Down in Noninvoluted |
| **MRPS17** | 1.091 | 0.724 | 0.592 | 1.23E-02 | 4.30E-02 | Up in Noninvoluted |
| **GLB1L2** | 1.665 | 0.322 | 2.370 | 1.23E-02 | 4.30E-02 | Up in Noninvoluted |
| **HIST1H2AJ** | 2.178 | 0.459 | 2.245 | 1.23E-02 | 4.30E-02 | Up in Noninvoluted |
| **DMWD** | 0.773 | 1.553 | -1.006 | 1.23E-02 | 4.30E-02 | Down in Noninvoluted |
| **PIP5K3** | 0.934 | 1.245 | -0.414 | 1.23E-02 | 4.30E-02 | Down in Noninvoluted |
| **SCGB3A1** | 1.915 | 0.249 | 2.940 | 1.24E-02 | 4.30E-02 | Up in Noninvoluted |
| **CENPK** | 1.769 | 0.404 | 2.131 | 1.24E-02 | 4.30E-02 | Up in Noninvoluted |
| **C9ORF6** | 1.433 | 0.762 | 0.912 | 1.25E-02 | 4.31E-02 | Up in Noninvoluted |
| **ANK2** | 0.925 | 1.813 | -0.971 | 1.26E-02 | 4.34E-02 | Down in Noninvoluted |
| **STRA6** | 1.602 | 0.269 | 2.576 | 1.28E-02 | 4.35E-02 | Up in Noninvoluted |
| **TMOD1** | 0.687 | 1.480 | -1.107 | 1.27E-02 | 4.35E-02 | Down in Noninvoluted |
| **TTYH2** | 1.397 | 0.749 | 0.900 | 1.28E-02 | 4.35E-02 | Up in Noninvoluted |
| **PPBP** | 0.841 | 1.344 | -0.677 | 1.27E-02 | 4.35E-02 | Down in Noninvoluted |
| **TSPAN15** | 0.955 | 1.394 | -0.546 | 1.27E-02 | 4.35E-02 | Down in Noninvoluted |
| **ZNF137** | 1.991 | 0.243 | 3.036 | 1.28E-02 | 4.35E-02 | Up in Noninvoluted |
| **PTK7** | 1.117 | 0.709 | 0.655 | 1.29E-02 | 4.36E-02 | Up in Noninvoluted |
| **CDCA3** | 1.316 | 0.248 | 2.407 | 1.30E-02 | 4.40E-02 | Up in Noninvoluted |
| **RHOXF1** | 1.319 | 0.415 | 1.669 | 1.33E-02 | 4.46E-02 | Up in Noninvoluted |
| **SDK1** | 1.120 | 0.553 | 1.019 | 1.33E-02 | 4.46E-02 | Up in Noninvoluted |
| **C18ORF45** | 1.089 | 0.812 | 0.424 | 1.33E-02 | 4.46E-02 | Up in Noninvoluted |
| **GM2A** | 1.173 | 0.845 | 0.473 | 1.33E-02 | 4.46E-02 | Up in Noninvoluted |
| **PKD2** | 0.859 | 1.381 | -0.686 | 1.34E-02 | 4.47E-02 | Down in Noninvoluted |
| **PELP1** | 1.243 | 0.680 | 0.871 | 1.34E-02 | 4.49E-02 | Up in Noninvoluted |
| **ZC3H8** | 1.281 | 0.733 | 0.806 | 1.36E-02 | 4.51E-02 | Up in Noninvoluted |
| **JMJD1A** | 0.786 | 1.290 | -0.715 | 1.39E-02 | 4.53E-02 | Down in Noninvoluted |
| **CANT1** | 1.194 | 0.695 | 0.780 | 1.38E-02 | 4.53E-02 | Up in Noninvoluted |
| **SLC26A5** | 1.247 | 0.616 | 1.018 | 1.38E-02 | 4.53E-02 | Up in Noninvoluted |
| **FAT** | 1.099 | 0.804 | 0.452 | 1.38E-02 | 4.53E-02 | Up in Noninvoluted |
| **SMA5** | 1.178 | 0.863 | 0.448 | 1.37E-02 | 4.53E-02 | Up in Noninvoluted |
| **KIAA1279** | 0.875 | 1.231 | -0.493 | 1.38E-02 | 4.53E-02 | Down in Noninvoluted |
| **TMEM165** | 0.904 | 1.292 | -0.515 | 1.39E-02 | 4.53E-02 | Down in Noninvoluted |
| **CDX1** | 3.731 | 0.271 | 3.784 | 1.38E-02 | 4.53E-02 | Up in Noninvoluted |
| **SBDS** | 0.866 | 1.121 | -0.372 | 1.41E-02 | 4.59E-02 | Down in Noninvoluted |
| **RTCD1** | 0.735 | 1.261 | -0.778 | 1.41E-02 | 4.60E-02 | Down in Noninvoluted |
| **SHMT2** | 1.163 | 0.747 | 0.639 | 1.42E-02 | 4.60E-02 | Up in Noninvoluted |
| **ANKRD12** | 0.886 | 1.616 | -0.867 | 1.42E-02 | 4.60E-02 | Down in Noninvoluted |
| **DLG4** | 1.873 | 0.512 | 1.871 | 1.43E-02 | 4.61E-02 | Up in Noninvoluted |
| **C1ORF115** | 0.808 | 1.727 | -1.095 | 1.44E-02 | 4.63E-02 | Down in Noninvoluted |
| **RPLP1** | 1.090 | 0.893 | 0.288 | 1.44E-02 | 4.63E-02 | Up in Noninvoluted |
| **EPS8L1** | 1.178 | 0.510 | 1.207 | 1.46E-02 | 4.63E-02 | Up in Noninvoluted |
| **H2AFY2** | 1.260 | 0.622 | 1.019 | 1.45E-02 | 4.63E-02 | Up in Noninvoluted |
| **PMS2L5** | 0.848 | 1.253 | -0.563 | 1.45E-02 | 4.63E-02 | Down in Noninvoluted |
| **TNFRSF17** | 1.986 | 0.622 | 1.675 | 1.47E-02 | 4.63E-02 | Up in Noninvoluted |
| **MAP4K1** | 2.111 | 0.708 | 1.576 | 1.46E-02 | 4.63E-02 | Up in Noninvoluted |
| **SMAD9** | 0.900 | 1.415 | -0.652 | 1.45E-02 | 4.63E-02 | Down in Noninvoluted |
| **DIO2** | 2.262 | 0.565 | 2.000 | 1.47E-02 | 4.63E-02 | Up in Noninvoluted |
| **TESSP5** | 1.666 | 0.161 | 3.369 | 1.46E-02 | 4.63E-02 | Up in Noninvoluted |
| **AKR1C2** | 0.844 | 1.364 | -0.693 | 1.47E-02 | 4.63E-02 | Down in Noninvoluted |
| **TRIM28** | 1.103 | 0.846 | 0.384 | 1.48E-02 | 4.64E-02 | Up in Noninvoluted |
| **COQ5** | 0.649 | 1.395 | -1.105 | 1.49E-02 | 4.65E-02 | Down in Noninvoluted |
| **BOP1** | 1.307 | 0.688 | 0.925 | 1.49E-02 | 4.65E-02 | Up in Noninvoluted |
| **PAOX** | 1.281 | 0.887 | 0.530 | 1.49E-02 | 4.65E-02 | Up in Noninvoluted |
| **TRPV1** | 1.240 | 0.675 | 0.877 | 1.50E-02 | 4.66E-02 | Up in Noninvoluted |
| **DIAPH3** | 1.742 | 0.662 | 1.395 | 1.50E-02 | 4.66E-02 | Up in Noninvoluted |
| **COX10** | 0.674 | 1.525 | -1.177 | 1.51E-02 | 4.67E-02 | Down in Noninvoluted |
| **PRAM1** | 2.204 | 0.501 | 2.139 | 1.51E-02 | 4.67E-02 | Up in Noninvoluted |
| **RB1** | 0.888 | 1.604 | -0.853 | 1.53E-02 | 4.70E-02 | Down in Noninvoluted |
| **SLCO4A1** | 0.630 | 2.723 | -2.112 | 1.52E-02 | 4.70E-02 | Down in Noninvoluted |
| **CD24** | 1.226 | 0.783 | 0.647 | 1.54E-02 | 4.70E-02 | Up in Noninvoluted |
| **MAPBPIP** | 1.181 | 0.808 | 0.548 | 1.54E-02 | 4.70E-02 | Up in Noninvoluted |
| **YPEL5** | 0.810 | 1.315 | -0.700 | 1.53E-02 | 4.70E-02 | Down in Noninvoluted |
| **HIST1H1B** | 2.054 | 0.363 | 2.500 | 1.54E-02 | 4.70E-02 | Up in Noninvoluted |
| **DDB2** | 1.234 | 0.895 | 0.463 | 1.54E-02 | 4.70E-02 | Up in Noninvoluted |
| **BTN3A2** | 1.088 | 0.667 | 0.707 | 1.56E-02 | 4.72E-02 | Up in Noninvoluted |
| **CXCL10** | 2.099 | 0.422 | 2.315 | 1.55E-02 | 4.72E-02 | Up in Noninvoluted |
| **LARP6** | 0.842 | 1.539 | -0.871 | 1.55E-02 | 4.72E-02 | Down in Noninvoluted |
| **PLAU** | 1.349 | 0.341 | 1.983 | 1.68E-02 | 4.72E-02 | Up in Noninvoluted |
| **PEG10** | 1.370 | 0.718 | 0.932 | 1.67E-02 | 4.72E-02 | Up in Noninvoluted |
| **FKRP** | 1.216 | 0.421 | 1.528 | 1.61E-02 | 4.72E-02 | Up in Noninvoluted |
| **TAS2R3** | 1.213 | 0.500 | 1.280 | 1.56E-02 | 4.72E-02 | Up in Noninvoluted |
| **ERO1LB** | 0.812 | 1.196 | -0.557 | 1.61E-02 | 4.72E-02 | Down in Noninvoluted |
| **UBFD1** | 1.178 | 0.745 | 0.661 | 1.60E-02 | 4.72E-02 | Up in Noninvoluted |
| **CPT1A** | 0.796 | 1.154 | -0.535 | 1.62E-02 | 4.72E-02 | Down in Noninvoluted |
| **RBM35B** | 1.129 | 0.658 | 0.779 | 1.68E-02 | 4.72E-02 | Up in Noninvoluted |
| **ASB6** | 1.097 | 0.752 | 0.544 | 1.58E-02 | 4.72E-02 | Up in Noninvoluted |
| **HNRPH1** | 1.075 | 0.803 | 0.420 | 1.67E-02 | 4.72E-02 | Up in Noninvoluted |
| **LAMC3** | 1.274 | 0.710 | 0.843 | 1.67E-02 | 4.72E-02 | Up in Noninvoluted |
| **LOC144983** | 1.133 | 0.837 | 0.436 | 1.57E-02 | 4.72E-02 | Up in Noninvoluted |
| **CDCA1** | 1.235 | 0.788 | 0.647 | 1.66E-02 | 4.72E-02 | Up in Noninvoluted |
| **KEAP1** | 1.128 | 0.897 | 0.330 | 1.65E-02 | 4.72E-02 | Up in Noninvoluted |
| **PATL1** | 0.769 | 1.207 | -0.651 | 1.64E-02 | 4.72E-02 | Down in Noninvoluted |
| **EIF4E3** | 0.896 | 1.323 | -0.562 | 1.61E-02 | 4.72E-02 | Down in Noninvoluted |
| **STK4** | 0.865 | 1.148 | -0.408 | 1.66E-02 | 4.72E-02 | Down in Noninvoluted |
| **HN1** | 1.185 | 0.905 | 0.389 | 1.63E-02 | 4.72E-02 | Up in Noninvoluted |
| **ZSCAN4** | 3.705 | 0.810 | 2.193 | 1.66E-02 | 4.72E-02 | Up in Noninvoluted |
| **B4GALNT1** | 1.385 | 0.766 | 0.854 | 1.57E-02 | 4.72E-02 | Up in Noninvoluted |
| **G3BP2** | 1.198 | 0.781 | 0.618 | 1.59E-02 | 4.72E-02 | Up in Noninvoluted |
| **S100A16** | 0.913 | 1.193 | -0.387 | 1.68E-02 | 4.72E-02 | Down in Noninvoluted |
| **KCTD20** | 0.803 | 1.647 | -1.036 | 1.68E-02 | 4.72E-02 | Down in Noninvoluted |
| **C14ORF126** | 0.905 | 1.332 | -0.557 | 1.69E-02 | 4.72E-02 | Down in Noninvoluted |
| **CTDSP2** | 0.961 | 1.294 | -0.430 | 1.61E-02 | 4.72E-02 | Down in Noninvoluted |
| **ZNF703** | 1.277 | 0.733 | 0.800 | 1.62E-02 | 4.72E-02 | Up in Noninvoluted |
| **STEAP4** | 0.848 | 1.684 | -0.989 | 1.58E-02 | 4.72E-02 | Down in Noninvoluted |
| **HRASLS** | 3.999 | 0.777 | 2.363 | 1.62E-02 | 4.72E-02 | Up in Noninvoluted |
| **SOX2** | 0.905 | 1.464 | -0.694 | 1.64E-02 | 4.72E-02 | Down in Noninvoluted |
| **MGC29506** | 1.456 | 0.708 | 1.040 | 1.63E-02 | 4.72E-02 | Up in Noninvoluted |
| **C21ORF7** | 0.854 | 2.131 | -1.320 | 1.67E-02 | 4.72E-02 | Down in Noninvoluted |
| **JAK1** | 0.854 | 1.385 | -0.698 | 1.66E-02 | 4.72E-02 | Down in Noninvoluted |
| **ZER1** | 0.817 | 1.449 | -0.827 | 1.63E-02 | 4.72E-02 | Down in Noninvoluted |
| **SH2D3A** | 3.333 | 0.333 | 3.323 | 1.58E-02 | 4.72E-02 | Up in Noninvoluted |
| **C6ORF136** | 0.890 | 1.919 | -1.108 | 1.60E-02 | 4.72E-02 | Down in Noninvoluted |
| **LGR6** | 1.764 | 0.565 | 1.643 | 1.65E-02 | 4.72E-02 | Up in Noninvoluted |
| **CHN2** | 0.888 | 1.782 | -1.004 | 1.58E-02 | 4.72E-02 | Down in Noninvoluted |
| **PRSS16** | 0.754 | 2.421 | -1.683 | 1.64E-02 | 4.72E-02 | Down in Noninvoluted |
| **GSG1L** | 1.110 | 0.600 | 0.888 | 1.70E-02 | 4.73E-02 | Up in Noninvoluted |
| **ITIH5L** | 2.752 | 0.465 | 2.564 | 1.70E-02 | 4.73E-02 | Up in Noninvoluted |
| **ANGPT1** | 0.691 | 1.861 | -1.428 | 1.70E-02 | 4.73E-02 | Down in Noninvoluted |
| **CD302** | 0.812 | 1.213 | -0.579 | 1.70E-02 | 4.73E-02 | Down in Noninvoluted |
| **WDR51A** | 1.117 | 0.445 | 1.329 | 1.74E-02 | 4.79E-02 | Up in Noninvoluted |
| **ZNF211** | 1.335 | 0.514 | 1.375 | 1.74E-02 | 4.79E-02 | Up in Noninvoluted |
| **PPAT** | 1.081 | 0.715 | 0.598 | 1.74E-02 | 4.79E-02 | Up in Noninvoluted |
| **PLSCR4** | 0.799 | 1.199 | -0.587 | 1.73E-02 | 4.79E-02 | Down in Noninvoluted |
| **C4ORF14** | 1.146 | 0.901 | 0.347 | 1.73E-02 | 4.79E-02 | Up in Noninvoluted |
| **BLK** | 2.562 | 0.650 | 1.979 | 1.74E-02 | 4.79E-02 | Up in Noninvoluted |
| **SYT8** | 2.438 | 0.194 | 3.654 | 1.76E-02 | 4.81E-02 | Up in Noninvoluted |
| **ZFYVE16** | 0.855 | 1.224 | -0.517 | 1.76E-02 | 4.82E-02 | Down in Noninvoluted |
| **ARHGEF5** | 1.155 | 0.818 | 0.497 | 1.77E-02 | 4.82E-02 | Up in Noninvoluted |
| **MITF** | 0.768 | 1.397 | -0.863 | 1.77E-02 | 4.82E-02 | Down in Noninvoluted |
| **ALPK2** | 1.827 | 0.526 | 1.795 | 1.78E-02 | 4.84E-02 | Up in Noninvoluted |
| **KIAA1370** | 0.931 | 1.585 | -0.768 | 1.79E-02 | 4.85E-02 | Down in Noninvoluted |
| **LOC90835** | 1.232 | 0.819 | 0.590 | 1.79E-02 | 4.85E-02 | Up in Noninvoluted |
| **PECAM1** | 0.949 | 1.413 | -0.575 | 1.80E-02 | 4.85E-02 | Down in Noninvoluted |
| **FLJ23356** | 1.215 | 0.728 | 0.738 | 1.80E-02 | 4.86E-02 | Up in Noninvoluted |
| **TMEM136** | 1.167 | 0.769 | 0.602 | 1.81E-02 | 4.86E-02 | Up in Noninvoluted |
| **MRC2** | 1.080 | 0.777 | 0.476 | 1.81E-02 | 4.86E-02 | Up in Noninvoluted |
| **GRIPAP1** | 0.677 | 1.229 | -0.860 | 1.81E-02 | 4.86E-02 | Down in Noninvoluted |
| **POLR1C** | 1.437 | 0.833 | 0.787 | 1.86E-02 | 4.86E-02 | Up in Noninvoluted |
| **TYMS** | 1.911 | 0.680 | 1.491 | 1.85E-02 | 4.86E-02 | Up in Noninvoluted |
| **GOLM1** | 1.209 | 0.840 | 0.526 | 1.83E-02 | 4.86E-02 | Up in Noninvoluted |
| **DHX32** | 1.128 | 0.785 | 0.523 | 1.84E-02 | 4.86E-02 | Up in Noninvoluted |
| **UMPS** | 0.699 | 1.608 | -1.201 | 1.86E-02 | 4.86E-02 | Down in Noninvoluted |
| **SRP19** | 1.213 | 0.898 | 0.435 | 1.87E-02 | 4.86E-02 | Up in Noninvoluted |
| **ASNS** | 1.395 | 0.790 | 0.821 | 1.86E-02 | 4.86E-02 | Up in Noninvoluted |
| **SHE** | 0.849 | 1.432 | -0.755 | 1.83E-02 | 4.86E-02 | Down in Noninvoluted |
| **TMEM1** | 0.868 | 1.226 | -0.499 | 1.85E-02 | 4.86E-02 | Down in Noninvoluted |
| **RNF167** | 0.871 | 1.247 | -0.518 | 1.86E-02 | 4.86E-02 | Down in Noninvoluted |
| **LIMCH1** | 0.882 | 1.468 | -0.736 | 1.83E-02 | 4.86E-02 | Down in Noninvoluted |
| **DTX3** | 1.328 | 0.830 | 0.678 | 1.84E-02 | 4.86E-02 | Up in Noninvoluted |
| **DARC** | 0.599 | 1.655 | -1.467 | 1.83E-02 | 4.86E-02 | Down in Noninvoluted |
| **C20ORF160** | 1.058 | 1.882 | -0.830 | 1.87E-02 | 4.86E-02 | Down in Noninvoluted |
| **PNMT** | 6.132 | 0.080 | 6.262 | 1.85E-02 | 4.86E-02 | Up in Noninvoluted |
| **SYCP2L** | 0.741 | 2.424 | -1.710 | 1.86E-02 | 4.86E-02 | Down in Noninvoluted |
| **SPTY2D1** | 1.131 | 0.805 | 0.491 | 1.89E-02 | 4.87E-02 | Up in Noninvoluted |
| **FDX1L** | 2.232 | 0.660 | 1.758 | 1.88E-02 | 4.87E-02 | Up in Noninvoluted |
| **NPNT** | 0.917 | 1.347 | -0.554 | 1.89E-02 | 4.87E-02 | Down in Noninvoluted |
| **IL13RA1** | 0.841 | 1.328 | -0.659 | 1.88E-02 | 4.87E-02 | Down in Noninvoluted |
| **COBLL1** | 0.908 | 1.377 | -0.601 | 1.88E-02 | 4.87E-02 | Down in Noninvoluted |
| **TFPI** | 0.840 | 1.417 | -0.755 | 1.89E-02 | 4.87E-02 | Down in Noninvoluted |
| **HIST1H3G** | 2.681 | 0.487 | 2.460 | 1.90E-02 | 4.88E-02 | Up in Noninvoluted |
| **OSBPL9** | 0.895 | 1.335 | -0.577 | 1.90E-02 | 4.89E-02 | Down in Noninvoluted |
| **RHPN1** | 1.186 | 0.420 | 1.497 | 1.91E-02 | 4.89E-02 | Up in Noninvoluted |
| **ELAVL1** | 0.773 | 1.700 | -1.138 | 1.91E-02 | 4.89E-02 | Down in Noninvoluted |
| **FGFR1OP2** | 0.869 | 1.085 | -0.321 | 1.92E-02 | 4.90E-02 | Down in Noninvoluted |
| **LOC57228** | 1.194 | 0.826 | 0.533 | 1.92E-02 | 4.90E-02 | Up in Noninvoluted |
| **GPBAR1** | 0.842 | 1.510 | -0.843 | 1.93E-02 | 4.92E-02 | Down in Noninvoluted |
| **SOX18** | 0.705 | 1.412 | -1.001 | 1.96E-02 | 4.93E-02 | Down in Noninvoluted |
| **KCNK12** | 1.413 | 0.298 | 2.246 | 1.99E-02 | 4.93E-02 | Up in Noninvoluted |
| **SPINT1** | 1.206 | 0.627 | 0.944 | 1.96E-02 | 4.93E-02 | Up in Noninvoluted |
| **IFT74** | 1.172 | 0.744 | 0.657 | 1.98E-02 | 4.93E-02 | Up in Noninvoluted |
| **HIST1H1D** | 1.131 | 0.748 | 0.598 | 1.97E-02 | 4.93E-02 | Up in Noninvoluted |
| **KCNMB2** | 1.505 | 0.608 | 1.307 | 1.95E-02 | 4.93E-02 | Up in Noninvoluted |
| **FZD1** | 1.113 | 0.798 | 0.479 | 1.97E-02 | 4.93E-02 | Up in Noninvoluted |
| **RHOV** | 1.218 | 0.829 | 0.556 | 1.95E-02 | 4.93E-02 | Up in Noninvoluted |
| **NPPC** | 2.205 | 0.773 | 1.512 | 1.98E-02 | 4.93E-02 | Up in Noninvoluted |
| **PARP14** | 0.924 | 1.216 | -0.395 | 1.98E-02 | 4.93E-02 | Down in Noninvoluted |
| **IKBKE** | 2.607 | 0.745 | 1.807 | 1.98E-02 | 4.93E-02 | Up in Noninvoluted |
| **UBE4A** | 0.785 | 1.633 | -1.055 | 1.98E-02 | 4.93E-02 | Down in Noninvoluted |
| **CXCR7** | 0.815 | 2.155 | -1.403 | 1.99E-02 | 4.93E-02 | Down in Noninvoluted |
| **FAM64A** | 2.393 | 0.212 | 3.494 | 1.96E-02 | 4.93E-02 | Up in Noninvoluted |
| **RASSF6** | 1.184 | 0.670 | 0.821 | 1.99E-02 | 4.93E-02 | Up in Noninvoluted |
| **NUP54** | 1.201 | 0.842 | 0.513 | 1.99E-02 | 4.93E-02 | Up in Noninvoluted |
| **DVL3** | 0.887 | 1.188 | -0.421 | 2.00E-02 | 4.94E-02 | Down in Noninvoluted |
| **COL7A1** | 1.219 | 0.742 | 0.717 | 2.02E-02 | 4.96E-02 | Up in Noninvoluted |
| **CRYGS** | 1.381 | 0.738 | 0.905 | 2.02E-02 | 4.96E-02 | Up in Noninvoluted |
| **MYO1B** | 0.797 | 1.081 | -0.439 | 2.03E-02 | 4.97E-02 | Down in Noninvoluted |
| **GALNS** | 1.157 | 0.895 | 0.370 | 2.04E-02 | 4.99E-02 | Up in Noninvoluted |
| **CLCC1** | 1.220 | 0.793 | 0.622 | 2.06E-02 | 5.01E-02 | Not significant |
| **C2ORF37** | 1.365 | 0.880 | 0.633 | 2.05E-02 | 5.01E-02 | Not significant |
| **RPL21** | 1.155 | 0.907 | 0.349 | 2.06E-02 | 5.01E-02 | Not significant |
| **POU4F1** | 5.446 | 0.814 | 2.742 | 2.07E-02 | 5.03E-02 | Not significant |
| **NHP2L1** | 1.124 | 0.854 | 0.396 | 2.07E-02 | 5.03E-02 | Not significant |
| **WDR74** | 1.167 | 0.794 | 0.556 | 2.07E-02 | 5.03E-02 | Not significant |
| **INADL** | 1.160 | 0.859 | 0.434 | 2.08E-02 | 5.03E-02 | Not significant |
| **DDHD1** | 0.842 | 1.176 | -0.481 | 2.08E-02 | 5.03E-02 | Not significant |
| **NR1D1** | 0.866 | 1.643 | -0.924 | 2.10E-02 | 5.06E-02 | Not significant |
| **ZNF460** | 1.001 | 1.642 | -0.714 | 2.10E-02 | 5.06E-02 | Not significant |
| **NUP93** | 1.223 | 0.689 | 0.829 | 2.11E-02 | 5.06E-02 | Not significant |
| **SWAP70** | 0.796 | 1.302 | -0.710 | 2.10E-02 | 5.06E-02 | Not significant |
| **RNF12** | 1.159 | 0.810 | 0.516 | 2.12E-02 | 5.07E-02 | Not significant |
| **SERINC5** | 1.360 | 0.757 | 0.846 | 2.13E-02 | 5.07E-02 | Not significant |
| **UCKL1** | 0.841 | 1.436 | -0.771 | 2.13E-02 | 5.07E-02 | Not significant |
| **ARHGEF6** | 0.956 | 1.387 | -0.537 | 2.13E-02 | 5.07E-02 | Not significant |
| **RAB31** | 0.797 | 1.543 | -0.954 | 2.13E-02 | 5.07E-02 | Not significant |
| **RPP30** | 2.275 | 0.476 | 2.255 | 2.14E-02 | 5.07E-02 | Not significant |
| **DUSP15** | 0.697 | 2.612 | -1.906 | 2.13E-02 | 5.07E-02 | Not significant |
| **DACT1** | 0.859 | 1.211 | -0.495 | 2.14E-02 | 5.08E-02 | Not significant |
| **RUNX3** | 1.077 | 0.674 | 0.675 | 2.15E-02 | 5.08E-02 | Not significant |
| **SLC5A6** | 1.090 | 0.720 | 0.597 | 2.15E-02 | 5.08E-02 | Not significant |
| **TNC** | 1.158 | 0.842 | 0.460 | 2.16E-02 | 5.09E-02 | Not significant |
| **ZAK** | 0.873 | 1.301 | -0.575 | 2.16E-02 | 5.09E-02 | Not significant |
| **WAC** | 0.879 | 1.273 | -0.534 | 2.17E-02 | 5.09E-02 | Not significant |
| **SLC12A2** | 1.142 | 0.738 | 0.629 | 2.17E-02 | 5.10E-02 | Not significant |
| **ACTR3** | 1.178 | 0.809 | 0.543 | 2.18E-02 | 5.11E-02 | Not significant |
| **ANTXR2** | 0.808 | 1.457 | -0.850 | 2.19E-02 | 5.11E-02 | Not significant |
| **KLF13** | 1.173 | 0.672 | 0.804 | 2.20E-02 | 5.13E-02 | Not significant |
| **SCYE1** | 1.221 | 0.746 | 0.711 | 2.20E-02 | 5.13E-02 | Not significant |
| **ZNF167** | 0.741 | 1.200 | -0.695 | 2.20E-02 | 5.13E-02 | Not significant |
| **HSN2** | 0.883 | 1.146 | -0.375 | 2.22E-02 | 5.15E-02 | Not significant |
| **NUAK1** | 0.748 | 1.888 | -1.336 | 2.23E-02 | 5.17E-02 | Not significant |
| **DHRS7** | 0.912 | 1.124 | -0.301 | 2.24E-02 | 5.18E-02 | Not significant |
| **DNA2** | 1.410 | 0.640 | 1.139 | 2.25E-02 | 5.18E-02 | Not significant |
| **DAZAP2** | 0.934 | 1.099 | -0.234 | 2.24E-02 | 5.18E-02 | Not significant |
| **CD34** | 0.918 | 1.387 | -0.596 | 2.25E-02 | 5.18E-02 | Not significant |
| **GPR35** | 2.237 | 0.435 | 2.364 | 2.25E-02 | 5.18E-02 | Not significant |
| **SCARNA15** | 1.210 | 0.771 | 0.651 | 2.26E-02 | 5.20E-02 | Not significant |
| **APCDD1L** | 1.334 | 0.838 | 0.670 | 2.27E-02 | 5.20E-02 | Not significant |
| **GPRC5A** | 0.927 | 1.345 | -0.537 | 2.27E-02 | 5.20E-02 | Not significant |
| **C1GALT1C1** | 1.062 | 0.790 | 0.426 | 2.30E-02 | 5.23E-02 | Not significant |
| **PLS3** | 0.804 | 1.468 | -0.868 | 2.29E-02 | 5.23E-02 | Not significant |
| **C5ORF32** | 0.834 | 1.742 | -1.063 | 2.29E-02 | 5.23E-02 | Not significant |
| **THOC3** | 1.156 | 0.837 | 0.466 | 2.31E-02 | 5.24E-02 | Not significant |
| **ZNF441** | 0.658 | 1.770 | -1.427 | 2.31E-02 | 5.25E-02 | Not significant |
| **PLVAP** | 0.878 | 1.257 | -0.517 | 2.32E-02 | 5.26E-02 | Not significant |
| **GOLGB1** | 1.056 | 0.730 | 0.532 | 2.34E-02 | 5.28E-02 | Not significant |
| **INSIG2** | 0.839 | 1.624 | -0.954 | 2.35E-02 | 5.31E-02 | Not significant |
| **MLLT11** | 2.120 | 0.374 | 2.503 | 2.36E-02 | 5.32E-02 | Not significant |
| **TTL** | 1.145 | 0.765 | 0.581 | 2.38E-02 | 5.33E-02 | Not significant |
| **ROR1** | 1.239 | 0.816 | 0.603 | 2.38E-02 | 5.33E-02 | Not significant |
| **RCBTB1** | 0.939 | 1.320 | -0.491 | 2.38E-02 | 5.33E-02 | Not significant |
| **GTSE1** | 8.295 | 0.638 | 3.699 | 2.38E-02 | 5.33E-02 | Not significant |
| **SERTAD4** | 0.862 | 1.687 | -0.969 | 2.37E-02 | 5.33E-02 | Not significant |
| **C12ORF35** | 0.920 | 1.177 | -0.355 | 2.39E-02 | 5.34E-02 | Not significant |
| **POT1** | 0.883 | 1.440 | -0.705 | 2.40E-02 | 5.36E-02 | Not significant |
| **IFITM4P** | 0.851 | 1.690 | -0.990 | 2.42E-02 | 5.40E-02 | Not significant |
| **GLCCI1** | 0.831 | 1.401 | -0.753 | 2.44E-02 | 5.42E-02 | Not significant |
| **SEPW1** | 0.863 | 1.656 | -0.940 | 2.44E-02 | 5.42E-02 | Not significant |
| **CCT4** | 1.259 | 0.859 | 0.552 | 2.46E-02 | 5.44E-02 | Not significant |
| **MED24** | 1.155 | 0.809 | 0.514 | 2.47E-02 | 5.45E-02 | Not significant |
| **DKFZP564O0523** | 0.779 | 1.145 | -0.556 | 2.47E-02 | 5.45E-02 | Not significant |
| **ZMYND10** | 2.473 | 0.605 | 2.031 | 2.47E-02 | 5.45E-02 | Not significant |
| **VAMP1** | 0.859 | 1.328 | -0.628 | 2.49E-02 | 5.47E-02 | Not significant |
| **FICD** | 0.873 | 1.128 | -0.370 | 2.49E-02 | 5.49E-02 | Not significant |
| **CENPM** | 1.967 | 0.469 | 2.068 | 2.50E-02 | 5.49E-02 | Not significant |
| **LRRC61** | 2.172 | 0.520 | 2.063 | 2.51E-02 | 5.49E-02 | Not significant |
| **SP6** | 1.506 | 0.730 | 1.045 | 2.51E-02 | 5.50E-02 | Not significant |
| **SMARCA4** | 1.123 | 0.837 | 0.424 | 2.58E-02 | 5.50E-02 | Not significant |
| **HIST1H3F** | 1.204 | 0.519 | 1.215 | 2.57E-02 | 5.50E-02 | Not significant |
| **CDCA7L** | 1.197 | 0.791 | 0.598 | 2.55E-02 | 5.50E-02 | Not significant |
| **SF3B14** | 1.101 | 0.822 | 0.423 | 2.54E-02 | 5.50E-02 | Not significant |
| **C1ORF61** | 1.561 | 0.313 | 2.318 | 2.54E-02 | 5.50E-02 | Not significant |
| **CEP290** | 1.236 | 0.889 | 0.475 | 2.55E-02 | 5.50E-02 | Not significant |
| **LOC388524** | 1.122 | 0.868 | 0.370 | 2.57E-02 | 5.50E-02 | Not significant |
| **DNASE2B** | 1.249 | 0.839 | 0.574 | 2.54E-02 | 5.50E-02 | Not significant |
| **KIAA0256** | 0.859 | 1.210 | -0.494 | 2.58E-02 | 5.50E-02 | Not significant |
| **DDX58** | 0.696 | 1.268 | -0.867 | 2.57E-02 | 5.50E-02 | Not significant |
| **CLCN4** | 0.639 | 1.682 | -1.396 | 2.57E-02 | 5.50E-02 | Not significant |
| **RASGRP4** | 0.860 | 1.530 | -0.831 | 2.56E-02 | 5.50E-02 | Not significant |
| **C6ORF174** | 1.894 | 0.299 | 2.663 | 2.55E-02 | 5.50E-02 | Not significant |
| **FBLN2** | 0.880 | 1.547 | -0.815 | 2.56E-02 | 5.50E-02 | Not significant |
| **HOXB8** | 1.013 | 2.762 | -1.447 | 2.52E-02 | 5.50E-02 | Not significant |
| **TOE1** | 1.729 | 0.586 | 1.561 | 2.53E-02 | 5.50E-02 | Not significant |
| **CYP24A1** | 5.050 | 0.556 | 3.182 | 2.55E-02 | 5.50E-02 | Not significant |
| **CEACAM1** | 1.273 | 0.864 | 0.559 | 2.59E-02 | 5.52E-02 | Not significant |
| **CUTC** | 0.624 | 1.199 | -0.943 | 2.60E-02 | 5.52E-02 | Not significant |
| **C14ORF43** | 0.859 | 1.229 | -0.517 | 2.60E-02 | 5.52E-02 | Not significant |
| **TMTC2** | 2.017 | 0.846 | 1.253 | 2.62E-02 | 5.55E-02 | Not significant |
| **CD248** | 1.151 | 0.770 | 0.580 | 2.65E-02 | 5.59E-02 | Not significant |
| **ADA** | 0.793 | 2.325 | -1.551 | 2.65E-02 | 5.59E-02 | Not significant |
| **ARNT** | 0.878 | 1.304 | -0.571 | 2.64E-02 | 5.59E-02 | Not significant |
| **TSHZ2** | 1.091 | 0.788 | 0.470 | 2.66E-02 | 5.60E-02 | Not significant |
| **ZCCHC24** | 0.862 | 1.403 | -0.703 | 2.66E-02 | 5.60E-02 | Not significant |
| **TBL1XR1** | 0.886 | 1.134 | -0.356 | 2.68E-02 | 5.62E-02 | Not significant |
| **CXCL6** | 1.728 | 0.664 | 1.380 | 2.68E-02 | 5.62E-02 | Not significant |
| **MND1** | 2.454 | 0.527 | 2.220 | 2.68E-02 | 5.62E-02 | Not significant |
| **IGSF10** | 0.817 | 1.385 | -0.761 | 2.70E-02 | 5.64E-02 | Not significant |
| **APLN** | 5.979 | 0.720 | 3.054 | 2.70E-02 | 5.64E-02 | Not significant |
| **MAD2L1** | 1.443 | 0.807 | 0.839 | 2.70E-02 | 5.64E-02 | Not significant |
| **ELF4** | 0.876 | 1.574 | -0.846 | 2.71E-02 | 5.65E-02 | Not significant |
| **LPCAT3** | 0.932 | 1.211 | -0.377 | 2.74E-02 | 5.67E-02 | Not significant |
| **NDN** | 0.855 | 1.322 | -0.629 | 2.73E-02 | 5.67E-02 | Not significant |
| **C3ORF42** | 0.921 | 2.261 | -1.295 | 2.73E-02 | 5.67E-02 | Not significant |
| **FLJ10213** | 1.089 | 0.730 | 0.577 | 2.82E-02 | 5.68E-02 | Not significant |
| **KIAA0649** | 1.161 | 0.622 | 0.901 | 2.78E-02 | 5.68E-02 | Not significant |
| **GNA11** | 1.097 | 0.710 | 0.628 | 2.83E-02 | 5.68E-02 | Not significant |
| **UBAC2** | 1.247 | 0.851 | 0.551 | 2.83E-02 | 5.68E-02 | Not significant |
| **IDUA** | 1.122 | 0.804 | 0.480 | 2.78E-02 | 5.68E-02 | Not significant |
| **SLC23A3** | 1.431 | 0.480 | 1.577 | 2.83E-02 | 5.68E-02 | Not significant |
| **CCT2** | 1.159 | 0.902 | 0.361 | 2.80E-02 | 5.68E-02 | Not significant |
| **RPS15A** | 1.104 | 0.886 | 0.316 | 2.76E-02 | 5.68E-02 | Not significant |
| **KRTCAP3** | 1.245 | 0.832 | 0.582 | 2.82E-02 | 5.68E-02 | Not significant |
| **PKIB** | 1.099 | 0.839 | 0.389 | 2.77E-02 | 5.68E-02 | Not significant |
| **ARHGAP17** | 0.945 | 1.201 | -0.346 | 2.81E-02 | 5.68E-02 | Not significant |
| **IFNG** | 1.341 | 0.808 | 0.730 | 2.82E-02 | 5.68E-02 | Not significant |
| **F3** | 0.743 | 2.007 | -1.434 | 2.78E-02 | 5.68E-02 | Not significant |
| **TBC1D9** | 0.952 | 1.172 | -0.301 | 2.81E-02 | 5.68E-02 | Not significant |
| **HCG4** | 2.368 | 0.782 | 1.599 | 2.83E-02 | 5.68E-02 | Not significant |
| **TMEM116** | 0.883 | 1.355 | -0.618 | 2.77E-02 | 5.68E-02 | Not significant |
| **BPHL** | 0.904 | 1.330 | -0.556 | 2.82E-02 | 5.68E-02 | Not significant |
| **HSPC171** | 1.470 | 0.434 | 1.759 | 2.78E-02 | 5.68E-02 | Not significant |
| **FAM83D** | 4.071 | 0.475 | 3.099 | 2.82E-02 | 5.68E-02 | Not significant |
| **SH3BGR** | 2.726 | 0.661 | 2.044 | 2.75E-02 | 5.68E-02 | Not significant |
| **CCDC3** | 0.749 | 1.543 | -1.042 | 2.83E-02 | 5.68E-02 | Not significant |
| **RPL23AP13** | 0.868 | 2.276 | -1.390 | 2.83E-02 | 5.68E-02 | Not significant |
| **LCT** | 2.318 | 0.273 | 3.087 | 2.81E-02 | 5.68E-02 | Not significant |
| **G6PC3** | 0.919 | 1.290 | -0.490 | 2.85E-02 | 5.70E-02 | Not significant |
| **TSLP** | 0.830 | 1.616 | -0.962 | 2.86E-02 | 5.71E-02 | Not significant |
| **THSD1** | 0.864 | 1.253 | -0.537 | 2.89E-02 | 5.73E-02 | Not significant |
| **MGST2** | 0.839 | 1.467 | -0.806 | 2.88E-02 | 5.73E-02 | Not significant |
| **MAP1LC3B** | 0.878 | 1.533 | -0.804 | 2.89E-02 | 5.73E-02 | Not significant |
| **LEPR** | 0.894 | 1.988 | -1.153 | 2.89E-02 | 5.73E-02 | Not significant |
| **SLC34A2** | 1.555 | 0.615 | 1.338 | 2.87E-02 | 5.73E-02 | Not significant |
| **CLASP1** | 0.817 | 1.789 | -1.130 | 2.88E-02 | 5.73E-02 | Not significant |
| **PCOLCE2** | 1.175 | 9.160 | -2.963 | 2.89E-02 | 5.73E-02 | Not significant |
| **KLHL9** | 1.101 | 0.873 | 0.335 | 2.93E-02 | 5.80E-02 | Not significant |
| **NBN** | 1.435 | 0.770 | 0.897 | 2.94E-02 | 5.81E-02 | Not significant |
| **PCDH11X** | 2.398 | 0.653 | 1.876 | 2.95E-02 | 5.81E-02 | Not significant |
| **COL4A6** | 2.202 | 0.378 | 2.545 | 2.97E-02 | 5.84E-02 | Not significant |
| **RGS5** | 0.844 | 1.288 | -0.609 | 2.98E-02 | 5.87E-02 | Not significant |
| **CCDC55** | 1.288 | 0.795 | 0.696 | 2.99E-02 | 5.87E-02 | Not significant |
| **LEFTY2** | 0.796 | 2.451 | -1.623 | 3.00E-02 | 5.87E-02 | Not significant |
| **SNTG2** | 1.044 | 13.600 | -3.703 | 3.00E-02 | 5.87E-02 | Not significant |
| **CHAD** | 0.782 | 1.667 | -1.092 | 3.02E-02 | 5.90E-02 | Not significant |
| **HOXD3** | 0.846 | 1.415 | -0.742 | 3.02E-02 | 5.90E-02 | Not significant |
| **RY1** | 0.741 | 1.604 | -1.114 | 3.03E-02 | 5.90E-02 | Not significant |
| **LOC400464** | 0.861 | 1.846 | -1.100 | 3.03E-02 | 5.91E-02 | Not significant |
| **HDAC2** | 1.157 | 0.875 | 0.402 | 3.05E-02 | 5.93E-02 | Not significant |
| **HSPA2** | 0.807 | 1.195 | -0.566 | 3.05E-02 | 5.93E-02 | Not significant |
| **B3GALT6** | 1.214 | 0.683 | 0.829 | 3.09E-02 | 5.93E-02 | Not significant |
| **PDLIM7** | 1.137 | 0.810 | 0.488 | 3.09E-02 | 5.93E-02 | Not significant |
| **ALG3** | 1.089 | 0.797 | 0.451 | 3.09E-02 | 5.93E-02 | Not significant |
| **NACA** | 1.096 | 0.883 | 0.311 | 3.07E-02 | 5.93E-02 | Not significant |
| **CXCL13** | 2.375 | 0.559 | 2.086 | 3.08E-02 | 5.93E-02 | Not significant |
| **DISP2** | 5.298 | 0.813 | 2.704 | 3.09E-02 | 5.93E-02 | Not significant |
| **FLYWCH1** | 0.882 | 1.303 | -0.562 | 3.10E-02 | 5.93E-02 | Not significant |
| **HMMR** | 1.925 | 0.598 | 1.688 | 3.09E-02 | 5.93E-02 | Not significant |
| **RAMP2** | 0.827 | 1.610 | -0.961 | 3.06E-02 | 5.93E-02 | Not significant |
| **NMUR1** | 0.574 | 2.671 | -2.218 | 3.06E-02 | 5.93E-02 | Not significant |
| **HIST1H3B** | 1.568 | 0.300 | 2.384 | 3.14E-02 | 5.93E-02 | Not significant |
| **CACNG4** | 1.583 | 0.828 | 0.935 | 3.14E-02 | 5.93E-02 | Not significant |
| **ATP5J** | 0.850 | 1.197 | -0.493 | 3.14E-02 | 5.93E-02 | Not significant |
| **CHGA** | 2.556 | 0.678 | 1.914 | 3.11E-02 | 5.93E-02 | Not significant |
| **CKAP2L** | 14.880 | 0.822 | 4.179 | 3.13E-02 | 5.93E-02 | Not significant |
| **OCEL1** | 0.839 | 1.232 | -0.554 | 3.12E-02 | 5.93E-02 | Not significant |
| **KIF18B** | 3.072 | 0.742 | 2.051 | 3.11E-02 | 5.93E-02 | Not significant |
| **UFSP1** | 2.172 | 0.488 | 2.154 | 3.10E-02 | 5.93E-02 | Not significant |
| **KIFC1** | 9.405 | 0.660 | 3.833 | 3.12E-02 | 5.93E-02 | Not significant |
| **EDG7** | 3.158 | 0.332 | 3.248 | 3.13E-02 | 5.93E-02 | Not significant |
| **ZBTB1** | 1.137 | 0.781 | 0.542 | 3.18E-02 | 5.95E-02 | Not significant |
| **WDR4** | 1.157 | 0.871 | 0.410 | 3.17E-02 | 5.95E-02 | Not significant |
| **COX6B2** | 1.747 | 0.736 | 1.248 | 3.18E-02 | 5.95E-02 | Not significant |
| **MCCC2** | 0.872 | 1.209 | -0.471 | 3.18E-02 | 5.95E-02 | Not significant |
| **HLA-A** | 0.933 | 1.244 | -0.415 | 3.16E-02 | 5.95E-02 | Not significant |
| **KANK2** | 0.696 | 1.306 | -0.909 | 3.18E-02 | 5.95E-02 | Not significant |
| **HS6ST2** | 2.094 | 0.447 | 2.227 | 3.18E-02 | 5.95E-02 | Not significant |
| **ASPRV1** | 1.289 | 3.967 | -1.621 | 3.16E-02 | 5.95E-02 | Not significant |
| **MCM7** | 1.166 | 0.879 | 0.407 | 3.21E-02 | 5.95E-02 | Not significant |
| **FGF2** | 0.890 | 1.355 | -0.607 | 3.19E-02 | 5.95E-02 | Not significant |
| **C1ORF21** | 0.935 | 1.323 | -0.501 | 3.21E-02 | 5.95E-02 | Not significant |
| **ZXDB** | 0.817 | 1.787 | -1.130 | 3.21E-02 | 5.95E-02 | Not significant |
| **ZC3H12B** | 0.767 | 1.432 | -0.901 | 3.19E-02 | 5.95E-02 | Not significant |
| **PHKB** | 0.872 | 1.374 | -0.657 | 3.21E-02 | 5.95E-02 | Not significant |
| **PRO1853** | 1.197 | 0.899 | 0.413 | 3.23E-02 | 5.97E-02 | Not significant |
| **RIT1** | 0.842 | 1.368 | -0.700 | 3.23E-02 | 5.97E-02 | Not significant |
| **SIRT6** | 5.811 | 0.749 | 2.956 | 3.24E-02 | 5.97E-02 | Not significant |
| **IGFL3** | 0.861 | 2.719 | -1.660 | 3.23E-02 | 5.97E-02 | Not significant |
| **PTPRB** | 0.848 | 2.043 | -1.269 | 3.24E-02 | 5.97E-02 | Not significant |
| **ZNF3** | 1.076 | 0.876 | 0.296 | 3.27E-02 | 6.01E-02 | Not significant |
| **CDH4** | 2.458 | 0.857 | 1.520 | 3.27E-02 | 6.01E-02 | Not significant |
| **FAM62C** | 5.504 | 0.767 | 2.844 | 3.27E-02 | 6.01E-02 | Not significant |
| **C18ORF22** | 1.103 | 0.754 | 0.549 | 3.30E-02 | 6.03E-02 | Not significant |
| **UBD** | 1.624 | 0.673 | 1.272 | 3.30E-02 | 6.03E-02 | Not significant |
| **LIPG** | 6.247 | 0.635 | 3.297 | 3.31E-02 | 6.03E-02 | Not significant |
| **RAD54L** | 1.389 | 0.739 | 0.910 | 3.30E-02 | 6.03E-02 | Not significant |
| **JAK3** | 0.820 | 1.607 | -0.971 | 3.31E-02 | 6.03E-02 | Not significant |
| **C11ORF67** | 0.723 | 2.544 | -1.815 | 3.30E-02 | 6.03E-02 | Not significant |
| **ZFHX4** | 0.805 | 1.342 | -0.737 | 3.33E-02 | 6.05E-02 | Not significant |
| **PREPL** | 0.937 | 1.243 | -0.408 | 3.34E-02 | 6.06E-02 | Not significant |
| **C15ORF44** | 1.056 | 0.863 | 0.291 | 3.34E-02 | 6.06E-02 | Not significant |
| **WNT4** | 2.159 | 0.322 | 2.744 | 3.35E-02 | 6.06E-02 | Not significant |
| **CPNE8** | 0.829 | 1.495 | -0.850 | 3.35E-02 | 6.07E-02 | Not significant |
| **MAPK6** | 0.814 | 1.299 | -0.674 | 3.36E-02 | 6.07E-02 | Not significant |
| **ALDH3B1** | 2.421 | 0.766 | 1.661 | 3.36E-02 | 6.07E-02 | Not significant |
| **FHL1** | 0.946 | 1.699 | -0.845 | 3.37E-02 | 6.07E-02 | Not significant |
| **GPR12** | 11.580 | 0.666 | 4.120 | 3.37E-02 | 6.07E-02 | Not significant |
| **SVEP1** | 0.746 | 2.112 | -1.502 | 3.38E-02 | 6.08E-02 | Not significant |
| **ZNF10** | 1.097 | 0.827 | 0.407 | 3.41E-02 | 6.08E-02 | Not significant |
| **CDH5** | 0.856 | 1.466 | -0.777 | 3.40E-02 | 6.08E-02 | Not significant |
| **CCNYL1** | 0.733 | 1.467 | -1.001 | 3.40E-02 | 6.08E-02 | Not significant |
| **OTOF** | 4.330 | 0.832 | 2.380 | 3.42E-02 | 6.08E-02 | Not significant |
| **PELI3** | 0.879 | 1.228 | -0.483 | 3.43E-02 | 6.08E-02 | Not significant |
| **TMEM209** | 0.929 | 1.214 | -0.386 | 3.43E-02 | 6.08E-02 | Not significant |
| **OPHN1** | 0.885 | 1.111 | -0.327 | 3.41E-02 | 6.08E-02 | Not significant |
| **RICTOR** | 0.993 | 1.481 | -0.576 | 3.43E-02 | 6.08E-02 | Not significant |
| **FAM80B** | 0.601 | 2.074 | -1.786 | 3.40E-02 | 6.08E-02 | Not significant |
| **PDE4DIP** | 0.845 | 1.343 | -0.669 | 3.42E-02 | 6.08E-02 | Not significant |
| **MMRN2** | 0.874 | 1.572 | -0.848 | 3.43E-02 | 6.08E-02 | Not significant |
| **NEK2** | 1.692 | 0.601 | 1.493 | 3.40E-02 | 6.08E-02 | Not significant |
| **SLCO1A2** | 1.496 | 0.797 | 0.909 | 3.44E-02 | 6.08E-02 | Not significant |
| **SFMBT2** | 1.249 | 0.843 | 0.567 | 3.47E-02 | 6.08E-02 | Not significant |
| **MPI** | 1.417 | 0.692 | 1.034 | 3.45E-02 | 6.08E-02 | Not significant |
| **SCG3** | 0.801 | 1.137 | -0.505 | 3.46E-02 | 6.08E-02 | Not significant |
| **UCP2** | 0.904 | 1.562 | -0.789 | 3.45E-02 | 6.08E-02 | Not significant |
| **LOC729021** | 0.928 | 1.240 | -0.418 | 3.46E-02 | 6.08E-02 | Not significant |
| **FASLG** | 1.522 | 0.635 | 1.260 | 3.45E-02 | 6.08E-02 | Not significant |
| **VEGFC** | 0.832 | 2.737 | -1.718 | 3.46E-02 | 6.08E-02 | Not significant |
| **CST6** | 3.877 | 0.877 | 2.145 | 3.52E-02 | 6.10E-02 | Not significant |
| **XPO5** | 1.084 | 0.837 | 0.374 | 3.53E-02 | 6.10E-02 | Not significant |
| **DJ222E13.2** | 1.139 | 0.763 | 0.577 | 3.50E-02 | 6.10E-02 | Not significant |
| **CDH12** | 3.817 | 0.811 | 2.235 | 3.51E-02 | 6.10E-02 | Not significant |
| **TESSP2** | 2.323 | 0.748 | 1.636 | 3.50E-02 | 6.10E-02 | Not significant |
| **MTERFD2** | 1.314 | 0.799 | 0.718 | 3.52E-02 | 6.10E-02 | Not significant |
| **HIST1H2BB** | 1.496 | 0.534 | 1.486 | 3.49E-02 | 6.10E-02 | Not significant |
| **RBAK** | 0.931 | 1.464 | -0.653 | 3.52E-02 | 6.10E-02 | Not significant |
| **EXOC8** | 0.870 | 1.222 | -0.489 | 3.51E-02 | 6.10E-02 | Not significant |
| **LRRC8B** | 0.896 | 1.875 | -1.065 | 3.52E-02 | 6.10E-02 | Not significant |
| **IGF2AS** | 1.519 | 0.818 | 0.894 | 3.51E-02 | 6.10E-02 | Not significant |
| **FAM21A** | 0.598 | 3.494 | -2.547 | 3.49E-02 | 6.10E-02 | Not significant |
| **GALNTL2** | 0.760 | 1.940 | -1.352 | 3.54E-02 | 6.10E-02 | Not significant |
| **DGKD** | 1.124 | 0.881 | 0.352 | 3.54E-02 | 6.11E-02 | Not significant |
| **RPL31** | 1.063 | 0.772 | 0.462 | 3.55E-02 | 6.11E-02 | Not significant |
| **PHF17** | 0.910 | 1.307 | -0.521 | 3.55E-02 | 6.11E-02 | Not significant |
| **BMPR2** | 0.808 | 1.174 | -0.539 | 3.60E-02 | 6.16E-02 | Not significant |
| **CNIH2** | 3.224 | 0.824 | 1.968 | 3.60E-02 | 6.16E-02 | Not significant |
| **RASAL1** | 3.424 | 0.821 | 2.060 | 3.59E-02 | 6.16E-02 | Not significant |
| **ELOVL5** | 0.818 | 1.845 | -1.174 | 3.60E-02 | 6.16E-02 | Not significant |
| **RXRG** | 0.795 | 1.915 | -1.269 | 3.60E-02 | 6.16E-02 | Not significant |
| **CELSR2** | 1.229 | 0.756 | 0.701 | 3.63E-02 | 6.17E-02 | Not significant |
| **C1ORF168** | 4.151 | 0.840 | 2.305 | 3.67E-02 | 6.17E-02 | Not significant |
| **IMPDH2** | 1.111 | 0.810 | 0.457 | 3.65E-02 | 6.17E-02 | Not significant |
| **ZGPAT** | 1.357 | 0.910 | 0.576 | 3.62E-02 | 6.17E-02 | Not significant |
| **LOC387856** | 1.276 | 0.782 | 0.706 | 3.64E-02 | 6.17E-02 | Not significant |
| **FEZ2** | 0.889 | 1.231 | -0.469 | 3.62E-02 | 6.17E-02 | Not significant |
| **GAB1** | 0.917 | 1.186 | -0.371 | 3.66E-02 | 6.17E-02 | Not significant |
| **RBMS1** | 0.940 | 1.201 | -0.354 | 3.67E-02 | 6.17E-02 | Not significant |
| **ITGA1** | 0.903 | 1.313 | -0.540 | 3.65E-02 | 6.17E-02 | Not significant |
| **EVC2** | 0.956 | 1.408 | -0.559 | 3.67E-02 | 6.17E-02 | Not significant |
| **ZDHHC23** | 1.282 | 0.855 | 0.584 | 3.67E-02 | 6.17E-02 | Not significant |
| **EEF2K** | 0.850 | 1.359 | -0.677 | 3.64E-02 | 6.17E-02 | Not significant |
| **KIAA1324L** | 0.794 | 1.440 | -0.858 | 3.66E-02 | 6.17E-02 | Not significant |
| **LIN7A** | 0.781 | 1.654 | -1.083 | 3.64E-02 | 6.17E-02 | Not significant |
| **TNFRSF8** | 4.657 | 0.892 | 2.384 | 3.69E-02 | 6.19E-02 | Not significant |
| **NOS3** | 0.723 | 1.684 | -1.219 | 3.70E-02 | 6.19E-02 | Not significant |
| **DTWD1** | 0.906 | 1.113 | -0.298 | 3.70E-02 | 6.19E-02 | Not significant |
| **H19** | 0.921 | 1.688 | -0.873 | 3.70E-02 | 6.19E-02 | Not significant |
| **LOC54103** | 0.835 | 1.410 | -0.756 | 3.72E-02 | 6.22E-02 | Not significant |
| **HNRPC** | 0.877 | 1.199 | -0.452 | 3.73E-02 | 6.22E-02 | Not significant |
| **COPG** | 1.081 | 0.824 | 0.391 | 3.74E-02 | 6.23E-02 | Not significant |
| **ZNF346** | 0.863 | 1.232 | -0.514 | 3.74E-02 | 6.23E-02 | Not significant |
| **ACSL4** | 0.957 | 1.327 | -0.471 | 3.75E-02 | 6.24E-02 | Not significant |
| **F12** | 1.608 | 0.402 | 1.999 | 3.78E-02 | 6.27E-02 | Not significant |
| **DUSP5** | 0.887 | 1.438 | -0.698 | 3.77E-02 | 6.27E-02 | Not significant |
| **AGTR1** | 0.739 | 1.943 | -1.395 | 3.79E-02 | 6.27E-02 | Not significant |
| **GPC6** | 0.806 | 2.424 | -1.588 | 3.78E-02 | 6.27E-02 | Not significant |
| **ZNF263** | 1.353 | 0.860 | 0.654 | 3.81E-02 | 6.28E-02 | Not significant |
| **IFNE1** | 6.322 | 0.898 | 2.816 | 3.82E-02 | 6.28E-02 | Not significant |
| **CRYAB** | 0.928 | 1.225 | -0.401 | 3.80E-02 | 6.28E-02 | Not significant |
| **KLC1** | 0.961 | 1.250 | -0.379 | 3.82E-02 | 6.28E-02 | Not significant |
| **SRPR** | 0.882 | 1.169 | -0.406 | 3.81E-02 | 6.28E-02 | Not significant |
| **IDH1** | 0.798 | 1.721 | -1.109 | 3.83E-02 | 6.28E-02 | Not significant |
| **C10ORF10** | 0.745 | 1.434 | -0.945 | 3.82E-02 | 6.28E-02 | Not significant |
| **PCDHGA11** | 0.735 | 2.127 | -1.534 | 3.83E-02 | 6.28E-02 | Not significant |
| **EPB41L5** | 0.891 | 2.106 | -1.242 | 3.81E-02 | 6.28E-02 | Not significant |
| **ZNF44** | 0.925 | 1.488 | -0.687 | 3.84E-02 | 6.28E-02 | Not significant |
| **TSSK3** | 2.042 | 0.940 | 1.120 | 3.85E-02 | 6.28E-02 | Not significant |
| **CRADD** | 0.835 | 1.772 | -1.086 | 3.85E-02 | 6.28E-02 | Not significant |
| **ICA1L** | 0.822 | 1.513 | -0.881 | 3.84E-02 | 6.28E-02 | Not significant |
| **FAM127A** | 0.837 | 1.263 | -0.594 | 3.87E-02 | 6.30E-02 | Not significant |
| **TRPC1** | 0.870 | 1.210 | -0.476 | 3.88E-02 | 6.31E-02 | Not significant |
| **TYRO3P** | 0.735 | 2.502 | -1.768 | 3.88E-02 | 6.31E-02 | Not significant |
| **DNAJC10** | 0.753 | 1.176 | -0.644 | 3.89E-02 | 6.32E-02 | Not significant |
| **KIAA1919** | 0.905 | 1.357 | -0.584 | 3.93E-02 | 6.34E-02 | Not significant |
| **NGRN** | 0.955 | 1.162 | -0.283 | 3.91E-02 | 6.34E-02 | Not significant |
| **MTHFSD** | 0.730 | 1.993 | -1.448 | 3.92E-02 | 6.34E-02 | Not significant |
| **PIM1** | 0.812 | 1.422 | -0.808 | 3.93E-02 | 6.34E-02 | Not significant |
| **ESAM** | 0.936 | 1.842 | -0.977 | 3.92E-02 | 6.34E-02 | Not significant |
| **GPR125** | 2.569 | 0.497 | 2.369 | 3.93E-02 | 6.34E-02 | Not significant |
| **ST3GAL5** | 1.066 | 0.942 | 0.179 | 3.94E-02 | 6.35E-02 | Not significant |
| **FAM122B** | 0.852 | 1.391 | -0.708 | 3.96E-02 | 6.36E-02 | Not significant |
| **BTRC** | 0.818 | 1.235 | -0.593 | 3.97E-02 | 6.37E-02 | Not significant |
| **ADCK2** | 0.794 | 1.156 | -0.542 | 3.99E-02 | 6.39E-02 | Not significant |
| **SLC29A1** | 0.899 | 1.285 | -0.515 | 3.98E-02 | 6.39E-02 | Not significant |
| **FLJ43276** | 0.882 | 1.459 | -0.726 | 3.99E-02 | 6.39E-02 | Not significant |
| **CHST7** | 0.876 | 1.312 | -0.583 | 3.99E-02 | 6.39E-02 | Not significant |
| **TMEM121** | 8.760 | 0.782 | 3.485 | 4.00E-02 | 6.39E-02 | Not significant |
| **FNDC4** | 0.911 | 1.533 | -0.751 | 4.00E-02 | 6.39E-02 | Not significant |
| **TAS2R48** | 0.680 | 1.254 | -0.883 | 4.02E-02 | 6.40E-02 | Not significant |
| **TIGD1** | 0.907 | 1.247 | -0.460 | 4.03E-02 | 6.41E-02 | Not significant |
| **SGSM3** | 3.126 | 0.721 | 2.116 | 4.04E-02 | 6.43E-02 | Not significant |
| **STC1** | 0.659 | 2.599 | -1.980 | 4.05E-02 | 6.43E-02 | Not significant |
| **WNT2** | 2.029 | 0.816 | 1.314 | 4.07E-02 | 6.46E-02 | Not significant |
| **COX19** | 1.382 | 0.894 | 0.629 | 4.07E-02 | 6.46E-02 | Not significant |
| **NDUFS5** | 0.692 | 1.256 | -0.860 | 4.09E-02 | 6.47E-02 | Not significant |
| **PRELP** | 0.699 | 1.627 | -1.220 | 4.10E-02 | 6.48E-02 | Not significant |
| **B2M** | 0.934 | 1.301 | -0.478 | 4.10E-02 | 6.48E-02 | Not significant |
| **ABI2** | 1.093 | 0.879 | 0.315 | 4.12E-02 | 6.50E-02 | Not significant |
| **TXNRD1** | 0.903 | 1.116 | -0.306 | 4.12E-02 | 6.50E-02 | Not significant |
| **IQSEC1** | 0.772 | 1.345 | -0.801 | 4.13E-02 | 6.50E-02 | Not significant |
| **MGC70870** | 3.439 | 0.897 | 1.939 | 4.13E-02 | 6.50E-02 | Not significant |
| **HNRNPF** | 1.176 | 0.785 | 0.583 | 4.15E-02 | 6.51E-02 | Not significant |
| **PDHB** | 0.831 | 1.135 | -0.450 | 4.16E-02 | 6.51E-02 | Not significant |
| **C13ORF3** | 1.505 | 0.673 | 1.161 | 4.16E-02 | 6.51E-02 | Not significant |
| **ENPP6** | 6.739 | 0.820 | 3.038 | 4.15E-02 | 6.51E-02 | Not significant |
| **FHL5** | 0.788 | 3.063 | -1.958 | 4.16E-02 | 6.51E-02 | Not significant |
| **PCTK3** | 2.158 | 0.632 | 1.772 | 4.17E-02 | 6.51E-02 | Not significant |
| **ATP6V0B** | 1.106 | 0.933 | 0.247 | 4.20E-02 | 6.52E-02 | Not significant |
| **AHCYL1** | 0.952 | 1.063 | -0.158 | 4.20E-02 | 6.52E-02 | Not significant |
| **MSRB3** | 0.925 | 1.163 | -0.331 | 4.21E-02 | 6.52E-02 | Not significant |
| **DHFRL1** | 0.700 | 1.263 | -0.852 | 4.21E-02 | 6.52E-02 | Not significant |
| **ABLIM2** | 0.873 | 1.988 | -1.188 | 4.22E-02 | 6.52E-02 | Not significant |
| **ZDHHC6** | 0.904 | 1.289 | -0.512 | 4.18E-02 | 6.52E-02 | Not significant |
| **BNIP3L** | 0.859 | 1.330 | -0.630 | 4.21E-02 | 6.52E-02 | Not significant |
| **GNA14** | 0.861 | 1.680 | -0.965 | 4.20E-02 | 6.52E-02 | Not significant |
| **ZNF385C** | 5.987 | 0.607 | 3.302 | 4.21E-02 | 6.52E-02 | Not significant |
| **HSPB7** | 0.808 | 3.004 | -1.894 | 4.21E-02 | 6.52E-02 | Not significant |
| **GPATCH3** | 0.891 | 1.221 | -0.454 | 4.24E-02 | 6.53E-02 | Not significant |
| **SBNO1** | 0.892 | 1.082 | -0.278 | 4.25E-02 | 6.53E-02 | Not significant |
| **CITED2** | 0.981 | 1.400 | -0.513 | 4.26E-02 | 6.53E-02 | Not significant |
| **RNF212** | 2.425 | 0.709 | 1.775 | 4.24E-02 | 6.53E-02 | Not significant |
| **MCTP1** | 0.818 | 2.073 | -1.342 | 4.24E-02 | 6.53E-02 | Not significant |
| **PHLDB2** | 0.837 | 1.562 | -0.899 | 4.25E-02 | 6.53E-02 | Not significant |
| **HERC3** | 0.826 | 1.193 | -0.531 | 4.29E-02 | 6.58E-02 | Not significant |
| **SLC2A9** | 2.679 | 0.540 | 2.311 | 4.29E-02 | 6.58E-02 | Not significant |
| **PTPRN2** | 0.868 | 1.435 | -0.726 | 4.32E-02 | 6.60E-02 | Not significant |
| **TMEM50B** | 0.809 | 1.351 | -0.740 | 4.32E-02 | 6.60E-02 | Not significant |
| **SH2D2A** | 1.413 | 0.617 | 1.194 | 4.32E-02 | 6.60E-02 | Not significant |
| **PVRL2** | 1.108 | 0.951 | 0.221 | 4.33E-02 | 6.61E-02 | Not significant |
| **MGC20983** | 2.462 | 0.737 | 1.741 | 4.35E-02 | 6.62E-02 | Not significant |
| **PKIA** | 0.787 | 1.443 | -0.874 | 4.35E-02 | 6.62E-02 | Not significant |
| **C14ORF139** | 0.817 | 1.923 | -1.236 | 4.35E-02 | 6.62E-02 | Not significant |
| **ELP4** | 0.831 | 2.296 | -1.466 | 4.36E-02 | 6.62E-02 | Not significant |
| **TGIF1** | 1.098 | 0.852 | 0.365 | 4.39E-02 | 6.63E-02 | Not significant |
| **PSD** | 1.847 | 0.894 | 1.046 | 4.39E-02 | 6.63E-02 | Not significant |
| **ZNF17** | 0.877 | 1.116 | -0.347 | 4.38E-02 | 6.63E-02 | Not significant |
| **FAM36A** | 0.772 | 1.719 | -1.155 | 4.38E-02 | 6.63E-02 | Not significant |
| **DOC2B** | 0.757 | 1.906 | -1.333 | 4.38E-02 | 6.63E-02 | Not significant |
| **IFI44** | 0.856 | 1.247 | -0.542 | 4.40E-02 | 6.63E-02 | Not significant |
| **C2ORF32** | 0.770 | 2.947 | -1.937 | 4.41E-02 | 6.64E-02 | Not significant |
| **ENPP4** | 0.674 | 1.785 | -1.406 | 4.41E-02 | 6.64E-02 | Not significant |
| **MGC35361** | 0.682 | 1.474 | -1.111 | 4.42E-02 | 6.64E-02 | Not significant |
| **SLC36A2** | 8.369 | 0.927 | 3.174 | 4.43E-02 | 6.65E-02 | Not significant |
| **PAIP2** | 0.905 | 1.159 | -0.357 | 4.43E-02 | 6.65E-02 | Not significant |
| **HIGD1A** | 0.883 | 1.101 | -0.319 | 4.46E-02 | 6.69E-02 | Not significant |
| **CRTC1** | 0.937 | 1.349 | -0.526 | 4.47E-02 | 6.69E-02 | Not significant |
| **CRY2** | 0.896 | 1.407 | -0.652 | 4.54E-02 | 6.78E-02 | Not significant |
| **C4ORF36** | 0.652 | 1.588 | -1.284 | 4.53E-02 | 6.78E-02 | Not significant |
| **CD93** | 0.810 | 2.186 | -1.432 | 4.54E-02 | 6.78E-02 | Not significant |
| **CLEC14A** | 0.917 | 1.769 | -0.948 | 4.55E-02 | 6.78E-02 | Not significant |
| **PPP3R1** | 0.911 | 1.725 | -0.922 | 4.56E-02 | 6.79E-02 | Not significant |
| **FPGT** | 0.833 | 1.714 | -1.042 | 4.57E-02 | 6.80E-02 | Not significant |
| **HRASLS3** | 0.800 | 2.038 | -1.349 | 4.57E-02 | 6.80E-02 | Not significant |
| **PRPSAP2** | 1.133 | 0.936 | 0.275 | 4.59E-02 | 6.81E-02 | Not significant |
| **MTRF1L** | 0.821 | 1.131 | -0.463 | 4.60E-02 | 6.81E-02 | Not significant |
| **MSH4** | 5.121 | 0.798 | 2.682 | 4.59E-02 | 6.81E-02 | Not significant |
| **TFEB** | 3.449 | 0.660 | 2.384 | 4.60E-02 | 6.81E-02 | Not significant |
| **SHOC2** | 0.833 | 1.338 | -0.684 | 4.63E-02 | 6.84E-02 | Not significant |
| **GSTM4** | 0.758 | 1.267 | -0.741 | 4.63E-02 | 6.84E-02 | Not significant |
| **GUCY1A2** | 0.919 | 1.459 | -0.666 | 4.64E-02 | 6.84E-02 | Not significant |
| **RRAS** | 0.929 | 1.457 | -0.648 | 4.68E-02 | 6.89E-02 | Not significant |
| **EVI1** | 0.662 | 1.736 | -1.390 | 4.67E-02 | 6.89E-02 | Not significant |
| **RMND1** | 0.810 | 1.658 | -1.034 | 4.68E-02 | 6.89E-02 | Not significant |
| **SAPS2** | 3.670 | 0.759 | 2.273 | 4.70E-02 | 6.91E-02 | Not significant |
| **CCDC65** | 1.593 | 0.773 | 1.043 | 4.72E-02 | 6.92E-02 | Not significant |
| **CPEB1** | 0.878 | 1.312 | -0.580 | 4.71E-02 | 6.92E-02 | Not significant |
| **PHACTR2** | 0.829 | 1.113 | -0.425 | 4.73E-02 | 6.93E-02 | Not significant |
| **EFHD1** | 1.139 | 0.950 | 0.262 | 4.77E-02 | 6.96E-02 | Not significant |
| **VWA5A** | 0.850 | 1.170 | -0.460 | 4.76E-02 | 6.96E-02 | Not significant |
| **NR3C1** | 0.957 | 1.911 | -0.998 | 4.76E-02 | 6.96E-02 | Not significant |
| **LOC283551** | 2.393 | 0.814 | 1.556 | 4.78E-02 | 6.97E-02 | Not significant |
| **MPPED2** | 0.821 | 1.581 | -0.946 | 4.79E-02 | 6.97E-02 | Not significant |
| **LOC440925** | 0.866 | 4.701 | -2.441 | 4.78E-02 | 6.97E-02 | Not significant |
| **ANO8** | 1.165 | 0.834 | 0.483 | 4.80E-02 | 6.98E-02 | Not significant |
| **PTH2** | 2.484 | 0.770 | 1.689 | 4.83E-02 | 7.01E-02 | Not significant |
| **EPAS1** | 0.934 | 1.559 | -0.740 | 4.82E-02 | 7.01E-02 | Not significant |
| **PDCD10** | 0.946 | 1.273 | -0.428 | 4.84E-02 | 7.02E-02 | Not significant |
| **CCDC46** | 0.887 | 1.318 | -0.571 | 4.86E-02 | 7.04E-02 | Not significant |
| **RAB28** | 0.845 | 1.357 | -0.684 | 4.88E-02 | 7.06E-02 | Not significant |
| **LGALS7** | 1.509 | 0.762 | 0.985 | 4.89E-02 | 7.07E-02 | Not significant |
| **XPO1** | 0.918 | 1.285 | -0.486 | 5.00E-02 | 7.14E-02 | Not significant |
| **ZNF114** | 3.622 | 0.810 | 2.160 | 4.98E-02 | 7.14E-02 | Not significant |
| **EIF3B** | 1.114 | 0.899 | 0.309 | 5.00E-02 | 7.14E-02 | Not significant |
| **FKBP8** | 0.926 | 1.123 | -0.279 | 5.00E-02 | 7.14E-02 | Not significant |
| **BAZ2B** | 0.863 | 1.220 | -0.499 | 4.95E-02 | 7.14E-02 | Not significant |
| **FBXO15** | 5.011 | 0.859 | 2.545 | 4.96E-02 | 7.14E-02 | Not significant |
| **CTSF** | 0.812 | 1.209 | -0.574 | 4.96E-02 | 7.14E-02 | Not significant |
| **MAOA** | 0.881 | 1.493 | -0.760 | 4.98E-02 | 7.14E-02 | Not significant |
| **C19ORF4** | 3.638 | 0.763 | 2.253 | 5.00E-02 | 7.14E-02 | Not significant |
| **ABHD4** | 0.923 | 1.358 | -0.557 | 4.95E-02 | 7.14E-02 | Not significant |
| **TNIK** | 0.821 | 1.805 | -1.136 | 4.99E-02 | 7.14E-02 | Not significant |
| **C9ORF163** | 7.709 | 0.802 | 3.266 | 4.99E-02 | 7.14E-02 | Not significant |
| **KIF15** | 6.919 | 0.839 | 3.043 | 5.02E-02 | 7.17E-02 | Not significant |
| **GLUD1** | 0.965 | 1.191 | -0.303 | 5.05E-02 | 7.18E-02 | Not significant |
| **BST2** | 0.907 | 1.410 | -0.636 | 5.05E-02 | 7.18E-02 | Not significant |
| **PRKCA** | 0.892 | 1.507 | -0.756 | 5.05E-02 | 7.18E-02 | Not significant |
| **KIF11** | 2.087 | 0.679 | 1.620 | 5.04E-02 | 7.18E-02 | Not significant |
| **SV2B** | 0.739 | 2.360 | -1.675 | 5.04E-02 | 7.18E-02 | Not significant |
| **CCDC85A** | 0.664 | 2.528 | -1.928 | 5.06E-02 | 7.18E-02 | Not significant |
| **OVOL1** | 2.896 | 0.828 | 1.807 | 5.07E-02 | 7.18E-02 | Not significant |
| **ARL9** | 2.688 | 0.689 | 1.964 | 5.08E-02 | 7.19E-02 | Not significant |
| **ACADSB** | 0.959 | 1.406 | -0.552 | 5.10E-02 | 7.21E-02 | Not significant |
| **RPS6KA2** | 0.817 | 1.891 | -1.211 | 5.10E-02 | 7.21E-02 | Not significant |
| **FLJ43806** | 2.783 | 0.756 | 1.880 | 5.11E-02 | 7.21E-02 | Not significant |
| **ATF6** | 0.744 | 1.242 | -0.738 | 5.12E-02 | 7.22E-02 | Not significant |
| **AGT** | 0.898 | 4.886 | -2.444 | 5.12E-02 | 7.22E-02 | Not significant |
| **C9ORF25** | 0.950 | 1.322 | -0.477 | 5.15E-02 | 7.24E-02 | Not significant |
| **EXOSC10** | 1.176 | 0.904 | 0.380 | 5.18E-02 | 7.27E-02 | Not significant |
| **RAVER2** | 0.686 | 1.407 | -1.036 | 5.18E-02 | 7.27E-02 | Not significant |
| **PLA2G4F** | 0.604 | 2.232 | -1.885 | 5.18E-02 | 7.27E-02 | Not significant |
| **CYP4X1** | 0.662 | 2.615 | -1.982 | 5.19E-02 | 7.27E-02 | Not significant |
| **NCOR1** | 0.920 | 1.245 | -0.437 | 5.20E-02 | 7.28E-02 | Not significant |
| **MAG** | 7.522 | 0.851 | 3.145 | 5.21E-02 | 7.28E-02 | Not significant |
| **MYADM** | 0.892 | 1.205 | -0.434 | 5.21E-02 | 7.28E-02 | Not significant |
| **ZNF544** | 1.635 | 0.477 | 1.778 | 5.22E-02 | 7.29E-02 | Not significant |
| **PAPSS2** | 0.676 | 1.837 | -1.442 | 5.25E-02 | 7.32E-02 | Not significant |
| **NFE2L3** | 0.945 | 1.206 | -0.352 | 5.31E-02 | 7.38E-02 | Not significant |
| **NR2F2** | 0.894 | 1.310 | -0.552 | 5.30E-02 | 7.38E-02 | Not significant |
| **HUS1B** | 0.779 | 1.482 | -0.928 | 5.31E-02 | 7.38E-02 | Not significant |
| **INVS** | 0.871 | 1.195 | -0.455 | 5.32E-02 | 7.38E-02 | Not significant |
| **RGPD5** | 0.927 | 1.486 | -0.682 | 5.32E-02 | 7.38E-02 | Not significant |
| **KDELR1** | 0.945 | 1.437 | -0.605 | 5.34E-02 | 7.39E-02 | Not significant |
| **MXRA7** | 0.965 | 1.341 | -0.475 | 5.34E-02 | 7.39E-02 | Not significant |
| **CDC37L1** | 0.935 | 1.533 | -0.714 | 5.35E-02 | 7.39E-02 | Not significant |
| **C14ORF178** | 6.793 | 0.687 | 3.306 | 5.35E-02 | 7.39E-02 | Not significant |
| **CCNB1** | 1.404 | 0.868 | 0.694 | 5.37E-02 | 7.42E-02 | Not significant |
| **TMBIM1** | 0.904 | 1.637 | -0.857 | 5.38E-02 | 7.42E-02 | Not significant |
| **C15ORF42** | 7.287 | 0.753 | 3.274 | 5.38E-02 | 7.42E-02 | Not significant |
| **RUNDC1** | 0.782 | 1.125 | -0.524 | 5.41E-02 | 7.44E-02 | Not significant |
| **CYB5B** | 0.957 | 1.316 | -0.460 | 5.45E-02 | 7.50E-02 | Not significant |
| **RREB1** | 0.893 | 1.148 | -0.364 | 5.46E-02 | 7.50E-02 | Not significant |
| **RAB2A** | 0.915 | 1.241 | -0.439 | 5.47E-02 | 7.50E-02 | Not significant |
| **SNX10** | 0.985 | 1.580 | -0.681 | 5.47E-02 | 7.50E-02 | Not significant |
| **ARHGAP29** | 0.782 | 1.775 | -1.182 | 5.48E-02 | 7.50E-02 | Not significant |
| **PREX2** | 0.840 | 2.247 | -1.420 | 5.48E-02 | 7.50E-02 | Not significant |
| **SILV** | 0.846 | 3.009 | -1.831 | 5.49E-02 | 7.51E-02 | Not significant |
| **AXL** | 0.931 | 1.531 | -0.718 | 5.50E-02 | 7.51E-02 | Not significant |
| **MEOX1** | 0.863 | 2.309 | -1.420 | 5.50E-02 | 7.51E-02 | Not significant |
| **CACNA2D1** | 0.968 | 1.479 | -0.611 | 5.51E-02 | 7.51E-02 | Not significant |
| **UGT2B15** | 2.826 | 0.773 | 1.870 | 5.52E-02 | 7.51E-02 | Not significant |
| **NKD1** | 0.794 | 1.946 | -1.294 | 5.52E-02 | 7.52E-02 | Not significant |
| **SLC25A16** | 0.935 | 1.477 | -0.659 | 5.53E-02 | 7.52E-02 | Not significant |
| **TEX11** | 0.991 | 1.335 | -0.430 | 5.55E-02 | 7.53E-02 | Not significant |
| **RAI14** | 0.902 | 1.278 | -0.502 | 5.55E-02 | 7.53E-02 | Not significant |
| **GPAM** | 0.733 | 3.534 | -2.269 | 5.57E-02 | 7.54E-02 | Not significant |
| **COL12A1** | 0.724 | 2.063 | -1.510 | 5.57E-02 | 7.54E-02 | Not significant |
| **CCDC148** | 5.656 | 0.768 | 2.880 | 5.59E-02 | 7.56E-02 | Not significant |
| **ANKDD1A** | 0.958 | 1.609 | -0.748 | 5.60E-02 | 7.57E-02 | Not significant |
| **CCDC91** | 0.808 | 1.164 | -0.527 | 5.61E-02 | 7.58E-02 | Not significant |
| **BRD7** | 1.252 | 0.879 | 0.510 | 5.62E-02 | 7.58E-02 | Not significant |
| **CASKIN1** | 8.173 | 0.777 | 3.395 | 5.65E-02 | 7.61E-02 | Not significant |
| **BCKDHB** | 0.913 | 1.253 | -0.456 | 5.66E-02 | 7.62E-02 | Not significant |
| **FAM119A** | 2.158 | 0.873 | 1.306 | 5.67E-02 | 7.62E-02 | Not significant |
| **DBNDD2** | 0.908 | 1.365 | -0.588 | 5.70E-02 | 7.65E-02 | Not significant |
| **NXNL1** | 0.783 | 1.890 | -1.271 | 5.69E-02 | 7.65E-02 | Not significant |
| **C8ORF32** | 0.861 | 1.579 | -0.875 | 5.73E-02 | 7.67E-02 | Not significant |
| **KIF20A** | 8.033 | 0.766 | 3.390 | 5.73E-02 | 7.67E-02 | Not significant |
| **FBXL5** | 0.901 | 1.339 | -0.572 | 5.72E-02 | 7.67E-02 | Not significant |
| **GABARAPL1** | 0.836 | 1.687 | -1.013 | 5.76E-02 | 7.70E-02 | Not significant |
| **RUSC2** | 0.742 | 1.199 | -0.693 | 5.77E-02 | 7.71E-02 | Not significant |
| **CDS2** | 0.918 | 1.338 | -0.545 | 5.79E-02 | 7.71E-02 | Not significant |
| **LRRC32** | 0.889 | 1.600 | -0.847 | 5.77E-02 | 7.71E-02 | Not significant |
| **GNG5** | 0.875 | 1.483 | -0.761 | 5.78E-02 | 7.71E-02 | Not significant |
| **PCDHB2** | 1.039 | 3.941 | -1.924 | 5.79E-02 | 7.71E-02 | Not significant |
| **YTHDC2** | 0.963 | 1.219 | -0.340 | 5.80E-02 | 7.72E-02 | Not significant |
| **LYST** | 0.879 | 1.211 | -0.462 | 5.81E-02 | 7.72E-02 | Not significant |
| **WFDC2** | 2.460 | 0.559 | 2.137 | 5.81E-02 | 7.72E-02 | Not significant |
| **FBP1** | 0.906 | 1.356 | -0.582 | 5.83E-02 | 7.73E-02 | Not significant |
| **RAPGEF3** | 0.782 | 2.150 | -1.459 | 5.84E-02 | 7.74E-02 | Not significant |
| **MAL** | 0.769 | 1.249 | -0.699 | 5.85E-02 | 7.75E-02 | Not significant |
| **C4ORF45** | 2.352 | 0.842 | 1.482 | 5.89E-02 | 7.76E-02 | Not significant |
| **RRAGB** | 0.864 | 1.274 | -0.561 | 5.89E-02 | 7.76E-02 | Not significant |
| **RAB11A** | 0.868 | 1.241 | -0.516 | 5.88E-02 | 7.76E-02 | Not significant |
| **ARHGAP6** | 0.890 | 1.304 | -0.550 | 5.88E-02 | 7.76E-02 | Not significant |
| **FAM127B** | 0.910 | 1.417 | -0.639 | 5.87E-02 | 7.76E-02 | Not significant |
| **TAPT1** | 0.838 | 1.494 | -0.834 | 5.89E-02 | 7.76E-02 | Not significant |
| **CASC5** | 5.797 | 0.715 | 3.020 | 5.91E-02 | 7.78E-02 | Not significant |
| **TRIM39** | 0.902 | 1.338 | -0.570 | 5.93E-02 | 7.79E-02 | Not significant |
| **BAG3** | 0.878 | 1.180 | -0.426 | 5.94E-02 | 7.80E-02 | Not significant |
| **DRD1** | 0.946 | 1.217 | -0.364 | 5.96E-02 | 7.82E-02 | Not significant |
| **F8A1** | 0.910 | 1.212 | -0.413 | 5.98E-02 | 7.83E-02 | Not significant |
| **EPHA4** | 0.898 | 1.234 | -0.459 | 5.97E-02 | 7.83E-02 | Not significant |
| **RECQL4** | 2.009 | 0.711 | 1.499 | 6.00E-02 | 7.84E-02 | Not significant |
| **STK32A** | 0.534 | 2.013 | -1.915 | 6.01E-02 | 7.85E-02 | Not significant |
| **UBE2H** | 0.942 | 1.129 | -0.261 | 6.03E-02 | 7.87E-02 | Not significant |
| **CCDC48** | 0.865 | 1.211 | -0.486 | 6.04E-02 | 7.87E-02 | Not significant |
| **HOXB5** | 0.912 | 1.337 | -0.553 | 6.04E-02 | 7.87E-02 | Not significant |
| **IGLL3** | 1.825 | 0.477 | 1.935 | 6.06E-02 | 7.88E-02 | Not significant |
| **BMP7** | 1.315 | 0.967 | 0.444 | 6.08E-02 | 7.91E-02 | Not significant |
| **UBE2A** | 0.895 | 1.241 | -0.471 | 6.09E-02 | 7.91E-02 | Not significant |
| **ENPP2** | 0.997 | 1.407 | -0.496 | 6.10E-02 | 7.92E-02 | Not significant |
| **GLDN** | 0.743 | 4.265 | -2.522 | 6.10E-02 | 7.92E-02 | Not significant |
| **GPR116** | 1.006 | 1.577 | -0.648 | 6.12E-02 | 7.93E-02 | Not significant |
| **FER** | 0.831 | 1.735 | -1.061 | 6.15E-02 | 7.93E-02 | Not significant |
| **CDCA8** | 2.412 | 0.804 | 1.584 | 6.15E-02 | 7.93E-02 | Not significant |
| **GATAD1** | 0.930 | 1.313 | -0.497 | 6.14E-02 | 7.93E-02 | Not significant |
| **KLKB1** | 0.991 | 1.535 | -0.632 | 6.15E-02 | 7.93E-02 | Not significant |
| **KIAA0367** | 0.664 | 1.537 | -1.211 | 6.13E-02 | 7.93E-02 | Not significant |
| **HIF3A** | 0.809 | 1.851 | -1.195 | 6.15E-02 | 7.93E-02 | Not significant |
| **PNLDC1** | 2.396 | 0.859 | 1.480 | 6.17E-02 | 7.94E-02 | Not significant |
| **MUTYH** | 0.809 | 1.308 | -0.693 | 6.19E-02 | 7.94E-02 | Not significant |
| **IFITM2** | 0.978 | 1.384 | -0.501 | 6.19E-02 | 7.94E-02 | Not significant |
| **TWIST1** | 0.921 | 1.466 | -0.670 | 6.18E-02 | 7.94E-02 | Not significant |
| **ESPL1** | 2.627 | 0.814 | 1.690 | 6.18E-02 | 7.94E-02 | Not significant |
| **RPS13** | 0.885 | 1.057 | -0.257 | 6.24E-02 | 7.96E-02 | Not significant |
| **KIAA1754** | 0.985 | 1.256 | -0.352 | 6.29E-02 | 7.96E-02 | Not significant |
| **ANKRD29** | 1.006 | 2.748 | -1.450 | 6.22E-02 | 7.96E-02 | Not significant |
| **SLC16A8** | 6.506 | 0.852 | 2.933 | 6.24E-02 | 7.96E-02 | Not significant |
| **HIP1** | 0.886 | 1.305 | -0.559 | 6.28E-02 | 7.96E-02 | Not significant |
| **SEMA3B** | 0.965 | 1.474 | -0.612 | 6.27E-02 | 7.96E-02 | Not significant |
| **PGAP1** | 0.941 | 1.251 | -0.411 | 6.27E-02 | 7.96E-02 | Not significant |
| **UGCGL1** | 0.876 | 1.217 | -0.473 | 6.30E-02 | 7.96E-02 | Not significant |
| **ZFX** | 0.922 | 1.349 | -0.548 | 6.24E-02 | 7.96E-02 | Not significant |
| **WDR93** | 3.754 | 0.843 | 2.155 | 6.29E-02 | 7.96E-02 | Not significant |
| **POU5F1P1** | 5.237 | 0.856 | 2.613 | 6.28E-02 | 7.96E-02 | Not significant |
| **CST3** | 0.847 | 1.256 | -0.568 | 6.26E-02 | 7.96E-02 | Not significant |
| **NDUFB2** | 0.824 | 1.388 | -0.752 | 6.24E-02 | 7.96E-02 | Not significant |
| **ASPH** | 0.868 | 1.589 | -0.873 | 6.23E-02 | 7.96E-02 | Not significant |
| **SERPINB13** | 2.262 | 0.750 | 1.593 | 6.30E-02 | 7.96E-02 | Not significant |
| **ITSN1** | 0.850 | 1.819 | -1.097 | 6.26E-02 | 7.96E-02 | Not significant |
| **ANGPTL2** | 0.824 | 1.436 | -0.800 | 6.30E-02 | 7.96E-02 | Not significant |
| **FAM83B** | 2.353 | 0.832 | 1.500 | 6.33E-02 | 7.99E-02 | Not significant |
| **VEZF1** | 0.906 | 1.418 | -0.647 | 6.36E-02 | 8.02E-02 | Not significant |
| **PPP1R3C** | 0.794 | 1.238 | -0.641 | 6.38E-02 | 8.04E-02 | Not significant |
| **SS18** | 0.741 | 1.325 | -0.839 | 6.39E-02 | 8.05E-02 | Not significant |
| **OR52K1** | 3.340 | 0.830 | 2.008 | 6.41E-02 | 8.06E-02 | Not significant |
| **N4BP3** | 0.912 | 2.149 | -1.237 | 6.42E-02 | 8.07E-02 | Not significant |
| **ZNF554** | 0.902 | 1.196 | -0.406 | 6.43E-02 | 8.07E-02 | Not significant |
| **MGC45491** | 0.938 | 1.316 | -0.489 | 6.43E-02 | 8.07E-02 | Not significant |
| **E2F4** | 0.935 | 1.315 | -0.492 | 6.47E-02 | 8.09E-02 | Not significant |
| **WASL** | 0.893 | 1.192 | -0.417 | 6.47E-02 | 8.09E-02 | Not significant |
| **MAP2K4** | 0.939 | 1.248 | -0.410 | 6.47E-02 | 8.09E-02 | Not significant |
| **CACNB2** | 0.876 | 1.809 | -1.046 | 6.46E-02 | 8.09E-02 | Not significant |
| **RSBN1L** | 0.900 | 1.214 | -0.432 | 6.48E-02 | 8.09E-02 | Not significant |
| **RNASE4** | 0.893 | 1.741 | -0.964 | 6.48E-02 | 8.09E-02 | Not significant |
| **CFI** | 0.911 | 1.227 | -0.430 | 6.50E-02 | 8.09E-02 | Not significant |
| **MED20** | 0.860 | 1.225 | -0.511 | 6.50E-02 | 8.09E-02 | Not significant |
| **SLC46A3** | 0.765 | 1.459 | -0.931 | 6.53E-02 | 8.13E-02 | Not significant |
| **LRRN4CL** | 0.520 | 1.597 | -1.619 | 6.55E-02 | 8.15E-02 | Not significant |
| **ANXA6** | 0.921 | 1.319 | -0.518 | 6.57E-02 | 8.15E-02 | Not significant |
| **LAMC1** | 0.883 | 1.951 | -1.143 | 6.56E-02 | 8.15E-02 | Not significant |
| **C9ORF140** | 4.331 | 0.842 | 2.363 | 6.58E-02 | 8.16E-02 | Not significant |
| **NDUFA7** | 0.776 | 1.608 | -1.052 | 6.58E-02 | 8.16E-02 | Not significant |
| **ACSL1** | 0.933 | 1.233 | -0.402 | 6.62E-02 | 8.18E-02 | Not significant |
| **ALG2** | 0.898 | 1.389 | -0.629 | 6.62E-02 | 8.18E-02 | Not significant |
| **MID1IP1** | 0.891 | 1.692 | -0.924 | 6.62E-02 | 8.18E-02 | Not significant |
| **LTBR** | 0.762 | 1.832 | -1.264 | 6.61E-02 | 8.18E-02 | Not significant |
| **ZCWPW2** | 0.640 | 1.411 | -1.141 | 6.65E-02 | 8.20E-02 | Not significant |
| **TCP10** | 0.945 | 1.145 | -0.277 | 6.66E-02 | 8.21E-02 | Not significant |
| **FOXI1** | 3.681 | 0.884 | 2.057 | 6.67E-02 | 8.22E-02 | Not significant |
| **DOCK11** | 0.946 | 1.458 | -0.623 | 6.72E-02 | 8.26E-02 | Not significant |
| **DUSP12** | 0.735 | 1.523 | -1.052 | 6.73E-02 | 8.26E-02 | Not significant |
| **CDKL3** | 2.404 | 0.671 | 1.841 | 6.73E-02 | 8.26E-02 | Not significant |
| **FAM107A** | 0.847 | 2.018 | -1.253 | 6.73E-02 | 8.26E-02 | Not significant |
| **BMP5** | 0.817 | 2.389 | -1.548 | 6.71E-02 | 8.26E-02 | Not significant |
| **ATP2B4** | 0.926 | 1.528 | -0.723 | 6.78E-02 | 8.29E-02 | Not significant |
| **STOX1** | 0.764 | 1.946 | -1.349 | 6.77E-02 | 8.29E-02 | Not significant |
| **LGR5** | 0.568 | 2.758 | -2.278 | 6.78E-02 | 8.29E-02 | Not significant |
| **GGNBP2** | 0.872 | 3.596 | -2.044 | 6.78E-02 | 8.29E-02 | Not significant |
| **CDKN1B** | 0.914 | 1.318 | -0.528 | 6.81E-02 | 8.32E-02 | Not significant |
| **MSTN** | 4.299 | 0.820 | 2.391 | 6.84E-02 | 8.34E-02 | Not significant |
| **PVRIG** | 1.601 | 0.781 | 1.035 | 6.85E-02 | 8.34E-02 | Not significant |
| **CAV2** | 0.986 | 1.517 | -0.622 | 6.84E-02 | 8.34E-02 | Not significant |
| **C2ORF30** | 0.730 | 1.284 | -0.815 | 6.86E-02 | 8.34E-02 | Not significant |
| **MAGI2** | 0.822 | 1.540 | -0.906 | 6.88E-02 | 8.36E-02 | Not significant |
| **ECM2** | 0.999 | 2.306 | -1.207 | 6.89E-02 | 8.38E-02 | Not significant |
| **GNG11** | 0.849 | 1.632 | -0.942 | 6.90E-02 | 8.38E-02 | Not significant |
| **FASTKD2** | 0.941 | 1.189 | -0.337 | 6.91E-02 | 8.38E-02 | Not significant |
| **HLA-DQA2** | 1.737 | 0.455 | 1.931 | 6.95E-02 | 8.42E-02 | Not significant |
| **DKK2** | 1.028 | 2.317 | -1.173 | 6.96E-02 | 8.43E-02 | Not significant |
| **GLUL** | 0.904 | 1.297 | -0.520 | 6.97E-02 | 8.43E-02 | Not significant |
| **LOC283874** | 0.911 | 1.145 | -0.330 | 6.99E-02 | 8.45E-02 | Not significant |
| **GPR20** | 0.865 | 2.676 | -1.629 | 6.99E-02 | 8.45E-02 | Not significant |
| **TTMA** | 6.414 | 0.921 | 2.800 | 7.01E-02 | 8.45E-02 | Not significant |
| **SOX17** | 0.795 | 1.452 | -0.869 | 7.02E-02 | 8.46E-02 | Not significant |
| **PRDM16** | 0.957 | 2.330 | -1.285 | 7.05E-02 | 8.49E-02 | Not significant |
| **PCDHB10** | 0.864 | 1.262 | -0.547 | 7.06E-02 | 8.49E-02 | Not significant |
| **UCHL1** | 0.847 | 1.267 | -0.581 | 7.07E-02 | 8.49E-02 | Not significant |
| **FAM58A** | 0.818 | 1.357 | -0.730 | 7.07E-02 | 8.49E-02 | Not significant |
| **ICOS** | 1.681 | 0.845 | 0.992 | 7.10E-02 | 8.53E-02 | Not significant |
| **SLC10A6** | 0.827 | 2.310 | -1.481 | 7.12E-02 | 8.54E-02 | Not significant |
| **FAM71E1** | 4.878 | 0.822 | 2.569 | 7.17E-02 | 8.60E-02 | Not significant |
| **TRIM35** | 0.831 | 1.910 | -1.200 | 7.20E-02 | 8.62E-02 | Not significant |
| **ART4** | 0.828 | 3.437 | -2.054 | 7.20E-02 | 8.62E-02 | Not significant |
| **SLC8A2** | 0.867 | 2.570 | -1.568 | 7.25E-02 | 8.67E-02 | Not significant |
| **C17ORF79** | 0.994 | 1.292 | -0.377 | 7.30E-02 | 8.71E-02 | Not significant |
| **CCPG1** | 0.869 | 1.570 | -0.852 | 7.34E-02 | 8.76E-02 | Not significant |
| **FLJ40453** | 0.891 | 1.627 | -0.869 | 7.36E-02 | 8.78E-02 | Not significant |
| **BCL3** | 0.953 | 1.396 | -0.550 | 7.39E-02 | 8.80E-02 | Not significant |
| **ANKRD55** | 0.921 | 1.205 | -0.387 | 7.41E-02 | 8.81E-02 | Not significant |
| **CHAC2** | 4.467 | 0.827 | 2.434 | 7.41E-02 | 8.81E-02 | Not significant |
| **NCOA7** | 0.897 | 1.266 | -0.496 | 7.42E-02 | 8.82E-02 | Not significant |
| **WBP4** | 0.822 | 1.288 | -0.647 | 7.43E-02 | 8.82E-02 | Not significant |
| **IFRG15** | 0.970 | 1.663 | -0.778 | 7.50E-02 | 8.88E-02 | Not significant |
| **GIMAP8** | 0.981 | 1.577 | -0.684 | 7.49E-02 | 8.88E-02 | Not significant |
| **ABCA6** | 0.922 | 1.436 | -0.640 | 7.52E-02 | 8.91E-02 | Not significant |
| **DMRT3** | 0.811 | 3.054 | -1.913 | 7.53E-02 | 8.91E-02 | Not significant |
| **GPR172B** | 8.336 | 0.815 | 3.355 | 7.56E-02 | 8.93E-02 | Not significant |
| **HOXB2** | 0.869 | 2.282 | -1.393 | 7.56E-02 | 8.93E-02 | Not significant |
| **MYO1C** | 0.955 | 1.578 | -0.725 | 7.57E-02 | 8.93E-02 | Not significant |
| **TSPAN3** | 0.859 | 1.357 | -0.659 | 7.60E-02 | 8.96E-02 | Not significant |
| **C1QTNF7** | 0.710 | 2.961 | -2.061 | 7.60E-02 | 8.96E-02 | Not significant |
| **FKSG30** | 6.701 | 0.857 | 2.967 | 7.63E-02 | 8.99E-02 | Not significant |
| **CAMK2A** | 5.291 | 0.728 | 2.861 | 7.64E-02 | 8.99E-02 | Not significant |
| **ZNF687** | 0.789 | 1.739 | -1.139 | 7.65E-02 | 8.99E-02 | Not significant |
| **RIMS1** | 0.954 | 2.599 | -1.445 | 7.66E-02 | 8.99E-02 | Not significant |
| **ZNF284** | 0.757 | 2.210 | -1.545 | 7.67E-02 | 9.00E-02 | Not significant |
| **TRIP13** | 6.475 | 0.645 | 3.327 | 7.68E-02 | 9.00E-02 | Not significant |
| **SORCS1** | 0.895 | 2.201 | -1.298 | 7.69E-02 | 9.01E-02 | Not significant |
| **BRUNOL5** | 4.668 | 0.301 | 3.954 | 7.71E-02 | 9.02E-02 | Not significant |
| **AGBL4** | 3.533 | 0.822 | 2.104 | 7.72E-02 | 9.03E-02 | Not significant |
| **KCNK5** | 2.184 | 0.706 | 1.629 | 7.74E-02 | 9.04E-02 | Not significant |
| **FILIP1** | 0.786 | 1.521 | -0.953 | 7.75E-02 | 9.04E-02 | Not significant |
| **BBS2** | 0.736 | 1.422 | -0.952 | 7.79E-02 | 9.09E-02 | Not significant |
| **SLC12A3** | 2.857 | 0.832 | 1.781 | 7.83E-02 | 9.12E-02 | Not significant |
| **ZDHHC3** | 0.866 | 1.393 | -0.687 | 7.83E-02 | 9.12E-02 | Not significant |
| **ASPHD2** | 4.705 | 0.824 | 2.513 | 7.85E-02 | 9.12E-02 | Not significant |
| **ACO1** | 0.910 | 1.373 | -0.593 | 7.84E-02 | 9.12E-02 | Not significant |
| **HBB** | 0.841 | 1.346 | -0.678 | 7.88E-02 | 9.15E-02 | Not significant |
| **ADRA1A** | 1.100 | 5.421 | -2.302 | 7.88E-02 | 9.15E-02 | Not significant |
| **C4ORF31** | 0.779 | 3.547 | -2.186 | 7.91E-02 | 9.18E-02 | Not significant |
| **RAB2B** | 0.969 | 1.414 | -0.545 | 7.92E-02 | 9.18E-02 | Not significant |
| **NCK1** | 0.747 | 1.663 | -1.154 | 7.92E-02 | 9.18E-02 | Not significant |
| **TAOK1** | 0.931 | 1.170 | -0.330 | 7.94E-02 | 9.18E-02 | Not significant |
| **SCNN1B** | 0.828 | 1.926 | -1.218 | 7.96E-02 | 9.20E-02 | Not significant |
| **LOC203547** | 0.871 | 1.202 | -0.464 | 7.97E-02 | 9.20E-02 | Not significant |
| **PDGFB** | 0.857 | 2.217 | -1.371 | 7.97E-02 | 9.20E-02 | Not significant |
| **NT5M** | 5.283 | 0.760 | 2.797 | 8.01E-02 | 9.24E-02 | Not significant |
| **HOXC11** | 0.947 | 1.463 | -0.627 | 8.05E-02 | 9.27E-02 | Not significant |
| **ADIPOQ** | 0.825 | 2.554 | -1.630 | 8.05E-02 | 9.27E-02 | Not significant |
| **PLOD2** | 0.802 | 1.319 | -0.718 | 8.08E-02 | 9.29E-02 | Not significant |
| **PALM** | 1.010 | 1.391 | -0.462 | 8.09E-02 | 9.30E-02 | Not significant |
| **CENPA** | 1.769 | 0.871 | 1.022 | 8.11E-02 | 9.31E-02 | Not significant |
| **ZNF470** | 0.899 | 1.514 | -0.752 | 8.13E-02 | 9.33E-02 | Not significant |
| **VNN1** | 0.847 | 1.494 | -0.819 | 8.14E-02 | 9.34E-02 | Not significant |
| **GLIS2** | 0.936 | 1.335 | -0.512 | 8.18E-02 | 9.36E-02 | Not significant |
| **ELMO1** | 0.869 | 1.349 | -0.634 | 8.18E-02 | 9.36E-02 | Not significant |
| **GALE** | 1.518 | 0.822 | 0.885 | 8.19E-02 | 9.36E-02 | Not significant |
| **R3HDM2** | 0.855 | 1.580 | -0.886 | 8.19E-02 | 9.36E-02 | Not significant |
| **PLEK** | 0.883 | 1.674 | -0.923 | 8.21E-02 | 9.37E-02 | Not significant |
| **PPARD** | 0.939 | 1.543 | -0.716 | 8.23E-02 | 9.39E-02 | Not significant |
| **GOLSYN** | 0.819 | 1.713 | -1.065 | 8.25E-02 | 9.40E-02 | Not significant |
| **TMEM104** | 0.796 | 2.168 | -1.445 | 8.27E-02 | 9.42E-02 | Not significant |
| **LACTB2** | 0.727 | 1.368 | -0.913 | 8.32E-02 | 9.47E-02 | Not significant |
| **RBMS2** | 0.939 | 1.266 | -0.430 | 8.33E-02 | 9.48E-02 | Not significant |
| **NUP107** | 0.651 | 1.405 | -1.109 | 8.43E-02 | 9.58E-02 | Not significant |
| **ABCC9** | 0.824 | 1.365 | -0.729 | 8.46E-02 | 9.61E-02 | Not significant |
| **BNIP3** | 0.638 | 1.335 | -1.066 | 8.47E-02 | 9.62E-02 | Not significant |
| **ASB9** | 0.771 | 2.058 | -1.417 | 8.58E-02 | 9.73E-02 | Not significant |
| **C11ORF57** | 0.882 | 1.288 | -0.547 | 8.61E-02 | 9.74E-02 | Not significant |
| **AQP7** | 0.935 | 1.743 | -0.899 | 8.61E-02 | 9.74E-02 | Not significant |
| **CHI3L2** | 2.193 | 0.666 | 1.720 | 8.61E-02 | 9.74E-02 | Not significant |
| **ZEB2** | 0.786 | 1.580 | -1.008 | 8.64E-02 | 9.76E-02 | Not significant |
| **CCDC153** | 0.877 | 1.525 | -0.798 | 8.64E-02 | 9.76E-02 | Not significant |
| **IGF2BP3** | 0.762 | 2.853 | -1.905 | 8.68E-02 | 9.80E-02 | Not significant |
| **SH2D3C** | 0.923 | 1.566 | -0.762 | 8.71E-02 | 9.82E-02 | Not significant |
| **TIAM1** | 0.722 | 1.506 | -1.062 | 8.79E-02 | 9.91E-02 | Not significant |
| **INMT** | 0.978 | 1.209 | -0.305 | 8.84E-02 | 9.94E-02 | Not significant |
| **RNU2-1** | 0.921 | 1.149 | -0.320 | 8.84E-02 | 9.94E-02 | Not significant |
| **PTGIR** | 0.735 | 1.942 | -1.401 | 8.86E-02 | 9.95E-02 | Not significant |
| **PAX2** | 1.994 | 0.646 | 1.626 | 8.88E-02 | 9.97E-02 | Not significant |
| **ELL** | 0.743 | 1.618 | -1.123 | 8.88E-02 | 9.97E-02 | Not significant |
| **NOTCH4** | 0.663 | 2.321 | -1.807 | 8.89E-02 | 9.97E-02 | Not significant |
| **SLC7A10** | 0.903 | 4.377 | -2.277 | 8.91E-02 | 9.99E-02 | Not significant |
| **APOL3** | 0.799 | 1.493 | -0.901 | 8.94E-02 | 1.00E-01 | Not significant |
| **PKD1L2** | 0.604 | 3.342 | -2.467 | 8.94E-02 | 1.00E-01 | Not significant |
| **GPR34** | 0.990 | 1.556 | -0.652 | 8.96E-02 | 1.00E-01 | Not significant |
| **SSTR1** | 0.887 | 1.249 | -0.494 | 9.00E-02 | 1.01E-01 | Not significant |
| **CBLN1** | 0.919 | 1.589 | -0.789 | 9.05E-02 | 1.01E-01 | Not significant |
| **ZFYVE26** | 0.906 | 1.340 | -0.565 | 9.08E-02 | 1.01E-01 | Not significant |
| **CHST4** | 2.573 | 0.724 | 1.829 | 9.20E-02 | 1.02E-01 | Not significant |
| **PCDH7** | 0.845 | 1.703 | -1.012 | 9.20E-02 | 1.02E-01 | Not significant |
| **PLIN** | 0.925 | 4.220 | -2.190 | 9.21E-02 | 1.02E-01 | Not significant |
| **KCNQ5** | 2.456 | 0.652 | 1.914 | 9.24E-02 | 1.03E-01 | Not significant |
| **GABRE** | 0.917 | 1.786 | -0.962 | 9.24E-02 | 1.03E-01 | Not significant |
| **LOC130576** | 0.771 | 1.441 | -0.903 | 9.32E-02 | 1.03E-01 | Not significant |
| **LOC340527** | 0.893 | 2.087 | -1.225 | 9.32E-02 | 1.03E-01 | Not significant |
| **SLC19A3** | 0.883 | 1.463 | -0.728 | 9.32E-02 | 1.03E-01 | Not significant |
| **HSD11B1** | 0.846 | 2.934 | -1.794 | 9.31E-02 | 1.03E-01 | Not significant |
| **TUBB2A** | 0.827 | 1.405 | -0.765 | 9.36E-02 | 1.03E-01 | Not significant |
| **HLA-F** | 0.808 | 1.762 | -1.124 | 9.36E-02 | 1.03E-01 | Not significant |
| **FLJ90650** | 0.900 | 3.265 | -1.858 | 9.35E-02 | 1.03E-01 | Not significant |
| **CNTLN** | 0.834 | 2.937 | -1.817 | 9.37E-02 | 1.03E-01 | Not significant |
| **OR4F21** | 1.291 | 4.051 | -1.650 | 9.41E-02 | 1.04E-01 | Not significant |
| **FOXP4** | 0.869 | 1.293 | -0.573 | 9.44E-02 | 1.04E-01 | Not significant |
| **SELP** | 0.858 | 1.651 | -0.945 | 9.44E-02 | 1.04E-01 | Not significant |
| **DAO** | 0.840 | 2.138 | -1.348 | 9.48E-02 | 1.04E-01 | Not significant |
| **CD207** | 0.858 | 2.504 | -1.545 | 9.54E-02 | 1.05E-01 | Not significant |
| **KIF13A** | 0.729 | 1.811 | -1.313 | 9.55E-02 | 1.05E-01 | Not significant |
| **ZNF792** | 0.921 | 1.646 | -0.838 | 9.61E-02 | 1.05E-01 | Not significant |
| **ZEB1** | 0.836 | 1.289 | -0.625 | 9.68E-02 | 1.06E-01 | Not significant |
| **LOC653566** | 0.892 | 1.563 | -0.808 | 9.76E-02 | 1.07E-01 | Not significant |
| **SCRN3** | 0.784 | 1.580 | -1.011 | 9.77E-02 | 1.07E-01 | Not significant |
| **KCNAB1** | 0.960 | 2.076 | -1.113 | 9.79E-02 | 1.07E-01 | Not significant |
| **CDH8** | 0.914 | 8.147 | -3.157 | 9.88E-02 | 1.08E-01 | Not significant |
| **C10ORF78** | 0.837 | 1.353 | -0.693 | 9.92E-02 | 1.08E-01 | Not significant |
| **REEP3** | 0.942 | 2.193 | -1.219 | 9.91E-02 | 1.08E-01 | Not significant |
| **MAN1C1** | 0.728 | 2.044 | -1.489 | 9.97E-02 | 1.09E-01 | Not significant |
| **SRXN1** | 0.816 | 1.771 | -1.118 | 1.00E-01 | 1.09E-01 | Not significant |
| **ITIH2** | 4.949 | 0.617 | 3.004 | 1.00E-01 | 1.09E-01 | Not significant |
| **PTPN11** | 0.714 | 1.985 | -1.476 | 1.00E-01 | 1.09E-01 | Not significant |
| **STT3B** | 0.869 | 1.572 | -0.856 | 1.01E-01 | 1.10E-01 | Not significant |
| **SPTBN4** | 0.926 | 2.643 | -1.513 | 1.01E-01 | 1.10E-01 | Not significant |
| **HRASLS2** | 0.886 | 1.537 | -0.795 | 1.01E-01 | 1.10E-01 | Not significant |
| **CPNE7** | 4.704 | 0.752 | 2.646 | 1.02E-01 | 1.10E-01 | Not significant |
| **CLDN6** | 1.306 | 8.544 | -2.709 | 1.02E-01 | 1.10E-01 | Not significant |
| **MYCT1** | 0.947 | 2.220 | -1.228 | 1.02E-01 | 1.10E-01 | Not significant |
| **EPHX1** | 0.709 | 2.286 | -1.689 | 1.03E-01 | 1.12E-01 | Not significant |
| **KPNA6** | 0.867 | 1.250 | -0.527 | 1.04E-01 | 1.12E-01 | Not significant |
| **TNMD** | 0.868 | 2.118 | -1.287 | 1.04E-01 | 1.12E-01 | Not significant |
| **TTPA** | 0.811 | 1.867 | -1.203 | 1.04E-01 | 1.12E-01 | Not significant |
| **RORB** | 0.845 | 4.173 | -2.305 | 1.04E-01 | 1.13E-01 | Not significant |
| **A2BP1** | 0.765 | 5.898 | -2.946 | 1.05E-01 | 1.13E-01 | Not significant |
| **PRKAR2B** | 1.009 | 2.877 | -1.511 | 1.06E-01 | 1.14E-01 | Not significant |
| **MMD** | 0.809 | 3.860 | -2.253 | 1.07E-01 | 1.15E-01 | Not significant |
| **PCDHGB7** | 0.733 | 2.688 | -1.874 | 1.08E-01 | 1.16E-01 | Not significant |
| **ANKRD43** | 0.804 | 2.395 | -1.575 | 1.09E-01 | 1.17E-01 | Not significant |
| **CNTFR** | 0.753 | 1.363 | -0.857 | 1.09E-01 | 1.17E-01 | Not significant |
| **TPO** | 0.801 | 1.587 | -0.986 | 1.09E-01 | 1.18E-01 | Not significant |
| **IGFBP7** | 0.821 | 1.207 | -0.556 | 1.10E-01 | 1.18E-01 | Not significant |
| **ACR** | 0.910 | 1.933 | -1.086 | 1.10E-01 | 1.18E-01 | Not significant |
| **RDH5** | 0.887 | 1.476 | -0.734 | 1.10E-01 | 1.18E-01 | Not significant |
| **C8ORF76** | 0.898 | 1.607 | -0.839 | 1.11E-01 | 1.18E-01 | Not significant |
| **AVPR1A** | 0.886 | 1.945 | -1.134 | 1.11E-01 | 1.19E-01 | Not significant |
| **THEM2** | 0.882 | 2.146 | -1.283 | 1.11E-01 | 1.19E-01 | Not significant |
| **TMEM63C** | 0.987 | 1.962 | -0.991 | 1.12E-01 | 1.19E-01 | Not significant |
| **RRAGC** | 0.869 | 1.461 | -0.749 | 1.12E-01 | 1.19E-01 | Not significant |
| **NOX5** | 3.368 | 0.815 | 2.046 | 1.12E-01 | 1.19E-01 | Not significant |
| **SERPINB9** | 0.901 | 1.522 | -0.756 | 1.12E-01 | 1.20E-01 | Not significant |
| **SELPLG** | 1.035 | 1.883 | -0.863 | 1.13E-01 | 1.21E-01 | Not significant |
| **IDH3A** | 0.837 | 1.504 | -0.845 | 1.14E-01 | 1.21E-01 | Not significant |
| **UNC5D** | 2.828 | 0.817 | 1.791 | 1.14E-01 | 1.21E-01 | Not significant |
| **VKORC1L1** | 0.867 | 1.774 | -1.032 | 1.14E-01 | 1.21E-01 | Not significant |
| **TKTL1** | 0.852 | 1.473 | -0.790 | 1.14E-01 | 1.21E-01 | Not significant |
| **CRLF1** | 2.812 | 0.906 | 1.635 | 1.14E-01 | 1.21E-01 | Not significant |
| **TMEM22** | 0.881 | 2.090 | -1.246 | 1.15E-01 | 1.21E-01 | Not significant |
| **UBAP2** | 1.031 | 2.125 | -1.043 | 1.15E-01 | 1.22E-01 | Not significant |
| **RASL10B** | 0.707 | 2.028 | -1.521 | 1.15E-01 | 1.22E-01 | Not significant |
| **COL8A2** | 0.843 | 1.541 | -0.871 | 1.16E-01 | 1.22E-01 | Not significant |
| **DRD4** | 0.819 | 1.663 | -1.021 | 1.16E-01 | 1.23E-01 | Not significant |
| **ELN** | 0.990 | 1.581 | -0.675 | 1.17E-01 | 1.23E-01 | Not significant |
| **CIDEC** | 0.912 | 1.345 | -0.560 | 1.17E-01 | 1.23E-01 | Not significant |
| **IL3RA** | 0.967 | 2.084 | -1.108 | 1.17E-01 | 1.23E-01 | Not significant |
| **THRSP** | 1.035 | 2.116 | -1.031 | 1.18E-01 | 1.24E-01 | Not significant |
| **FAM122A** | 0.807 | 1.701 | -1.075 | 1.18E-01 | 1.25E-01 | Not significant |
| **NMB** | 0.665 | 6.396 | -3.266 | 1.22E-01 | 1.29E-01 | Not significant |
| **AOC3** | 0.984 | 1.756 | -0.835 | 1.22E-01 | 1.29E-01 | Not significant |
| **TMEM47** | 0.942 | 1.430 | -0.602 | 1.23E-01 | 1.29E-01 | Not significant |
| **IL8** | 0.716 | 3.134 | -2.131 | 1.23E-01 | 1.29E-01 | Not significant |
| **GPD1** | 0.727 | 4.427 | -2.607 | 1.24E-01 | 1.30E-01 | Not significant |
| **LSAMP** | 0.846 | 1.519 | -0.844 | 1.24E-01 | 1.30E-01 | Not significant |
| **CA4** | 1.135 | 2.268 | -0.999 | 1.24E-01 | 1.30E-01 | Not significant |
| **TACR2** | 3.875 | 0.870 | 2.155 | 1.25E-01 | 1.31E-01 | Not significant |
| **PCDHGA4** | 0.786 | 2.078 | -1.403 | 1.27E-01 | 1.33E-01 | Not significant |
| **KYNU** | 0.931 | 1.869 | -1.006 | 1.27E-01 | 1.33E-01 | Not significant |
| **LHCGR** | 0.516 | 2.626 | -2.349 | 1.27E-01 | 1.33E-01 | Not significant |
| **CEBPA** | 0.921 | 1.538 | -0.739 | 1.28E-01 | 1.33E-01 | Not significant |
| **ITGA7** | 0.816 | 2.120 | -1.379 | 1.29E-01 | 1.34E-01 | Not significant |
| **SIX1** | 0.897 | 2.301 | -1.359 | 1.30E-01 | 1.35E-01 | Not significant |
| **CCDC57** | 0.740 | 3.715 | -2.327 | 1.31E-01 | 1.37E-01 | Not significant |
| **ID1** | 0.873 | 1.927 | -1.143 | 1.31E-01 | 1.37E-01 | Not significant |
| **SNF1LK2** | 0.823 | 2.257 | -1.456 | 1.32E-01 | 1.37E-01 | Not significant |
| **C1ORF51** | 0.708 | 1.983 | -1.486 | 1.33E-01 | 1.38E-01 | Not significant |
| **RANBP10** | 0.848 | 3.002 | -1.824 | 1.33E-01 | 1.38E-01 | Not significant |
| **HOXA13** | 0.594 | 2.370 | -1.995 | 1.34E-01 | 1.39E-01 | Not significant |
| **MRAP** | 0.745 | 2.873 | -1.946 | 1.34E-01 | 1.39E-01 | Not significant |
| **PGM5** | 0.743 | 1.865 | -1.327 | 1.34E-01 | 1.39E-01 | Not significant |
| **IFI27** | 0.850 | 1.698 | -0.998 | 1.35E-01 | 1.39E-01 | Not significant |
| **PPP1R16B** | 0.808 | 1.719 | -1.089 | 1.35E-01 | 1.40E-01 | Not significant |
| **PLAG1** | 0.798 | 2.237 | -1.487 | 1.36E-01 | 1.40E-01 | Not significant |
| **FLJ32011** | 1.356 | 6.943 | -2.356 | 1.37E-01 | 1.42E-01 | Not significant |
| **PPARGC1A** | 0.641 | 2.101 | -1.713 | 1.38E-01 | 1.43E-01 | Not significant |
| **GLYAT** | 0.971 | 4.206 | -2.115 | 1.38E-01 | 1.43E-01 | Not significant |
| **GPR4** | 0.763 | 2.487 | -1.705 | 1.40E-01 | 1.44E-01 | Not significant |
| **ARL8B** | 0.972 | 1.951 | -1.005 | 1.40E-01 | 1.44E-01 | Not significant |
| **KIAA1826** | 0.681 | 1.536 | -1.173 | 1.41E-01 | 1.45E-01 | Not significant |
| **KCNB1** | 0.770 | 3.210 | -2.060 | 1.41E-01 | 1.45E-01 | Not significant |
| **LALBA** | 60.150 | 0.755 | 6.317 | 1.41E-01 | 1.45E-01 | Not significant |
| **SOX30** | 0.770 | 2.152 | -1.483 | 1.42E-01 | 1.46E-01 | Not significant |
| **PYY** | 0.701 | 1.940 | -1.469 | 1.45E-01 | 1.49E-01 | Not significant |
| **CALB2** | 1.105 | 2.997 | -1.439 | 1.45E-01 | 1.49E-01 | Not significant |
| **C12ORF60** | 0.912 | 1.766 | -0.953 | 1.47E-01 | 1.50E-01 | Not significant |
| **PTPN7** | 1.095 | 2.253 | -1.041 | 1.49E-01 | 1.53E-01 | Not significant |
| **SEMA7A** | 3.664 | 0.832 | 2.139 | 1.50E-01 | 1.53E-01 | Not significant |
| **MCF2L** | 0.948 | 3.037 | -1.680 | 1.50E-01 | 1.53E-01 | Not significant |
| **C6ORF107** | 0.740 | 2.792 | -1.916 | 1.54E-01 | 1.58E-01 | Not significant |
| **LOC650695** | 0.964 | 4.738 | -2.298 | 1.55E-01 | 1.58E-01 | Not significant |
| **GRRP1** | 0.890 | 2.033 | -1.191 | 1.57E-01 | 1.60E-01 | Not significant |
| **C22ORF34** | 1.200 | 2.316 | -0.949 | 1.62E-01 | 1.65E-01 | Not significant |
| **PNLIPRP3** | 1.956 | 5.905 | -1.594 | 1.66E-01 | 1.69E-01 | Not significant |
| **CDO1** | 1.175 | 1.934 | -0.718 | 1.67E-01 | 1.70E-01 | Not significant |
| **CYP4Z1** | 0.908 | 2.366 | -1.381 | 1.68E-01 | 1.71E-01 | Not significant |
| **HOXB7** | 0.505 | 2.304 | -2.191 | 1.68E-01 | 1.71E-01 | Not significant |
| **SLC22A14** | 0.674 | 3.017 | -2.162 | 1.68E-01 | 1.71E-01 | Not significant |
| **DHRS2** | 1.573 | 5.758 | -1.872 | 1.69E-01 | 1.72E-01 | Not significant |
| **NNAT** | 0.954 | 2.050 | -1.103 | 1.69E-01 | 1.72E-01 | Not significant |
| **UGT2B11** | 1.126 | 4.650 | -2.046 | 1.71E-01 | 1.73E-01 | Not significant |
| **IL6R** | 0.900 | 2.132 | -1.244 | 1.71E-01 | 1.73E-01 | Not significant |
| **SLC25A20** | 0.677 | 1.580 | -1.221 | 1.74E-01 | 1.76E-01 | Not significant |
| **C6** | 0.912 | 3.597 | -1.980 | 1.81E-01 | 1.83E-01 | Not significant |
| **SLC4A4** | 0.861 | 3.467 | -2.009 | 1.81E-01 | 1.83E-01 | Not significant |
| **XRN2** | 3.241 | 7.687 | -1.246 | 1.82E-01 | 1.84E-01 | Not significant |
| **CYP2C19** | 0.764 | 2.223 | -1.541 | 1.87E-01 | 1.89E-01 | Not significant |
| **KLK4** | 0.663 | 2.446 | -1.883 | 1.89E-01 | 1.91E-01 | Not significant |
| **C6ORF27** | 0.822 | 1.870 | -1.185 | 1.90E-01 | 1.91E-01 | Not significant |
| **CALCRL** | 0.728 | 1.841 | -1.338 | 1.91E-01 | 1.92E-01 | Not significant |
| **CDKN2B** | 0.741 | 3.736 | -2.333 | 1.94E-01 | 1.96E-01 | Not significant |
| **C16ORF3** | 0.874 | 1.440 | -0.720 | 1.95E-01 | 1.96E-01 | Not significant |
| **ACADL** | 0.632 | 3.028 | -2.261 | 2.00E-01 | 2.01E-01 | Not significant |
| **MYOC** | 1.273 | 12.530 | -3.299 | 2.01E-01 | 2.02E-01 | Not significant |
| **WNT11** | 0.798 | 5.140 | -2.688 | 2.12E-01 | 2.13E-01 | Not significant |
| **UGT2B28** | 2.452 | 9.096 | -1.891 | 2.17E-01 | 2.18E-01 | Not significant |
| **MGEA5** | 1.002 | 1.425 | -0.508 | 2.18E-01 | 2.18E-01 | Not significant |
| **TUSC5** | 0.816 | 4.773 | -2.549 | 2.29E-01 | 2.29E-01 | Not significant |
| **LIPE** | 0.929 | 7.480 | -3.010 | 2.72E-01 | 2.72E-01 | Not significant |
| **LOXL2** | 0.996 | 1.756 | -0.818 | 3.10E-01 | 3.10E-01 | Not significant |

**Table S4. Breast-tumour contrasts used for tumor-context overlap meta-analysis**

Overlap between the noninvoluted-high gene set (higher in noninvoluted vs completely involuted; nominal P < 0.05) and tumor-associated gene lists from external breast-tumour expression contrasts was tested by Fisher’s exact test. Contrasts are grouped by phenotype axis; ref. numbers correspond to manuscript references.

**High grade (n = 14 contrasts)**

| **External tumor contrast** | **Fisher’s exact-test P** | **Ref.** |
| --- | --- | --- |
| SBR grade 3 vs 1 | 9.8E-23 | [6] |
| SBR grade 3 vs 1 | 3.3E-23 | [38] |
| SBR grade 3 vs 1 | 4.7E-26 | [39] |
| SBR grade 3 vs 1 | 3.5E-26 | [40] |
| Elston grade 3 vs 1 | 2.1E-24 | [41] |
| Elston grade 3 vs 1 | 6.6E-25 | [42] |
| Elston grade 3 vs 1 | 1.1E-23 | [43] |
| Elston grade 3 vs 1 | 1.1E-20 | [44] |
| Histological grade 3 vs 1 | 3.6E-20 | [45] |
| Histological grade 3 vs 1 | 1.3E-18 | [46] |
| Histological grade 3 vs 1 | 4.2E-21 | [47] |
| Histological grade 3 vs 1 | 2.8E-22 | [48] |
| Histological grade 3 vs 1 | 2.5E-29 | [49] |
| Histological grade 3 vs 1 | 2.5E-32 | [50] |

**Poor prognosis (n = 9 contrasts)**

| **External tumor contrast** | **Fisher’s exact-test P** | **Ref.** |
| --- | --- | --- |
| Tumor 2-5 cm vs <2 cm | 7.6E-22 | [51] |
| Tumor 2-5 cm vs <2 cm | 1.6E-8 | [47] |
| DFS <31 mo vs >72 mo | 1.4E-13 | [47] |
| DFS 2 yr vs >4 yr | 5.9E-19 | [40] |
| OS <5yr vs >10 yr | 8.4E-13 | [42] |
| LN mets vs no mets | 2.8E-9 | [42] |
| Relapse or mets vs no | 3.1E-8 | [39] |
| Relapse vs no | 4.8E-15 | [44] |
| Distant mets vs no | 6.1E-16 | [49] |

**Basal-like (n = 6 contrasts)**

| **External tumor contrast** | **Fisher’s exact-test P** | **Ref.** |
| --- | --- | --- |
| Basal vs normal-like | 2.6E-26 | [44] |
| Basal vs normal | 3.3E-33 | [38] |
| Basal vs normal subtype | 2.3E-17 | [52] |
| Basal vs normal subtype | 1.3E-65 | [50] |
| Luminal-A vs basal subtype | 2.0E-20 | [48] |
| Basal vs normal | 4.6E-30 | [40] |

**Table S5. Menopause-dependent effect modification (feature×proximity interactions) for baseline NanoString features predicting involution pace**

Each row reports results from an ordinary least squares interaction model: pace ~ feature + proximity-to-menopause + feature×proximity + follow-up interval + baseline lobular area + baseline acini. All continuous predictors (including pace) were z-scored. The interaction coefficient (β_int) tests whether the feature→pace slope changes with menopausal proximity. ‘Simple slopes’ are estimated at prelike (+1 SD proximity) and postlike (−1 SD proximity) states. FDR q-values were computed across features using the Benjamini–Hochberg procedure.

**Column definitions**

**Feature:** Human-readable program/anchor name.

**Category:** Prespecified composite score, single-gene anchor, or derived module.

**n:** Number of women included in the model for that feature (complete cases).

**β_int:** Coefficient for the feature×proximity interaction term (standardized units).

**p_int:** Two-sided p-value for β_int.

**q(FDR):** Benjamini–Hochberg FDR-adjusted p-value for β_int across the tested features.

**Slope_pre:** Estimated feature→pace slope at +1 SD proximity-to-menopause (‘prelike’).

**p_pre:** Two-sided p-value for Slope_pre.

**Slope_post:** Estimated feature→pace slope at −1 SD proximity-to-menopause (‘postlike’).

**p_post:** Two-sided p-value for Slope_post.

**Flip-point (years):** Approximate proximity-to-menopause value (in years; + = pre, − = post) at which the fitted feature→pace slope crosses 0.

**R²:** Model R² (variance explained) for the full interaction model.

| **Feature** | **Category** | **n** | **β_int** | **p_int** | **q(FDR)** | **Slope_pre** | **p_pre** | **Slope_post** | **p_post** | **Flip-point (years)** | **R²** |
| --- | --- | --- | --- | --- | --- | --- | --- | --- | --- | --- | --- |
| EGFR–STAT3–innate hub (+inflammatory drive; Hub12) | Derived module | 71 | -0.405 | 1.5e-05 | 0.0002 | -0.287 | 0.0460 | +0.524 | 1.9e-05 | +0.81 | 0.467 |
| EGFR–STAT3–innate hub (Hub9) | Derived module | 71 | -0.374 | 0.0006 | 0.0045 | -0.291 | 0.0757 | +0.457 | 0.0006 | +0.27 | 0.389 |
| SASP core (prespecified composite) | Composite program | 71 | -0.381 | 0.0010 | 0.0045 | -0.309 | 0.0639 | +0.453 | 0.0013 | +0.01 | 0.374 |
| Cytokine/STAT3-SASP (derived module) | Derived module | 71 | -0.292 | 0.0012 | 0.0076 | -0.155 | 0.2756 | +0.367 | 0.0039 | +1.68 | 0.362 |
| STAT3 axis (derived module) | Derived module | 71 | -0.350 | 0.0002 | 0.0103 | -0.137 | 0.3787 | +0.428 | 0.0008 | +2.55 | 0.373 |
| TOP2A (anchor) | Single-gene anchor | 71 | +0.330 | 0.0082 | 0.0191 | +0.325 | 0.0654 | -0.336 | 0.0299 | -1.32 | 0.319 |
| Proliferation (prespecified composite) | Composite program | 71 | +0.135 | 0.2039 | 0.3880 | +0.007 | 0.9625 | -0.263 | 0.0800 | +5.87 | 0.274 |
| Matrix remodeling (derived module) | Derived module | 71 | -0.104 | 0.2307 | 0.3880 | -0.178 | 0.2871 | +0.030 | 0.8001 | -6.95 | 0.255 |
| FCGR3A/B (CD16; anchor) | Single-gene anchor | 71 | -0.089 | 0.2649 | 0.3880 | -0.023 | 0.8894 | +0.154 | 0.2264 | +4.26 | 0.262 |
| MKI67 (anchor) | Single-gene anchor | 71 | +0.114 | 0.2772 | 0.3880 | +0.005 | 0.9787 | -0.223 | 0.1017 | +5.98 | 0.270 |
| Myeloid activation (prespecified composite) | Composite program | 71 | +0.034 | 0.6378 | 0.7784 | +0.279 | 0.1454 | +0.212 | 0.0731 | -58.02 | 0.277 |
| EGFR ligands (prespecified composite) | Composite program | 71 | +0.038 | 0.6672 | 0.7784 | -0.037 | 0.8071 | -0.114 | 0.4262 | +13.66 | 0.245 |
| EREG (anchor) | Single-gene anchor | 71 | -0.036 | 0.7780 | 0.8378 | -0.068 | 0.6765 | +0.004 | 0.9805 | -8.27 | 0.240 |
| EGFR field (derived module) | Derived module | 71 | +0.059 | 0.5411 | 0.9638 | -0.077 | 0.6086 | -0.070 | 0.5970 | -158.93 | 0.242 |

**Table S6. Gene membership and provenance of NanoString-derived program scores and anchor transcripts**

Composite scores were computed from baseline NanoString IO360/BC360 data after log1p transformation, cohort z-scoring of each gene, and averaging within functional groups (requiring ≥50% of genes, minimum 2, to be present). Single-gene anchors were analyzed separately.

| **Feature (score/anchor)** | **Program / marker** | **Category** | **Gene membership** | **Selection basis / rationale** | **Orthogonal alignment** | **Notes** |
| --- | --- | --- | --- | --- | --- | --- |
| Proliferation_score | Proliferation program | Prespecified composite score | MKI67, TOP2A, PCNA, MCM2, CCNB1, CCNE1 CDC20, AURKA, BIRC5, MAD2L1, UBE2C, CENPF CCNA2 | Canonical cell-cycle genes commonly used as breast epithelial proliferation markers; present on IO360/BC360; designed to align with orthogonal proliferation assays (Ki67/TOP2A IHC/MxIF). | Ki67 and TOP2A IHC/MxIF. |  |
| Myeloid_score | Myeloid program | Prespecified composite score | CD14, CSF1R, ITGAM | Core monocyte/macrophage lineage markers with high cell-type specificity; restricted to minimize confounding by broadly induced inflammatory genes. | Macrophage / innate spatial panels (CD16; CD68/CD206) |  |
| EGFR_ligands_score | EGFR ligand field | Prespecified composite score | EREG, AREG, EGFR | EGFR ligands plus receptor represented on IO360/BC360 to capture paracrine growth factor field (ligand availability + receptor competence). | EREG MxIF; hub localization |  |
| SASPcore_score | SASP core | Prespecified composite score | IL6, CXCL8, MMP1, MMP3, EREG | Panel-available 'core' SASP spanning inflammatory cytokine/chemokine (IL6, CXCL8), matrix remodeling (MMP1, MMP3), and growth-factor output (EREG), avoiding an ad hoc 'kitchen sink' set. | p16/EREG spatial hubs; pSTAT3 hubs |  |
| EREG_z_log1p | EREG (epiregulin) | Single-gene anchor | EREG | EGFR ligand central to senescence-associated epithelial fields in later spatial assays; highlighted in extreme-group contrasts. | EREG MxIF |  |
| FCGR3A/B_z_log1p | FCGR3A/B (CD16) | Single-gene anchor | FCGR3A/B | Widely used marker for FcγR-bearing innate effector cells; directly corresponds to CD16 spatial marker. | CD16 MxIF |  |
| MKI67_z_log1p | MKI67 | Single-gene anchor | MKI67 | Canonical proliferation marker; aligns with Ki67 IHC/MxIF. | Ki67 IHC/MxIF |  |
| TOP2A_z_log1p | TOP2A | Single-gene anchor | TOP2A | Canonical proliferation marker; aligns with TOP2A IHC/MxIF and is prominent in the postmenopausal proliferation signature. | TOP2A IHC/MxIF |  |
| STAT3_axis_score | STAT3 axis | Derived module | IL6, JAK1, STAT3, SOCS3 | Minimal 'pathway spine' summarizing STAT3 signaling (ligand → kinase → TF → feedback target). | pSTAT3 MxIF |  |
| Cytokine_SASP_score | Cytokine/STAT3-SASP | Derived module | IL6, CXCL8, IL1A, IL1B, TNF, CXCL1 CXCL2, SOCS3 | Cytokine/chemokine-heavy SASP arm expected to couple to STAT3 activation and inflammatory drive. | pSTAT3 hubs |  |
| Matrix_remodel_score | Matrix remodeling | Derived module | MMP1, MMP3, MMP9 | Panel-available matrix remodeling enzymes, capturing extracellular-matrix remodeling component of SASP/involution. | Remodeling context for involution pace. |  |
| EGFR_field_score | EGFR field (expanded) | Derived module | EREG, AREG, EGF, EGFR | Expanded EGFR ligand field module including EGF (in addition to prespecified EGFR ligands score). | EREG spatial field |  |
| Hub9_score | EGFR–STAT3–innate hub (Hub9) | Derived module | EREG, AREG, EGF, EGFR, IL6, JAK1 STAT3, SOCS3, FCGR3A/B | Union of EGFR field + STAT3 axis + innate CD16 anchor to operationalize a transcript-to-spatial hub bridge. | pSTAT3/CD16 hubs |  |
| Hub12_score | EGFR–STAT3–innate hub + inflammatory drive (Hub12) | Derived module | EREG, AREG, EGF, EGFR, IL6, JAK1 STAT3, SOCS3, FCGR3A/B, IL1A, IL1B, CXCL8 | Hub9 plus minimal inflammatory 'drive' genes (IL1A/IL1B/CXCL8) feeding into IL6/STAT3 activation. | pSTAT3-centered senescent–innate hubs and EREG fields |  |

**Supplementary Note (Table S6): program provenance and design principles**

Proliferation program (cell-cycle activity). The proliferation score was defined from canonical cell-cycle genes that (i) are consistently used in breast biology as proliferation markers, (ii) are represented on IO360/BC360, and (iii) align with orthogonal proliferation assays (Ki67/TOP2A IHC/MxIF) used elsewhere in the manuscript. This score summarizes epithelial proliferative reserve and reduces sensitivity to any single marker transcript.

Myeloid program (monocyte/macrophage presence). The myeloid score was defined using core monocyte/macrophage lineage markers present on IO360/BC360. We intentionally restricted this program to a small set of high-specificity markers to minimize confounding by broader inflammatory signaling genes that are not cell-type specific.

EGFR ligand field program (paracrine growth factor signaling). The EGFR ligand program was defined as the set of EGFR ligands and the EGFR receptor represented on the panel. This captures the capacity for an epithelial growth factor field (ligand availability plus receptor competence) in a way that is interpretable across transcriptional and spatial assays.

SASPcore program (senescence-associated secretory phenotype). SASP is context-dependent and can include dozens of factors. To avoid ad hoc “kitchen sink” scoring, we defined a core SASP program limited to panel-available genes spanning three canonical SASP functional classes: (i) inflammatory cytokine/chemokine signaling, (ii) matrix remodeling enzymes, and (iii) growth factor/EGFR ligand output. This yields a score with clear biological interpretation and direct relevance to involution remodeling while maintaining robust coverage on IO360/BC360.

STAT3 axis program (inflammatory signaling module). To represent STAT3 signaling with minimal arbitrariness, we used a “pathway spine” design: an upstream ligand, a proximal kinase, the transcription factor, and a canonical transcriptional feedback target (IL6 → JAK1 → STAT3 → SOCS3, where SOCS3 serves as a readout of STAT3-dependent transcriptional output). SOCS3 was included as a transcriptional readout of STAT3 activity, not as a regulator: at the mRNA level its expression rises with recent STAT3 transcriptional output and is summed with the same (positive) sign as the other pathway members, so a transcript-abundance score is not affected by its protein-level negative-feedback role. The menopause-conditioned reversal was unchanged when SOCS3, or any other single member, was removed (leave-one-gene-out, **Fig. S4F**).

EGFR–STAT3–innate hub program (Hub score). The Hub score was defined a priori as the union of: (i) EGFR field genes, (ii) STAT3 axis genes, (iii) a minimal inflammatory “drive” set that feeds into IL6/STAT3 activity, and (iv) an innate Fcγ receptor anchor corresponding to CD16+ effector cells. This score was constructed to operationalize a mechanistic hypothesis supported by prior involution literature implicating epithelial STAT3 activation and to enable a transcript→spatial hub bridge across assays, rather than to maximize association with pace.

Anchors. A small number of single-gene anchors were retained (rather than absorbed into composites) when they (i) serve as widely recognized lineage or ligand markers, and (ii) directly correspond to spatial markers used for orthogonal validation.

Sensitivity / robustness. Key findings for anchors and core programs were stable to alternative Pre→Post definitions, follow-up restrictions, and influence checks (Supplementary Methods).
