# Supplementary material for: Menopausal timing and senescent–immune coupling in age-related lobular involution of the human breast: a longitudinal cohort study
Source: eBioMedicine. 2026 Aug 31;131:106463. doi: 10.1016/j.ebiom.2026.106463 (PMC13560535; doi:10.1016/j.ebiom.2026.106463)
Supplement: Supplementary file 3 [file mmc3.docx]

# Key Resources and Reagents Table with RRIDs

## Primary antibodies: endpoint, triad, and follow-up panels

| **Marker / target** | **Primary antibody** | **Vendor** | **Catalogue no.** | **Clone / host / clonality** | **Manuscript use** | **Working dilution(s)** | **RRID** |
| --- | --- | --- | --- | --- | --- | --- | --- |
| Ki67 | Monoclonal Mouse Anti-Human Ki-67 Antigen, clone MIB-1 | Dako / Agilent | M7240 | MIB-1; mouse monoclonal | IHC; postmenopausal triad MxIF; longitudinal baseline panels 1–2; follow-up MxIF | IHC: 1:200; triad 1:200; baseline/follow-up 1:100 | RRID:AB_2142367 |
| p16 / CDKN2A | CDKN2A/p16 Antibody (JC8) | Santa Cruz Biotechnology | sc-56330 | JC8; mouse monoclonal | Postmenopausal triad MxIF; longitudinal baseline panels 1–3; follow-up MxIF | Triad 1:600; baseline panel 1 1:400; baseline panel 2 1:400; baseline panel 3 1:600; follow-up 1:600 | RRID:AB_785018 |
| TOP2A | Anti-TOP2A antibody | Invitrogen | MA5-32096 | Rabbit monoclonal | IHC; postmenopausal triad MxIF | IHC: 1:200; triad 1:1200 | RRID:AB_2809389 |
| CD45 / PTPRC | Anti-CD45 antibody [EP322Y] | Abcam | ab40763 | EP322Y; rabbit monoclonal / RabMAb | Postmenopausal triad MxIF; longitudinal baseline panel 1; follow-up MxIF | 1:300 | RRID:AB_726545 |
| Pan-cytokeratin | Monoclonal Mouse Anti-Human Cytokeratin, clones AE1/AE3 | Dako / Agilent | M3515 | AE1/AE3; mouse monoclonal | Epithelial compartment mask in all MxIF panels | Triad/baseline panel 3/follow-up 1:150; baseline panels 1–2 1:100 | RRID:AB_2132885 |

## Primary antibodies: baseline hub and macrophage panels

| **Marker / target** | **Primary antibody** | **Vendor** | **Catalogue no.** | **Clone / host / clonality** | **Manuscript use** | **Working dilution(s)** | **RRID** |
| --- | --- | --- | --- | --- | --- | --- | --- |
| EREG / Epiregulin | Epiregulin Antibody (C-9) | Santa Cruz Biotechnology | sc-376284 | C-9; mouse monoclonal IgG2b kappa | Longitudinal baseline MxIF panels 1–2 | 1:100 | RRID:AB_11014189 |
| Phospho-STAT3 | Anti-STAT3 (phospho Y705) antibody [EP2147Y] | Abcam | ab76315 | EP2147Y; rabbit monoclonal / RabMAb | Longitudinal baseline MxIF panel 2 | 1:100 | RRID:AB_1658549 |
| CD16 / CD16A/FCGR3A | Anti-CD16 antibody [EPR22409-124] | Abcam | ab246222 | EPR22409-124; rabbit monoclonal / RabMAb | Longitudinal baseline MxIF panel 2 | 1:500 | RRID:AB_2910166 |
| CD206 / MRC1 | Anti-CD206 / Mannose Receptor antibody | Abcam | ab64693 | Rabbit polyclonal | Longitudinal baseline macrophage MxIF panel 3 | 1:200 | RRID:AB_1523910 |
| CD68 | Anti-CD68 antibody | Abcam | ab125212 | Rabbit polyclonal | Longitudinal baseline macrophage MxIF panel 3 | 1:500 | RRID:AB_10975465 |

## Detection reagents, dyes, kits, and assay resources

| **Resource** | **Vendor** | **Catalogue no.** | **Use** | **RRID** |
| --- | --- | --- | --- | --- |
| Opal 480 TSA reagent | Akoya Biosciences | FP1500001KT | MxIF fluorophore | Not assigned |
| Opal 520 TSA reagent | Akoya Biosciences | FP1487001KT | MxIF fluorophore | Not assigned |
| Opal 570 TSA reagent | Akoya Biosciences | FP1488001KT | MxIF fluorophore | Not assigned |
| Opal 620 TSA reagent | Akoya Biosciences | FP1495001KT | MxIF fluorophore | Not assigned |
| Opal 690 TSA reagent | Akoya Biosciences | FP1497001KT | MxIF fluorophore | Not assigned |
| Opal 780 TSA reagent | Akoya Biosciences | FP1501001KT | MxIF fluorophore | Not assigned |
| DAPI nuclear counterstain | Akoya Biosciences | FP1490 | MxIF nuclear counterstain | Not assigned |
| Opal HRP secondary/polymer reagent | Akoya Biosciences | ARH1001EA | TSA detection step | Not assigned |
| Citrate antigen retrieval buffer, pH 6.0 | Akoya Biosciences | AR6001KT | IHC antigen retrieval | Not assigned |
| RNeasy FFPE Kit | Qiagen | 73504 | FFPE RNA extraction | Not assigned |

## Software and databases

| **Instrument / software / database** | **Use in manuscript** | **Vendor / source** | **RRID / identifier** |
| --- | --- | --- | --- |
| QuPath v0.6.0 | IHC ROI analysis / digital pathology quantification | Open-source QuPath | RRID:SCR_018257 |
| Vectra Polaris multispectral imaging system | MxIF slide scanning | Akoya Biosciences / Revvity | RRID:SCR_025508 |
| inForm Tissue Analysis Software v3.1.0 | Spectral unmixing, autofluorescence subtraction, segmentation | Akoya Biosciences / Revvity | RRID:SCR_019155 |
| GenomeStudio 2011.1 | DASL expression preprocessing | Illumina | RRID:SCR_010973 |
| nCounter Analysis System v6.0.2 | NanoString profiling | NanoString / Bruker Spatial Biology | RRID:SCR_021712 |
| GSEAPreranked / GSEA v4.4.0 | Preranked gene-set enrichment | Broad Institute | GSEA RRID:SCR_003199 |
| MSigDB Hallmark gene sets v2026.1 | Gene-set database | Broad Institute | RRID:SCR_016863 |
| R Project for Statistical Computing v4.6.1 | Statistical analyses | R Project | RRID:SCR_001905 |
| brms v2.23.0 | Bayesian hierarchical clearance-hub model | CRAN | RRID:SCR_023862 |
| Stan v2.39.0 | Bayesian model backend | Stan Development Team | RRID:SCR_018459 |
